# Biocatalytic Construction of Chiral Pyrrolidines and Indolines via Intramolecular C(sp^3^)–H Amination

**DOI:** 10.1021/acscentsci.3c00516

**Published:** 2023-12-14

**Authors:** Zi-Yang Qin, Shilong Gao, Yike Zou, Zhen Liu, James B. Wang, Kendall N. Houk, Frances H. Arnold

**Affiliations:** † Division of Chemistry and Chemical Engineering, California Institute of Technology, Pasadena, California 91125, United States; ‡ Department of Chemistry and Biochemistry, University of California, Los Angeles, California 90095, United States

## Abstract

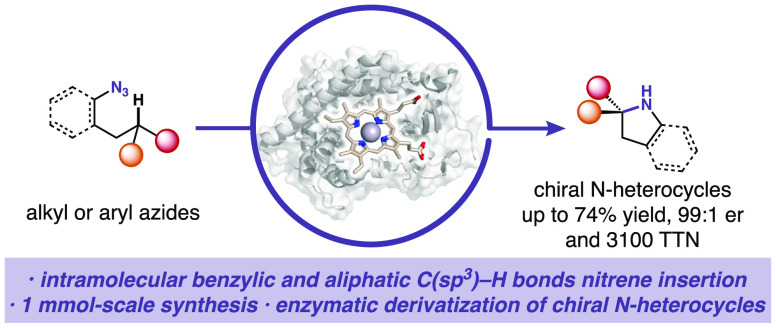

Nature harnesses exquisite enzymatic cascades to construct *N*-heterocycles and further uses these building blocks to assemble the molecules of life. Here we report an enzymatic platform to construct important chiral *N*-heterocyclic products, pyrrolidines and indolines, via abiological intramolecular C(sp^3^)–H amination of organic azides. Directed evolution of cytochrome P411 (a P450 enzyme with serine as the heme-ligating residue) yielded variant **P411-PYS-5149**, capable of catalyzing the insertion of alkyl nitrene into C(sp^3^)–H bonds to build pyrrolidine derivatives with good enantioselectivity and catalytic efficiency. Further evolution of activity on aryl azide substrates yielded variant **P411-INS-5151** that catalyzes intramolecular C(sp^3^)–H amination to afford chiral indolines. In addition, we show that these enzymatic aminations can be coupled with a P411-based carbene transferase or a tryptophan synthase to generate an α-amino lactone or a noncanonical amino acid, respectively, underscoring the power of new-to-nature biocatalysis in complexity-building chemical synthesis.

## Abstract 2

Engineered enzymatic platform enables the construction of chiral pyrrolidines and indolines via intramolecular alkyl/aryl nitrene C−H insertion.

## Introduction

**Scheme 1:**
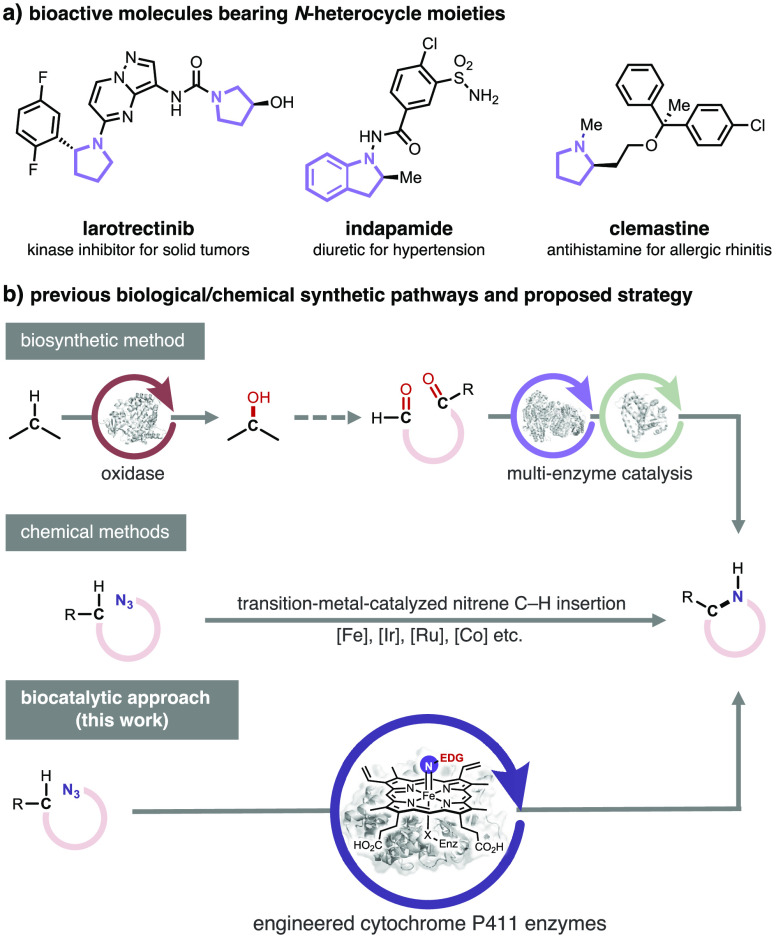
Background and Project Synopsis

*N*-Heterocycles are ubiquitous in functional materials, bioactive natural products, and pharmaceutical compounds (Scheme 1a).^1,2^ Especially privileged are saturated cyclic amines such as pyrrolidines, whose desirable structural and pharmacokinetic/pharmacodynamic (PK/PD) properties help make them one of the most common *N*-heterocyclic moieties in drug molecules.^3−5^ A prevalent biosynthetic and biocatalytic approach to forging chiral cyclic amines is intramolecular condensation of aminoketones or aminoaldehydes followed by reduction using imine reductases.^6−15^ This route requires preoxidation at a given position and preinstalled carbonyl/amino functionality on the substrates. Very recently, Hyster and co-workers demonstrated new-to-nature photoenzymatic hydroamination of olefins to synthesize cyclic amines.^16^ Chemists have been seeking catalytic C–H functionalization methodologies for the synthesis of cyclic amines in order to maximize atom and step economy (Scheme 1b);^17^ transition-metal-catalyzed alkyl nitrene C–H insertion reactions are attractive in this context. Compared to well-established metallonitrenes with electron-withdrawing substituents on the nitrogen, C(sp^3^)–H insertion with alkyl nitrenes is less developed due to lower reactivity and propensity to undergo an unproductive 1,2-hydride shift leading to the irreversible formation of undesired imines.^18^ Another major challenge is to achieve high stereoselectivity;^18,19^ stereoselective examples with an environmentally benign 3d transition metal are even rarer.^20,21^ We posited that importing this human-invented chemistry into metalloenzymes could leverage the remarkable selectivity and sustainability of enzymes to streamline the construction of chiral cyclic amines.

Cytochrome P450 heme monooxygenases utilize molecular oxygen and NAD(P)H to generate a high-valent iron-oxo species^22^ to perform selective C–H functionalization transformations that are challenging for small-molecule catalysts. With tunable protein–substrate interactions in the chiral environment of the active site, these biocatalysts use an earth-abundant metal (iron) and exert exquisite control over oxidation chemistries. Over the past decade, our lab and others have ventured beyond the native oxidation activities of P450s to develop an array of non-natural functions based on transfer of reactive carbene- and nitrene-like intermediates.^23,24^ Enzymatic nitrene transfer reactions have expanded the biocatalytic repertoire to include transformations ranging from C–H sulfamidation to C–H amination/amidation.^25−31^ However, heme enzymes have not been demonstrated to employ more challenging nitrene species bearing electron-donating substitutions in asymmetric C–H functionalization processes. We thus set out to tackle this challenge with a heme enzyme to perform cyclic amine synthesis.

## Results and Discussion

**Figure 1:**
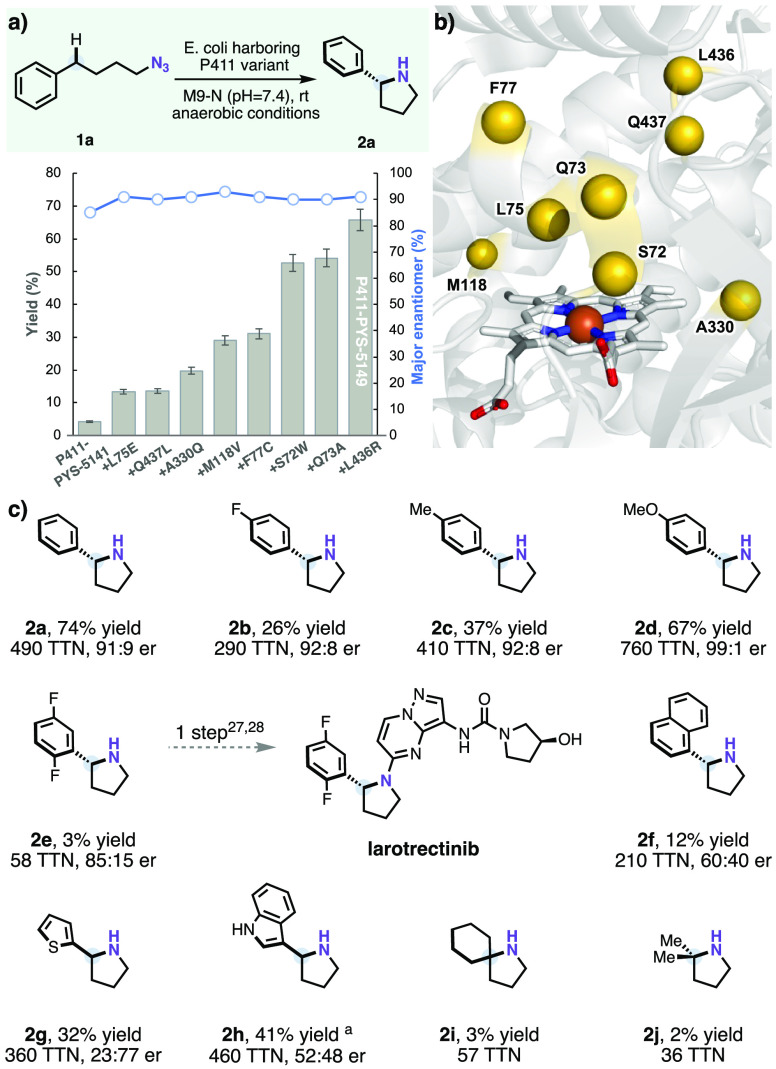
(a) Directed evolution for enantioselective alkyl nitrene transfer to make pyrrolidine **2a**. Cytochrome P411 variant **P411-PYS-5149** was obtained after eight rounds of site-saturation mutagenesis and screening from **P411-PYS-5141** (see Section IX for sequences). Indicated mutations are relative to **P411-PYS-5141**. Experiments were performed using *E. coli* (OD_600_ = 30) expressing P411 enzymes with 2.5 mM substrate **1a** in M9-N buffer (pH = 7.4) at room temperature under anaerobic conditions for 16 h. Yields were quantified by liquid chromatography–mass spectrometry (LC-MS) based on the calibration curve of **2a**. Enantioselectivities were measured by high-performance liquid chromatography (HPLC) on a chiral phase after benzoyl protection. (b) Mutated residues (S72, Q73, L75, F77, M118, A330, L436, and Q437) are highlighted in the active site of P411 variant **E10** (PDB ID: 5UCW). (c) Substrate scope of enantioselective alkyl nitrene transfer. Experiments were performed at analytical scale using *E. coli* (OD_600_ = 30) that expressed the **P411-PYS-5149** variant with a 2.5 mM substrate (**1a**–**j**) in M9-N buffer (pH = 8.4) at room temperature under anaerobic conditions for 16 h. Yields were quantified by LC-MS based on the calibration curves of the corresponding reference products. Enantioselectivities were measured by HPLC on a chiral phase after benzoyl protection of the pyrrolidine products. ^a^**2h** was obtained in 60% yield, 51:49 er with variant **P411-PYS-5148**.

Azides, readily prepared from alcohols and bromides, serve as atom-economical nitrene precursors for making the corresponding pyrrolidines because they eliminate only nitrogen gas.^32^ We commenced this investigation with model substrate (4-azidobutyl)benzene **1a**. A panel of hemoproteins previously engineered for other nitrene and carbene transfer reactions was screened for activity on **1a** as whole *Escherichia coli* cell catalysts. To our delight, a P411 (cytochrome P450 having a Ser in place of Cys as the heme axial ligand) variant engineered for carbene C–H insertion was able to catalyze the desired transformation with a 4% yield and moderate enantioselectivity (82:18 er). Control studies showed that free heme had no activity in this reaction. This variant, renamed **P411-PYS-5141** (see Section IX for sequence details), was subjected to four rounds of site-saturation mutagenesis (SSM) and screening to accumulate beneficial mutations L75E, Q437L, A330Q, and M118 V, which improved the yield to 29% and the enantioselectivity to 94:6 er. Four additional rounds of evolution led to variant **P411-PYS-5149** with 74% yield and slightly decreased enantioselectivity (91:9 er) (Figures 1a and 1b). The absolute configuration of the pyrrolidine product **2a** was confirmed as *R* by comparing the enzymatically produced product with the commercially available enantiomer (*S*)-**2a** after benzoyl protection (see Section VIII in the Supporting Information).

Evaluating the substrate scope of **P411-PYS-5149** for pyrrolidine synthesis (Figure 1c), we found that substrates *para*-fluoro **1b**, *para*-methyl **1c**, and *para*-methoxyl **1d** generally afforded the corresponding pyrrolidine products in moderate to good yield and enantioselectivity (up to 67% yield and 99:1 er). The *ortho*-methyl substrate was not well-tolerated in this system, however, presumably due to steric hindrance between *ortho* substituents and the porphyrin (Figures S2 and S7). Variant **P411-PYS-5149** also showed promising initial activity (3% yield, 85:15 er) with 2,5-difluoro substrate **1e**, which serves as a building block for the drug molecule larotrectinib;^33,34^ further evolution for activity on **1e** should improve this activity. Other substrates bearing different aromatic rings, such as naphthalene (**1f**), thiophene (**1g**), and indole (**1h**), were also compatible. Surprisingly, variant **P411-PYS-5149** failed to provide high enantioselectivity (bonds indicate uncertainty regarding the absolute configuration) for thiophene **1g** and indole substrate **1h**, even though the yields were moderate (32% and 41%, respectively). We also investigated more challenging substrates with unactivated C(sp^3^)–H bonds. Azidobutylcyclohexane **1i** and azido-4-methylpentane **1j** afforded small amounts (2–3% yield) of product cyclized at the tertiary C–H position. It is likely that these promising activities can be improved to synthetically useful levels by further enzyme engineering, as has been demonstrated many times.^35−37^

While evolving the cyclic amine synthases, we also investigated the ability of the evolved enzymes to forge indolines, another important class of bioactive *N*-heterocycles.^38,39^ Aryl azides have been used as nitrene precursors to synthesize indolines via nitrene C–H insertion, but rarely in an asymmetric manner.^40^ We utilized 1-azido-2-propylbenzene **3a** as the starting material for the initial enzyme screening. In contrast to pyrrolidine synthesis via insertion of alkyl metallonitrene into activated benzylic C–H bonds, indoline synthesis requires aryl metallonitrene insertion into unactivated aliphatic C–H bonds.

**Figure 2:**
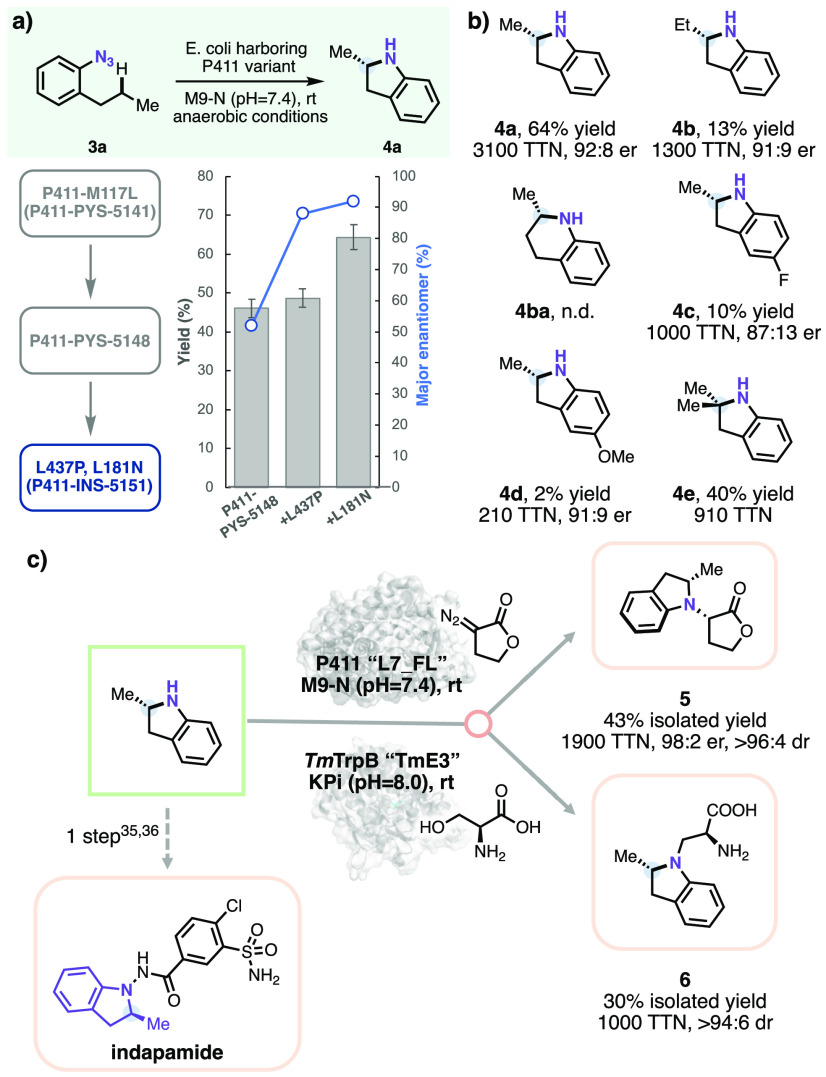
(a) Directed evolution for enantioselective aryl nitrene transfer: evolutionary trajectory for synthesis of methylindoline **4a**. **P411-INS-5151** was obtained after two rounds of site-saturation mutagenesis and screening starting from **P411-PYS-5148** (see Table S2 for sequence details). Indicated mutations are relative to **P411-PYS-5148**. Experiments were performed using *E. coli* (OD_600_ = 40) expressing enzymes with 5.0 mM substrate **3a** in M9-N buffer (pH = 7.4) at room temperature under anaerobic conditions overnight. Buffer was switched to M9-N (pH = 8.4) after condition optimization on **P411-INS-5151**. Yields were quantified by LC-MS based on the calibration curve of **4a**. Enantioselectivities were measured by HPLC in a chiral phase. (b) Substrate scope of the enantioselective aryl nitrene transfer reaction. Experiments were performed at analytical scale using *E. coli* (OD_600_ = 40) that expressed **P411-INS-5151** with 5 mM substrate (**3a**–**d**) in M9-N buffer (pH = 8.4) at room temperature under anaerobic conditions for 16 h. Yields were quantified by LC-MS based on the calibration curves of the corresponding reference products. Enantioselectivities were measured by HPLC on a chiral phase. Reactions with **3e** were performed at a 1 mmol scale using the same conditions as the analytical-scale reaction, and the yield was an isolated yield. (c) Further enzymatic elaboration of enzymatically produced methylindoline. Experiments were performed using *E. coli* (OD_600_ = 30) expressing the P411 enzyme with 10 mM substrate **4a** in M9-N buffer (pH = 7.4) at room temperature under anaerobic conditions overnight. The experiment with tryptophan synthase was performed using purified enzyme with 10 mM **4a** in phosphate buffer (0.1 M, pH = 8.0) at room temperature overnight.

Upon testing the lineage of “pyrrolidine synthases”, we found that variant **P411-PYS-5148** afforded the best yield (46%), although with negligible enantioselectivity (48:52 er) for indoline synthesis from **3a**. **P411-PYS-5148** was used for directed evolution of an “indoline synthase”. Mutations L437P and L181N introduced during two rounds of mutagenesis and screening generated **P411-INS-5151**, with improved activity and enantioselectivity for indoline formation (64% analytical yield, 60% isolated yield, and 92:8 er) (Figure 2a). The absolute configuration of the methylindoline product **4a** was confirmed as *S* by comparing the enzymatic product with the commercially available enantiomer (*R*)-**4a** (see Section VIII in the Supporting Information).

We proceeded to probe the scope of the enzymatic indoline synthesis (Figure 2b). Cyclization of 1-azido-2-butylbenzene **3b** using evolved enzyme generated **P411-INS-5151** predominantly afforded a five-membered heterocycle, ethylindoline **4b**, with 13% yield and good selectivity (91:9 er). A plausible six-membered tetrahydroquinoline product (**4ba**) through C–H insertion at the subterminal site was not detected. A substrate bearing a longer carbon chain (1-azido-2-pentylbenzene) also showed initial activity (Table S4). Substitutions on phenyl groups such as **3c** and **3d** yielded the corresponding indoline products with good enantioselectivities. Finally, a 1.0 mmol scale enzymatic reaction using substrate 1-azido-2-isobutylbenzene **3e** bearing a tertiary C–H bond gave **4e** in 40% isolated yield.

As versatile synthetic intermediates, *N*-heterocycles can be converted into various high-value-added compounds (for example, methylindoline product **4a** can be used in the synthesis of indapamide (Figure 2c)).^41,42^ To demonstrate the utility of the engineered enzymes in constructing synthetically challenging molecules, we conducted two biocatalytic derivatization reactions, one to synthesize an α-amino ester and another to make a noncanonical amino acid, both bearing two chiral centers. Cytochrome P411 variant **L7_FL** from a carbene N–H insertion lineage^43^ accepted methylindoline **4a** and a lactone-based diazo substrate in the form of whole-cell catalysts to provide the enantioenriched α-amino ester **5** in good yield with excellent enantioselectivity and diastereoselectivity (47% yield, 98:2 er, and >96:4 dr). In a preparative-scale reaction, the enzyme afforded α-amino ester **5** as a white solid in 43% yield with identical selectivities. In another demonstration, *Thermotoga maritima* tryptophan synthase (*Tm*TrpB) variant **TmE3** from a ketone alkylation lineage^44^ coupled **4a** and l-serine to provide the *N*-alkylated noncanonical amino acid **6** in 30% isolated yield with excellent diastereoselectivity (>94:6 dr). These biocatalytic derivatization reactions using engineered enzymes demonstrate how complexity can be built rapidly and under mild conditions for the asymmetric synthesis of *N*-heterocycle-containing molecules.

**Figure 3:**
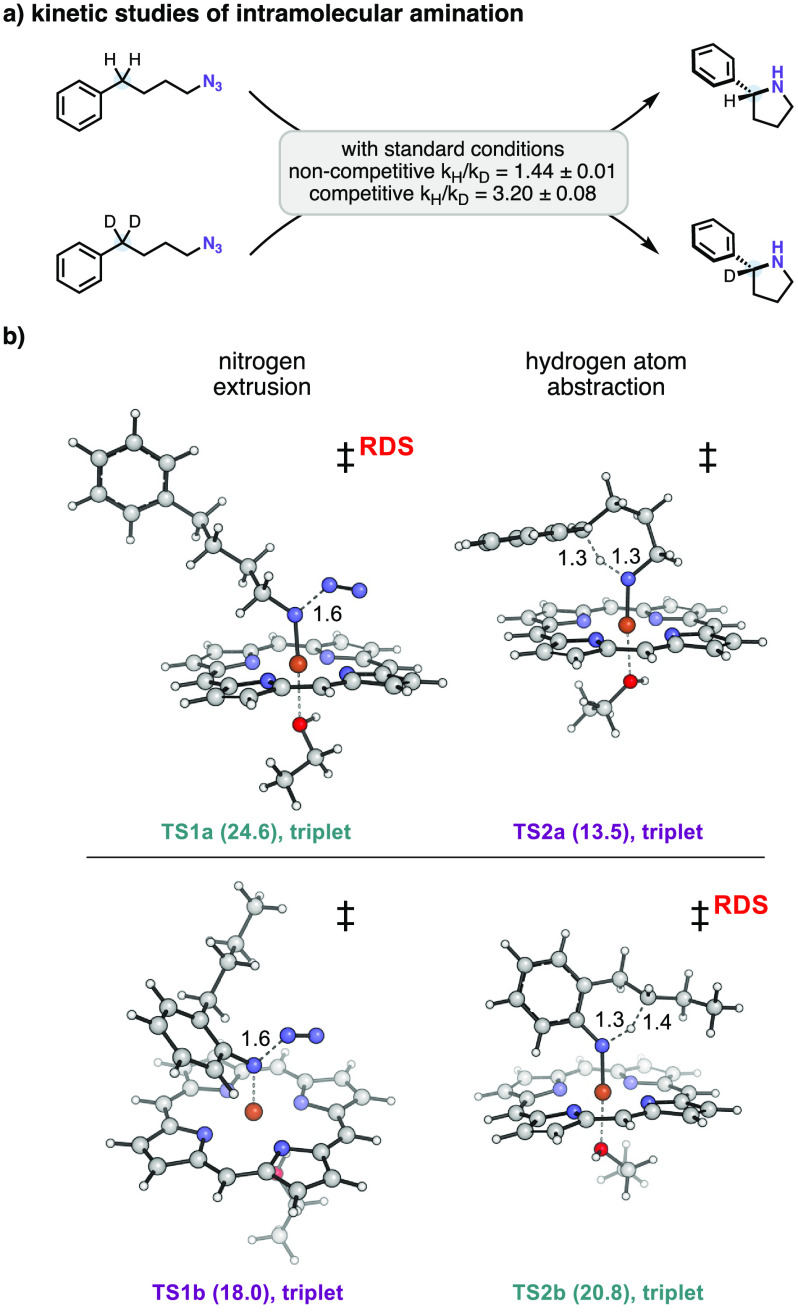
DFT-computed transition states for iron(II)-porphyrin-catalyzed intramolecular alkyl nitrene C(sp^3^)–H amination using substrates **2a** and **4b**. Gibbs free energies are obtained at the B3LYP(D3)/def2tzvp/CPCM(Et_2_O)//B3LYP/6-31G(d),SDD(Fe)/CPCM(Et_2_O) level of theory, all in triplet state, and are given in kcal·mol^–1^. Key distances are given in Å.

To better understand the alkyl/aryl nitrene C(sp^3^)–H insertion processes, density functional theory (DFT) calculations were carried out on a model iron-porphyrin system using substrates **1a** (Figure S8) and **3b** (Figure S9).^45^ In agreement with previous results for enzymatic nitrene insertion reactions, transformations for both **1a** and **3b** proceed via three general steps (see Figures S8 and S9 for the full energy profiles): (1) nitrene formation and nitrogen extrusion, (2) hydrogen atom abstraction to initially generate a carbon-centered radical, and (3) radical rebound to form corresponding products **2a** and **4b**.^25,46^ Interestingly, for alkyl azide substrate **1a**, the nitrogen extrusion step is computed to have a high energy barrier, reflecting the difficulty of activating alkyl azides to eliminate nitrogen at room temperature in a truncated system (Figure 3b, 24.6 kcal/mol vs the subsequent HAT transition state of 13.5 kcal/mol).^26,46^ Experimental observation reveals a noncompetitive kinetic isotopic effect (KIE) of 1.4 and a competitive KIE of 3.2 (Figure 3a), which also indicates the HAT step might not be involved in the rate-determining step.

For the aryl azide substrate, this is reversed (Figure 3b). The HAT step (20.8 kcal/mol) has a higher barrier than the nitrogen extrusion step (18.0 kcal/mol); this is due to the 10 kcal/mol greater stability of the iron-aryl nitrene intermediate vs the iron-alkyl nitrene intermediate. Thus, the aryl nitrene species is both easier to form and less reactive. There is no significant barrier to cyclization of the diradical intermediate to a cyclic amine in either case. We docked each transition state with the iron-porphyrin coenzyme into the enzyme structure and performed 1 μs molecular dynamics simulations with the whole enzyme and a water box to obtain an equilibrium structure of the transition state and the catalytic active site. These simulations indicate that no single residue contributes strong stabilizing interactions; the preferred enantiomeric transition state shape is complementary to the active site, while the antipodal transition state experiences significant destabilizing steric clashes (see Figure S10).

## Conclusion

In conclusion, we have demonstrated new-to-nature enzyme-catalyzed intramolecular C(sp^3^)–H amination via alkyl/aryl nitrene intermediates to forge two key classes of chiral *N*-heterocycles from simple azide precursors. This work represents the first example of enzymatic intramolecular alkyl/aryl nitrene C(sp^3^)–H insertion reactions. Using directed evolution, we engineered two P411 variants, **P411-PYS-5149** and **P411-INS-5151**, which can catalyze pyrrolidine and indoline synthesis, respectively, in moderate to good efficiency and selectivity (up to 74% yield and 99:1 er). These new biocatalysts can be coupled with other biocatalytic transformations to construct complex molecules. DFT calculations suggest that the selectivity is controlled by the binding pose of substrates inside the enzymes’ active sites. This biocatalytic platform provides a general and concise route for the preparation of chiral *N*-heterocycles. More importantly, it lays a foundation for future work toward the biocatalytic construction of *N*-heterocycles of different sizes and intermolecular alkyl and aryl nitrene C–H insertion reactions. We envision that this alkyl/aryl nitrene C–H insertion system can be used to prepare chiral *N*-heterocyclic building blocks for synthetic chemistry and drug discovery.

## Supporting Information Available

Materials, experimental methods, compound characterization data, computational methods, and supplementary data (PDF)
