# Supplementary material for: Biocatalytic Construction of Chiral Pyrrolidines and Indolines via Intramolecular C(sp3)–H Amination
Source: ACS Cent Sci. 2023 Dec 14;9(12):2333–8. doi: 10.1021/acscentsci.3c00516 (PMC10755850; doi:10.1021/acscentsci.3c00516)
Supplement: Supplementary file 1 — oc3c00516_si_001.pdf [file oc3c00516_si_001.pdf]

Supporting Information

Biocatalytic Construction of Chiral Pyrrolidines and Indolines *via* Intramolecular C(sp^3)–H Amination

Zi-Yang Qin,[†,§] Shilong Gao,[†,§] Yike Zou,[‡,#] Zhen Liu,[†,‖] James B. Wang,[†] K. N. Houk,[‡] Frances H. Arnold*[,†]

[†]Division of Chemistry and Chemical Engineering, California Institute of Technology, Pasadena, California 91125, USA.

[‡]Division of Chemistry and Biochemistry, University of California, Los Angeles, California 90095, USA.

[#]Current address: Lawrence Livermore National Laboratory, Livermore, CA, 94550, USA.

[‖]Current address: National Institute of Biological Sciences, Beijing 102206, China.

[§]These authors contributed equally.

*To whom correspondence should be addressed. E-mail: frances@cheme.caltech.edu

Table of Contents

Biocatalytic Construction of Chiral Pyrrolidines and Indolines *via* Intramolecular C(sp^3)–H Amination 1

I. General Procedures 2

II. Directed Evolution of P411-PYS-5149 and P411-INS-5151 for Intramolecular C(sp^3)–H Amination 6

III. Supporting Experimental Figures and Miscellaneous Experiments 9

IV. Preparation and Characterization of the Alkyl and Aryl Azides 14

V. Preparation and Characterization of the Racemic Standards of Products 19

VI. Analytic Scale Enzymatic Reactions and Calibration Curves for Products 21

VII. Preparative-scale Enzymatic Synthesis 35

VIII. Chiral-phase HPLC Traces 38

IX. Sequence Information 54

X. Computational methods 69

XI. NMR Spectra 98

XII. References 126

I. General Procedures

(A) General

Unless otherwise noted, all chemicals and reagents were obtained from commercial suppliers (Millipore Sigma, VWR, Alfa Aesar, Ambeed, Combi-Blocks, TCI America, and Fisher Scientific) and used without further purification. Silica-gel chromatography was carried out using AMD Silica Gel 60, 230–400 mesh. ^{1}H and ^{13}C NMR spectra were recorded on a Varian Inova 300 MHz or Bruker 400 MHz instrument in $CDCl_3$. Data for ^{1}H NMR are reported as follows: chemical shift (δ ppm), multiplicity (s = singlet, d = doublet, t = triplet, q = quartet, hept = heptet, m = multiplet), coupling constant (Hz), and integration. Chemical reactions were monitored using thin-layer chromatography (Merck 60 silica gel plates) and a UV lamp for visualization. Sonication was performed using a Qsonica Q500 sonicator. High-resolution mass spectra HRMS were acquired from the Caltech Mass Spectral Facility using Field Desorption (FD-MS) with a JEOL AccuTOF GC-Alpha (JMS-T2000GC) mass spectrometer (enabled by funds from DOW next generation instrumentation). High-performance liquid-chromatography mass spectroscopy (HPLC-MS) for analysis was carried out using Agilent 1200 series instruments, with C18 (Kromasil®, 4.6 × 50 mm, 5 µm) columns. Water and acetonitrile containing 0.1% acetic acid were used as eluents. Normal-phase chiral HPLC was performed using Daicel Chiralpak IC column (4.6 × 250 mm, 5 µm) with hexane and isopropanol as the mobile phase. Distilled water was utilized for growth media while double-distilled water was employed for all buffer preparations.

The M9-N buffer described in this study was prepared as follows:

A 5X stock solution was prepared by dissolving Na_2HPO_4 (34 g), KH_2PO_4 (15 g) and NaCl (2.5 g) in 1 L of water followed by sterilization by autoclaving. A 1X stock was then prepared by diluting 200 mL of the 5X solution to 1 L, followed by addition of 1 mL $CaCl_2$ (0.1 M), 2 mL $MgSO_4$ (1.0 M), and adjusting the pH to 7.4 or 8.4 by the addition of 6.0 M aqueous NaOH.

In all cases, commercially available cyclic amines (Sigma, Enamine, Ambeed, Combi-Blocks) were utilized as amine standards without further purification. These were acetylated with benzoyl chloride to give the corresponding acetyl amides for use as amide standards for enantioselectivity.

(B) Cloning, mutagenesis, and expression of enzymes

Expression vector pET22b(+) (Novagen) was used for cloning and expression of all variants described in this paper. Site-saturation mutagenesis was performed using a modified QuikChangeTM mutagenesis protocol using the 22-codon trick.[1] The PCR products were purified with New England Biolabs gel purification kit, and the gaps were repaired using Gibson Mix™.[2] Without further purification, 1 µL of the Gibson product was used to transform 50 µL of electrocompetent *Escherichia coli* cells. Electrocompetent *E. coli* BL21 *E. cloni*® (Lucigen) cells were utilized for all experiments. Luria-Bertani (LB) media were used for growth.

E. coli transformed with pET22b(+) constructs encoding various P411 variants were grown overnight (14 h to 16 h) in 5-mL LB medium supplemented with ampicillin (LB_{amp}). Subsequently, 1 mL of this preculture was used to inoculate 49 mL of Hyper-Broth™ (HB, AthenaES) medium

in a 150-mL Erlenmeyer flask, supplemented with ampicillin (HB_{amp}) and Glucose Mix for expression. The expression culture was incubated at 37 °C and shaken at 220 rpm for 2 hours until the optical cell density at 600 nm (OD_{600}) was ~0.9–1.1. Then, the expression culture was cooled in an ice bath for 45–60 minutes and was treated with 1 mM 5-aminolevulinic acid (ALA) and 0.5 mM isopropyl *β*-D-1-thiogalactopyranoside (IPTG) (final concentrations). Cells were grown at 22 °C and 140 rpm for 20–24 hours, and the shaking radius was 25 mm. Once expression was finished, the cultures were centrifuged (4,000*g*, 6 minutes, 4 °C) and the pellets were resuspended to an OD_{600} of 30–40 (or other appropriate OD as necessary) in M9-N buffer with pH adjusted to 8.4. This cell suspension was utilized to setup whole-cell reactions. Aliquots of the cell suspension (3–4 mL) were used to determine protein concentration after lysis by sonication.

(C) Determination of hemoprotein concentration

The concentration of P411 enzymes in whole-cell experiments was determined from ferrous carbon monoxide binding difference spectra using the previously reported extinction coefficient for serine-ligated enzymes ($\varepsilon = 103{,}000\ M^{-1}\bullet cm^{-1}$).[3] Lysate was obtained by sonication (2 minutes, 1 second on, 1 second off, 30% amplitude, on wet ice). The cell debris was removed by centrifugation (14,000 × g, 10 minutes, 4 °C), and 180 µL of the supernatant were then transferred to a well in a 96-well UV-vis flat bottom plate (measurements were performed in quadruplicate). To each well, 20 µL of freshly prepared 0.5 M sodium dithionite aqueous solution were added. Absorbance measurements were performed on a Tecan Spark instrument before and after CO binding. The following formula was used for estimating protein concentration.

$$[Enzyme]\ (\mu M) = 1.1 * \frac{\Delta 411_{CO-red} - \Delta 490_{CO-red}}{0.74\,cm * 0.103\ \mu M^{-1} cm^{-1}}$$

(D) Analytic reaction setup and product quantification

All biocatalytic reactions were set up in an anaerobic chamber (oxygen level: <40 ppm).

• For pyrrolidine construction, 300 µL of resuspended cells (diluted to OD_{600} = 40 with M9-N minimal buffer, pH = 8.4) were added to 2-mL screw cap vials, followed by addition of D-glucose (90 µL, 500 mM in M9-N buffer), M9-N buffer, and the azide substrate (10 µL of 100 mM stock solution in EtOH). Final concentrations were 2.5 mM alkyl azide and 112.5 mM D-glucose; final reaction volume was 400 µL. The vials were capped and then shaken inside coy chamber (600 rpm) at room temperature for 12–20 hours (overnight). After the reaction was completed and the vials were removed from the shaker, 400 µL of 2.5 mM internal standard (ethyl benzoate) acetonitrile solution were added. The mixture was transferred to a 2.0-mL Eppendorf tube and then subjected to vortexing (20 s × 2) and centrifugation (14,000 × g, 5 min, 4 °C). A sample of the supernatant (0.2 mL) was transferred to a vial with an insert for LC-MS analysis. Products were identified and quantified based on the corresponding reference compounds (**Section V** and **VI**). To further determine the enantiomeric excess (*ee*), the remaining supernatants of three parallel analytical reactions were combined and transferred to a 2-dram vial and the solvent was removed by blowing air. To the remaining residues from air blowing were added benzoyl chloride (60 µL of 1 M stock solution in acetonitrile) and 60 µL saturated $NaHCO_3$ solution. After shaking the vials overnight, the benzoyl-protected pyrrolidines were extracted to a solution of hexane and EtOAc mixture (1:1) and subjected to normal-phase HPLC to determine the *ee*.

• For indoline construction, 360 µL of resuspended cells (diluted to OD_{600} = 45 with M9-N minimal buffer, pH = 8.4) were added to 2-mL screw cap vials, followed by D-glucose (30 µL, 500 mM in M9-N buffer), M9-N buffer the azide substrate (10 µL of 200 mM stock solution in EtOH). Final concentrations were 5.0 mM alkyl azide and 37.5 mM D-glucose; final reaction volume was 400 µL. The vials were capped and then shaken inside coy chamber (600 rpm) at room temperature for 12–20 hours (overnight). After the reaction was completed and the vials were removed from the shaker, 400 µL of 5.0 mM internal standard (1,2,3-trimethoxylbenzene) acetonitrile solution were added. The mixture was transferred to a 2.0-mL Eppendorf tube and then subjected to vortexing (20 s × 2) and centrifugation (14,000 × g, 5 min, 4 °C). A sample of the supernatant (0.2 mL) was transferred to a vial with an insert for LC-MS analysis. Products were identified and quantified based on the corresponding reference compounds (**Section V** and **VI**). To further determine the enantiomeric excess (*ee*), the remaining supernatants of three parallel analytical reactions were combined and transferred to a 2-dram vial and the solvent was removed by blowing air. After shaking the vials for 1 hour, the indolines were extracted to a solution of hexane and EtOAc mixture (1:1) and subjected to normal-phase HPLC to determine the *ee*.

(E) Reaction screening in 96-well plate in whole-cell format

After a single-site saturation mutagenesis (SSM) library was generated, 88 single colonies were randomly picked and cultured in 300 µL of LB medium with 0.1 mg/mL ampicillin (LB_{amp}) in a sterilized 96-well culture plate. The plate typically contained six wells inoculated with single colonies expressing the parent enzyme and two sterile wells. The cultures were grown at 37 °C, 250 rpm, and 80% relative humidity for 13–15 hours. A separate, sterilized 96-well culture plate was filled with 950 µL of Hyperbroth medium containing 0.1 mg/mL ampicillin (HB_{amp}) in each well. Likewise, a glycerol stock replica plate of the preculture (50 µL of preculture added to 50 µL of 50% glycerol per well) was also prepared and stored at -80 °C for future reference. The plate with HB_{amp} was inoculated with the LB preculture (50 µL/well), and incubated at 37 °C, 250 rpm, and 80% relative humidity for 2.5 hours. The plate was cooled on ice for 1 hour, induced with 0.5 mM IPTG and 1 mM ALA (final concentrations), and then expressed at 22 °C and 220 rpm for 21–23 hours. The cells were pelleted (4,000*g*, 6 minutes), and after removal of the supernatant, 390 µL of M9-N (pH 7.4, 20 mM D-glucose) were added to each well. After the cell pellets were fully resuspended by shaking at 500 rpm, the 96-well plate was transferred to an anaerobic chamber. Inside the anaerobic chamber, to each well were added 10 µL of a reactant stock solution in ethanol (100 or 200 mM stock solution, 2.5 or 5.0 mM final concentration). The plate was sealed with aluminum foil tape and shaken at 600 rpm in the anaerobic chamber at room temperature overnight.

Once the plate was taken out of the anaerobic chamber and the seal was removed, ethanol (400 µL/well) was added. The resulting suspension in the wells was mixed by vortexing and shaking. The plate was then centrifuged (5,000*g*, 5 minutes) to precipitate proteins and cell debris. The supernatant (200 µL/well) was transferred to a shallow 96-well plate for reverse-phase HPLC-MS analysis. The MS product signals from each well were compared, and wells showing signals higher than the parent wells were identified. These 'hits' were recultured using the wells from the frozen replica glycerol stock plate and then sequenced. Among these, the activities of candidates showing mutations at the targeted site were revalidated in analytical scale reactions. In this way,

the best variant was selected as the final hit and was used as the parent for the next round of site-saturation mutagenesis.

II. Directed Evolution of P411-PYS-5149 and P411-INS-5151 for Intramolecular C(sp^3)–H Amination

Discovery of Initial Activity

The Arnold lab collection of heme enzymes (including P450s, P411s, protoglobins, and cytochromes *c*) was screened in 96-well-plate whole-cell reactions for product formation (see Section I.E). The collection consisted of nearly 400 distinct variants accumulated from prior directed evolution campaigns. For pyrrolidine synthesis, one variant from a previous carbene transferring lineage showed the highest product formation as assayed by LCMS. For indoline synthesis, the pyrrolidine synthase lineage showed the most promising results, and the 7th generation showed highest activity as assayed by LCMS. No product was detected in control reactions with free heme.

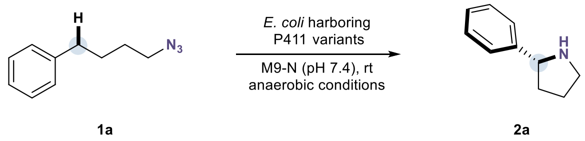

Pyrrolidine synthesis variants showing product formation:

Variant	Ion Count
P450-BM3 (WT)	0
P411-A330Y	2786747
P411-M177L	4420892

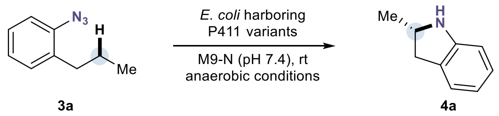

Indoline synthesis variants showing product formation:

Variant	Ion Count
P450-BM3 (WT)	0
P411-PYS-5146	2027686
P411-PYS-5148	8380970

Table S1. Detailed information of the evolutionary lineage for **PY**rrolidine **S**ynthase (**PYS**).

**P411-PYS** variant	**Yield (%)**	***er***
**P411-M177L** (**P411-PYS-5141**)	4 ± 0.8	84:16
**P411-PYS-5141**- L75E (**P411-PYS-5142**)	13 ± 0.2	91:9
**P411-PYS-5141**- L75E Q437L (**P411-PYS-5143**)	13 ± 1.0	90:10
**P411-PYS-5141**- L75E Q437L A330Q (**P411-PYS-5144**)	20 ± 0.9	91:9
**P411-PYS-5141**- L75E Q437L A330Q M118V(**P411-PYS-5145**)	29 ± 1.6	93:7

**P411-PYS-5141-** L75E Q437L A330Q M118V F77C (**P411-PYS-5146**)	31 ± 1.0	91:9
**P411-PYS-5141-** L75E Q437L A330Q M118V F77C S72W (**P411-PYS-5147**)	53 ± 0.6	90:10
**P411-PYS-5141-** L75E Q437L A330Q M118V F77C S72W Q73A (**P411-PYS-5148**)	54 ± 1.6	91:9
**P411-PYS-5141-** L75E Q437L A330Q M118V F77C S72W Q73A L436R (**P411-PYS-5149**)	66 ± 3.5	91:9

Table S2. Detailed information of the evolutionary lineage for **IN**doline **S**ynthase (**INS**).

**P411-INS** variant	**Yield (%)**	***er***
**P411-PYS-5148**	46 ± 3.2	51:49
**P411-PYS-5148**- L437P (**P411-INS-5150**)	49 ± 1.7	88:12
**P411-PYS-5148-** L437P L181N (**P411-INS-5151**)	52 ± 1.0	91:9

Table S3. Further information on directed evolution experiments.

**Round #**	**Parent**	**Sites targeted for SSM**	**Screen for activity or enantioselectivity**
1	**P411-PYS-5141**	75X	**activity**
2	**P411-PYS-5142**	78X, 87X, 263X, 268X, 327X, 328X, **437X**	**activity**
3	**P411-PYS-5143**	86X, 267X, **330X**, 400X	**activity**
4	**P411-PYS-5144**	82X, **118X**, 181X, 395X, 438X, 401X	**activity**
5	**P411-PYS-5145**	**77X**, 177X 266X, 326X, 409X, 436X	**activity**
6	**P411-PYS-5146**	**72X**, 88X, 162X, 264X, 333X, 435X	**activity**
7	**P411-PYS-5147**	70X, 71X, **73X**, 74X, 76X, 178X, 269X, 331X, 332X	**activity**
8	**P411-PYS-5148**	87X, 267X, 268X, 327X, **436X**, 437X	**activity**
9	**P411-PYS-5148**	87X, 267X, 268X, 327X,	**both**

	**(for indoline synthesis)**	436X, **437X**	
10	**P411-INS-5149**	72X, 78X, 82X, **181X**, 188X, 438X	**both**

III. Supporting Experimental Figures and Miscellaneous Experiments

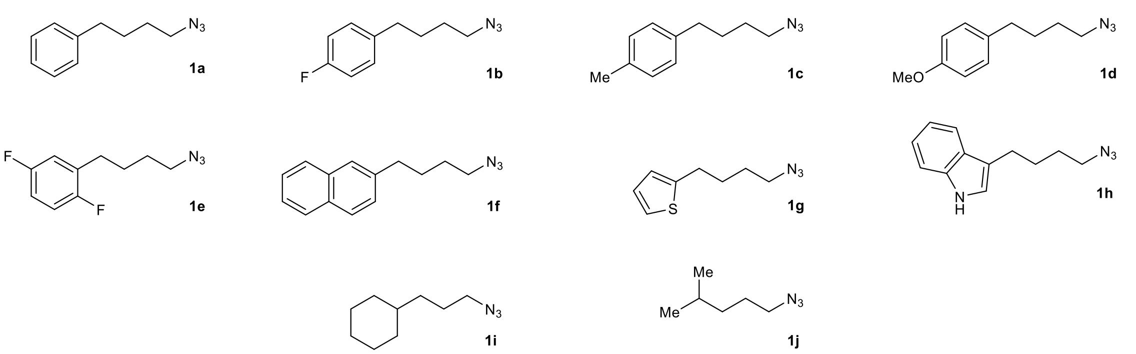

Figure S1. Summary of alkyl azide substrates (**1a**–**1j**).

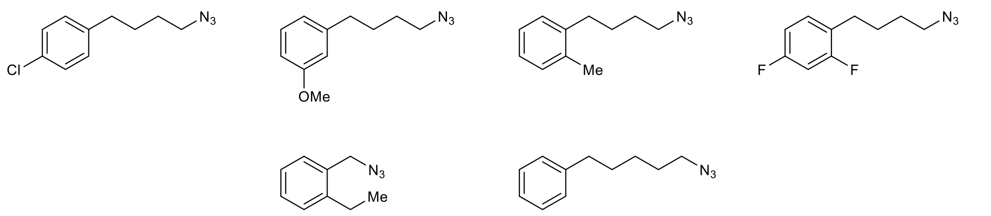

Figure S2. Other substrates tested for intramolecular alkyl nitrene C(sp^3)–H amination reaction.

Notes: The alkyl azide substrates listed here have been tested briefly for the intramolecular C(sp^3)–H amination chemistry catalyzed by **P411-PYS-5149**. However, these substrates only gave low activity, as preliminarily analyzed by LC-MS and chiral HPLC. These results are noteworthy, but they cannot be used for drawing final conclusions before further validation.

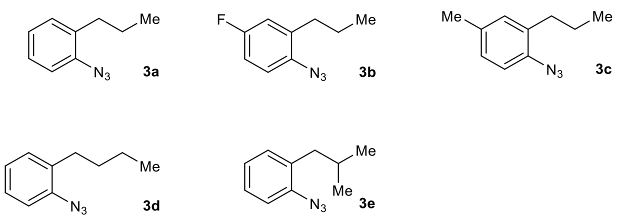

Figure S3. Summary of aryl azide substrates (**1a**–**1j**).

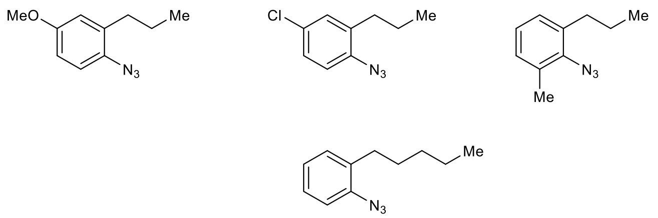

Figure S4. Other substrates tested for intramolecular aryl nitrene C(sp^3)–H amination reaction.

Notes: The aryl azide substrates listed here have been tested briefly for the intramolecular C(sp^3)–H amination chemistry catalyzed by **P411-INS-5151**. However, these substrates only gave low activity, as preliminarily analyzed by LC-MS and chiral HPLC. These results are noteworthy, but they cannot be used for drawing final conclusions before further validation.

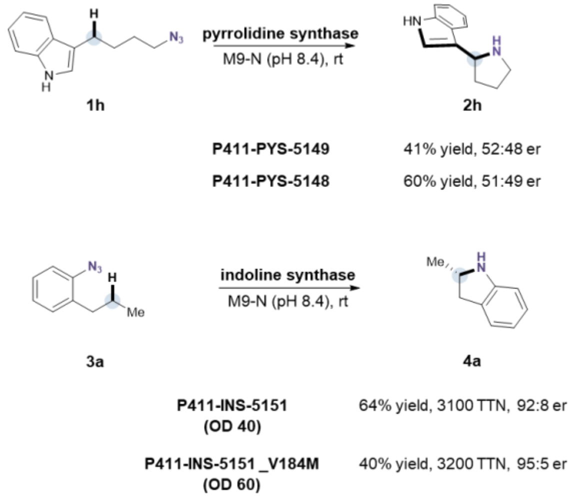

Figure S5. Variants that showed improved activity or selectivity for examples **1h** and **3a**.

Notes: Variant **P411-PYS-5148 (CA-G7)** is the seventh generation of 'pyrrolidine synthase' lineage. Variant **P411-INS-5151_V184M** has one mutation (V184M) relative to **P411-INS-5151 (AN-G2)**.

Kinetic Isotope Effect (KIE) studies

From a freshly streaked plate of *E. coli.* cells with the **P411-PYS-5149** plasmid, two single colonies were inoculated into two separate, 4-mL LB media tubes for ~14 h at 37 ºC. These were utilized for duplicate experiments. After growing the overnight culture for ~14 h, 1 mL of the starter culture was transferred to 49 mL HB media (supplemented with Ampicillin and Glucose Mix) in a 150-mL Erlenmeyer flask and grown with vigorous shaking at 37 ºC (230 rpm) for 2 h, by which time the cell optical density (OD_{600}) was 0.8 to 1.0. The flask was chilled on ice for 45 min. ALA (1 mM) and IPTG (0.5 mM) were supplemented, and the flask shaken at 22 ºC (140 rpm) for 22 h. The culture was centrifuged (4,000g, 6 min), and the cell pellets were resuspended in 12 mL of M9-N buffer (pH 8.4) to give a suspension with OD_{600}~40. All manipulations were done on ice. Reaction setup follows the standard procedure mentioned in **Section I (D)**.

• Calibration curve of non-deuterated product **2a** at low yields (using ion count in single-mass channel to quantify)

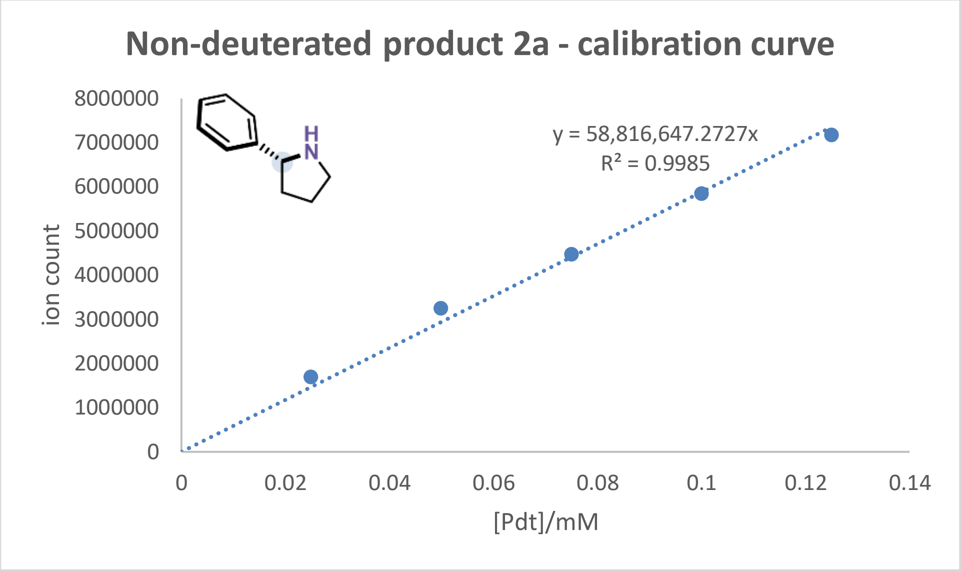

• Non-competitive KIE Experiments

Entry	Subs	Ion Count			Yield (%)			$K_{H/D}$	KIE
		30 s	60 s	90 s	30 s	60 s	90 s		(K_H/K_D)
1	H-1a	146156	247766	176056	0.099	0.169	0.220	0.00261	1.43
	D-1a	100606	176056	224437	0.068	0.120	0.153	0.00182	
2	H-1a	161435	267685	353785	0.110	0.182	0.241	0.00285	1.45
	D-1a	105536	190294	240509	0.072	0.129	0.164	0.00196	

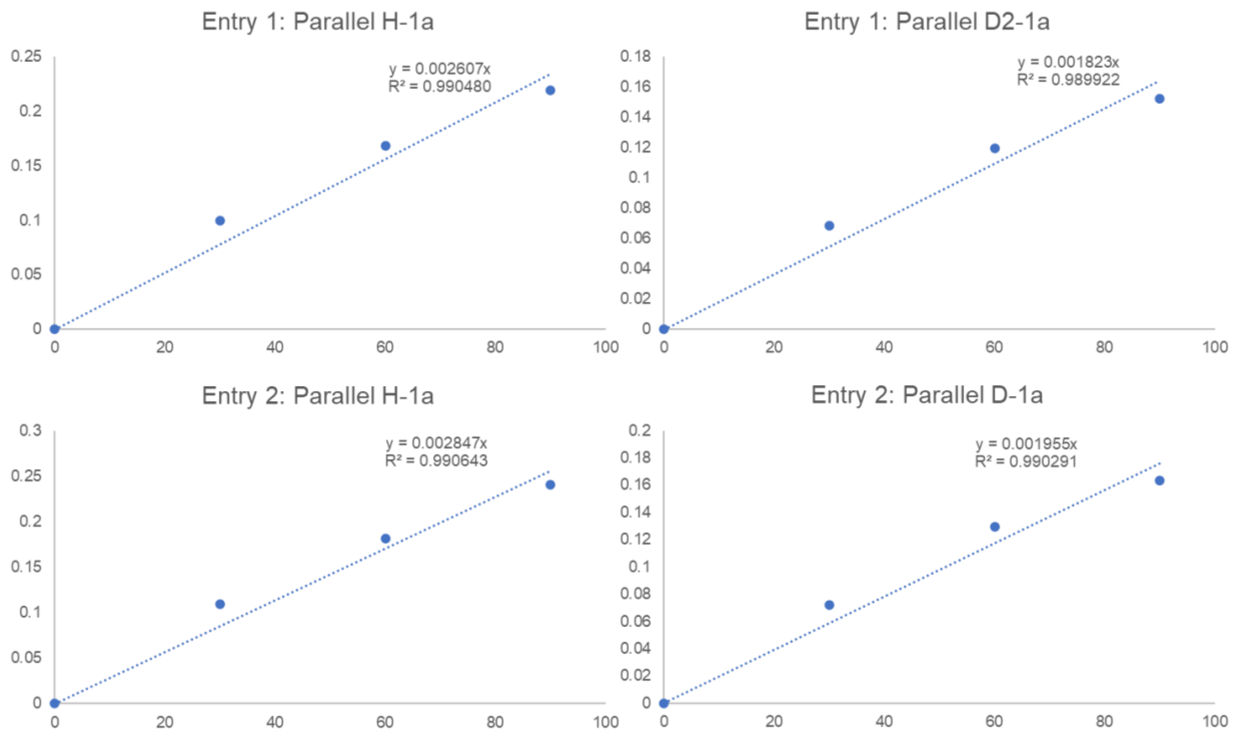

Figure S6. Duplicate of non-competitive kinetic isotope effect (KIE) experiments

• Competitive KIE Experiments

Entry	Subs	Ion Count			Yield (%)			$K_{H/D}$	KIE (K_H/K_D)
		30 s	60 s	90 s	30 s	60 s	90 s		
1	1:1 H/D-1a	167110	276341	367201	0.114	0.188	0.250	0.00295	3.14
		56674	87321	116399	0.039	0.059	0.079	0.00094	
2	1:1 H/D-1a	182657	290499	382915	0.124	0.198	0.260	0.00310	3.26
		42133	91205	120526	0.028	0.062	0.082	0.00095	

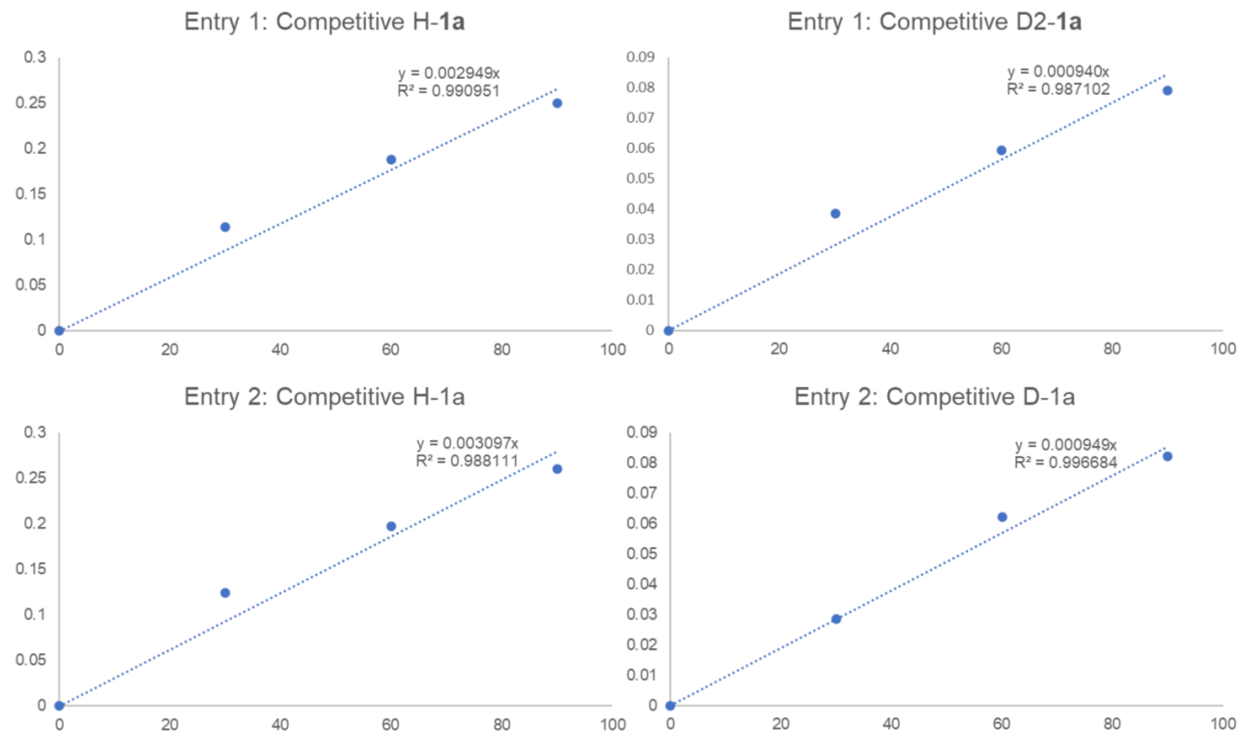

Figure S7. Duplicate of competitive kinetic isotope effect (KIE) experiments

• KIE Experiments Summary

Entry		KIE	Avg. KIE	SD KIE	KIE
Non-competitive KIE	1	1.43	1.44	0.01	1.44 ± 0.01
	2	1.45			
Competitive KIE	1	3.14	3.20	0.08	3.20 ± 0.08
	2	3.26			

IV. Preparation and Characterization of the Azide Substrates

General procedure A:[4]

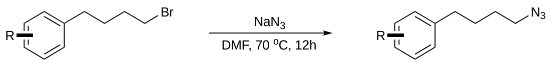

To a 100-mL flask were added alkyl bromide (5.0 mmol, 1.0 equiv.), sodium azide (487.6 mg, 7.5 mmol, 1.5 equiv.), and anhydrous DMF (50 mL). The mixture was stirred at 70 °C for 12 h, then cooled to room temperature, and diluted with diethyl ether/water (50 mL, 1:1). The product was extracted by diethyl ether (30 mL × 3), and the combined organic layer was washed with brine (50 mL). The organic phase was further dried over Na_2SO_4 and concentrated to afford a yellow residue. Flash column chromatography was performed to afford pure product in 60% to 80% yield.

General procedure B:

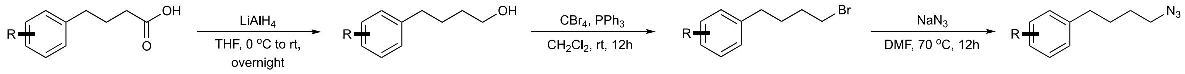

LAH reduction:[5] To a round-bottom flask containing carboxylic acids (1.0 equiv.) and anhydrous THF (0.1 M) at 0 °C was slowly added $LiAlH_4$ (5.0 equiv.). The mixture was gradually warmed to room temperature and stirred for 18 h. Fieser method was conducted to workup the LAH reduction. After filtration, the organic phase was dried over Na_2SO_4, and further concentrated to afford a yellow residue. Flash column chromatography was performed to afford pure product in quantitative yield.

Bromination:[6] To a round-bottom flask containing alcohols (1.0 equiv.) and anhydrous dichloromethane (DCM, 0.3 M) was added CBr_4 (1.2 equiv.) and PPh_3 (1.2 equiv.) sequentially. The mixture was stirred at room temperature for 4 h before quenching with saturated aqueous $NaHCO_3$ and extracted with DCM (× 3). The combined organic layer was washed with brine, further dried with Na_2SO_4, and concentrated to afford a colored residue. Flash column chromatography was performed to afford pure product in 70% to 80% yield.

Azidation: To a round-bottom flask were added alkyl bromide (1.0 equiv.), sodium azide (1.5 equiv.), and anhydrous DMF (0.1 M). The mixture was stirred at 70 °C for 12 h, then cooled to room temperature, and diluted with diethyl ether/water (1:1). The product was extracted by diethyl ether (× 3), and the combined organic layer was washed with brine (50 mL). The organic phase was further dried over Na_2SO_4 and concentrated to afford a yellow residue. Flash column chromatography was performed to afford pure product in 60% to 80% yield.

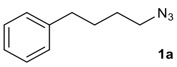

1a **(4-azidobutyl)benzene (1a):** The synthesis was conducted through general procedure A. **^{1}H NMR** (400 MHz, $CDCl_3$) δ 7.33 – 7.24 (m, 2H), 7.23 – 7.13 (m, 3H), 3.29 (t, *J* = 6.6 Hz, 2H), 2.65 (t, *J* = 7.4 Hz, 2H), 1.78 – 1.58 (m, 4H). **^{13}C NMR** (101 MHz, $CDCl_3$) δ 142.0, 128.5, 128.5, 126.0, 51.5, 35.5, 28.6.

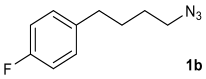

1b **1-(4-azidobutyl)-4-fluorobenzene (1b):** The synthesis was conducted through general procedure B. **^{1}H NMR** (400 MHz, $CDCl_3$) δ 7.15 – 7.03 (m, 4H), 3.28 (t, *J* = 6.7 Hz, 2H), 2.61 (t, *J* = 7.3 Hz, 2H), 2.32 (s, 3H), 1.78 – 1.57 (m, 4H). **^{13}C NMR** (101 MHz, $CDCl_3$) δ 161.4 (d, *J* = 243.5 Hz), 137.5 (d, *J* = 3.2 Hz), 129.8 (d, *J* = 7.7 Hz), 115.3 (d, *J* = 21.2 Hz), 51.4, 34.7, 28.7, 28.5. **^{19}F NMR** (376 MHz, $CDCl_3$) δ -117.6.

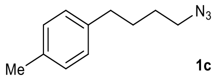

1c **1-(4-azidobutyl)-4-methylbenzene (1c):** The synthesis was conducted through general procedure B. **^{1}H NMR** (400 MHz, $CDCl_3$) δ 7.14 – 7.02 (m, 4H), 3.28 (t, J = 6.5 Hz, 2H), 2.61 (t, J = 7.2 Hz, 2H), 2.32 (s, 3H), 1.76 – 1.57 (m, 4H). **^{13}C NMR** (101 MHz, CDCl3) δ 138.9, 135.5, 129.2, 128.4, 51.5, 35.0, 28.7, 28.6, 21.1.

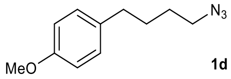

1d **1-(4-azidobutyl)-4-methoxybenzene (1d):** The synthesis was conducted through general procedure A. **^{1}H NMR** (400 MHz, $CDCl_3$) δ 7.13 – 7.06 (m, 2H), 6.87 – 6.79 (m, 2H), 3.79 (s, 3H), 3.28 (t, J = 6.5 Hz, 2H), 2.59 (t, J = 7.2 Hz, 2H), 1.74 – 1.56 (m, 4H). **^{13}C NMR** (101 MHz, $CDCl_3$) δ 158.0, 134.0, 129.4, 113.9, 55.4, 51.5, 34.6, 28.8, 28.5.

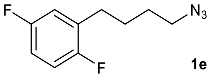

1e **2-(4-azidobutyl)-1,4-difluorobenzene (1e):** The synthesis was conducted through general procedure B. **^{1}H NMR** (400 MHz, $CDCl_3$) δ 6.96 (td, *J* = 8.8, 4.4 Hz, 1H), 6.92 – 6.80 (m, 2H), 3.30 (t, *J* = 6.5 Hz, 2H), 2.65 (t, *J* = 6.7 Hz, 2H), 1.74 – 1.60 (m, 4H). **^{13}C NMR** (101 MHz, $CDCl_3$) δ 158.7 (d, *J* = 239.4 Hz), 157.2 (d, *J* = 240.6 Hz), 130.5 (dd, *J* = 18.7, 7.7 Hz), 116.9 (dd, *J* = 23.6, 5.3 Hz), 116.3 (dd, *J* = 25.4, 8.8 Hz), 114.0 (dd, *J* = 24.0, 8.6 Hz), 51.3, 28.6, 28.5, 27.1. **^{19}F NMR** (376 MHz, $CDCl_3$) δ -119.6 (d, *J* = 18.0 Hz), -125.0 (d, *J* = 18.0 Hz).

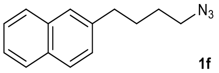

1f **2-(4-azidobutyl)naphthalene (1f):** The synthesis was conducted through general procedure B. **^{1}H NMR** (400 MHz, $CDCl_3$) δ 7.79 (ddd, *J* = 10.9, 7.7, 1.1 Hz, 3H), 7.61 (dd, *J* = 1.8, 0.9 Hz, 1H), 7.55 – 7.38 (m, 2H), 7.33 (dd, *J* = 8.5, 1.7 Hz, 1H), 3.31 (t, *J* = 6.8 Hz, 2H), 2.82 (t, *J* = 7.7 Hz, 2H), 1.87 – 1.74 (m, 2H), 1.74 – 1.59 (m, 2H). **^{13}C NMR** (101 MHz, $CDCl_3$) δ 139.4, 133.7, 132.2, 128.1, 127.8, 127.6, 127.3, 126.6, 126.1, 125.3, 51.5, 35.6, 28.6, 28.4.

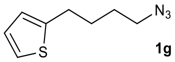

1g **2-(4-azidobutyl)thiophene (1g):** The synthesis was conducted through general procedure B. **^{1}H NMR** (400 MHz, $CDCl_3$) δ 7.13 (dd, *J* = 5.1, 1.2 Hz, 1H), 6.92 (dd, *J* = 5.1, 3.4 Hz, 1H), 6.79 (dq, *J* = 3.2, 1.0 Hz, 1H), 3.30 (t, *J* = 6.7 Hz, 2H), 2.87 (td, *J* = 7.3, 1.0 Hz, 2H), 1.83 – 1.72 (m, 2H), 1.72 – 1.61 (m, 2H). **^{13}C NMR** (101 MHz, $CDCl_3$) δ 144.7, 126.9, 124.4, 123.3, 51.4, 29.5, 28.9, 28.4.

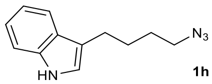

1h **3-(4-azidobutyl)-1H-indole (1h):** The synthesis was conducted through general procedure B. **^{1}H NMR** (400 MHz, $CDCl_3$) δ 7.92 (s, 1H), 7.61 (dq, *J* = 7.8, 0.9 Hz, 1H), 7.37 (dt, *J* = 8.1, 0.9 Hz, 1H), 7.21 (ddd, *J* = 8.1, 7.0, 1.2 Hz, 1H), 7.13 (ddd, *J* = 8.0, 7.0, 1.1 Hz, 1H), 6.99 (dt, *J* = 2.0, 0.9 Hz, 1H), 3.31 (t, *J* = 6.8 Hz, 2H), 2.81 (td, *J* = 7.4, 0.9 Hz, 2H), 1.86 – 1.76 (m, 2H), 1.76 – 1.66 (m, 2H). **^{13}C NMR** (101 MHz, $CDCl_3$) δ 136.5, 127.6, 122.1, 121.3, 119.3, 119.0, 116.3, 111.2, 51.6, 28.8, 27.3, 24.8.

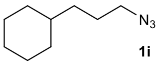

1i **(3-azidopropyl)cyclohexane (1i):** The synthesis was conducted through general procedure A. **^{1}H NMR** (400 MHz, $CDCl_3$) δ 3.24 (t, *J* = 7.0 Hz, 2H), 1.75 – 1.51 (m, 7H), 1.27 – 1.08 (m, 6H), 0.89 (tt, *J* = 11.6, 6.4 Hz, 2H). **^{13}C NMR** (101 MHz, $CDCl_3$) δ 52.0, 37.5, 34.6, 33.4, 26.7, 26.5, 26.4.

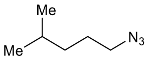

1j **1-azido-4-methylpentane (1j):** The synthesis was conducted through general procedure A. **^{1}H NMR** (400 MHz, $CDCl_3$) δ 3.25 (t, *J* = 7.0 Hz, 2H), 1.68 – 1.51 (m, 3H), 1.30 – 1.20 (m, 2H), 0.90 (d, *J* = 6.6 Hz, 6H). **^{13}C NMR** (101 MHz, $CDCl_3$) δ 51.9, 36.0, 27.9, 26.9, 22.6.

General procedure C:[7]

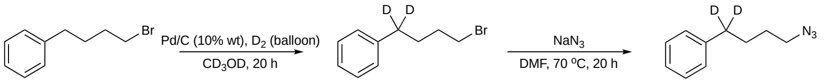

A mixture of (4-bromobutyl)benzene (0.53 g, 2.5 mmol, 1.0 equiv.) and Pd/C (10 wt%, 0.33 g, 60 mol%) in CD_3OD (5 mL) was placed under nitrogen and equipped with a deuterium balloon. After 20 h of reaction the mixture was filtered over celite, and the product diluted with diethyl ether (20 mL). The solution was washed with water (20 mL) and a saturated aqueous solution of sodium bicarbonate (× 2). The organic fraction was dried over $MgSO_4$ filtered and solvent removed to yield 4-bromo-1,1-bisdeutero-1-phenylbutane as a colorless oil which was used in the next step without additional purification.

The obtained 4-bromo-1,1-bisdeutero-1-phenylbutane was dissolved in DMF (10 mL) and sodium azide was added to the stirred solution. After heating at 70 °C for 20 hours the mixture was cooled to room temperature and water (20 mL) was added. The product was extracted with diethyl ether (20 mL), and the organic fraction was washed with water (5 × 30 mL), dried over $MgSO_4$, filtered and concentrated to afford a yellow residue. Flash column chromatography was performed to afford pure product as a colorless oil (0.25 g, 1.4 mmol, 90% deuteration rate, 56% overall yield).

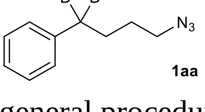

1aa **(4-azidobutyl-1,1-d2)benzene** (**1aa**): The synthesis was conducted through general procedure C (90% deuteration rate). ^{1}H NMR (400 MHz, $CDCl_3$) δ 7.29 (tt, *J* = 6.9, 0.9 Hz, 2H), 7.23 – 7.16 (m, 3H), 3.29 (t, *J* = 6.6 Hz, 2H), 1.75 – 1.59 (m, 3H). ^{13}C NMR (101 MHz, $CDCl_3$) δ 141.8, 128.4, 128.4, 125.9, 51.4, 35.2 – 34.4, 28.4, 28.3.

General procedure D:[8]

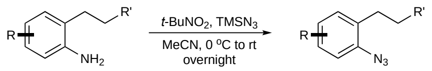

To a 100-mL flask at 0 ºC were added aniline (1.0 equiv.), MeCN (0.4 M), *t*-BuNO2 (4.0 equiv.), and TMSN3 (3.0 equiv.) dropwise, then the flask was warmed to room temperature and stirred overnight. De-ionized water was added after completion, the reaction mixture was extracted by diethyl ether (× 3), and the combined organic layer was washed with brine. The organic phase was further dried over Na_2SO_4 and concentrated to afford a yellow residue. Flash column chromatography was performed to afford pure product in 50% to 70% yield.

General procedure E:[9]

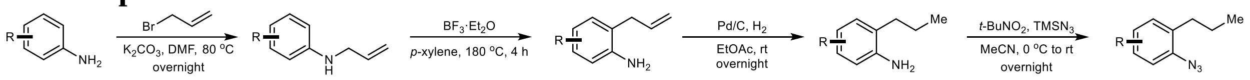

Allylation: To a 100-mL round-bottom flask were added aniline (1.0 equiv.), K_2CO_3 (2.4 equiv.), DMF (0.4 M) and allyl bromide (1.0 equiv.) dropwise. The solution was sealed and heated to 80 ºC overnight. The reaction mixture was filtered, washed with H_2O, and extracted with ethyl acetate twice. The combined organic layer was washed with brine, then dried over Na_2SO_4 and concentrated to afford a yellow residue. The crude product was purified by flash column chromatography (eluent: 10% Hex in EtOAc) to afford corresponding allyl aniline as a yellow oil with 40% to 50% yield.

Aza-Claisen rearrangement: To a 50-mL Schlenk tube were added allyl aniline (1.0 equiv.), *p*-xylene (2.0 M) and then added $BF_3{\cdot}Et_2O$ (1.0 equiv.) dropwise. The Schlenk tube was sealed and heated to 180 ºC for 4 h (sometimes longer based on how fast the rearrangement is).

Hydrogenation: To a 25-mL round-bottom flask were added corresponding aniline (1.0 equiv.) ethyl acetate (0.2 M) and Pd/C (100 mg/g of aniline) and then the mixture was degassed and recharged with a balloon of hydrogen. The reaction was vigorously stirred at room temperature overnight. After the reaction was completed, the mixture was filtered through a pad of celite before being concentrated to afford a yellow residue. The residue was directly used for the next step without any further purification.

Azidation: Same as General Procedure C described above.

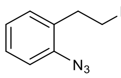

3a **1-azido-2-propylbenzene (3a):** The synthesis was conducted through general procedure C. **^{1}H NMR** (400 MHz, $CDCl_3$) δ 7.28 – 7.19 (m, 1H), 7.14 (ddd, *J* = 12.2, 7.7, 1.4 Hz, 2H), 7.06 (td, *J* = 7.4, 1.3 Hz, 1H), 2.58 – 2.50 (m, 2H), 1.66 – 1.52 (m, 2H), 0.94 (t, *J* = 7.3 Hz, 3H). **^{13}C NMR** (101 MHz, $CDCl_3$) δ 138.1, 134.3, 130.6, 127.3, 124.7, 118.2, 33.4, 23.6, 14.1.

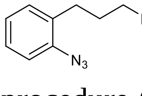

3b **1-azido-2-butylbenzene (3b):** The synthesis was conducted through general procedure C. **^{1}H NMR** (400 MHz, $CDCl_3$) δ 7.23 (td, *J* = 7.6, 1.7 Hz, 1H), 7.14 (ddd, *J* = 13.8, 7.7, 1.4 Hz, 2H), 7.06 (td, *J* = 7.4, 1.2 Hz, 1H), 2.56 (t, *J* = 7.6 Hz, 2H), 1.60 – 1.48 (m, 2H), 1.35

(dq, J = 14.6, 7.3 Hz, 2H), 0.93 (t, J = 7.3 Hz, 3H). **^{13}C NMR** (101 MHz, $CDCl_3$) δ 138.0, 134.5, 130.5, 127.2, 124.8, 118.2, 32.6, 31.1, 22.7, 14.1.

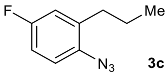

3c **1-azido-4-fluoro-2-propylbenzene (3c):** The synthesis was conducted through general procedure D. **^{1}H NMR** (400 MHz, $CDCl_3$) δ 7.06 (dd, J = 8.7, 4.8 Hz, 1H), 6.98 – 6.84 (m, 2H), 2.55 – 2.49 (m, 2H), 1.64 – 1.53 (m, 2H), 0.94 (t, J = 7.4 Hz, 3H). **^{13}C NMR** (101 MHz, $CDCl_3$) δ 159.9 (d, J = 243.7 Hz), 136.4 (d, J = 7.4 Hz), 133.8 (d, J = 2.8 Hz), 119.3 (d, J = 8.7 Hz), 117.2 (d, J = 22.4 Hz), 113.9 (d, J = 23.2 Hz), 33.3 (d, J = 1.4 Hz), 23.3, 14.0. **^{19}F NMR** (376 MHz, $CDCl_3$) δ -118.5.

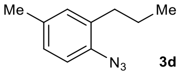

3d **1-azido-4-methyl-2-propylbenzene (3d):** The synthesis was conducted through general procedure D. **^{1}H NMR** (400 MHz, $CDCl_3$) δ 7.05 – 6.99 (m, 2H), 6.96 (s, 1H), 2.54 – 2.43 (m, 2H), 2.30 (s, 3H), 1.64 – 1.51 (m, 2H), 0.94 (t, J = 7.4 Hz, 3H). **^{13}C NMR** (101 MHz, $CDCl_3$) δ 135.2, 134.4, 134.0, 131.3, 127.8, 118.1, 33.4, 23.7, 21.0, 14.1.

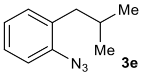

3e **1-azido-2-isobutylbenzene (3e):** The synthesis was conducted through general procedure C. **^{1}H NMR** (400 MHz, $CDCl_3$) δ 7.26 – 7.20 (m, 1H), 7.12 (ddd, J = 7.6, 3.6, 1.5 Hz, 2H), 7.05 (td, J = 7.4, 1.2 Hz, 1H), 2.44 (d, J = 7.2 Hz, 2H), 1.95 – 1.82 (m, 1H), 0.89 (d, J = 6.6 Hz, 6H). **^{13}C NMR** (101 MHz, $CDCl_3$) δ 138.3, 133.4, 131.5, 127.3, 124.5, 118.2, 40.5, 29.3, 22.5.

V. Preparation and Characterization of the Racemic Standards of Products

***Notes*:** All the pyrrolidine derivative standard products were commercially available and purchased from vendors mentioned in **Section I**. Unless otherwise noted, indoline derivative standard products were commercially available and purchased from vendors.

General procedure F:[10]

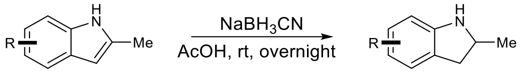

To a round-bottom flask at 0 ºC were added indole (1.0 equiv.), AcOH (0.2 M), and $NaBH_3CN$ (6.0 equiv.). The mixture was gradually warmed to room temperature and stirred overnight. After completion detected by TLC analysis, water and additional NaOH pellets were added until pH >12 before extracting with diethyl ether (three times). After extraction, the organic phase was combined, further dried over Na_2SO_4, and concentrated to afford a yellow residue. Flash column chromatography was performed to afford pure product in 50% to 60% yield.

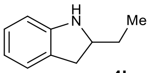

4b **2-ethylindoline (4b):** **^{1}H NMR** (400 MHz, $CDCl_3$) δ 7.11 – 7.04 (m, 1H), 7.01 (dddd, *J* = 7.7, 6.8, 1.3, 0.7 Hz, 1H), 6.70 (td, *J* = 7.4, 1.0 Hz, 1H), 6.63 (d, *J* = 7.7 Hz, 1H), 3.79 (tt, *J* = 8.5, 6.6 Hz, 1H), 3.13 (dd, *J* = 15.5, 8.6 Hz, 1H), 2.69 (dd, *J* = 15.5, 8.4 Hz, 1H), 1.65 (dtd, *J* = 13.8, 7.3, 3.0 Hz, 2H), 0.98 (t, *J* = 7.4 Hz, 3H). **^{13}C NMR** (101 MHz, $CDCl_3$) δ 150.7, 129.2, 127.4, 124.8, 118.9, 109.5, 61.7, 35.9, 29.6, 10.9. **HRMS** (FD) Calcd for $C_{10}H_{13}N$ [M] 147.1048, found 147.1038.

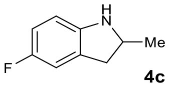

4c **5-fluoro-2-methylindoline (4c):** **^{1}H NMR** (400 MHz, $CDCl_3$) δ 6.80 (ddt, *J* = 8.5, 2.5, 1.2 Hz, 1H), 6.70 (dddt, *J* = 9.3, 8.4, 2.7, 0.8 Hz, 1H), 6.53 (ddd, *J* = 8.4, 4.4, 1.1 Hz, 1H), 4.08 – 3.94 (m, 1H), 3.13 (dd, *J* = 16.3, 8.9 Hz, 1H), 2.63 (dd, *J* = 15.4, 7.6 Hz, 1H), 1.30 (d, *J* = 6.3 Hz, 3H). **^{13}C NMR** (101 MHz, $CDCl_3$) δ 157.3 (d, *J* = 235.2 Hz), 146.6 (d, *J* = 1.5 Hz), 131.0 (d, *J* = 8.2 Hz), 113.3 (d, *J* = 23.1 Hz), 112.3 (d, *J* = 23.7 Hz), 109.8 (d, *J* = 8.4 Hz), 56.1, 38.1 (d, *J* = 1.8 Hz), 22.2. **^{19}F NMR** (376 MHz, $CDCl_3$) δ -126.3. **HRMS** (FD) Calcd for $C_9H_{10}NF$ [M] 151.0797, found 151.0798.

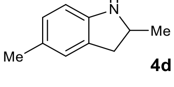

4d **2,5-dimethylindoline (4d):** **^{1}H NMR** (400 MHz, $CDCl_3$) δ 6.92 (s, 1H), 6.83 (d, *J* = 7.8 Hz, 1H), 6.54 (d, *J* = 7.8 Hz, 1H), 3.98 (ddq, *J* = 8.4, 7.8, 6.2 Hz, 1H), 3.11 (dd, *J* = 15.4, 8.4 Hz, 1H), 2.61 (dd, *J* = 15.4, 7.8 Hz, 1H), 2.25 (s, 3H), 1.29 (d, *J* = 6.3 Hz, 3H). **^{13}C NMR** (101

MHz, $CDCl_3$) δ 148.0, 129.7, 128.6, 127.7, 125.7, 109.8, 55.6, 37.9, 22.2, 21.0. **HRMS** (FD) Calcd for $C_{10}H_{13}N$ [M] 147.1048, found 147.1046.

General procedure G:[11]

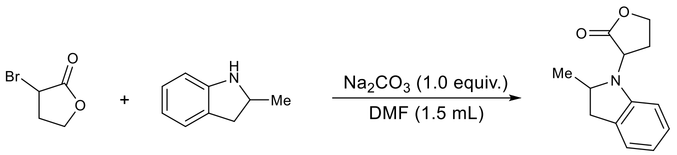

To a 40-mL dram vial were added a solution of alkylamine (2.2 mmol) in DMF (1.5 mL) and α-bromo-γ-butyrolactone (330 mg, 0.185 mL, 2 mmol). The resulting mixture was stirred at room temperature for 24 h. After reaction completion, the mixture was extracted with ethyl acetate (7 mL × 3), and the combined organic layer was washed with saturated $NaHCO_3$ solution (15 mL) and brine (15 mL). The organic phase was further dried over Na_2SO_4 and concentrated to afford a brown residue. The final product was purified through silica gel column chromatography.

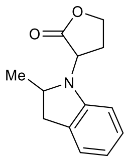

5 **3-(2-methylindolin-1-yl)dihydrofuran-2(3H)-one (5):** ^{1}**H NMR** (400 MHz, $CDCl_3$) δ 7.09 – 6.98 (m, 2H), 6.70 (dtd, *J* = 12.4, 7.4, 0.9 Hz, 1H), 6.30 (dd, *J* = 11.4, 7.9 Hz, 1H), 4.55 (tdd, *J* = 9.2, 6.7, 1.7 Hz, 1H), 4.39 – 4.22 (m, 2H), 4.07 – 3.70 (m, 1H), 3.28 – 3.13 (m, 1H), 2.77 – 2.45 (m, 2H), 2.43 – 2.29 (m, 1H), 1.34 (dd, *J* = 41.8, 6.2 Hz, 3H). ^{13}**C NMR** (101 MHz, $CDCl_3$) δ 175.2, 174.8, 148.7, 148.2, 129.7, 129.1, 127.4, 127.3, 125.0, 124.9, 119.1, 118.6, 108.8, 107.0, 65.7, 65.5, 60.0, 59.5, 55.0, 54.4, 37.3, 37.2, 24.7, 22.7, 20.3, 20.2. **HRMS** (FD) Calcd for $C_{13}H_{15}NO_2$ [M] 217.1103, found 217.1108.

VI. Analytic Scale Enzymatic Reactions and Calibration Curves for Products

All enzymatic reactions for intramolecular alkyl/aryl nitrene C–H insertion on analytical scale were conducted following the general procedure described in **Section I (D)**. Reactions for every substrate were set up in triplicate. Product formation was quantified by LC-MS based on the calibration curve of the corresponding racemic standard compound (**Section IV**). All TTNs for different products were calculated as the concentration of products divided by the concentration of hemoproteins measured by the CO binding assay (**Section I (C)**).

LC-MS calibration curve:

Calibration curves of synthesized reference compounds were created for the determination of yield and TTN. For each substrate, five different concentrations of product (1.00, 2.00, 3.00, 4.00, and 5.00 mM) in 400-μL *E. coli* cell solutions were quenched, each with 400 μL of 5.00 mM internal standard (ethyl benzoate or 1,2,3-trimethoxylbenzene) acetonitrile solution. The mixtures were vortexed and then analyzed by LC-MS based on UV absorbance at 254 or 210 nm. All data points represent the average of duplicate runs. The calibration curves depict the ratio of product area to internal standard area (y-axis) against product concentration in mM (x-axis).

Notes: Pdt = product area, IS = internal standard area, [Pdt] = product concentration in reaction, [PC] = protein concentration in reaction, Avg. TTN = average total turnover number, SD TTN = standard deviation of TTN, Avg. Yield = average yield, SD Yield = standard deviation of yield.

2-Phenylpyrrolidine (2a)

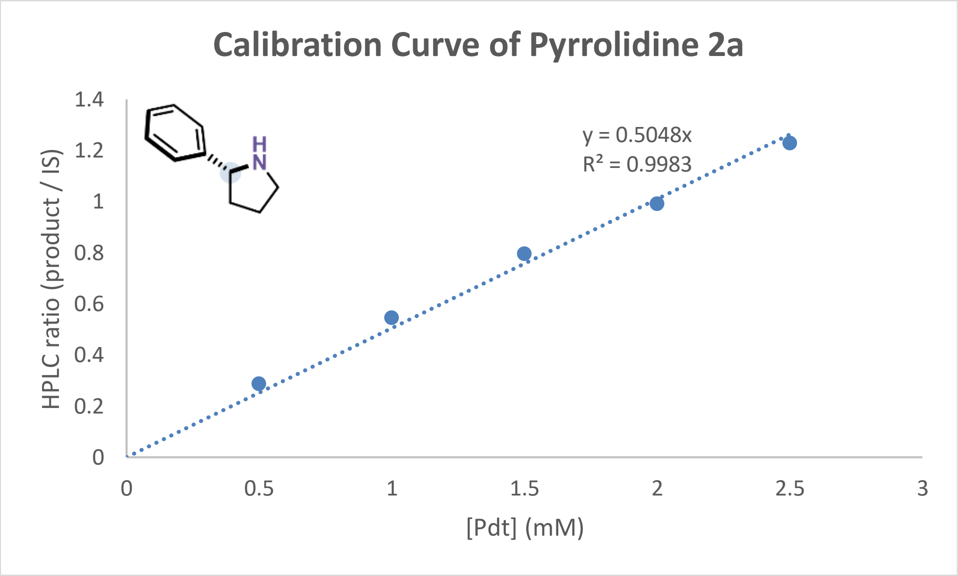

Data analysis for **P411-PYS-5141** catalyzed intramolecular C–H insertion with **1a** (2.5 mM, OD_{600} = 30):

Entry	Pdt	IS	Pdt/IS	[Pdt]/mM	[PC]/µM	Avg. TTN	SD TTN	Avg. Yield [%]	SD Yield [%]
1	50.6	967.3	0.0523	0.10	1.95	54	10	4	0.8
2	40.9	945.9	0.0432	0.086	1.95				
3	60.3	952.1	0.0633	0.13	1.95				

Data analysis for **P411-PYS-5142** catalyzed intramolecular C–H insertion with **1a** (2.5 mM, OD_{600} = 30):

Entry	Pdt	IS	Pdt/IS	[Pdt]/mM	[PC]/µM	Avg. TTN	SD TTN	Avg. Yield [%]	SD Yield [%]
1	159.7	964.7	0.166	0.33	1.58	210	3	13	0.2
2	161.9	952	0.170	0.34	1.58				
3	160.3	956.1	0.168	0.33	1.58				

Data analysis for **P411-PYS-5143** catalyzed intramolecular C–H insertion with **1a** (2.5 mM, OD_{600} = 30):

Entry	Pdt	IS	Pdt/IS	[Pdt]/mM	[PC]/µM	Avg. TTN	SD TTN	Avg. Yield [%]	SD Yield [%]
1	159.6	972.7	0.164	0.32	2.38	142	10	14	1.0
2	155.2	956	0.162	0.32	2.38				
3	179.5	970.8	0.185	0.37	2.38				

Data analysis for **P411-PYS-5144** catalyzed intramolecular C–H insertion with **1a** (2.5 mM, OD_{600} = 30):

Entry	Pdt	IS	Pdt/IS	[Pdt]/mM	[PC]/µM	Avg. TTN	SD TTN	Avg. Yield [%]	SD Yield [%]
1	241.6	932.6	0.259	0.51	2.21	224	10	20	0.9
2	240.9	951.2	0.253	0.50	2.21				
3	225.4	946.2	0.238	0.47	2.21				

Data analysis for **P411-PYS-5145** catalyzed intramolecular C–H insertion with **1a** (2.5 mM, OD_{600} = 30):

Entry	Pdt	IS	Pdt/IS	[Pdt]/mM	[PC]/μM	Avg. TTN	SD TTN	Avg. Yield [%]	SD Yield [%]
1	344.2	907.4	0.379	0.75	1.73	420	24	29	1.6
2	361.8	957.5	0.378	0.75	1.73				
3	329.4	960.3	0.343	0.68	1.73				

Data analysis for **P411-PYS-5146** catalyzed intramolecular C–H insertion with **1a** (2.5 mM, OD_{600} = 30):

Entry	Pdt	IS	Pdt/IS	[Pdt]/mM	[PC]/μM	Avg. TTN	SD TTN	Avg. Yield [%]	SD Yield [%]
1	369.4	916.5	0.403	0.80	1.68	463	15	31	1.0
2	368.8	931.8	0.396	0.78	1.68				
3	353	933.8	0.378	0.75	1.68				

Data analysis for **P411-PYS-5147** catalyzed intramolecular C–H insertion with **1a** (2.5 mM, OD_{600} = 30):

Entry	Pdt	IS	Pdt/IS	[Pdt]/mM	[PC]/μM	Avg. TTN	SD TTN	Avg. Yield [%]	SD Yield [%]
1	608.4	927.9	0.656	1.30	3.76	350	4	53	0.6
2	637.8	952.7	0.669	1.33	3.76				
3	628.5	938.6	0.670	1.33	3.76				

Data analysis for **P411-PYS-5148** catalyzed intramolecular C–H insertion with **1a** (2.5 mM, OD_{600} = 30):

Entry	Pdt	IS	Pdt/IS	[Pdt]/mM	[PC]/μM	Avg. TTN	SD TTN	Avg. Yield [%]	SD Yield [%]
1	635.7	958.9	0.663	1.31	3.76	361	11	54	1.6
2	645.5	938.4	0.688	1.36	3.76				
3	676.4	961.4	0.704	1.39	3.76				

Data analysis for **P411-PYS-5149** catalyzed intramolecular C–H insertion with **1a** (2.5 mM, OD_{600} = 30):

Entry	Pdt	IS	Pdt/IS	[Pdt]/mM	[PC]/μM	Avg. TTN	SD TTN	Avg. Yield [%]	SD Yield [%]
1	725.9	927.9	0.782	1.55	3.44	479	25	66	3.5
2	797.5	940.6	0.848	1.68	3.44				
3	812.8	938.3	0.866	1.72	3.44				

Data analysis for **P411-PYS-5149** (M9-N, pH = **8.4**) catalyzed intramolecular C–H insertion with **1a** (2.5 mM, OD_{600} = 30):

Entry	Pdt	IS	Pdt/IS	[Pdt]/mM	[PC]/μM	Avg. TTN	SD TTN	Avg. Yield [%]	SD Yield [%]
1	874.9	930.6	0.940	1.86	3.74	494	4	74	0.7
2	877.2	949.8	0.924	1.83	3.74				
3	877.7	940.7	0.933	1.85	3.74				

2-(4-Fluorophenyl)pyrrolidine (3b)

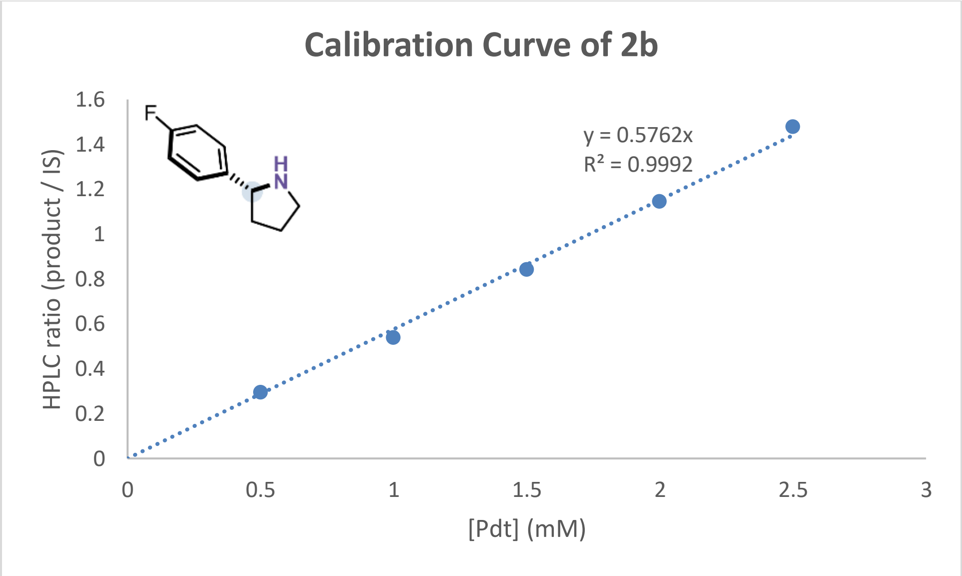

Data analysis for **P411-PYS-5149** (M9-N, pH = **8.4**) catalyzed intramolecular C–H insertion with **1b** (2.5 mM, OD_{600} = 30):

Entry	Pdt	IS	Pdt/IS	[Pdt]/mM	[PC]/μM	Avg. TTN	SD TTN	Avg. Yield [%]	SD Yield [%]
1	307	965.9	0.249	0.43	2.21	290	37	26	3.2

2	395.6	966.9	0.320	0.56	2.21				
3	371.1	974	0.301	0.52	2.21				

2-(*p*-Tolyl)pyrrolidine (2c)

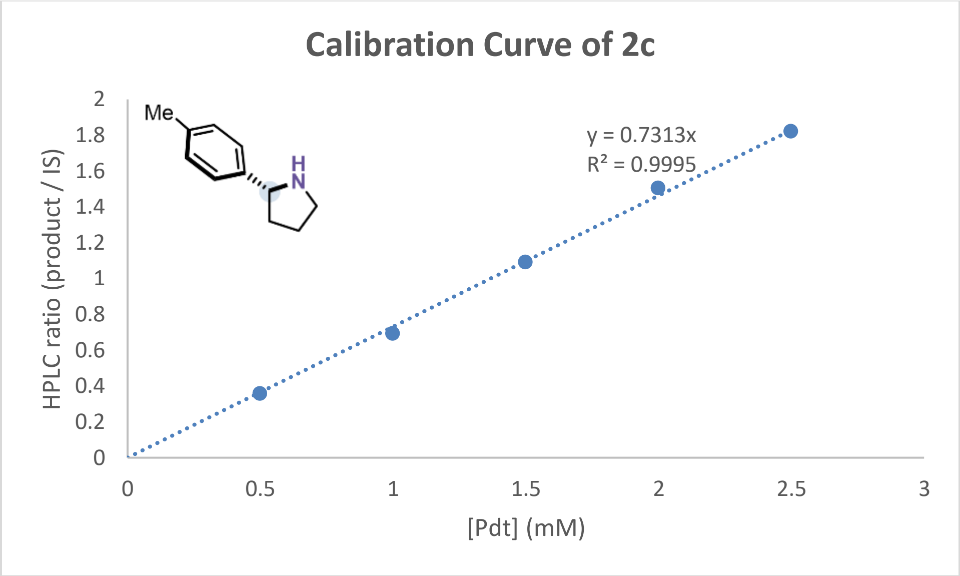

Data analysis for **P411-PYS-5149** (M9-N, pH = **8.4**) catalyzed intramolecular C–H insertion with **1c** (2.5 mM, OD_{600} = 30):

Entry	Pdt	IS	Pdt/IS	[Pdt]/mM	[PC]/μM	Avg. TTN	SD TTN	Avg. Yield [%]	SD Yield [%]
1	560.1	902.7	0.620	0.85	2.21	414	43	37	3.8
2	681.4	911	0.748	1.02	2.21				
3	576.7	902	0.639	0.87	2.21				

2-(4-Methoxyphenyl)pyrrolidine (2d)

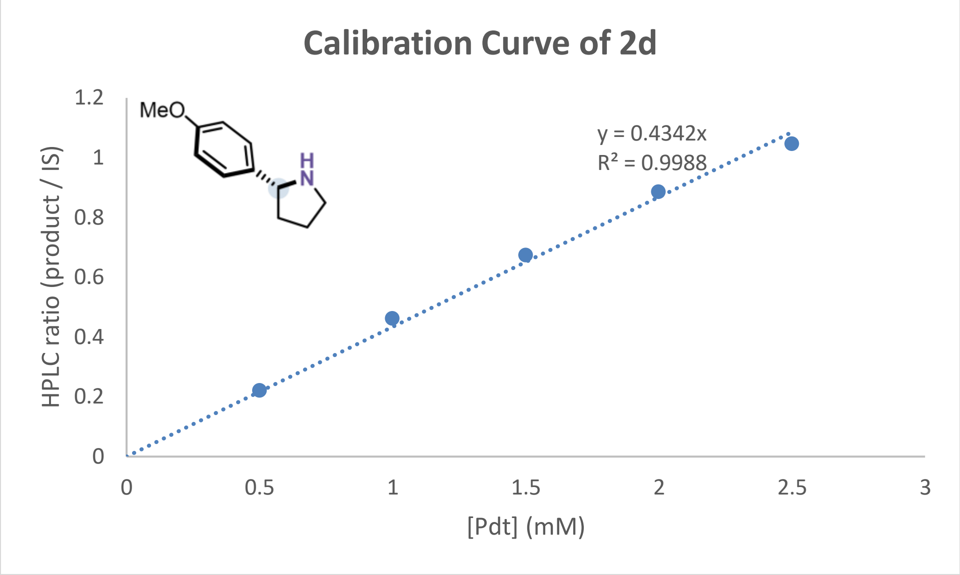

Data analysis for **P411-PYS-5149** (M9-N, pH = **8.4**) catalyzed intramolecular C–H insertion with **1d** (2.5 mM, OD_{600} = 30):

Entry	Pdt	IS	Pdt/IS	[Pdt]/mM	[PC]/μM	Avg. TTN	SD TTN	Avg. Yield [%]	SD Yield [%]
1	659.8	928.2	0.711	1.64	2.21	761	30	67	2.7
2	697.7	913.9	0.763	1.76	2.21				
3	656.5	916.3	0.716	1.65	2.21				

2-(2,5-Difluorophenyl)pyrrolidine (2e)

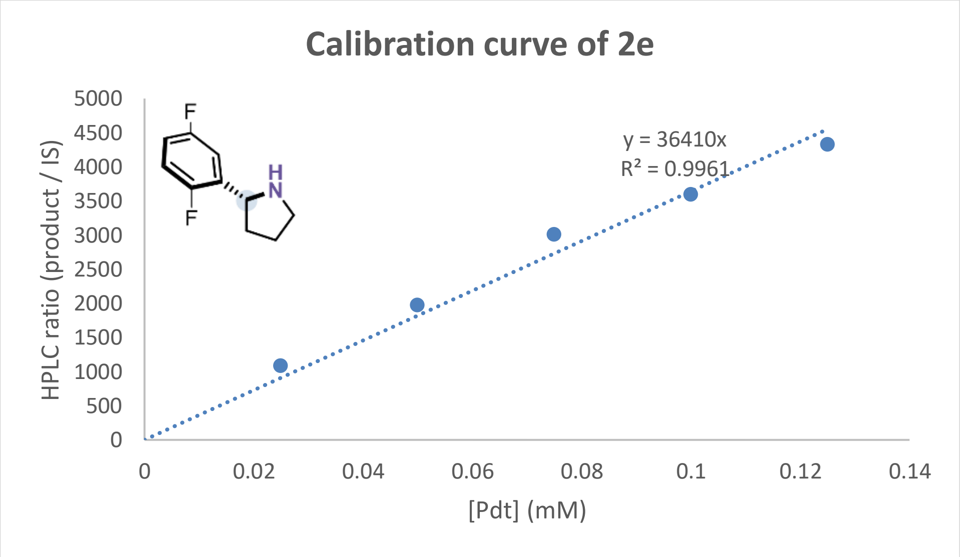

Data analysis for **P411-PYS-5149** (M9-N, pH = **8.4**) catalyzed intramolecular C–H insertion with **1e** (2.5 mM, OD_{600} = 30):

Entry	Pdt (Ion Count)	IS	Pdt/IS	[Pdt]/mM	[PC]/µM	Avg. TTN	SD TTN	Avg. Yield [%]	SD Yield [%]
1	2730675.0	919.7	2969.1	0.082	1.44	58	1	3.3	0.06
2	2790964.8	915.6	3048.2	0.084	1.44				
3	2751191.0	895.4	3072.6	0.084	1.44				

2-(Naphthalen-2-yl)pyrrolidine (2f)

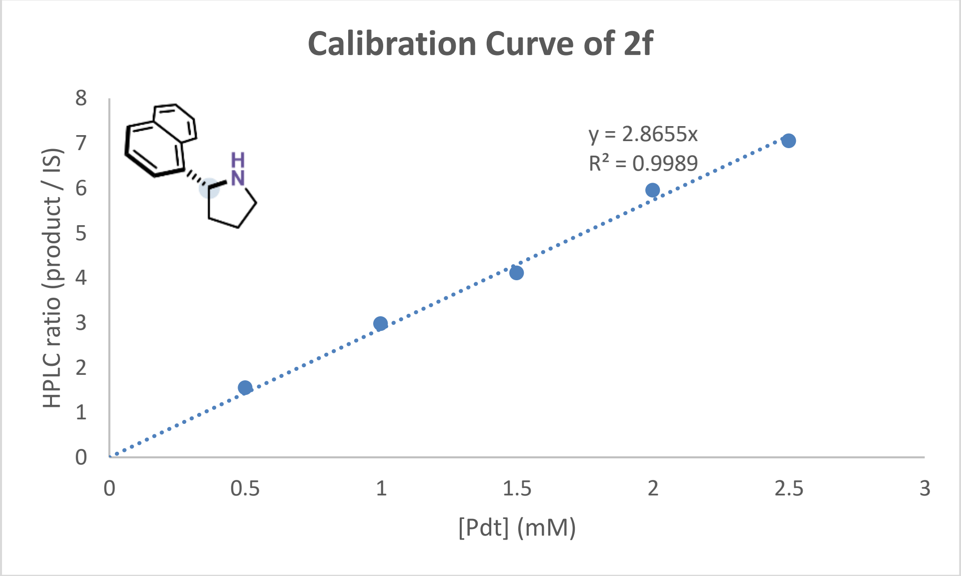

Data analysis for **P411-PYS-5149** (M9-N, pH = **8.4**) catalyzed intramolecular C–H insertion with **1f** (2.5 mM, OD_{600} = 30):

Entry	Pdt	IS	Pdt/IS	[Pdt]/mM	[PC]/µM	Avg. TTN	SD TTN	Avg. Yield [%]	SD Yield [%]
1	166.8	212.1	0.786	0.27	1.44	211	19	12	1.1
2	162.5	183.0	0.888	0.31	1.44				
3	155.6	165.8	0.938	0.33	1.44				

2-(Thiophen-2-yl)pyrrolidine (2g)

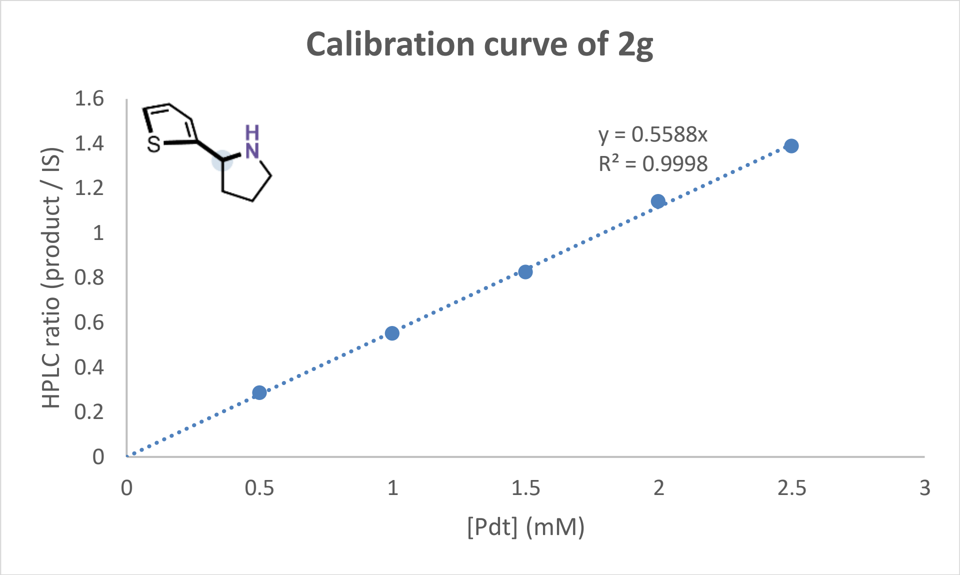

Data analysis for **P411-PYS-5149** (M9-N, pH = **8.4**) catalyzed intramolecular C–H insertion with **1g** (2.5 mM, OD_{600} = 30):

Entry	Pdt	IS	Pdt/IS	[Pdt]/mM	[PC]/µM	Avg. TTN	SD TTN	Avg. Yield [%]	SD Yield [%]
1	76.5	184.5	0.415	0.74	2.21	360	23	32	2.0
2	84.3	178.9	0.471	0.84	2.21				
3	80.4	179.9	0.447	0.80	2.21				

3-(Pyrrolidin-2-yl)-1H-indole (2h)

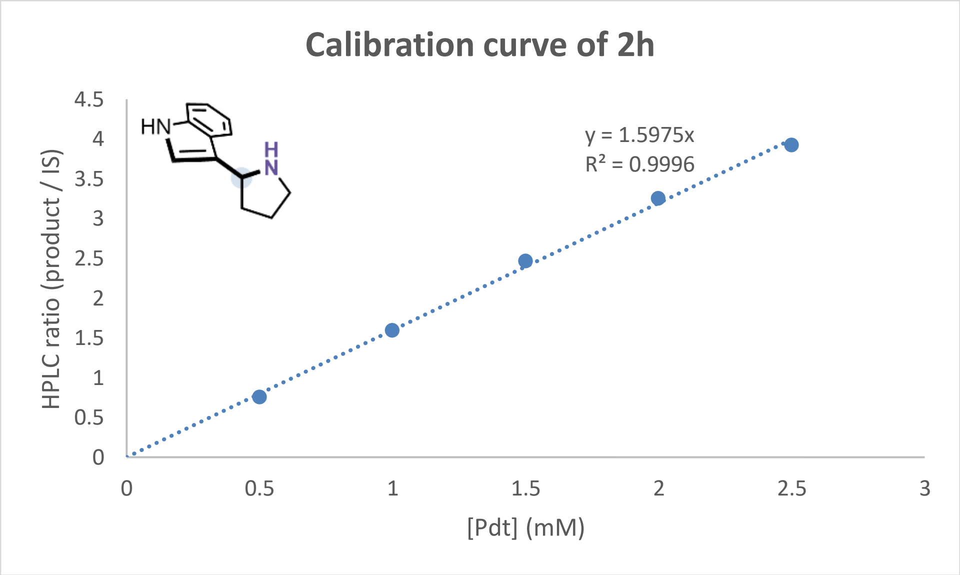

Data analysis for **P411-PYS-5149** (M9-N, pH = **8.4**) catalyzed intramolecular C–H insertion with **1h** (2.5 mM, OD_{600} = 30):

Entry	Pdt	IS	Pdt/IS	[Pdt]/mM	[PC]/μM	Avg. TTN	SD TTN	Avg. Yield [%]	SD Yield [%]
1	254.4	177.8	1.43	0.90	2.21	461	49	41	4.3
2	306.1	175.4	1.75	1.09	2.21				
3	290.4	170.1	1.71	1.07	2.21				

1-Azaspiro[4.5]decane (2i)

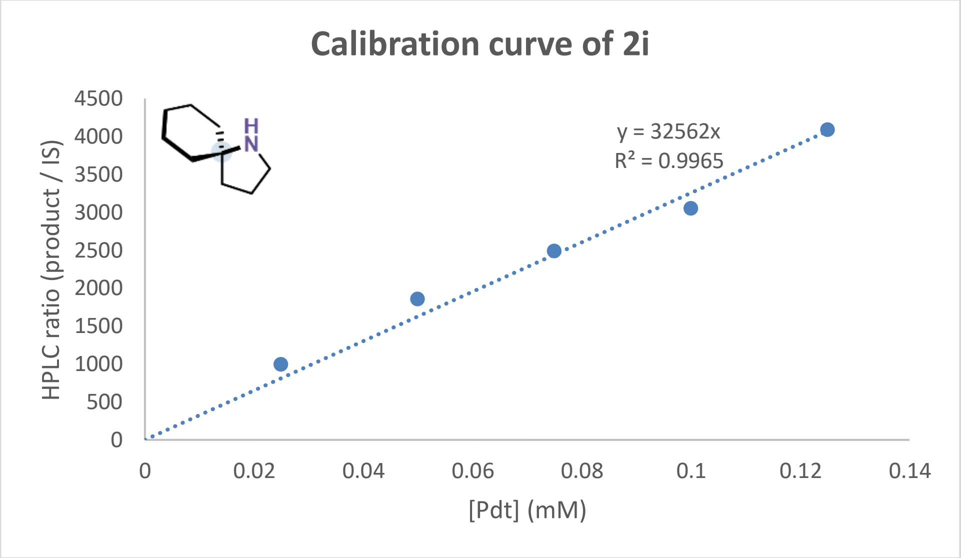

Data analysis for **P411-PYS-5149** (M9-N, pH = **8.4**) catalyzed intramolecular C–H insertion with **1i** (2.5 mM, OD_{600} = 30):

Entry	Pdt (Ion Count)	IS	Pdt/IS	[Pdt]/mM	[PC]/μM	Avg. TTN	SD TTN	Avg. Yield [%]	SD Yield [%]
1	2544228.3	931.9	2730.1516	0.084	1.44	57	2	3.3	0.1
2	2383615.3	931.8	2558.0761	0.079	1.44				
3	2489055.3	931.6	2671.8069	0.082	1.44				

2,2-Dimethylpyrrolidine (2j)

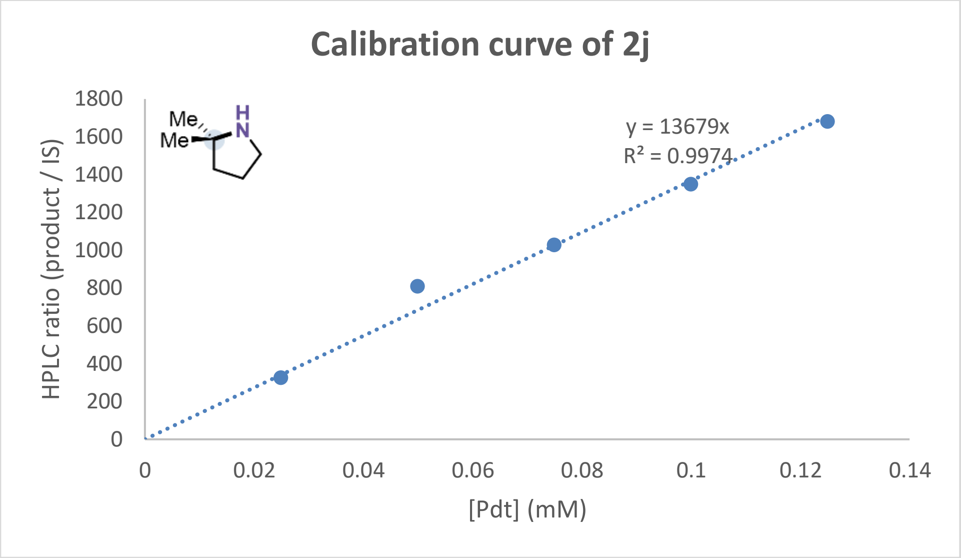

Data analysis for **P411-PYS-5149** (M9-N, pH = **8.4**) catalyzed intramolecular C–H insertion with **1j** (2.5 mM, OD_{600} = 30):

Entry	Pdt (Ion Count)	IS	Pdt/IS	[Pdt]/mM	[PC]/μM	Avg. TTN	SD TTN	Avg. Yield [%]	SD Yield [%]
1	657275.0	896.6	733.1	0.054	1.44	36	1	2.0	0.08
2	631301.0	915.8	689.3	0.050	1.44				
3	622256.9	916	679.3	0.050	1.44				

2-Methylindoline (4a)

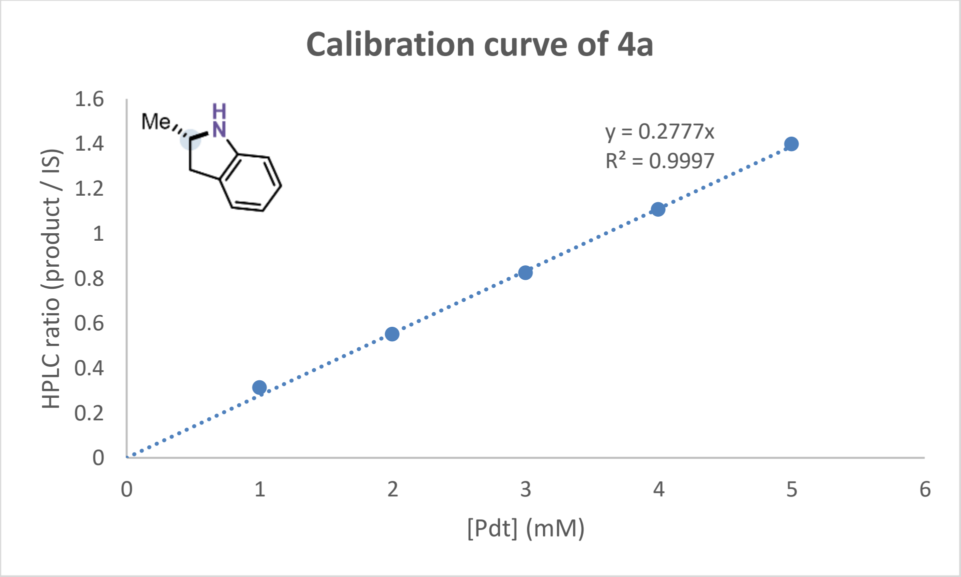

Data analysis for **P411-PYS-5148** (M9-N, pH = **7.4**) catalyzed intramolecular C–H insertion with **3a** (5.0 mM, OD_{600} = 40):

Entry	Pdt	IS	Pdt/IS	[Pdt]/mM	[PC]/µM	Avg. TTN	SD TTN	Avg. Yield [%]	SD Yield [%]
1	59.3	97.6	0.61	2.18	4.71	488	34	46	3.2
2	68.8	99.7	0.69	2.48	4.71				
3	58.2	94.2	0.62	2.22	4.71				

Data analysis for **P411-INS-5150** (M9-N, pH = **7.4**) catalyzed intramolecular C–H insertion with **3a** (5.0 mM, OD_{600} = 40):

Entry	Pdt	IS	Pdt/IS	[Pdt]/mM	[PC]/µM	Avg. TTN	SD TTN	Avg. Yield [%]	SD Yield [%]
1	66.3	95.2	0.70	2.51	3.29	742	25	49	1.7
2	63.7	97.7	0.65	2.35	3.29				
3	63.8	93.2	0.68	2.47	3.29				

Data analysis for **P411-INS-5151** (M9-N, pH = **7.4**) catalyzed intramolecular C–H insertion with **3a** (5.0 mM, OD_{600} = 40):

Entry	Pdt	IS	Pdt/IS	[Pdt]/mM	[PC]/µM	Avg. TTN	SD TTN	Avg. Yield [%]	SD Yield [%]
1	72.8	100.1	0.73	2.62	0.97	2690	49	52	1.0
2	67.8	92.3	0.73	2.65	0.97				
3	67.7	95.5	0.71	2.55	0.97				

Data analysis for **P411-INS-5151** (M9-N, pH = **8.4**) catalyzed intramolecular C–H insertion with **3a** (5.0 mM, OD_{600} = 40):

Entry	Pdt	IS	Pdt/IS	[Pdt]/mM	[PC]/µM	Avg. TTN	SD TTN	Avg. Yield [%]	SD Yield [%]
1	82.4	91.8	0.90	3.23	1.04	3100	48	64	1.0
2	88.5	97.5	0.91	3.27	1.04				
3	83.7	95.1	0.88	3.17	1.04				

Ethylindoline (4b)

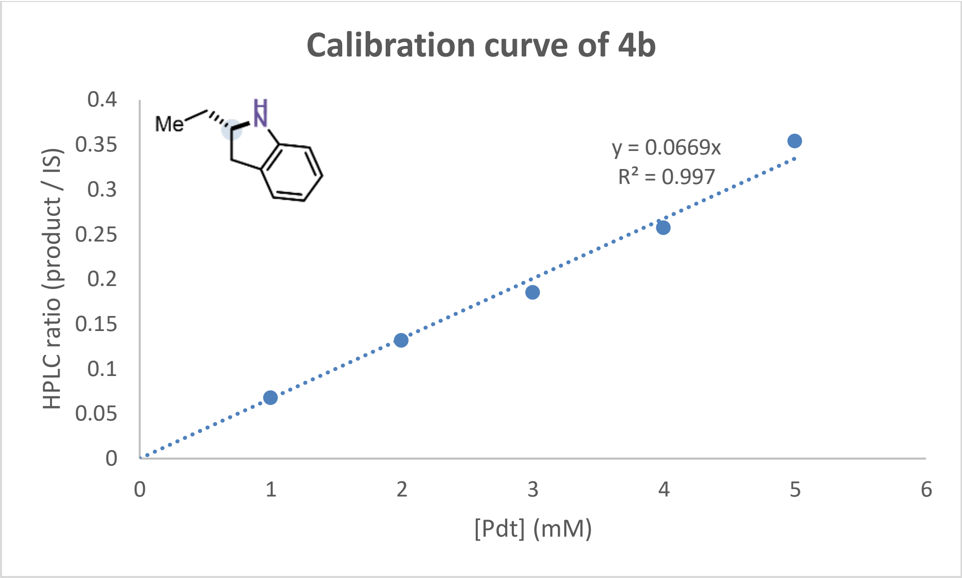

Data analysis for **P411-INS-5151** (M9-N, pH = **8.4**) catalyzed intramolecular C–H insertion with **3c** (5.0 mM, OD_{600} = 40):

Entry	Pdt	IS	Pdt/IS	[Pdt]/mM	[PC]/μM	Avg. TTN	SD TTN	Avg. Yield [%]	SD Yield [%]
1	258.0	5814.1	0.044	0.66	0.49	1310	57	13	0.6
2	261.0	5945.0	0.044	0.66	0.49				
3	232.6	5684.5	0.041	0.61	0.49				

5-Fluoro2-methylindoline (4c)

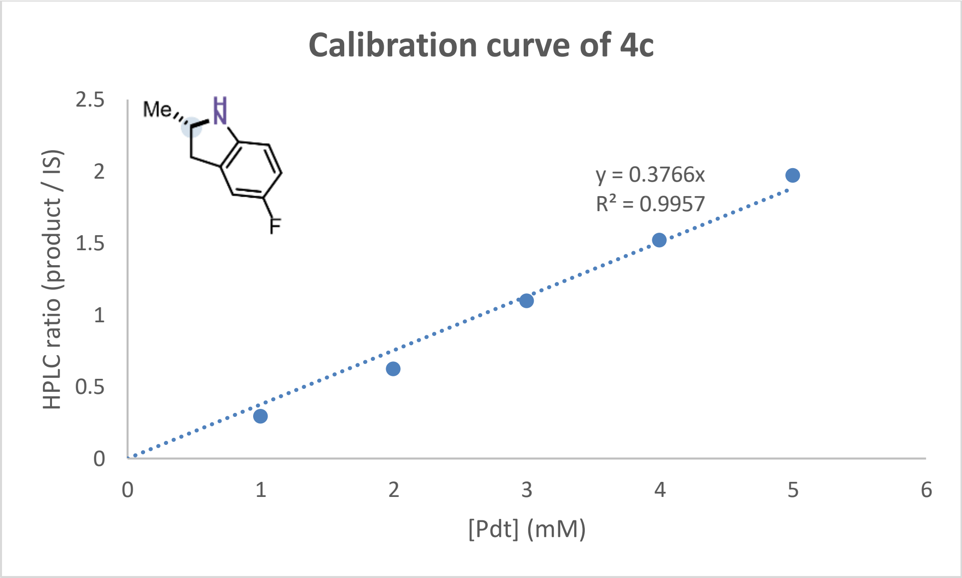

Data analysis for **P411-INS-5151** (M9-N, pH = **8.4**) catalyzed intramolecular C–H insertion with **3b** (5.0 mM, OD_{600} = 40):

Entry	Pdt	IS	Pdt/IS	[Pdt]/mM	[PC]/µM	Avg. TTN	SD TTN	Avg. Yield [%]	SD Yield [%]
1	17.9	95.3	0.19	0.51	0.49	1050	43	10	0.4
2	17.4	93.9	0.19	0.50	0.49				
3	19.1	95.5	0.20	0.54	0.49				

2,5-Dimethylindoline (4d)

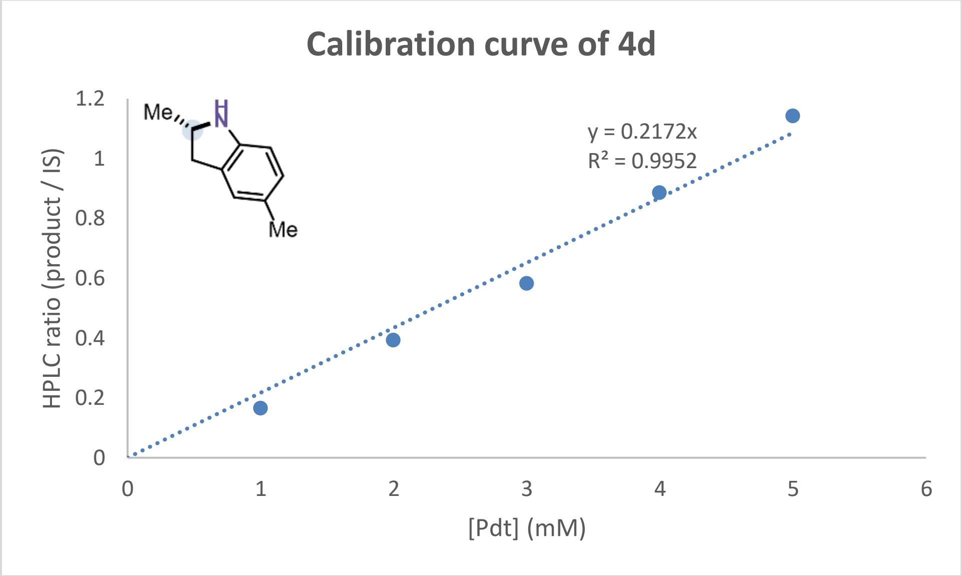

Data analysis for **P411-INS-5151** (M9-N, pH = **8.4**) catalyzed intramolecular C–H insertion with **3c** (5.0 mM, OD_{600} = 40):

Entry	Pdt	IS	Pdt/IS	[Pdt]/mM	[PC]/µM	Avg. TTN	SD TTN	Avg. Yield [%]	SD Yield [%]
1	48.2	6019.7	0.0080	0.11	0.49	211	18	2	0.2
2	40.1	5831.5	0.0069	0.10	0.49				
3	41.4	5869.6	0.0071	0.10	0.49				

3-(2-Methylindolin-1-yl)dihydrofuran-2(3H)-one (5)

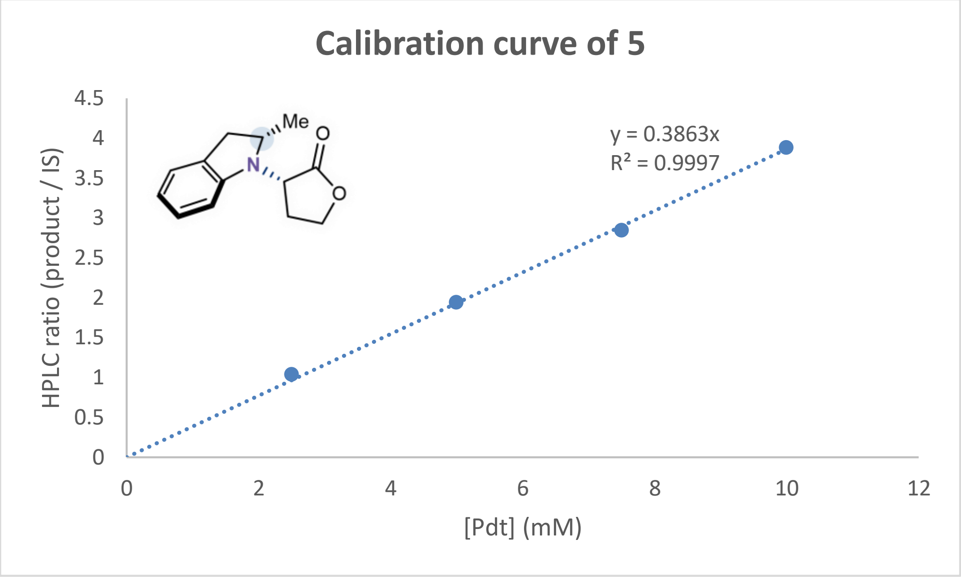

Data analysis for **L7_FL** (M9-N, pH = **7.4**) catalyzed intermolecular N–H insertion with **4a** (10.0 mM, OD_{600} = 30):

Entry	Pdt	IS	Pdt/IS	[Pdt]/mM	[PC]/µM	Avg. TTN	SD TTN	Avg. Yield [%]	SD Yield [%]
1	697.6	359.4	1.94	0.90	2.38	1985	115	47	2.7
2	621.8	357.6	1.74	1.09	2.38				
3	640.6	358.8	1.79	1.07	2.38				

VII. Preparative-Scale Enzymatic Synthesis

General procedure for 1-mmol scale enzymatic intramolecular C–H aminations with 3a:

In a 500-mL flask, a suspension of 150 mL *E. coli* expressing variant **P411-INS-5151** (OD_{600} = 40) in M9-N (pH = 8.4), 45 mL D-glucose (500 mM in M9-N, pH = 8.4), and 5 mL 1-azido-2-propylbenzene **3a** (161.2 mg, 200 mM in EtOH) were added sequentially under anaerobic conditions. The flask was capped and sealed with parafilm inside the anaerobic chamber. The mixture was shaken at 250 rpm in a shaker outside of an anaerobic chamber overnight.

After the reaction was completed, the reaction mixture was transferred to two 500-mL centrifuge bottles. The aqueous phase was extracted with organic solvent (hexane/EtOAc = 1:2, 120 mL × 4). The organic layers were combined in an Erlenmeyer flask, dried over Na_2SO_4, and concentrated under reduced pressure. Purification by preparative TLC with dichloromethane (with 0.5% NEt_3) as eluents afforded the cyclized product as a dark oil. TTNs were calculated based on measured protein concentration using CO binding and isolated product yield.

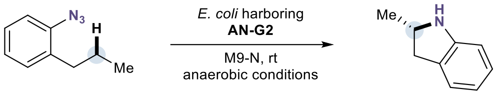

3a 4a

(*S*)-2-Methylindoline

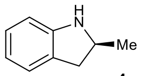

4a

[largescale](*S*)-**4a**: 79.8 mg, 60% yield, 2900 TTN, 92:8 *er* **^{1}H NMR** (400 MHz, $CDCl_3$) δ 7.08 (d, *J* = 7.3 Hz, 1H), 7.02 (dddd, *J* = 9.1, 7.6, 1.4, 0.8 Hz, 1H), 6.71 (td, *J* = 7.4, 1.0 Hz, 1H), 6.64 (d, *J* = 7.8 Hz, 1H), 4.01 (ddq, *J* = 8.5, 7.7, 6.2 Hz, 1H), 3.15 (dd, *J* = 15.3, 8.4 Hz, 1H), 2.65 (dd, *J* = 15.4, 7.7 Hz, 1H), 1.31 (d, *J* = 6.2 Hz, 3H). **^{13}C NMR** (101 MHz, $CDCl_3$) δ 150.6, 129.2, 127.4, 124.9, 119.0, 109.7, 55.4, 37.9, 22.3.

General procedure for 1-mmol scale enzymatic intramolecular C–H aminations with 3e:

In a 500-mL flask, a suspension of 150 mL *E. coli* expressing variant **P411-INS-5151** (OD_{600} = 40) in M9-N (pH = 8.4), 45 mL D-glucose (500 mM in M9-N, pH = 8.4), and 5 mL 1-azido-2-propylbenzene **3a** (175.2 mg, 200 mM in EtOH) were added sequentially under anaerobic conditions. The flask was capped and sealed with parafilm inside the anaerobic chamber. The mixture was shaken at 250 rpm in a shaker outside of an anaerobic chamber overnight.

After the reaction was completed, the reaction mixture was transferred to two 500-mL centrifuge bottles. The aqueous phase was extracted with organic solvent (hexane/EtOAc = 1:2, 120 mL × 4). The organic layers were combined in an Erlenmeyer flask, dried over Na_2SO_4, and concentrated under reduced pressure. Purification by preparative TLC with EtOAc/hexanes (10% hexanes in EtOAc) as eluents afforded the cyclized product as a dark oil. TTNs were calculated based on measured protein concentration using CO binding and isolated product yield.

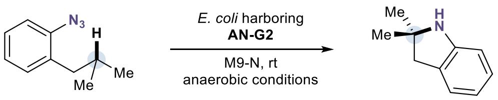

3e 4e

2,2-Dimethylindoline

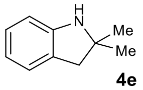

4e

largescale **4e**: 58.6 mg, 40% yield, 1470 TTN ^{1}**H NMR** (400 MHz, $CDCl_3$) δ 7.06 (d, *J* = 7.3 Hz, 1H), 7.01 (t, *J* = 7.6 Hz, 1H), 6.69 (t, *J* = 7.4 Hz, 1H), 6.58 (d, *J* = 7.7 Hz, 1H), 2.85 (s, 2H), 1.33 (s, 6H). ^{13}**C NMR** (101 MHz, $CDCl_3$) δ 129.7, 127.6, 125.3, 120.2, 117.5, 111.2, 62.3, 44.1, 28.9. **HRMS** (FD) Calcd for $C_{10}H_{13}N$ [M] 147.1048, found 147.1046.

General procedure for 0.5-mmol scale enzymatic N–H insertion reactions:

In a 250-mL flask, a suspension of 45 mL *E. coli* expressing variant **L7_FL** (OD_{600} = 40) in M9-N (pH = 7.4), 2.5 mL D-glucose (500 mM in M9-N, pH = 7.4), 1.25 mL (*S*)-2-methylindoline **4a** (66.6 mg, 1.0 equiv. 400 mM in EtOH), and 1.25 mL lactone diazo (LAD, 67.2 mg, 1.2 equiv. 480 mM in EtOH) were added sequentially under anaerobic conditions. The flask was capped and sealed with parafilm inside the anaerobic chamber. The mixture was shaken at 250 rpm in a shaker outside of an anaerobic chamber overnight.

After the reaction was completed, the reaction mixture was transferred to four 50-mL Eppendorf tubes. The aqueous phase was extracted with organic solvent (hexane/EtOAc = 1:2, 120 mL × 4). The organic layers were combined in an Erlenmeyer flask, dried over Na_2SO_4, and concentrated under reduced pressure. Purification by silica column chromatography with EtOAc/hexanes as eluents afforded the desired product. TTNs were calculated based on measured protein concentration using CO binding and isolated product yield.

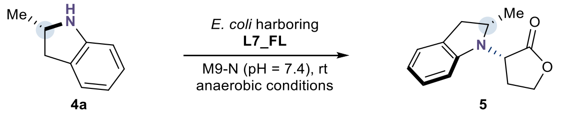

(S)-3-((S)-2-Methylindolin-1-yl)dihydrofuran-2(3H)-one

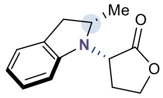

5

5: 46.6 mg, 43% yield, 2770 TTN, 98:2 *er*, >96:4 *dr* ^{1}**H NMR** (400 MHz, $CDCl_3$) δ 7.11 – 6.97 (m, 2H), 6.72 (t, *J* = 7.4 Hz, 1H), 6.30 (d, *J* = 7.9 Hz, 1H), 4.54 (td, *J* = 9.2, 1.6 Hz, 1H), 4.36 – 4.20 (m, 2H), 3.76 (ddq, *J* = 9.5, 8.7, 6.1 Hz, 1H), 3.18 (dd, *J* = 15.5, 8.6 Hz, 1H), 2.71 (dd, *J* = 15.4, 9.4 Hz, 1H), 2.60 – 2.43 (m, 1H), 2.42 – 2.29 (m, 1H), 1.40 (d, *J* = 6.1 Hz, 3H). ^{13}**C NMR** (101 MHz, $CDCl_3$) δ 174.8, 148.1, 129.8, 127.4, 124.9, 119.2, 108.8, 65.7, 60.0, 55.1, 37.3, 22.7, 20.2. **HRMS** (FD) Calcd for $C_{13}H_{15}NO_2$ [M] 217.1103, found 217.1104.

General procedure for 0.5-mmol scale enzymatic non-canonical amino acid synthesis:

In a 100-mL flask, a suspension of 25 mL kPi (pH = 8.0), L-serine (52.5 mg as a solid), (*S*)-2-methylindoline **4a** (66.6 mg, 1.0 equiv. 400 mM in EtOH), and 300 μL purified *Tm*TrpB synthase **TmE3** (238 μM in KPi buffer, 2.856 μM final concentration) were added sequentially under aerobic conditions. The flask was capped and sealed with parafilm. The mixture was shaken at 250 rpm in a shaker overnight.

After the reaction was completed, the reaction mixture was transferred to a 100-mL round-bottom flask. The aqueous phase was concentrated under reduced pressure. Purification by reverse column chromatography with EtOAc/hexanes as eluents afforded the desired product. TTNs were calculated based on measured protein concentration using CO binding and isolated product yield.

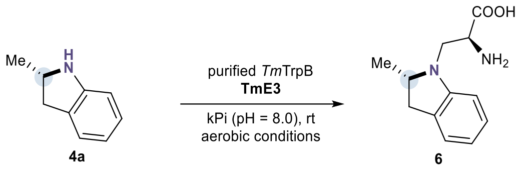

(2S)-2-Amino-3-(2-methylindolin-1-yl)propanoic acid

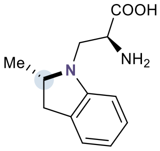

6

6: 32.5 mg, 30% yield, 2100 TTN, >94:6 *dr* **^{1}H NMR** (400 MHz, D_2O) δ 7.14 (tdd, *J* = 7.7, 4.2, 1.3 Hz, 2H), 6.73 (t, *J* = 7.4 Hz, 1H), 6.62 (d, *J* = 7.8 Hz, 1H), 3.82 – 3.72 (m, 1H), 3.69 (dd, *J* = 7.4, 5.5 Hz, 1H), 3.43 (qd, *J* = 14.8, 6.4 Hz, 2H), 3.15 (dd, *J* = 15.7, 8.4 Hz, 1H), 2.61 (dd, *J* = 15.7, 7.5 Hz, 1H), 1.23 (d, *J* = 6.2 Hz, 3H). **^{13}C NMR** (101 MHz, D_2O) δ 178.3, 151.8, 129.6, 127.5, 124.7, 118.0, 106.8, 61.3, 55.3, 50.0, 36.4, 17.8. **HRMS** (FD) Calcd for $C_{12}H_{16}N_2O_2$ [M] 220.1211, found 220.1214.

VIII. Chiral-Phase HPLC Traces

The absolute stereochemistry for enzymatic products **2a** and **4a** were assigned to be *R* and *S*, respectively, by comparing with their optical pure standard product. All other products **2b–2g**, **2j**, **4b–4d** were assigned by analogy. Before analysis of *ee*, all pyrrolidine products were protected by the benzoyl group, while indoline products were not.

Determination of absolute stereochemistry for **2a**:

Chiral-phase HPLC conditions: Chiralpak IC, 25% *i*-PrOH in hexane, 1.5 mL/min, 25 ºC, 210 nm

Racemic 2-Phenylpyrrolidine:

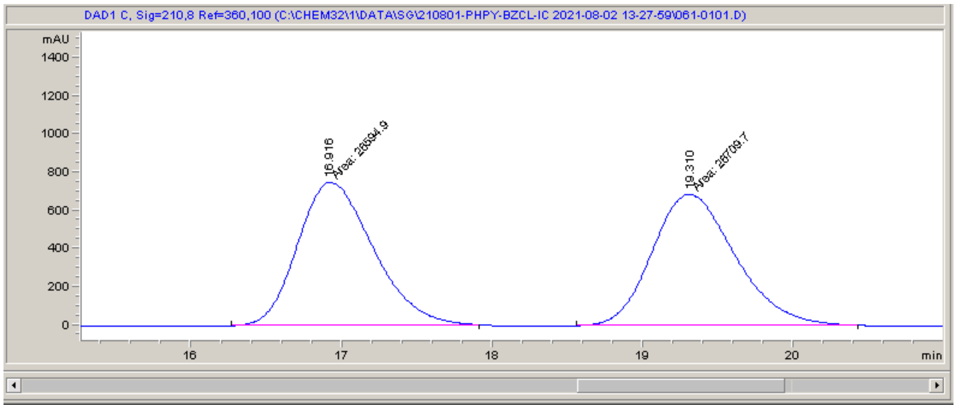

#	Time	Area	Height	Width	Area%	Symmetry
1	16.916	26594.9	753.9	0.5879	49.892	0.707
2	19.31	26709.7	691.5	0.6438	50.108	0.799

(*S*)-Phenylpyrrolidine: commercially available enantiopure compound

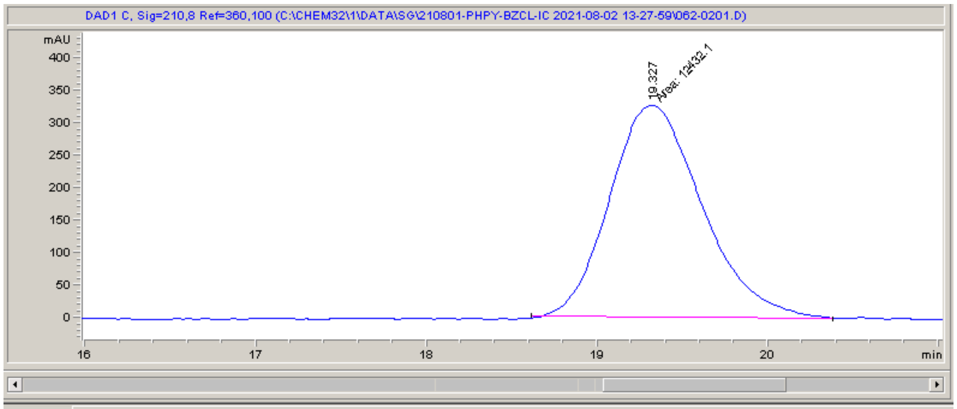

#	Time	Area	Height	Width	Area%	Symmetry
1	19.327	12432.1	328.2	0.6313	100.000	0.84

Determination of absolute stereochemistry for **4a**:

Chiral-phase HPLC conditions: Chiralpak IB, 2% *i*-PrOH in hexane, 1.0 mL/min, 25 ºC, 254 nm

Racemic 2-Methylindoline:

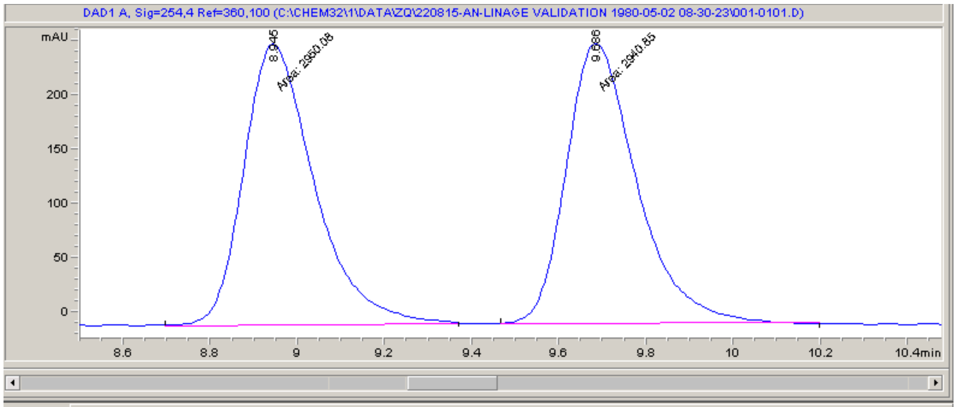

#	Time	Area	Height	Width	Area%	Symmetry
1	8.945	2950.1	259.6	0.1894	50.078	0.688
2	9.686	2940.9	259.3	0.189	49.922	0.698

(*R*)- 2-Methylindoline: commercially available enantiopure compound

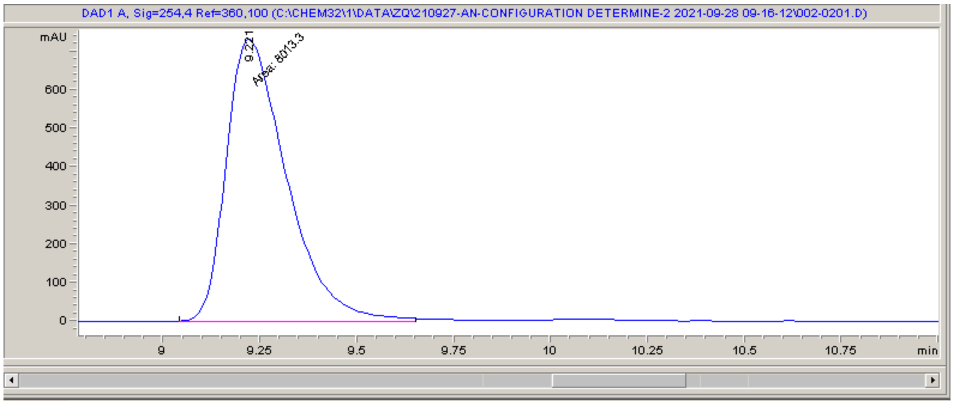

#	Time	Area	Height	Width	Area%	Symmetry
1	9.221	8013.3	735.8	0.1815	100.000	0.573

Determination of stereochemistry for all products:

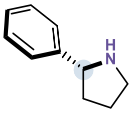

(*R*)-2-Phenylpyrrolidine (2a)

Chiral-phase HPLC conditions: Chiralpak IC, 25% *i*-PrOH in hexane, 1.5 mL/min, 25 ºC, 210 nm

Racemic 2a:

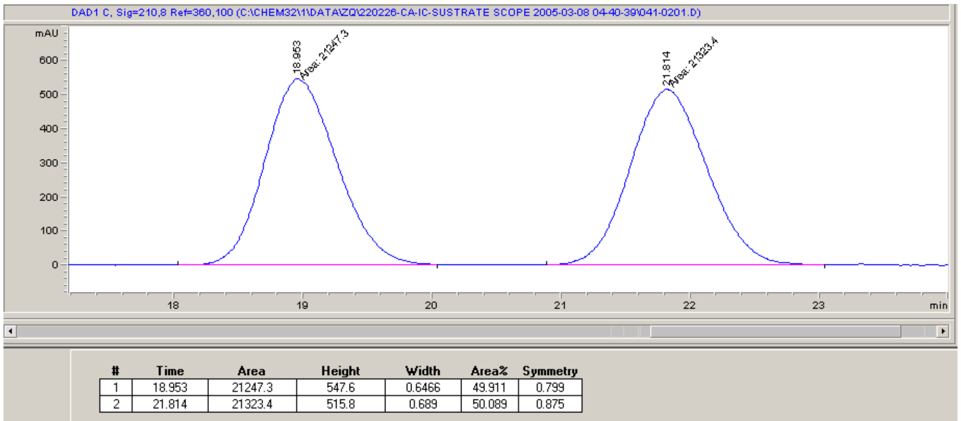

#	Time	Area	Height	Width	Area%	Symmetry
1	18.953	21247.3	547.6	0.6466	49.911	0.799
2	21.814	21323.4	515.8	0.689	50.089	0.875

Enzymatic preparation of 2a with P411-PYS-5149: 91:9 *er*

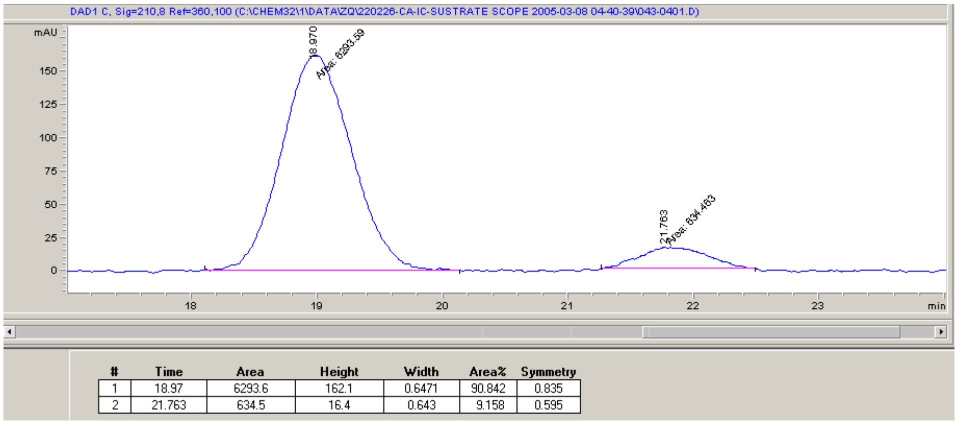

#	Time	Area	Height	Width	Area%	Symmetry
1	18.97	6293.6	162.1	0.6471	90.842	0.835
2	21.763	634.5	16.4	0.643	9.158	0.595

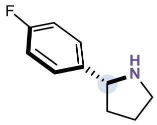

2-(4-Fluorophenyl)pyrrolidine (2b)

Chiral-phase HPLC conditions: Chiralpak IA, 10% *i*-PrOH in hexane, 1.5 mL/min, 25 ºC, 210 nm

Racemic 2b:

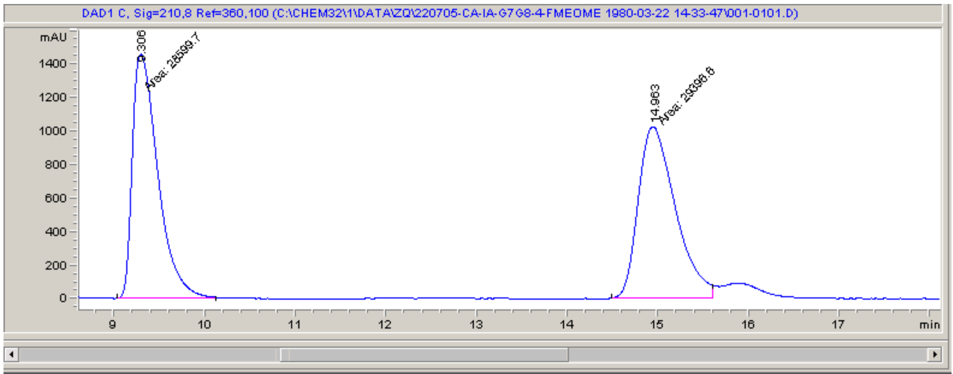

#	Time	Area	Height	Width	Area%	Symmetry
1	9.306	28599.7	1455.2	0.3275	49.313	0.512
2	14.963	29396.6	1021.4	0.4797	50.687	0.703

Enzymatic preparation of 2b with P411-PYS-5149: 92:8 *er*

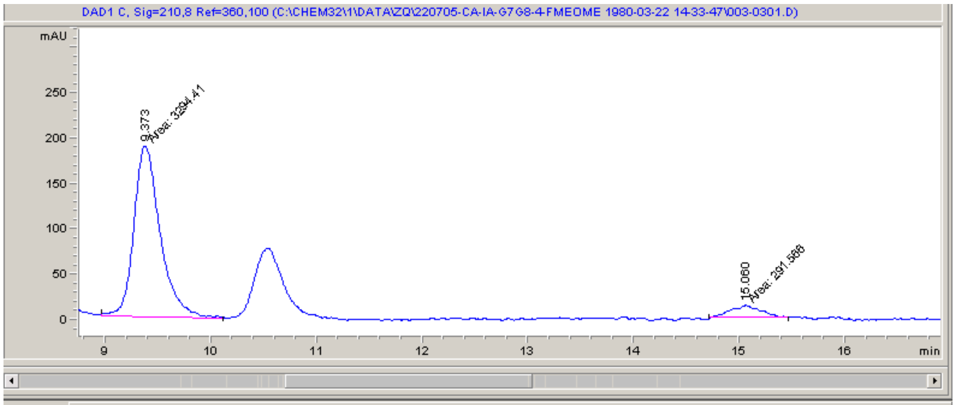

#	Time	Area	Height	Width	Area%	Symmetry
1	9.373	3294.4	188.7	0.291	91.869	0.718
2	15.06	291.6	13.8	0.3521	8.131	0.999

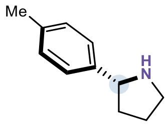

2-(*p*-Tolyl)pyrrolidine (2c)
Chiral-phase HPLC conditions: Chiralpak IA, 10% *i*-PrOH in hexane, 1.5 mL/min, 25 ºC, 210 nm

Racemic 2c:

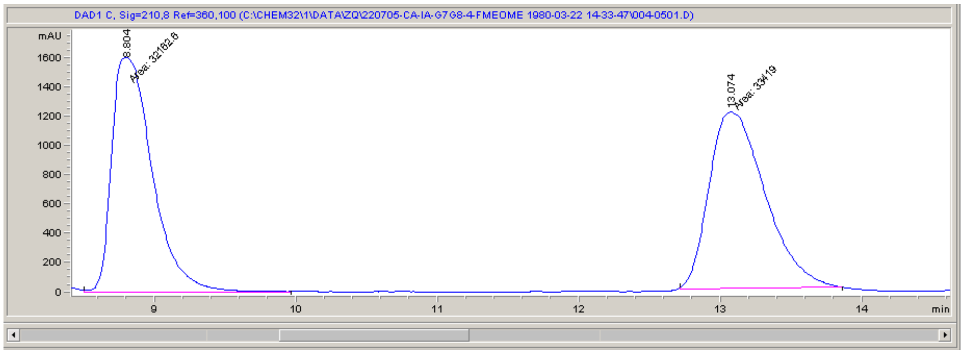

#	Time	Area	Height	Width	Area%	Symmetry
1	8.804	32162.6	1602.8	0.3344	49.042	0.553
2	13.074	33419	1208.5	0.4609	50.958	0.609

Enzymatic preparation of 2c with P411-PYS-5149: 92:8 *er*

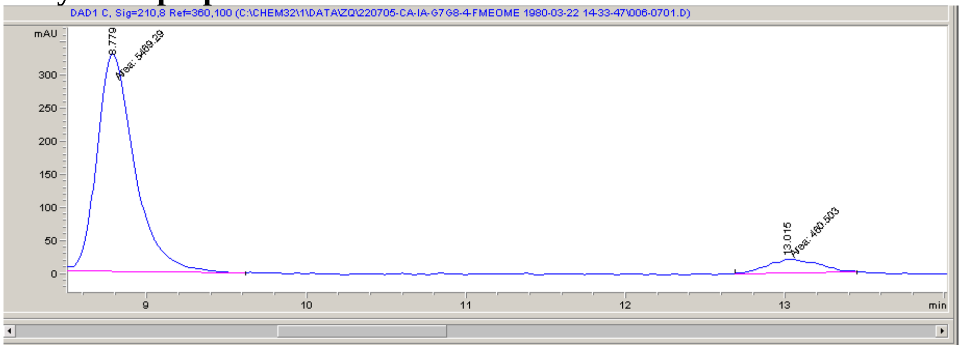

#	Time	Area	Height	Width	Area%	Symmetry
1	8.779	5469.3	330.1	0.2762	92.234	0.666
2	13.015	460.5	20.3	0.378	7.766	0.7

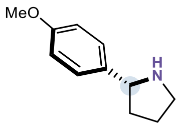

(*R*)-2-(4-Methoxyphenyl)pyrrolidine (2d)

Chiral-phase HPLC conditions: Chiralpak IA, 15% *i*-PrOH in hexane, 1.5 mL/min, 25 ºC, 210 nm

Racemic 2d:

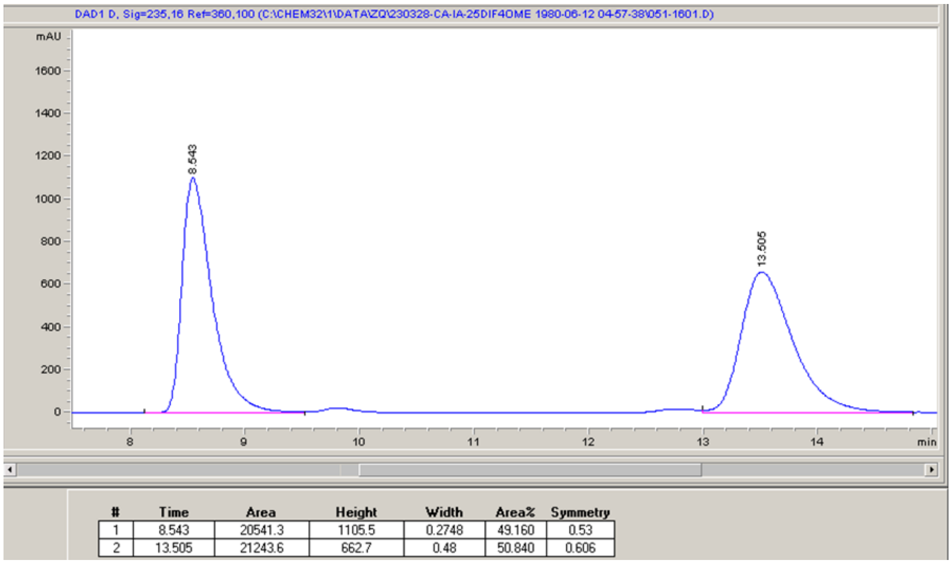

#	Time	Area	Height	Width	Area%	Symmetry
1	8.543	20541.3	1105.5	0.2748	49.160	0.53
2	13.505	21243.6	662.7	0.48	50.840	0.606

Enzymatic preparation of 2d with P411-PYS-5149: 99:1 *er*

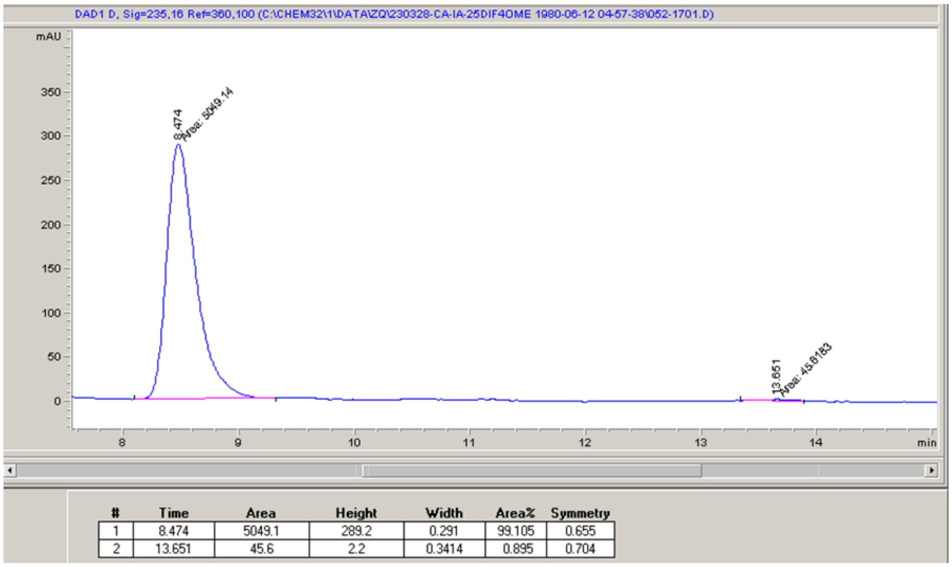

#	Time	Area	Height	Width	Area%	Symmetry
1	8.474	5049.1	289.2	0.291	99.105	0.655
2	13.651	45.6	2.2	0.3414	0.895	0.704

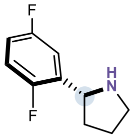

(*R*)-2-(2,5-Difluorophenyl)pyrrolidine (2e)
Chiral-phase HPLC conditions: Chiralpak IA, 7% *i*-PrOH in hexane, 1.5 mL/min, 25 ºC, 210 nm

Racemic 2e:

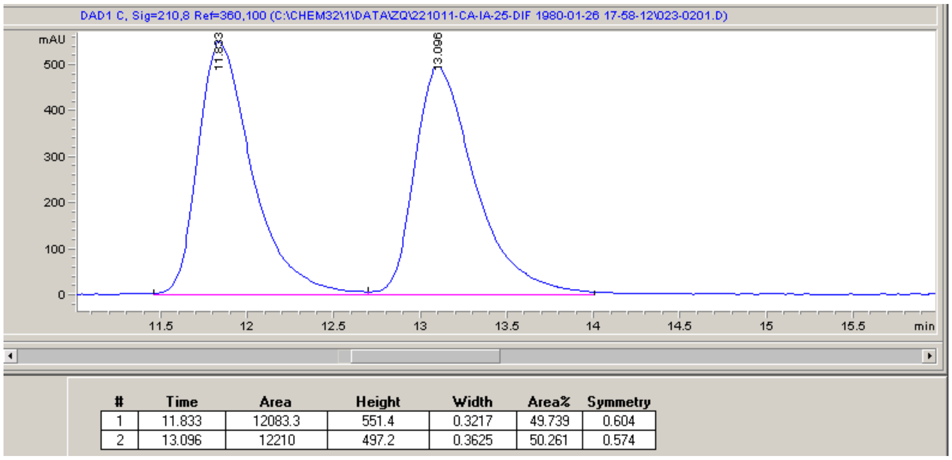

#	Time	Area	Height	Width	Area%	Symmetry
1	11.833	12083.3	551.4	0.3217	49.739	0.604
2	13.096	12210	497.2	0.3625	50.261	0.574

Enzymatic preparation of 2e with P411-PYS-5149: 85:15 *er*

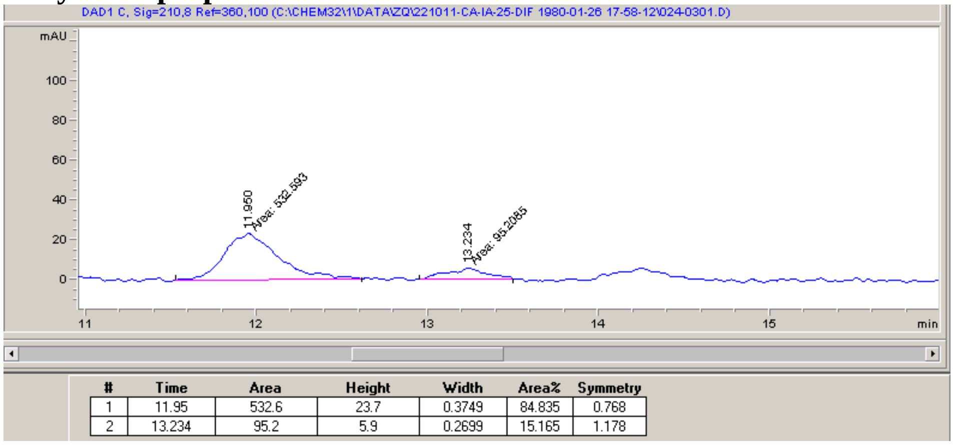

#	Time	Area	Height	Width	Area%	Symmetry
1	11.95	532.6	23.7	0.3749	84.835	0.768
2	13.234	95.2	5.9	0.2699	15.165	1.178

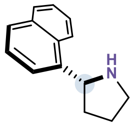

(*R*)-2-(Naphthalen-2-yl)pyrrolidine (2f)

Chiral-phase HPLC conditions: Chiralpak IA, 10% *i*-PrOH in hexane, 1.5 mL/min, 25 ºC, 210 nm

Racemic 2f:

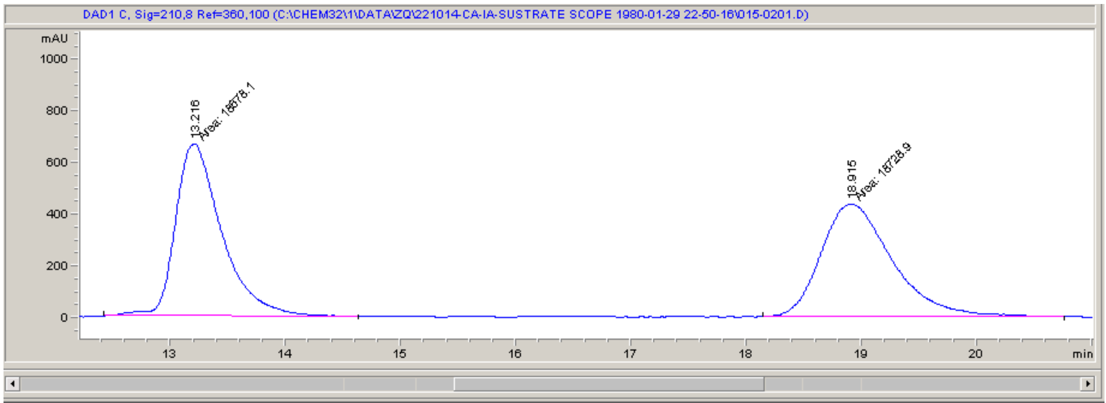

#	Time	Area	Height	Width	Area%	Symmetry
1	13.216	18678.1	667.2	0.4666	49.932	0.677
2	18.915	18728.9	437	0.7144	50.068	0.705

Enzymatic preparation of 2f with P411-PYS-5149: 60:40 *er*

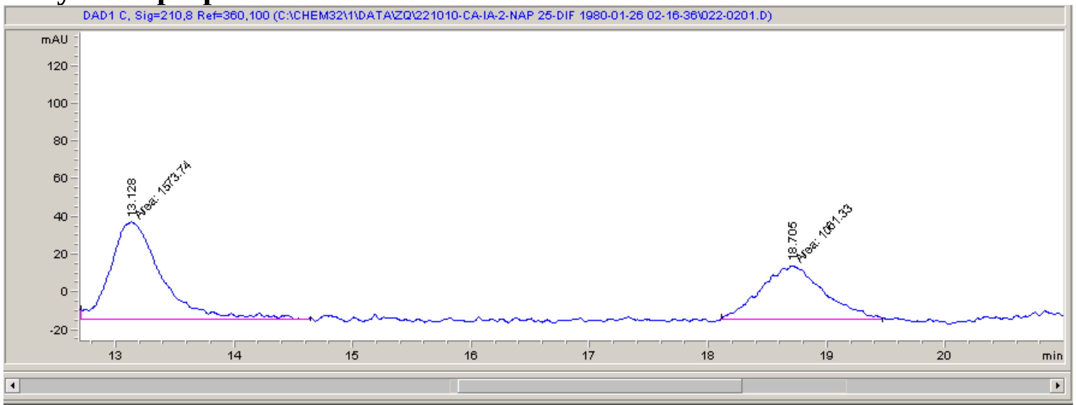

#	Time	Area	Height	Width	Area%	Symmetry
1	13.128	1573.7	52	0.5047	59.723	0.661
2	18.705	1061.3	28.5	0.6202	40.277	0.972

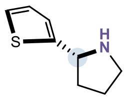

2-(Thiophen-2-yl)pyrrolidine (2g)
Chiral-phase HPLC conditions: Chiralpak IC, 25% *i*-PrOH in hexane, 1.5 mL/min, 25 ºC, 254 nm

Racemic 2g:

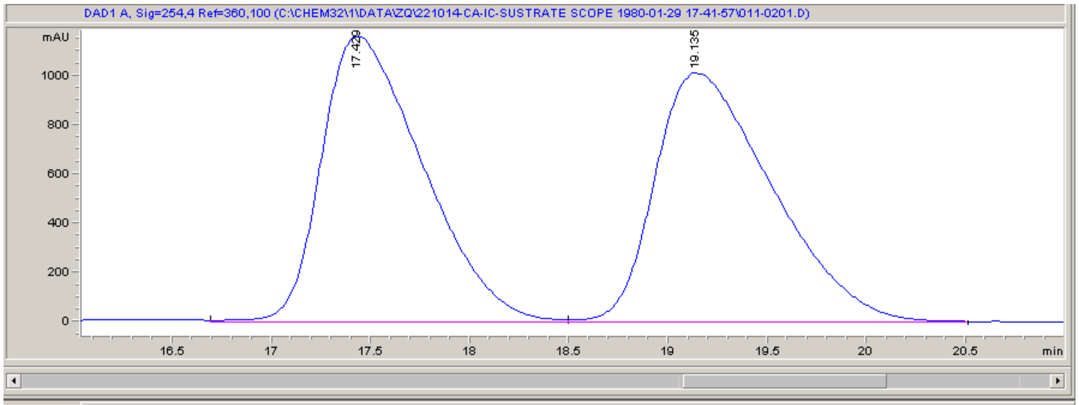

#	Time	Area	Height	Width	Area%	Symmetry
1	17.429	41411.2	1158.9	0.4749	49.901	0.503
2	19.135	41575.5	1013.2	0.5427	50.099	0.489

Enzymatic preparation of 2g with P411-PYS-5149: 23:77 *er*

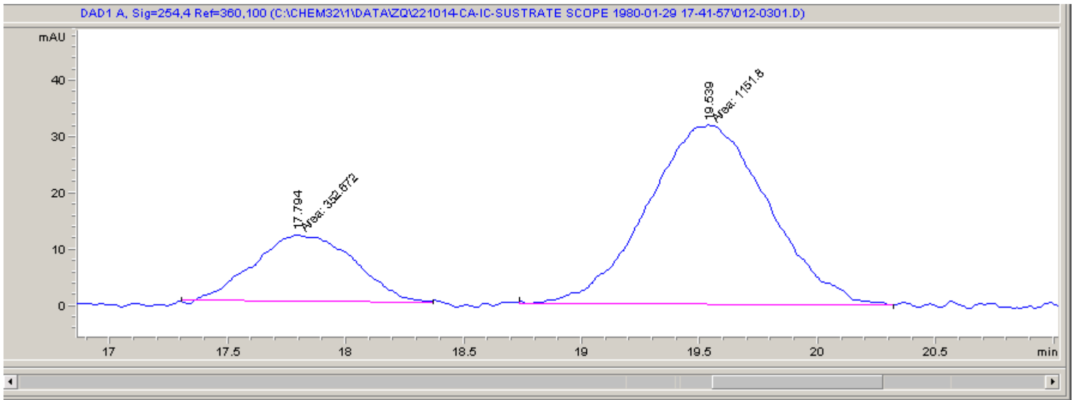

#	Time	Area	Height	Width	Area%	Symmetry
1	17.794	352.7	11.9	0.4941	23.442	0.765
2	19.539	1151.8	32	0.5998	76.558	1.003

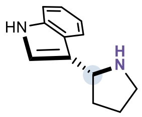

3-(Pyrrolidin-2-yl)-1H-indole (2h)
Chiral-phase HPLC conditions: Chiralpak IC, 20% *i*-PrOH in hexane, 1.5 mL/min, 25 ºC, 210 nm

Racemic 2h:

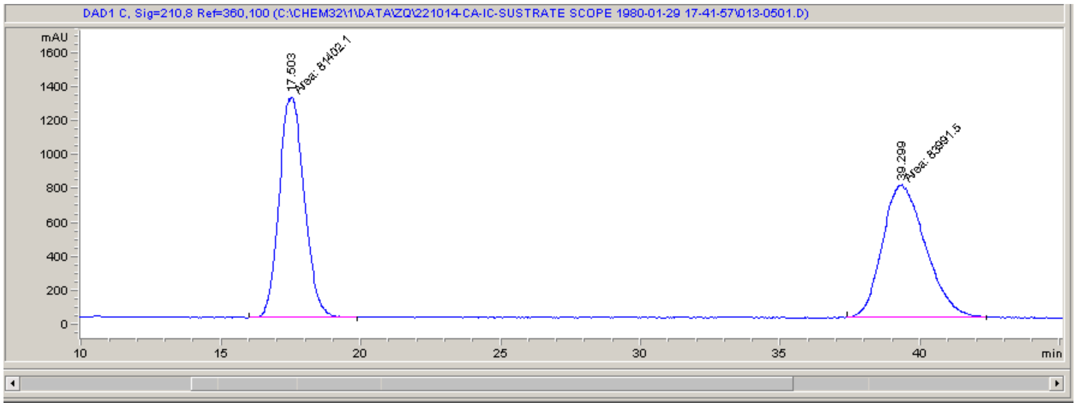

#	Time	Area	Height	Width	Area%	Symmetry
1	17.503	81402.1	1301.3	1.0426	49.217	0.941
2	39.299	83991.5	783.7	1.7862	50.783	0.762

Enzymatic preparation of 2h with P411-PYS-5149: 52:48 *er*

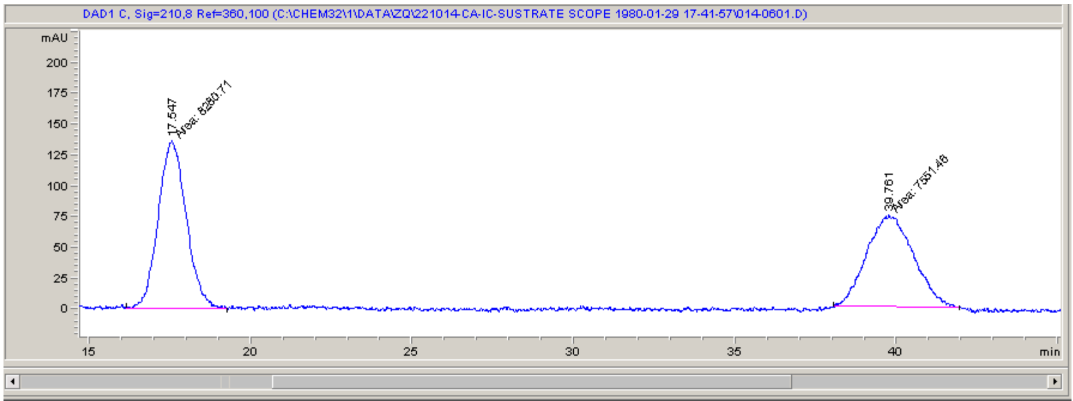

#	Time	Area	Height	Width	Area%	Symmetry
1	17.547	8280.7	136.3	1.0124	52.303	0.955
2	39.761	7551.5	74.5	1.6903	47.697	0.909

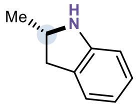

(*S*)-2-Methylindoline (4a)

Chiral-phase HPLC conditions: Chiralpak IB, 2% *i*-PrOH in hexane, 1.0 mL/min, 25 ºC, 254 nm

Racemic 4a:

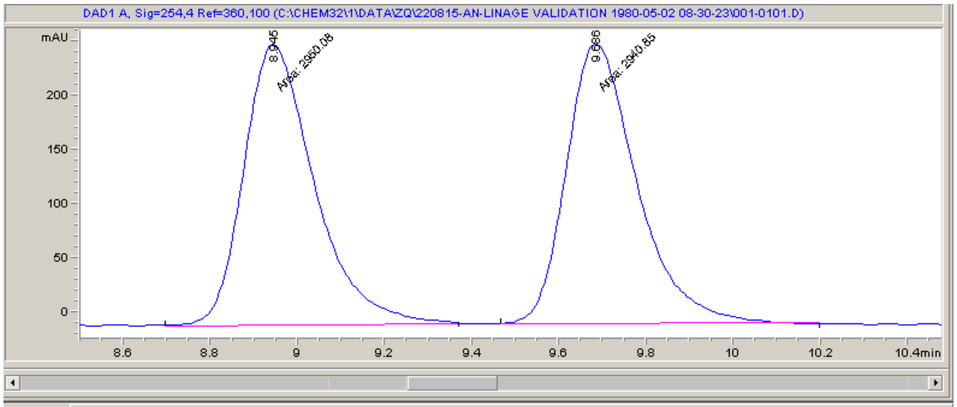

#	Time	Area	Height	Width	Area%	Symmetry
1	8.945	2950.1	259.6	0.1894	50.078	0.688
2	9.686	2940.9	259.3	0.189	49.922	0.698

Enzymatic preparation of 4a with P411-INS-5151: 8:92 *er*

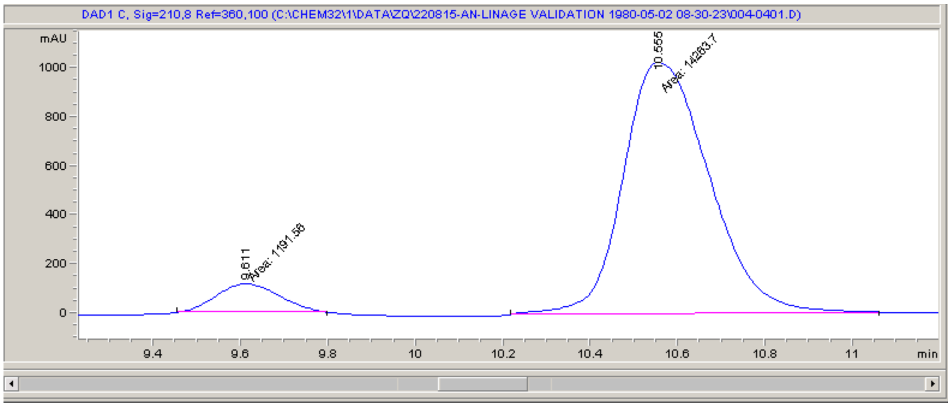

#	Time	Area	Height	Width	Area%	Symmetry
1	9.611	1191.6	117.2	0.1694	7.710	0.846
2	10.555	14263.7	1031.2	0.2305	92.290	0.666

Preparative scale enzymatic preparation of 4a with P411-INS-5151: 9:91 *er*

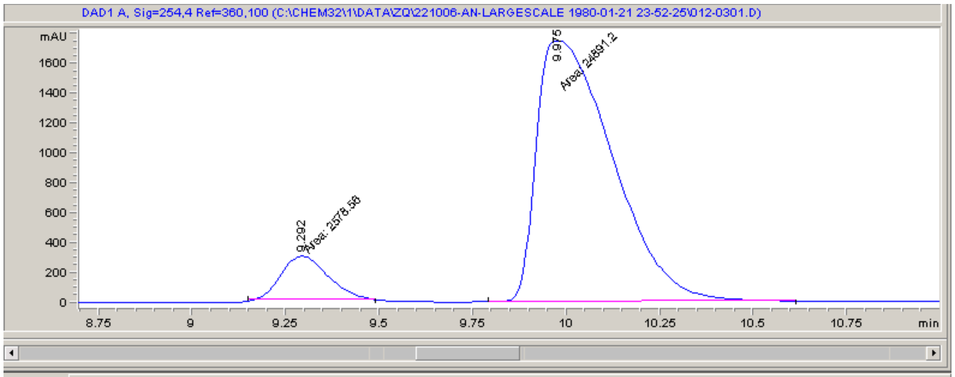

#	Time	Area	Height	Width	Area%	Symmetry
1	9.292	2578.6	293.8	0.1463	9.387	0.765
2	9.975	24891.2	1750.6	0.237	90.613	0.364

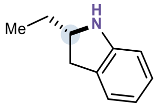

(*S*)-2-Ethylindoline (4b)
Chiral-phase HPLC conditions: Chiralpak IB, 2% *i*-PrOH in hexane, 1.0 mL/min, 25 ºC, 254 nm

Racemic 4b:

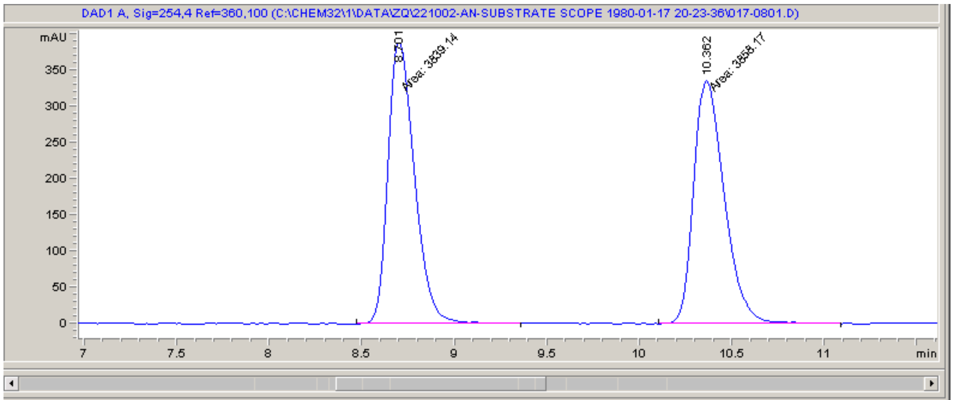

#	Time	Area	Height	Width	Area%	Symmetry
1	8.701	3839.1	388.6	0.1647	49.876	0.678
2	10.362	3858.2	336.4	0.1911	50.124	0.657

Enzymatic preparation of 4b with P411-INS-5151: 9:91 *er*

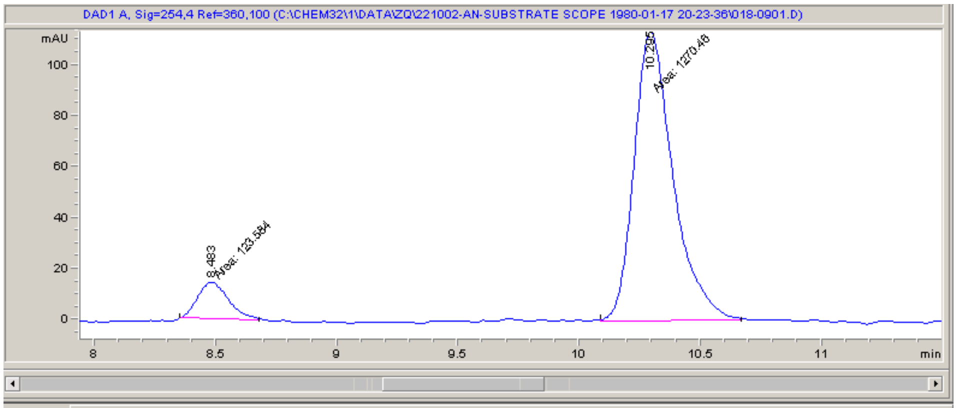

#	Time	Area	Height	Width	Area%	Symmetry
1	8.483	123.6	14.2	0.1449	8.865	0.784
2	10.295	1270.5	113.5	0.1866	91.135	0.717

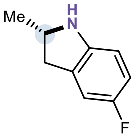

(*S*)-5-Fluoro-2-methylindoline (4c)
Chiral-phase HPLC conditions: Chiralpak IB, 2% *i*-PrOH in hexane, 1.0 mL/min, 25 ºC, 254 nm

Racemic 4c:

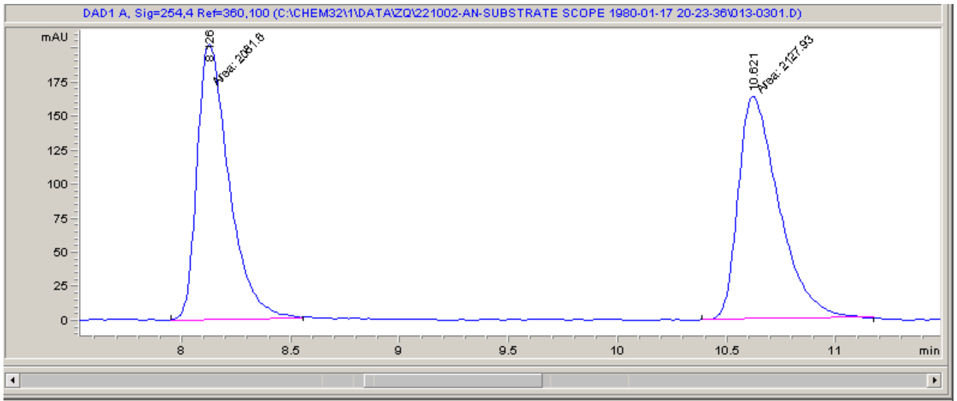

#	Time	Area	Height	Width	Area%	Symmetry
1	8.126	2081.6	203.3	0.1707	49.450	0.59
2	10.621	2127.9	163.7	0.2167	50.550	0.55

Enzymatic preparation of 4c with P411-INS-5151: 13:87 *er*

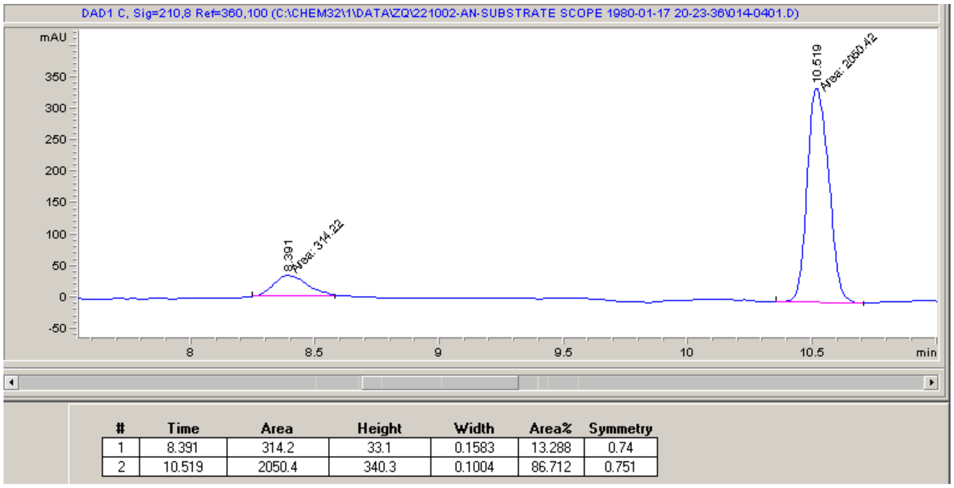

#	Time	Area	Height	Width	Area%	Symmetry
1	8.391	314.2	33.1	0.1583	13.288	0.74
2	10.519	2050.4	340.3	0.1004	86.712	0.751

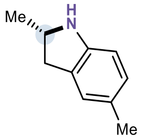

(*S*)-2,5-Dimethylindoline (4d)

Chiral-phase HPLC conditions: Chiralpak IB, 2% *i*-PrOH in hexane, 1.0 mL/min, 25 ºC, 254 nm

Racemic 4d:

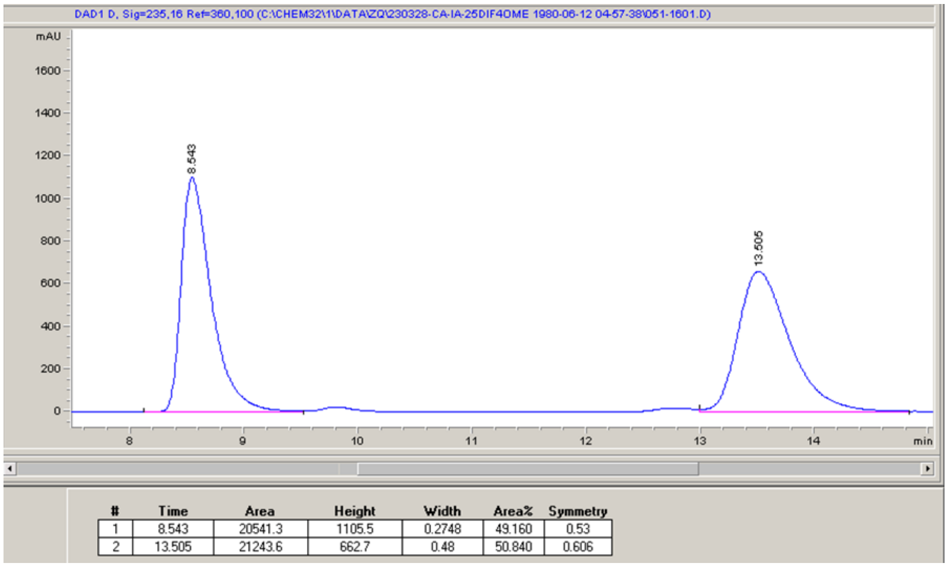

#	Time	Area	Height	Width	Area%	Symmetry
1	8.543	20541.3	1105.5	0.2748	49.160	0.53
2	13.505	21243.6	662.7	0.48	50.840	0.606

Enzymatic preparation of 4d with P411-INS-5151: 79:21 *er*

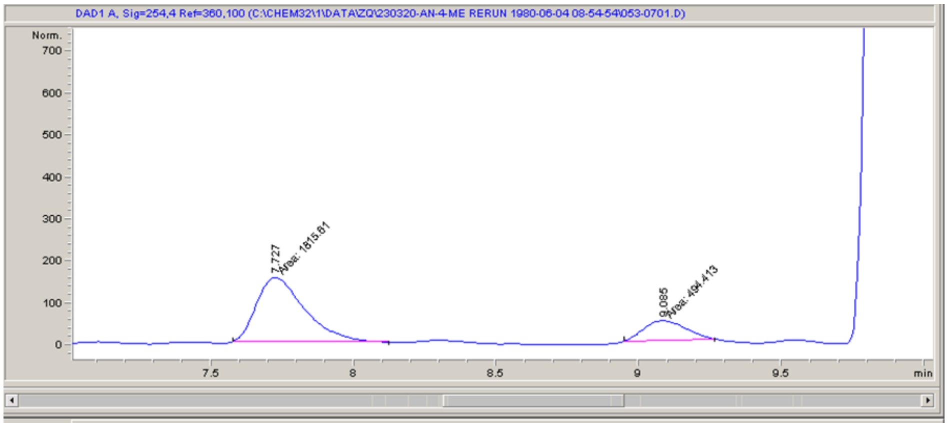

#	Time	Area	Height	Width	Area%	Symmetry
1	7.727	1815.6	153.8	0.1968	78.597	0.606
2	9.085	494.4	47.6	0.1731	21.403	0.793

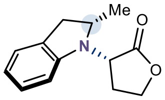

(S)-3-((S)-2-Methylindolin-1-yl)dihydrofuran-2(3H)-one (5):
Chiral-phase HPLC conditions: Chiralpak IC, 25% *i*-PrOH in hexane, 1.5 mL/min, 25 ºC, 254 nm

Racemic 5:

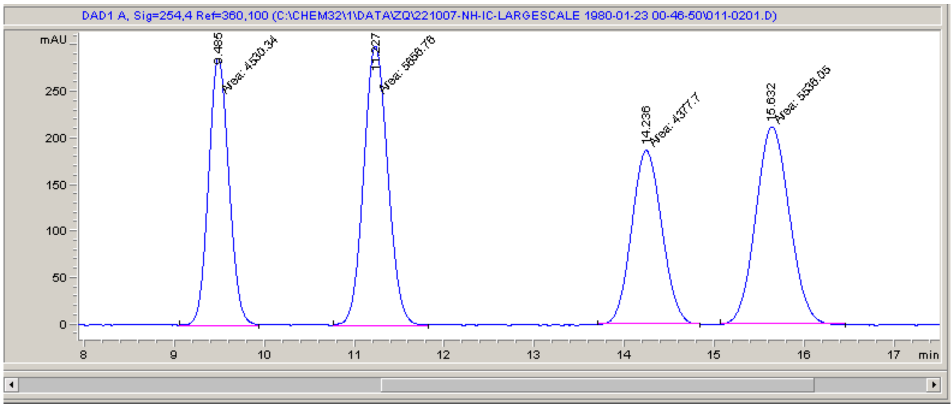

#	Time	Area	Height	Width	Area%	Symmetry
1	9.485	4530.3	287.4	0.2627	22.538	0.904
2	11.227	5656.8	301.1	0.3131	28.142	0.905
3	14.236	4377.7	187.5	0.3892	21.779	0.888
4	15.632	5536	212.6	0.434	27.541	0.884

Racemic 3-((S)-2-methylindolin-1-yl)dihydrofuran-2(3H)-one:

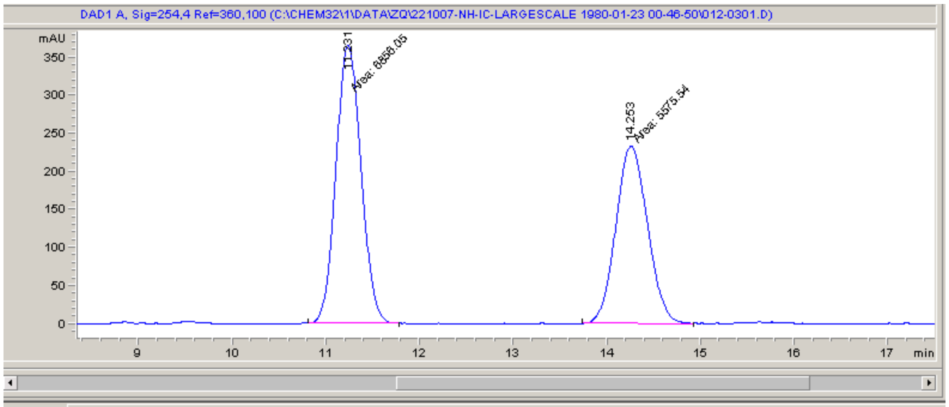

#	Time	Area	Height	Width	Area%	Symmetry
1	11.231	6856	366.5	0.3118	55.150	0.892
2	14.253	5575.5	234.3	0.3967	44.850	0.876

Preparative scale enzymatic preparation of 5 with L7_FL: 98:2 *er* and >24:1 dr

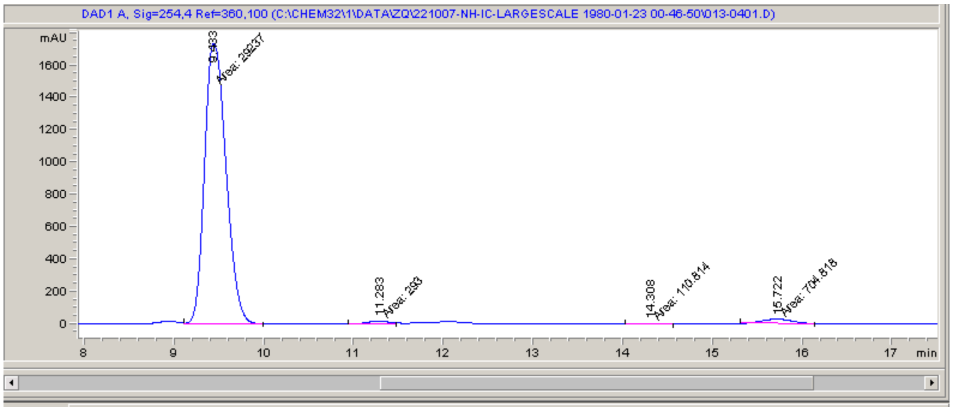

#	Time	Area	Height	Width	Area%	Symmetry
1	9.433	29237	1743.6	0.2795	96.347	0.684
2	11.283	293	16.8	0.2915	0.966	1.139
3	14.308	110.8	5.4	0.344	0.365	1.022
4	15.722	704.8	29.5	0.3986	2.323	1.055

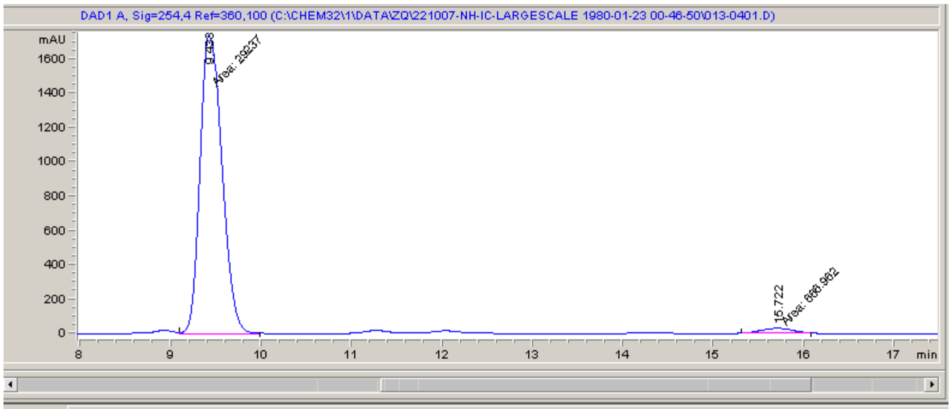

#	Time	Area	Height	Width	Area%	Symmetry
1	9.433	29237	1743.6	0.2795	97.770	0.684
2	15.722	667	28.7	0.3875	2.230	1.151

IX. Sequence Information

Mutations present in P411 variants described in this study:

P411 variant	Mutations relative to wild-type P450$_{BM3}$
P411-M177L (**P411-PYS-5141**)	A74G V78L A82L F87A P142S T175I M177L A184V S226R H236Q E252G I263Y H266V T268G A290V T327I A328V L353V I366V C400S T436L L437Q E442K
**P411-PYS-5142**	A74G **L75E** V78L A82L F87A P142S T175I M177L A184V S226R H236Q E252G I263Y H266V T268G A290V T327I A328V L353V I366V C400S T436L L437Q E442K
**P411-PYS-5143**	A74G **L75E** V78L A82L F87A P142S T175I M177L A184V S226R H236Q E252G I263Y H266V T268G A290V T327I A328V L353V I366V C400S T436L E442K
**P411-PYS-5144**	A74G **L75E** V78L A82L F87A P142S T175I M177L A184V S226R H236Q E252G I263Y H266V T268G A290V T327I A328V **A330Q** L353V I366V C400S T436L E442K
**P411-PYS-5145**	A74G **L75E** V78L A82L F87A **M118V** P142S T175I M177L A184V S226R H236Q E252G I263Y H266V T268G A290V T327I A328V **A330Q** L353V I366V C400S T436L E442K
**P411-PYS-5146**	A74G **L75E F77C** V78L A82L F87A **M118V** P142S T175I M177L A184V S226R H236Q E252G I263Y H266V T268G A290V T327I A328V **A330Q** L353V I366V C400S T436L E442K
**P411-PYS-5147**	**S72W** A74G **L75E F77C** V78L A82L F87A **M118V** P142S T175I M177L A184V S226R H236Q E252G I263Y H266V T268G A290V T327I A328V **A330Q** L353V I366V C400S T436L E442K
**P411-PYS-5148**	**S72W Q73A** A74G **L75E F77C** V78L A82L F87A **M118V** P142S T175I M177L A184V S226R H236Q E252G I263Y H266V T268G A290V T327I A328V **A330Q** L353V I366V C400S T436L E442K
**P411-PYS-5149**	**S72W Q73A** A74G **L75E F77C** V78L A82L F87A **M118V** P142S T175I M177L A184V S226R H236Q E252G I263Y H266V T268G A290V T327I A328V **A330Q** L353V I366V C400S **T436R** E442K
**P411-INS-5150**	**S72W Q73A** A74G **L75E F77C** V78L A82L F87A **M118V** P142S T175I M177L A184V S226R H236Q E252G I263Y H266V T268G A290V T327I A328V **A330Q** L353V I366V C400S T436L **L437P** E442K
**P411-INS-5151**	**S72W Q73A** A74G **L75E F77C** V78L A82L F87A **M118V** P142S T175I M177L **L181N** A184V S226R H236Q E252G I263Y H266V T268G A290V T327I A328V **A330Q** L353V I366V C400S T436L **L437P** E442K

Nucleotide and amino acid sequences of P411-M177L (P411-PYS-5141):
ATGACAATTAAAGAAATGCCTCAGCCAAAAACGTTTGGAGAGCTTAAAAATTTACCGTTATTAAACACAG
ATAAACCGGTTCAAGCTTTGATGAAAATTGCGGATGAATTAGGAGAAATCTTTAAATTCGAGGCGCCTGG
TCGTGTAACGCGCTACTTATCAAGTCAGCGTCTAATTAAAGAAGCATGCGATGAATCACGCTTTGATAAA
AACTTAAGTCAAGGTCTGAAATTTCTGCGTGATTTTCTTGGAGACGGGTTAGCCACAAGCTGGACGCATG
AAAAAAATTGGAAAAAAGCGCATAATATCTTACTTCCAAGCTTTAGTCAGCAGGCAATGAAAGGCTATCA
TGCGATGATGGTCGATATCGCCGTGCAGCTTGTTCAAAAGTGGGAGCGTCTAAATGCAGATGAGCATATT
GAAGTATCGGAAGACATGACACGTTTAACGCTTGATACAATTGGTCTTTGCGGCTTTAACTATCGCTTTA
ACAGCTTTTACCGAGATCAGCCTCATCCATTTATTATAAGTCTGGTCCGTGCACTGGATGAAGTAATGAA
CAAGCTGCAGCGAGCAAATCCAGACGACCCAGCTTATGATGAAAACAAGCGCCAGTTTCAAGAAGATATC
AAGGTGATGAACGACCTAGTAGATAAAATTATTGCAGATCGCAAAGCAAGGGGTGAACAAAGCGATGATT
TATTAACGCAGATGCTAAACGGAAAAGATCCAGAAACGGGTGAGCCGCTTGATGACGGGAACATTCGCTA
TCAAATTATTACATTCTTATATGCGGGAGTTGAAGGTACAAGTGGTCTTTTATCATTTGCGCTGTATTTC
TTAGTGAAAAATCCACATGTATTACAAAAAGTAGCAGAAGAAGCAGCACGAGTTCTAGTAGATCCTGTTC
CAAGCTACAAACAAGTCAAACAGCTTAAATATGTCGGCATGGTCTTAAACGAAGCGCTGCGCTTATGGCC
AATTGTTCCTGCGTTTTCCCTATATGCAAAAGAAGATACGGTGCTTGGAGGAGAATATCCTTTAGAAAAA
GGCGACGAAGTAATGGTTCTGATTCCTCAGCTTCACCGTGATAAAACAGTTTGGGGAGACGATGTGGAGG
AGTTCCGTCCAGAGCGTTTTGAAAATCCAAGTGCGATTCCGCAGCATGCGTTTAAACCGTTTGGAAACGG
TCAGCGTGCGTCTATCGGTCAGCAGTTCGCTCTTCATGAAGCAACGCTGGTACTTGGTATGATGCTAAAA
CACTTTGACTTTGAAGATCATACAAACTACGAGCTCGATATTAAAGAACTGCAGACGTTAAAACCTAAAG
GCTTTGTGGTAAAAGCAAAATCGAAAAAAATTCCGCTTGGCGGTATTCCTTCACCTAGCACTGAACAGTC
TGCTAAAAAAGTACGCAAAAAGGCAGAAAACGCTCATAATACGCCGCTGCTTGTGCTATACGGTTCAAAT
ATGGGTACCGCTGAAGGAACGGCGCGTGATTTAGCAGATATTGCAATGAGCAAAGGATTTGCACCGCAGG
TCGCAACGCTTGATTCACACGCCGGAAATCTTCCGCGCGAAGGAGCTGTATTAATTGTAACGGCGTCTTA
TAACGGTCATCCGCCTGATAACGCAAAGCAATTTGTCGACTGGTTAGACCAAGCGTCTGCTGATGAAGTA
AAAGGCGTTCGCTACTCCGTATTTGGATGCGGCGATAAAAACTGGGCTACTACGTATCAAAAAGTGCCTG
CTTTTATCGATGAAACGCTTGCCGCTAAAGGGGCAGAAAACATCGCTGACCGCGGTGAAGCAGATGCAAG
CGACGACTTTGAAGGCACATATGAAGAATGGCGTGAACATATGTGGAGTGACGTAGCAGCCTACTTTAAC
CTCGACATTGAAAACAGTGAAGATAATAAATCTACTCTTTCACTTCAATTTGTCGACAGCGCCGCGGATA
TGCCGCTTGCGAAAATGCACGGTGCGTTTTCAACGAACGTCGTAGCAAGCAAAGAACTTCAACAGCCAGG
CAGTGCACGAAGCACGCGACATCTTGAAATTGAACTTCCAAAAGAAGCTTCTTATCAAGAAGGAGATCAT
TTAGGTGTTATTCCTCGCAACTATGAAGGAATAGTAAACCGTGTAACAGCAAGGTTCGGCCTAGATGCAT
CACAGCAAATCCGTCTGGAAGCAGAAGAAGAAAAATTAGCTCATTTGCCACTCGCTAAAACAGTATCCGT
AGAAGAGCTTCTGCAATACGTGGAGCTTCAAGATCCTGTTACGCGCACGCAGCTTCGCGCAATGGCTGCT
AAAACGGTCTGCCCGCCGCATAAAGTAGAGCTTGAAGCCTTGCTTGAAAAGCAAGCCTACAAAGAACAAG
TGCTGGCAAAACGTTTAACAATGCTTGAACTGCTTGAAAAATACCCGGCGTGTGAAATGAAATTCAGCGA
ATTTATCGCCCTTCTGCCAAGCATACGCCCGCGCTATTACTCGATTTCTTCATCACCTCGTGTCGATGAA
AAACAAGCAAGCATCACGGTCAGCGTTGTCTCAGGAGAAGCGTGGAGCGGATATGGAGAATATAAAGGAA
TTGCGTCGAACTATCTTGCCGAGCTGCAAGAAGGAGATACGATTACGTGCTTTATTTCCACACCGCAGTC
AGAATTTACGCTGCCAAAAGACCCTGAAACGCCGCTTATCATGGTCGGACCGGGAACAGGCGTCGCGCCG
TTTAGAGGCTTTGTGCAGGCGCGCAAACAGCTAAAAGAACAAGGACAGTCACTTGGAGAAGCACATTTAT
ACTTCGGCTGCCGTTCACCTCATGAAGACTATCTGTATCAAGAAGAGCTTGAAAACGCCCAAAGCGAAGG
CATCATTACGCTTCATACCGCTTTTTCTCGCATGCCAAATCAGCCGAAAACATACGTTCAGCACGTAATG
GAACAAGACGGCAAGAAATTGATTGAACTTCTTGATCAAGGAGCGCACTTCTATATTTGCGGAGACGGAA
GCCAAATGGCACCTGCCGTTGAAGCAACGCTTATGAAAAGCTATGCTGACGTTCACCAAGTGAGTGAAGC
AGACGCTCGCTTATGGCTGCAGCAGCTAGAAGAAAAAGGCCGATACGCAAAAGACGTGTGGGCTGGGCTC
GAGCACCACCACCACCACCACTGA

MTIKEMPQPKTFGELKNLPLLNTDKPVQALMKIADELGEIFKFEAPGRVTRYLSSQRLIKEACDESRFDK
NLSQGLKFLRDFLGDGLATSWTHEKNWKKAHNILLPSFSQQAMKGYHAMMVDIAVQLVQKWERLNADEHI

EVSEDMTRLTLDTIGLCGFNYRFNSFYRDQPHPFIISLVRALDEVMNKLQRANPDDPAYDENKRQFQEDI
KVMNDLVDKIIADRKARGEQSDDLLTQMLNGKDPETGEPLDDGNIRYQIITFLYAGVEGTSGLLSFALYF
LVKNPHVLQKVAEEAARVLVDPVPSYKQVKQLKYVGMVLNEALRLWPIVPAFSLYAKEDTVLGGEYPLEK
GDEVMVLIPQLHRDKTVWGDDVEEFRPERFENPSAIPQHAFKPFGNGQRASIGQQFALHEATLVLGMMLK
HFDFEDHTNYELDIKELQTLKPKGFVVKAKSKKIPLGGIPSPSTEQSAKKVRKKAENAHNTPLLVLYGSN
MGTAEGTARDLADIAMSKGFAPQVATLDSHAGNLPREGAVLIVTASYNGHPPDNAKQFVDWLDQASADEV
KGVRYSVFGCGDKNWATTYQKVPAFIDETLAAKGAENIADRGEADASDDFEGTYEEWREHMWSDVAAYFN
LDIENSEDNKSTLSLQFVDSAADMPLAKMHGAFSTNVVASKELQQPGSARSTRHLEIELPKEASYQEGDH
LGVIPRNYEGIVNRVTARFGLDASQQIRLEAEEEKLAHLPLAKTVSVEELLQYVELQDPVTRTQLRAMAA
KTVCPPHKVELEALLEKQAYKEQVLAKRLTMLELLEKYPACEMKFSEFIALLPSIRPRYYSISSSPRVDE
KQASITVSVVSGEAWSGYGEYKGIASNYLAELQEGDTITCFISTPQSEFTLPKDPETPLIMVGPGTGVAP
FRGFVQARKQLKEQGQSLGEAHLYFGCRSPHEDYLYQEELENAQSEGIITLHTAFSRMPNQPKTYVQHVM
EQDGKKLIELLDQGAHFYICGDGSQMAPAVEATLMKSYADVHQVSEADARLWLQQLEEKGRYAKDVWAGL
EHHHHHH

Nucleotide and amino acid sequences of P411-PYS-5142:
ATGACAATTAAAGAAATGCCTCAGCCAAAAACGTTTGGAGAGCTTAAAAATTTACCGTTATTAAACACAG
ATAAACCGGTTCAAGCTTTGATGAAAATTGCGGATGAATTAGGAGAAATCTTTAAATTCGAGGCGCCTGG
TCGTGTAACGCGCTACTTATCAAGTCAGCGTCTAATTAAAGAAGCATGCGATGAATCACGCTTTGATAAA
AACTTAAGTCAAGGTGAGAAATTTCTGCGTGATTTTCTTGGAGACGGGTTAGCCACAAGCTGGACGCATG
AAAAAAATTGGAAAAAAGCGCATAATATCTTACTTCCAAGCTTTAGTCAGCAGGCAATGAAAGGCTATCA
TGCGATGATGGTCGATATCGCCGTGCAGCTTGTTCAAAAGTGGGAGCGTCTAAATGCAGATGAGCATATT
GAAGTATCGGAAGACATGACACGTTTAACGCTTGATACAATTGGTCTTTGCGGCTTTAACTATCGCTTTA
ACAGCTTTTACCGAGATCAGCCTCATCCATTTATTATAAGTCTGGTCCGTGCACTGGATGAAGTAATGAA
CAAGCTGCAGCGAGCAAATCCAGACGACCCAGCTTATGATGAAAACAAGCGCCAGTTTCAAGAAGATATC
AAGGTGATGAACGACCTAGTAGATAAAATTATTGCAGATCGCAAAGCAAGGGGTGAACAAAGCGATGATT
TATTAACGCAGATGCTAAACGGAAAAGATCCAGAAACGGGTGAGCCGCTTGATGACGGGAACATTCGCTA
TCAAATTATTACATTCTTATATGCGGGAGTTGAAGGTACAAGTGGTCTTTTATCATTTGCGCTGTATTTC
TTAGTGAAAAATCCACATGTATTACAAAAAGTAGCAGAAGAAGCAGCACGAGTTCTAGTAGATCCTGTTC
CAAGCTACAAACAAGTCAAACAGCTTAAATATGTCGGCATGGTCTTAAACGAAGCGCTGCGCTTATGGCC
AATTGTTCCTGCGTTTTCCCTATATGCAAAAGAAGATACGGTGCTTGGAGGAGAATATCCTTTAGAAAAA
GGCGACGAAGTAATGGTTCTGATTCCTCAGCTTCACCGTGATAAAACAGTTTGGGGAGACGATGTGGAGG
AGTTCCGTCCAGAGCGTTTTGAAAATCCAAGTGCGATTCCGCAGCATGCGTTTAAACCGTTTGGAAACGG
TCAGCGTGCGTCTATCGGTCAGCAGTTCGCTCTTCATGAAGCAACGCTGGTACTTGGTATGATGCTAAAA
CACTTTGACTTTGAAGATCATACAAACTACGAGCTCGATATTAAAGAACTGCAGACGTTAAAACCTAAAG
GCTTTGTGGTAAAAGCAAAATCGAAAAAAATTCCGCTTGGCGGTATTCCTTCACCTAGCACTGAACAGTC
TGCTAAAAAAGTACGCAAAAAGGCAGAAAACGCTCATAATACGCCGCTGCTTGTGCTATACGGTTCAAAT
ATGGGTACCGCTGAAGGAACGGCGCGTGATTTAGCAGATATTGCAATGAGCAAAGGATTTGCACCGCAGG
TCGCAACGCTTGATTCACACGCCGGAAATCTTCCGCGCGAAGGAGCTGTATTAATTGTAACGGCGTCTTA
TAACGGTCATCCGCCTGATAACGCAAAGCAATTTGTCGACTGGTTAGACCAAGCGTCTGCTGATGAAGTA
AAAGGCGTTCGCTACTCCGTATTTGGATGCGGCGATAAAAACTGGGCTACTACGTATCAAAAAGTGCCTG
CTTTTATCGATGAAACGCTTGCCGCTAAAGGGGCAGAAAACATCGCTGACCGCGGTGAAGCAGATGCAAG
CGACGACTTTGAAGGCACATATGAAGAATGGCGTGAACATATGTGGAGTGACGTAGCAGCCTACTTTAAC
CTCGACATTGAAAACAGTGAAGATAATAAATCTACTCTTTCACTTCAATTTGTCGACAGCGCCGCGGATA
TGCCGCTTGCGAAAATGCACGGTGCGTTTTCAACGAACGTCGTAGCAAGCAAAGAACTTCAACAGCCAGG
CAGTGCACGAAGCACGCGACATCTTGAAATTGAACTTCCAAAAGAAGCTTCTTATCAAGAAGGAGATCAT
TTAGGTGTTATTCCTCGCAACTATGAAGGAATAGTAAACCGTGTAACAGCAAGGTTCGGCCTAGATGCAT
CACAGCAAATCCGTCTGGAAGCAGAAGAAGAAAAATTAGCTCATTTGCCACTCGCTAAAACAGTATCCGT
AGAAGAGCTTCTGCAATACGTGGAGCTTCAAGATCCTGTTACGCGCACGCAGCTTCGCGCAATGGCTGCT
AAAACGGTCTGCCCGCCGCATAAAGTAGAGCTTGAAGCCTTGCTTGAAAAGCAAGCCTACAAAGAACAAG
TGCTGGCAAAACGTTTAACAATGCTTGAACTGCTTGAAAAATACCCGGCGTGTGAAATGAAATTCAGCGA

ATTTATCGCCCTTCTGCCAAGCATACGCCCGCGCTATTACTCGATTTCTTCATCACCTCGTGTCGATGAA
AAACAAGCAAGCATCACGGTCAGCGTTGTCTCAGGAGAAGCGTGGAGCGGATATGGAGAATATAAAGGAA
TTGCGTCGAACTATCTTGCCGAGCTGCAAGAAGGAGATACGATTACGTGCTTTATTTCCACACCGCAGTC
AGAATTTACGCTGCCAAAAGACCCTGAAACGCCGCTTATCATGGTCGGACCGGGAACAGGCGTCGCGCCG
TTTAGAGGCTTTGTGCAGGCGCGCAAACAGCTAAAAGAACAAGGACAGTCACTTGGAGAAGCACATTTAT
ACTTCGGCTGCCGTTCACCTCATGAAGACTATCTGTATCAAGAAGAGCTTGAAAACGCCCAAAGCGAAGG
CATCATTACGCTTCATACCGCTTTTTCTCGCATGCCAAATCAGCCGAAAACATACGTTCAGCACGTAATG
GAACAAGACGGCAAGAAATTGATTGAACTTCTTGATCAAGGAGCGCACTTCTATATTTGCGGAGACGGAA
GCCAAATGGCACCTGCCGTTGAAGCAACGCTTATGAAAAGCTATGCTGACGTTCACCAAGTGAGTGAAGC
AGACGCTCGCTTATGGCTGCAGCAGCTAGAAGAAAAAGGCCGATACGCAAAAGACGTGTGGGCTGGGCTC
GAGCACCACCACCACCACCACTGA

MTIKEMPQPKTFGELKNLPLLNTDKPVQALMKIADELGEIFKFEAPGRVTRYLSSQRLIKEACDESRFDK
NLSQGEKFLRDFLGDGLATSWTHEKNWKKAHNILLPSFSQQAMKGYHAMMVDIAVQLVQKWERLNADEHI
EVSEDMTRLTLDTIGLCGFNYRFNSFYRDQPHPFIISLVRALDEVMNKLQRANPDDPAYDENKRQFQEDI
KVMNDLVDKIIADRKARGEQSDDLLTQMLNGKDPETGEPLDDGNIRYQIITFLYAGVEGTSGLLSFALYF
LVKNPHVLQKVAEEAARVLVDPVPSYKQVKQLKYVGMVLNEALRLWPIVPAFSLYAKEDTVLGGEYPLEK
GDEVMVLIPQLHRDKTVWGDDVEEFRPERFENPSAIPQHAFKPFGNGQRASIGQQFALHEATLVLGMMLK
HFDFEDHTNYELDIKELQTLKPKGFVVKAKSKKIPLGGIPSPSTEQSAKKVRKKAENAHNTPLLVLYGSN
MGTAEGTARDLADIAMSKGFAPQVATLDSHAGNLPREGAVLIVTASYNGHPPDNAKQFVDWLDQASADEV
KGVRYSVFGCGDKNWATTYQKVPAFIDETLAAKGAENIADRGEADASDDFEGTYEEWREHMWSDVAAYFN
LDIENSEDNKSTLSLQFVDSAADMPLAKMHGAFSTNVVASKELQQPGSARSTRHLEIELPKEASYQEGDH
LGVIPRNYEGIVNRVTARFGLDASQQIRLEAEEEKLAHLPLAKTVSVEELLQYVELQDPVTRTQLRAMAA
KTVCPPHKVELEALLEKQAYKEQVLAKRLTMLELLEKYPACEMKFSEFIALLPSIRPRYYSISSSPRVDE
KQASITVSVVSGEAWSGYGEYKGIASNYLAELQEGDTITCFISTPQSEFTLPKDPETPLIMVGPGTGVAP
FRGFVQARKQLKEQGQSLGEAHLYFGCRSPHEDYLYQEELENAQSEGIITLHTAFSRMPNQPKTYVQHVM
EQDGKKLIELLDQGAHFYICGDGSQMAPAVEATLMKSYADVHQVSEADARLWLQQLEEKGRYAKDVWAGL
EHHHHHH

Nucleotide and amino acid sequences of P411-PYS-5143:
ATGACAATTAAAGAAATGCCTCAGCCAAAAACGTTTGGAGAGCTTAAAAATTTACCGTTATTAAACACAG
ATAAACCGGTTCAAGCTTTGATGAAAATTGCGGATGAATTAGGAGAAATCTTTAAATTCGAGGCGCCTGG
TCGTGTAACGCGCTACTTATCAAGTCAGCGTCTAATTAAAGAAGCATGCGATGAATCACGCTTTGATAAA
AACTTAAGTCAAGGTGAGAAATTTCTGCGTGATTTTCTTGGAGACGGGTTAGCCACAAGCTGGACGCATG
AAAAAAATTGGAAAAAAGCGCATAATATCTTACTTCCAAGCTTTAGTCAGCAGGCAATGAAAGGCTATCA
TGCGATGATGGTCGATATCGCCGTGCAGCTTGTTCAAAAGTGGGAGCGTCTAAATGCAGATGAGCATATT
GAAGTATCGGAAGACATGACACGTTTAACGCTTGATACAATTGGTCTTTGCGGCTTTAACTATCGCTTTA
ACAGCTTTTACCGAGATCAGCCTCATCCATTTATTATAAGTCTGGTCCGTGCACTGGATGAAGTAATGAA
CAAGCTGCAGCGAGCAAATCCAGACGACCCAGCTTATGATGAAAACAAGCGCCAGTTTCAAGAAGATATC
AAGGTGATGAACGACCTAGTAGATAAAATTATTGCAGATCGCAAAGCAAGGGGTGAACAAAGCGATGATT
TATTAACGCAGATGCTAAACGGAAAAGATCCAGAAACGGGTGAGCCGCTTGATGACGGGAACATTCGCTA
TCAAATTATTACATTCTTATATGCGGGAGTTGAAGGTACAAGTGGTCTTTTATCATTTGCGCTGTATTTC
TTAGTGAAAAATCCACATGTATTACAAAAAGTAGCAGAAGAAGCAGCACGAGTTCTAGTAGATCCTGTTC
CAAGCTACAAACAAGTCAAACAGCTTAAATATGTCGGCATGGTCTTAAACGAAGCGCTGCGCTTATGGCC
AATTGTTCCTGCGTTTTCCCTATATGCAAAAGAAGATACGGTGCTTGGAGGAGAATATCCTTTAGAAAAA
GGCGACGAAGTAATGGTTCTGATTCCTCAGCTTCACCGTGATAAAACAGTTTGGGGAGACGATGTGGAGG
AGTTCCGTCCAGAGCGTTTTGAAAATCCAAGTGCGATTCCGCAGCATGCGTTTAAACCGTTTGGAAACGG
TCAGCGTGCGTCTATCGGTCAGCAGTTCGCTCTTCATGAAGCAACGCTGGTACTTGGTATGATGCTAAAA
CACTTTGACTTTGAAGATCATACAAACTACGAGCTCGATATTAAAGAACTGCTGACGTTAAAACCTAAAG
GCTTTGTGGTAAAAGCAAAATCGAAAAAAATTCCGCTTGGCGGTATTCCTTCACCTAGCACTGAACAGTC
TGCTAAAAAAGTACGCAAAAAGGCAGAAAACGCTCATAATACGCCGCTGCTTGTGCTATACGGTTCAAAT

ATGGGTACCGCTGAAGGAACGGCGCGTGATTTAGCAGATATTGCAATGAGCAAAGGATTTGCACCGCAGG
TCGCAACGCTTGATTCACACGCCGGAAATCTTCCGCGCGAAGGAGCTGTATTAATTGTAACGGCGTCTTA
TAACGGTCATCCGCCTGATAACGCAAAGCAATTTGTCGACTGGTTAGACCAAGCGTCTGCTGATGAAGTA
AAAGGCGTTCGCTACTCCGTATTTGGATGCGGCGATAAAAACTGGGCTACTACGTATCAAAAAGTGCCTG
CTTTTATCGATGAAACGCTTGCCGCTAAAGGGGCAGAAAACATCGCTGACCGCGGTGAAGCAGATGCAAG
CGACGACTTTGAAGGCACATATGAAGAATGGCGTGAACATATGTGGAGTGACGTAGCAGCCTACTTTAAC
CTCGACATTGAAAACAGTGAAGATAATAAATCTACTCTTTCACTTCAATTTGTCGACAGCGCCGCGGATA
TGCCGCTTGCGAAAATGCACGGTGCGTTTTCAACGAACGTCGTAGCAAGCAAAGAACTTCAACAGCCAGG
CAGTGCACGAAGCACGCGACATCTTGAAATTGAACTTCCAAAAGAAGCTTCTTATCAAGAAGGAGATCAT
TTAGGTGTTATTCCTCGCAACTATGAAGGAATAGTAAACCGTGTAACAGCAAGGTTCGGCCTAGATGCAT
CACAGCAAATCCGTCTGGAAGCAGAAGAAGAAAAATTAGCTCATTTGCCACTCGCTAAAACAGTATCCGT
AGAAGAGCTTCTGCAATACGTGGAGCTTCAAGATCCTGTTACGCGCACGCAGCTTCGCGCAATGGCTGCT
AAAACGGTCTGCCCGCCGCATAAAGTAGAGCTTGAAGCCTTGCTTGAAAAGCAAGCCTACAAAGAACAAG
TGCTGGCAAAACGTTTAACAATGCTTGAACTGCTTGAAAAATACCCGGCGTGTGAAATGAAATTCAGCGA
ATTTATCGCCCTTCTGCCAAGCATACGCCCGCGCTATTACTCGATTTCTTCATCACCTCGTGTCGATGAA
AAACAAGCAAGCATCACGGTCAGCGTTGTCTCAGGAGAAGCGTGGAGCGGATATGGAGAATATAAAGGAA
TTGCGTCGAACTATCTTGCCGAGCTGCAAGAAGGAGATACGATTACGTGCTTTATTTCCACACCGCAGTC
AGAATTTACGCTGCCAAAAGACCCTGAAACGCCGCTTATCATGGTCGGACCGGGAACAGGCGTCGCGCCG
TTTAGAGGCTTTGTGCAGGCGCGCAAACAGCTAAAAGAACAAGGACAGTCACTTGGAGAAGCACATTTAT
ACTTCGGCTGCCGTTCACCTCATGAAGACTATCTGTATCAAGAAGAGCTTGAAAACGCCCAAAGCGAAGG
CATCATTACGCTTCATACCGCTTTTTCTCGCATGCCAAATCAGCCGAAAACATACGTTCAGCACGTAATG
GAACAAGACGGCAAGAAATTGATTGAACTTCTTGATCAAGGAGCGCACTTCTATATTTGCGGAGACGGAA
GCCAAATGGCACCTGCCGTTGAAGCAACGCTTATGAAAAGCTATGCTGACGTTCACCAAGTGAGTGAAGC
AGACGCTCGCTTATGGCTGCAGCAGCTAGAAGAAAAAGGCCGATACGCAAAAGACGTGTGGGCTGGGCTC
GAGCACCACCACCACCACCACTGA

MTIKEMPQPKTFGELKNLPLLNTDKPVQALMKIADELGEIFKFEAPGRVTRYLSSQRLIKEACDESRFDK
NLSQGEKFLRDFLGDGLATSWTHEKNWKKAHNILLPSFSQQAMKGYHAMMVDIAVQLVQKWERLNADEHI
EVSEDMTRLTLDTIGLCGFNYRFNSFYRDQPHPFIISLVRALDEVMNKLQRANPDDPAYDENKRQFQEDI
KVMNDLVDKIIADRKARGEQSDDLLTQMLNGKDPETGEPLDDGNIRYQIITFLYAGVEGTSGLLSFALYF
LVKNPHVLQKVAEEAARVLVDPVPSYKQVKQLKYVGMVLNEALRLWPIVPAFSLYAKEDTVLGGEYPLEK
GDEVMVLIPQLHRDKTVWGDDVEEFRPERFENPSAIPQHAFKPFGNGQRASIGQQFALHEATLVLGMMLK
HFDFEDHTNYELDIKELLTLKPKGFVVKAKSKKIPLGGIPSPSTEQSAKKVRKKAENAHNTPLLVLYGSN
MGTAEGTARDLADIAMSKGFAPQVATLDSHAGNLPREGAVLIVTASYNGHPPDNAKQFVDWLDQASADEV
KGVRYSVFGCGDKNWATTYQKVPAFIDETLAAKGAENIADRGEADASDDFEGTYEEWREHMWSDVAAYFN
LDIENSEDNKSTLSLQFVDSAADMPLAKMHGAFSTNVVASKELQQPGSARSTRHLEIELPKEASYQEGDH
LGVIPRNYEGIVNRVTARFGLDASQQIRLEAEEEKLAHLPLAKTVSVEELLQYVELQDPVTRTQLRAMAA
KTVCPPHKVELEALLEKQAYKEQVLAKRLTMLELLEKYPACEMKFSEFIALLPSIRPRYYSISSSPRVDE
KQASITVSVVSGEAWSGYGEYKGIASNYLAELQEGDTITCFISTPQSEFTLPKDPETPLIMVGPGTGVAP
FRGFVQARKQLKEQGQSLGEAHLYFGCRSPHEDYLYQEELENAQSEGIITLHTAFSRMPNQPKTYVQHVM
EQDGKKLIELLDQGAHFYICGDGSQMAPAVEATLMKSYADVHQVSEADARLWLQQLEEKGRYAKDVWAGL
EHHHHHH

Nucleotide and amino acid sequences of P411-PYS-5144:
ATGACAATTAAAGAAATGCCTCAGCCAAAAACGTTTGGAGAGCTTAAAAATTTACCGTTATTAAACACAG
ATAAACCGGTTCAAGCTTTGATGAAAATTGCGGATGAATTAGGAGAAATCTTTAAATTCGAGGCGCCTGG
TCGTGTAACGCGCTACTTATCAAGTCAGCGTCTAATTAAAGAAGCATGCGATGAATCACGCTTTGATAAA
AACTTAAGTCAAGGTGAGAAATTTCTGCGTGATTTTCTTGGAGACGGGTTAGCCACAAGCTGGACGCATG
AAAAAAATTGGGAAAAAAGCGCATAATATCTTACTTCCAAGCTTTAGTCAGCAGGCAATGAAAGGCTATC
ATGCGATGATGGTCGATATCGCCGTGCAGCTTGTTCAAAAGTGGGAGCGTCTAAATGCAGATGAGCATAT
TGAAGTATCGGAAGACATGACACGTTTAACGCTTGATACAATTGGTCTTTGCGGCTTTAACTATCGCTTT

AACAGCTTTTACCGAGATCAGCCTCATCCATTTATTATAAGTCTGGTCCGTGCACTGGATGAAGTAATGA
ACAAGCTGCAGCGAGCAAATCCAGACGACCCAGCTTATGATGAAAACAAGCGCCAGTTTCAAGAAGATAT
CAAGGTGATGAACGACCTAGTAGATAAAATTATTGCAGATCGCAAAGCAAGGGGTGAACAAAGCGATGAT
TTATTAACGCAGATGCTAAACGGAAAAGATCCAGAAACGGGTGAGCCGCTTGATGACGGGAACATTCGCT
ATCAAATTATTACATTCTTATATGCGGGAGTTGAAGGTACAAGTGGTCTTTTATCATTTGCGCTGTATTT
CTTAGTGAAAAATCCACATGTATTACAAAAAGTAGCAGAAGAAGCAGCACGAGTTCTAGTAGATCCTGTT
CCAAGCTACAAACAAGTCAAACAGCTTAAATATGTCGGCATGGTCTTAAACGAAGCGCTGCGCTTATGGC
CAATTGTTCCTCAGTTTTCCCTATATGCAAAAGAAGATACGGTGCTTGGAGGAGAATATCCTTTAGAAAA
AGGCGACGAAGTAATGGTTCTGATTCCTCAGCTTCACCGTGATAAAACAGTTTGGGGAGACGATGTGGAG
GAGTTCCGTCCAGAGCGTTTTGAAAATCCAAGTGCGATTCCGCAGCATGCGTTTAAACCGTTTGGAAACG
GTCAGCGTGCGTCTATCGGTCAGCAGTTCGCTCTTCATGAAGCAACGCTGGTACTTGGTATGATGCTAAA
ACACTTTGACTTTGAAGATCATACAAACTACGAGCTCGATATTAAAGAACTGCTGACGTTAAAACCTAAA
GGCTTTGTGGTAAAAGCAAAATCGAAAAAAATTCCGCTTGGCGGTATTCCTTCACCTAGCACTGAACAGT
CTGCTAAAAAAGTACGCAAAAAGGCAGAAAACGCTCATAATACGCCGCTGCTTGTGCTATACGGTTCAAA
TATGGGTACCGCTGAAGGAACGGCGCGTGATTTAGCAGATATTGCAATGAGCAAAGGATTTGCACCGCAG
GTCGCAACGCTTGATTCACACGCCGGAAATCTTCCGCGCGAAGGAGCTGTATTAATTGTAACGGCGTCTT
ATAACGGTCATCCGCCTGATAACGCAAAGCAATTTGTCGACTGGTTAGACCAAGCGTCTGCTGATGAAGT
AAAAGGCGTTCGCTACTCCGTATTTGGATGCGGCGATAAAAACTGGGCTACTACGTATCAAAAAGTGCCT
GCTTTTATCGATGAAACGCTTGCCGCTAAAGGGGCAGAAAACATCGCTGACCGCGGTGAAGCAGATGCAA
GCGACGACTTTGAAGGCACATATGAAGAATGGCGTGAACATATGTGGAGTGACGTAGCAGCCTACTTTAA
CCTCGACATTGAAAACAGTGAAGATAATAAATCTACTCTTTCACTTCAATTTGTCGACAGCGCCGCGGAT
ATGCCGCTTGCGAAAATGCACGGTGCGTTTTCAACGAACGTCGTAGCAAGCAAAGAACTTCAACAGCCAG
GCAGTGCACGAAGCACGCGACATCTTGAAATTGAACTTCCAAAAGAAGCTTCTTATCAAGAAGGAGATCA
TTTAGGTGTTATTCCTCGCAACTATGAAGGAATAGTAAACCGTGTAACAGCAAGGTTCGGCCTAGATGCA
TCACAGCAAATCCGTCTGGAAGCAGAAGAAGAAAAATTAGCTCATTTGCCACTCGCTAAAACAGTATCCG
TAGAAGAGCTTCTGCAATACGTGGAGCTTCAAGATCCTGTTACGCGCACGCAGCTTCGCGCAATGGCTGC
TAAAACGGTCTGCCCGCCGCATAAAGTAGAGCTTGAAGCCTTGCTTGAAAAGCAAGCCTACAAAGAACAA
GTGCTGGCAAAACGTTTAACAATGCTTGAACTGCTTGAAAAATACCCGGCGTGTGAAATGAAATTCAGCG
AATTTATCGCCCTTCTGCCAAGCATACGCCCGCGCTATTACTCGATTTCTTCATCACCTCGTGTCGATGA
AAAACAAGCAAGCATCACGGTCAGCGTTGTCTCAGGAGAAGCGTGGAGCGGATATGGAGAATATAAAGGA
ATTGCGTCGAACTATCTTGCCGAGCTGCAAGAAGGAGATACGATTACGTGCTTTATTTCCACACCGCAGT
CAGAATTTACGCTGCCAAAAGACCCTGAAACGCCGCTTATCATGGTCGGACCGGGAACAGGCGTCGCGCC
GTTTAGAGGCTTTGTGCAGGCGCGCAAACAGCTAAAAGAACAAGGACAGTCACTTGGAGAAGCACATTTA
TACTTCGGCTGCCGTTCACCTCATGAAGACTATCTGTATCAAGAAGAGCTTGAAAACGCCCAAAGCGAAG
GCATCATTACGCTTCATACCGCTTTTTCTCGCATGCCAAATCAGCCGAAAACATACGTTCAGCACGTAAT
GGAACAAGACGGCAAGAAATTGATTGAACTTCTTGATCAAGGAGCGCACTTCTATATTTGCGGAGACGGA
AGCCAAATGGCACCTGCCGTTGAAGCAACGCTTATGAAAAGCTATGCTGACGTTCACCAAGTGAGTGAAG
CAGACGCTCGCTTATGGCTGCAGCAGCTAGAAGAAAAAGGCCGATACGCAAAAGACGTGTGGGCTGGGCT
CGAGCACCACCACCACCACCACTGA

MTIKEMPQPKTFGELKNLPLLNTDKPVQALMKIADELGEIFKFEAPGRVTRYLSSQRLIKEACDESRFDK
NLSQGEKFLRDFLGDGLATSWTHEKNWKKAHNILLPSFSQQAMKGYHAMMVDIAVQLVQKWERLNADEHI
EVSEDMTRLTLDTIGLCGFNYRFNSFYRDQPHPFIISLVRALDEVMNKLQRANPDDPAYDENKRQFQEDI
KVMNDLVDKIIADRKARGEQSDDLLTQMLNGKDPETGEPLDDGNIRYQIITFLYAGVEGTSGLLSFALYF
LVKNPHVLQKVAEEAARVLVDPVPSYKQVKQLKYVGMVLNEALRLWPIVPQFSLYAKEDTVLGGEYPLEK
GDEVMVLIPQLHRDKTVWGDDVEEFRPERFENPSAIPQHAFKPFGNGQRASIGQQFALHEATLVLGMMLK
HFDFEDHTNYELDIKELLTLKPKGFVVKAKSKKIPLGGIPSPSTEQSAKKVRKKAENAHNTPLLVLYGSN
MGTAEGTARDLADIAMSKGFAPQVATLDSHAGNLPREGAVLIVTASYNGHPPDNAKQFVDWLDQASADEV
KGVRYSVFGCGDKNWATTYQKVPAFIDETLAAKGAENIADRGEADASDDFEGTYEEWREHMWSDVAAYFN
LDIENSEDNKSTLSLQFVDSAADMPLAKMHGAFSTNVVASKELQQPGSARSTRHLEIELPKEASYQEGDH
LGVIPRNYEGIVNRVTARFGLDASQQIRLEAEEEKLAHLPLAKTVSVEELLQYVELQDPVTRTQLRAMAA
KTVCPPHKVELEALLEKQAYKEQVLAKRLTMLELLEKYPACEMKFSEFIALLPSIRPRYYSISSSPRVDE

KQASITVSVVSGEAWSGYGEYKGIASNYLAELQEGDTITCFISTPQSEFTLPKDPETPLIMVGPGTGVAP
FRGFVQARKQLKEQGQSLGEAHLYFGCRSPHEDYLYQEELENAQSEGIITLHTAFSRMPNQPKTYVQHVM
EQDGKKLIELLDQGAHFYICGDGSQMAPAVEATLMKSYADVHQVSEADARLWLQQLEEKGRYAKDVWAGL
EHHHHHH

Nucleotide and amino acid sequences of P411-PYS-5145:

ATGACAATTAAAGAAATGCCTCAGCCAAAAACGTTTGGAGAGCTTAAAAATTTACCGTTATTAAACACAG
ATAAACCGGTTCAAGCTTTGATGAAAATTGCGGATGAATTAGGAGAAATCTTTAAATTCGAGGCGCCTGG
TCGTGTAACGCGCTACTTATCAAGTCAGCGTCTAATTAAAGAAGCATGCGATGAATCACGCTTTGATAAA
AACTTAAGTCAAGGTGAGAAATTTCTGCGTGATTTTCTTGGAGACGGGTTAGCCACAAGCTGGACGCATG
AAAAAAATTGGGAAAAAAGCGCATAATATCTTACTTCCAAGCTTTAGTCAGCAGGCAATGAAAGGCTATC
ATGCGGTGATGGTCGATATCGCCGTGCAGCTTGTTCAAAAGTGGGAGCGTCTAAATGCAGATGAGCATAT
TGAAGTATCGGAAGACATGACACGTTTAACGCTTGATACAATTGGTCTTTGCGGCTTTAACTATCGCTTT
AACAGCTTTTACCGAGATCAGCCTCATCCATTTATTATAAGTCTGGTCCGTGCACTGGATGAAGTAATGA
ACAAGCTGCAGCGAGCAAATCCAGACGACCCAGCTTATGATGAAAACAAGCGCCAGTTTCAAGAAGATAT
CAAGGTGATGAACGACCTAGTAGATAAAATTATTGCAGATCGCAAAGCAAGGGGTGAACAAAGCGATGAT
TTATTAACGCAGATGCTAAACGGAAAAGATCCAGAAACGGGTGAGCCGCTTGATGACGGGAACATTCGCT
ATCAAATTATTACATTCTTATATGCGGGAGTTGAAGGTACAAGTGGTCTTTTATCATTTGCGCTGTATTT
CTTAGTGAAAAATCCACATGTATTACAAAAAGTAGCAGAAGAAGCAGCACGAGTTCTAGTAGATCCTGTT
CCAAGCTACAAACAAGTCAAACAGCTTAAATATGTCGGCATGGTCTTAAACGAAGCGCTGCGCTTATGGC
CAATTGTTCCTCAGTTTTCCCTATATGCAAAAGAAGATACGGTGCTTGGAGGAGAATATCCTTTAGAAAA
AGGCGACGAAGTAATGGTTCTGATTCCTCAGCTTCACCGTGATAAAACAGTTTGGGGAGACGATGTGGAG
GAGTTCCGTCCAGAGCGTTTTGAAAATCCAAGTGCGATTCCGCAGCATGCGTTTAAACCGTTTGGAAACG
GTCAGCGTGCGTCTATCGGTCAGCAGTTCGCTCTTCATGAAGCAACGCTGGTACTTGGTATGATGCTAAA
ACACTTTGACTTTGAAGATCATACAAACTACGAGCTCGATATTAAAGAACTGCTGACGTTAAAACCTAAA
GGCTTTGTGGTAAAAGCAAAATCGAAAAAAATTCCGCTTGGCGGTATTCCTTCACCTAGCACTGAACAGT
CTGCTAAAAAAGTACGCAAAAAGGCAGAAAACGCTCATAATACGCCGCTGCTTGTGCTATACGGTTCAAA
TATGGGTACCGCTGAAGGAACGGCGCGTGATTTAGCAGATATTGCAATGAGCAAAGGATTTGCACCGCAG
GTCGCAACGCTTGATTCACACGCCGGAAATCTTCCGCGCGAAGGAGCTGTATTAATTGTAACGGCGTCTT
ATAACGGTCATCCGCCTGATAACGCAAAGCAATTTGTCGACTGGTTAGACCAAGCGTCTGCTGATGAAGT
AAAAGGCGTTCGCTACTCCGTATTTGGATGCGGCGATAAAAACTGGGCTACTACGTATCAAAAAGTGCCT
GCTTTTATCGATGAAACGCTTGCCGCTAAAGGGGCAGAAAACATCGCTGACCGCGGTGAAGCAGATGCAA
GCGACGACTTTGAAGGCACATATGAAGAATGGCGTGAACATATGTGGAGTGACGTAGCAGCCTACTTTAA
CCTCGACATTGAAAACAGTGAAGATAATAAATCTACTCTTTCACTTCAATTTGTCGACAGCGCCGCGGAT
ATGCCGCTTGCGAAAATGCACGGTGCGTTTTCAACGAACGTCGTAGCAAGCAAAGAACTTCAACAGCCAG
GCAGTGCACGAAGCACGCGACATCTTGAAATTGAACTTCCAAAAGAAGCTTCTTATCAAGAAGGAGATCA
TTTAGGTGTTATTCCTCGCAACTATGAAGGAATAGTAAACCGTGTAACAGCAAGGTTCGGCCTAGATGCA
TCACAGCAAATCCGTCTGGAAGCAGAAGAAGAAAAATTAGCTCATTTGCCACTCGCTAAAACAGTATCCG
TAGAAGAGCTTCTGCAATACGTGGAGCTTCAAGATCCTGTTACGCGCACGCAGCTTCGCGCAATGGCTGC
TAAAACGGTCTGCCCGCCGCATAAAGTAGAGCTTGAAGCCTTGCTTGAAAAGCAAGCCTACAAAGAACAA
GTGCTGGCAAAACGTTTAACAATGCTTGAACTGCTTGAAAAATACCCGGCGTGTGAAATGAAATTCAGCG
AATTTATCGCCCTTCTGCCAAGCATACGCCCGCGCTATTACTCGATTTCTTCATCACCTCGTGTCGATGA
AAAACAAGCAAGCATCACGGTCAGCGTTGTCTCAGGAGAAGCGTGGAGCGGATATGGAGAATATAAAGGA
ATTGCGTCGAACTATCTTGCCGAGCTGCAAGAAGGAGATACGATTACGTGCTTTATTTCCACACCGCAGT
CAGAATTTACGCTGCCAAAAGACCCTGAAACGCCGCTTATCATGGTCGGACCGGGAACAGGCGTCGCGCC
GTTTAGAGGCTTTGTGCAGGCGCGCAAACAGCTAAAAGAACAAGGACAGTCACTTGGAGAAGCACATTTA
TACTTCGGCTGCCGTTCACCTCATGAAGACTATCTGTATCAAGAAGAGCTTGAAAACGCCCAAAGCGAAG
GCATCATTACGCTTCATACCGCTTTTTCTCGCATGCCAAATCAGCCGAAAACATACGTTCAGCACGTAAT
GGAACAAGACGGCAAGAAATTGATTGAACTTCTTGATCAAGGAGCGCACTTCTATATTTGCGGAGACGGA
AGCCAAATGGCACCTGCCGTTGAAGCAACGCTTATGAAAAGCTATGCTGACGTTCACCAAGTGAGTGAAG
CAGACGCTCGCTTATGGCTGCAGCAGCTAGAAGAAAAAGGCCGATACGCAAAAGACGTGTGGGCTGGGCT

CGAGCACCACCACCACCACCACTGA

MTIKEMPQPKTFGELKNLPLLNTDKPVQALMKIADELGEIFKFEAPGRVTRYLSSQRLIKEACDESRFDK
NLSQGEKFLRDFLGDGLATSWTHEKNWKKAHNILLPSFSQQAMKGYHAVMVDIAVQLVQKWERLNADEHI
EVSEDMTRLTLDTIGLCGFNYRFNSFYRDQPHPFIISLVRALDEVMNKLQRANPDDPAYDENKRQFQEDI
KVMNDLVDKIIADRKARGEQSDDLLTQMLNGKDPETGEPLDDGNIRYQIITFLYAGVEGTSGLLSFALYF
LVKNPHVLQKVAEEAARVLVDPVPSYKQVKQLKYVGMVLNEALRLWPIVPQFSLYAKEDTVLGGEYPLEK
GDEVMVLIPQLHRDKTVWGDDVEEFRPERFENPSAIPQHAFKPFGNGQRASIGQQFALHEATLVLGMMLK
HFDFEDHTNYELDIKELLTLKPKGFVVKAKSKKIPLGGIPSPSTEQSAKKVRKKAENAHNTPLLVLYGSN
MGTAEGTARDLADIAMSKGFAPQVATLDSHAGNLPREGAVLIVTASYNGHPPDNAKQFVDWLDQASADEV
KGVRYSVFGCGDKNWATTYQKVPAFIDETLAAKGAENIADRGEADASDDFEGTYEEWREHMWSDVAAYFN
LDIENSEDNKSTLSLQFVDSAADMPLAKMHGAFSTNVVASKELQQPGSARSTRHLEIELPKEASYQEGDH
LGVIPRNYEGIVNRVTARFGLDASQQIRLEAEEEKLAHLPLAKTVSVEELLQYVELQDPVTRTQLRAMAA
KTVCPPHKVELEALLEKQAYKEQVLAKRLTMLELLEKYPACEMKFSEFIALLPSIRPRYYSISSSPRVDE
KQASITVSVVSGEAWSGYGEYKGIASNYLAELQEGDTITCFISTPQSEFTLPKDPETPLIMVGPGTGVAP
FRGFVQARKQLKEQGQSLGEAHLYFGCRSPHEDYLYQEELENAQSEGIITLHTAFSRMPNQPKTYVQHVM
EQDGKKLIELLDQGAHFYICGDGSQMAPAVEATLMKSYADVHQVSEADARLWLQQLEEKGRYAKDVWAGL
EHHHHHH

Nucleotide and amino acid sequences of P411-PYS-5146:

ATGACAATTAAAGAAATGCCTCAGCCAAAAACGTTTGGAGAGCTTAAAAATTTACCGTTATTAAACACAG
ATAAACCGGTTCAAGCTTTGATGAAAATTGCGGATGAATTAGGAGAAATCTTTAAATTCGAGGCGCCTGG
TCGTGTAACGCGCTACTTATCAAGTCAGCGTCTAATTAAAGAAGCATGCGATGAATCACGCTTTGATAAA
AACTTAAGTCAAGGTGAGAAATGTCTGCGTGATTTTCTTGGAGACGGGTTAGCCACAAGCTGGACGCATG
AAAAAAATTGGGAAAAAAGCGCATAATATCTTACTTCCAAGCTTTAGTCAGCAGGCAATGAAAGGCTATC
ATGCGGTGATGGTCGATATCGCCGTGCAGCTTGTTCAAAAGTGGGAGCGTCTAAATGCAGATGAGCATAT
TGAAGTATCGGAAGACATGACACGTTTAACGCTTGATACAATTGGTCTTTGCGGCTTTAACTATCGCTTT
AACAGCTTTTACCGAGATCAGCCTCATCCATTTATTATAAGTCTGGTCCGTGCACTGGATGAAGTAATGA
ACAAGCTGCAGCGAGCAAATCCAGACGACCCAGCTTATGATGAAAACAAGCGCCAGTTTCAAGAAGATAT
CAAGGTGATGAACGACCTAGTAGATAAAATTATTGCAGATCGCAAAGCAAGGGGTGAACAAAGCGATGAT
TTATTAACGCAGATGCTAAACGGAAAAGATCCAGAAACGGGTGAGCCGCTTGATGACGGGAACATTCGCT
ATCAAATTATTACATTCTTATATGCGGGAGTTGAAGGTACAAGTGGTCTTTTATCATTTGCGCTGTATTT
CTTAGTGAAAAATCCACATGTATTACAAAAAGTAGCAGAAGAAGCAGCACGAGTTCTAGTAGATCCTGTT
CCAAGCTACAAACAAGTCAAACAGCTTAAATATGTCGGCATGGTCTTAAACGAAGCGCTGCGCTTATGGC
CAATTGTTCCTCAGTTTTCCCTATATGCAAAAGAAGATACGGTGCTTGGAGGAGAATATCCTTTAGAAAA
AGGCGACGAAGTAATGGTTCTGATTCCTCAGCTTCACCGTGATAAAACAGTTTGGGGAGACGATGTGGAG
GAGTTCCGTCCAGAGCGTTTTGAAAATCCAAGTGCGATTCCGCAGCATGCGTTTAAACCGTTTGGAAACG
GTCAGCGTGCGTCTATCGGTCAGCAGTTCGCTCTTCATGAAGCAACGCTGGTACTTGGTATGATGCTAAA
ACACTTTGACTTTGAAGATCATACAAACTACGAGCTCGATATTAAAGAACTGCTGACGTTAAAACCTAAA
GGCTTTGTGGTAAAAGCAAAATCGAAAAAAATTCCGCTTGGCGGTATTCCTTCACCTAGCACTGAACAGT
CTGCTAAAAAAGTACGCAAAAAGGCAGAAAACGCTCATAATACGCCGCTGCTTGTGCTATACGGTTCAAA
TATGGGTACCGCTGAAGGAACGGCGCGTGATTTAGCAGATATTGCAATGAGCAAAGGATTTGCACCGCAG
GTCGCAACGCTTGATTCACACGCCGGAAATCTTCCGCGCGAAGGAGCTGTATTAATTGTAACGGCGTCTT
ATAACGGTCATCCGCCTGATAACGCAAAGCAATTTGTCGACTGGTTAGACCAAGCGTCTGCTGATGAAGT
AAAAGGCGTTCGCTACTCCGTATTTGGATGCGGCGATAAAAACTGGGCTACTACGTATCAAAAAGTGCCT
GCTTTTATCGATGAAACGCTTGCCGCTAAAGGGGCAGAAAACATCGCTGACCGCGGTGAAGCAGATGCAA
GCGACGACTTTGAAGGCACATATGAAGAATGGCGTGAACATATGTGGAGTGACGTAGCAGCCTACTTTAA
CCTCGACATTGAAAACAGTGAAGATAATAAATCTACTCTTTCACTTCAATTTGTCGACAGCGCCGCGGAT
ATGCCGCTTGCGAAAATGCACGGTGCGTTTTCAACGAACGTCGTAGCAAGCAAAGAACTTCAACAGCCAG
GCAGTGCACGAAGCACGCGACATCTTGAAATTGAACTTCCAAAAGAAGCTTCTTATCAAGAAGGAGATCA
TTTAGGTGTTATTCCTCGCAACTATGAAGGAATAGTAAACCGTGTAACAGCAAGGTTCGGCCTAGATGCA

TCACAGCAAATCCGTCTGGAAGCAGAAGAAGAAAAATTAGCTCATTTGCCACTCGCTAAAACAGTATCCG
TAGAAGAGCTTCTGCAATACGTGGAGCTTCAAGATCCTGTTACGCGCACGCAGCTTCGCGCAATGGCTGC
TAAAACGGTCTGCCCGCCGCATAAAGTAGAGCTTGAAGCCTTGCTTGAAAAGCAAGCCTACAAAGAACAA
GTGCTGGCAAAACGTTTAACAATGCTTGAACTGCTTGAAAAATACCCGGCGTGTGAAATGAAATTCAGCG
AATTTATCGCCCTTCTGCCAAGCATACGCCCGCGCTATTACTCGATTTCTTCATCACCTCGTGTCGATGA
AAAACAAGCAAGCATCACGGTCAGCGTTGTCTCAGGAGAAGCGTGGAGCGGATATGGAGAATATAAAGGA
ATTGCGTCGAACTATCTTGCCGAGCTGCAAGAAGGAGATACGATTACGTGCTTTATTTCCACACCGCAGT
CAGAATTTACGCTGCCAAAAGACCCTGAAACGCCGCTTATCATGGTCGGACCGGGAACAGGCGTCGCGCC
GTTTAGAGGCTTTGTGCAGGCGCGCAAACAGCTAAAAGAACAAGGACAGTCACTTGGAGAAGCACATTTA
TACTTCGGCTGCCGTTCACCTCATGAAGACTATCTGTATCAAGAAGAGCTTGAAAACGCCCAAAGCGAAG
GCATCATTACGCTTCATACCGCTTTTTCTCGCATGCCAAATCAGCCGAAAACATACGTTCAGCACGTAAT
GGAACAAGACGGCAAGAAATTGATTGAACTTCTTGATCAAGGAGCGCACTTCTATATTTGCGGAGACGGA
AGCCAAATGGCACCTGCCGTTGAAGCAACGCTTATGAAAAGCTATGCTGACGTTCACCAAGTGAGTGAAG
CAGACGCTCGCTTATGGCTGCAGCAGCTAGAAGAAAAAGGCCGATACGCAAAAGACGTGTGGGCTGGGCT
CGAGCACCACCACCACCACCACTGA

MTIKEMPQPKTFGELKNLPLLNTDKPVQALMKIADELGEIFKFEAPGRVTRYLSSQRLIKEACDESRFDK
NLSQGEKCLRDFLGDGLATSWTHEKNWKKAHNILLPSFSQQAMKGYHAVMVDIAVQLVQKWERLNADEHI
EVSEDMTRLTLDTIGLCGFNYRFNSFYRDQPHPFIISLVRALDEVMNKLQRANPDDPAYDENKRQFQEDI
KVMNDLVDKIIADRKARGEQSDDLLTQMLNGKDPETGEPLDDGNIRYQIITFLYAGVEGTSGLLSFALYF
LVKNPHVLQKVAEEAARVLVDPVPSYKQVKQLKYVGMVLNEALRLWPIVPQFSLYAKEDTVLGGEYPLEK
GDEVMVLIPQLHRDKTVWGDDVEEFRPERFENPSAIPQHAFKPFGNGQRASIGQQFALHEATLVLGMMLK
HFDFEDHTNYELDIKELLTLKPKGFVVKAKSKKIPLGGIPSPSTEQSAKKVRKKAENAHNTPLLVLYGSN
MGTAEGTARDLADIAMSKGFAPQVATLDSHAGNLPREGAVLIVTASYNGHPPDNAKQFVDWLDQASADEV
KGVRYSVFGCGDKNWATTYQKVPAFIDETLAAKGAENIADRGEADASDDFEGTYEEWREHMWSDVAAYFN
LDIENSEDNKSTLSLQFVDSAADMPLAKMHGAFSTNVVASKELQQPGSARSTRHLEIELPKEASYQEGDH
LGVIPRNYEGIVNRVTARFGLDASQQIRLEAEEEKLAHLPLAKTVSVEELLQYVELQDPVTRTQLRAMAA
KTVCPPHKVELEALLEKQAYKEQVLAKRLTMLELLEKYPACEMKFSEFIALLPSIRPRYYSISSSPRVDE
KQASITVSVVSGEAWSGYGEYKGIASNYLAELQEGDTITCFISTPQSEFTLPKDPETPLIMVGPGTGVAP
FRGFVQARKQLKEQGQSLGEAHLYFGCRSPHEDYLYQEELENAQSEGIITLHTAFSRMPNQPKTYVQHVM
EQDGKKLIELLDQGAHFYICGDGSQMAPAVEATLMKSYADVHQVSEADARLWLQQLEEKGRYAKDVWAGL
EHHHHHH

Nucleotide and amino acid sequences of P411-PYS-5147:
ATGACAATTAAAGAAATGCCTCAGCCAAAAACGTTTGGAGAGCTTAAAAATTTACCGTTATTAAACACAG
ATAAACCGGTTCAAGCTTTGATGAAAATTGCGGATGAATTAGGAGAAATCTTTAAATTCGAGGCGCCTGG
TCGTGTAACGCGCTACTTATCAAGTCAGCGTCTAATTAAAGAAGCATGCGATGAATCACGCTTTGATAAA
AACTTATGGCAAGGTGAGAAATGTCTGCGTGATTTTCTTGGAGACGGGTTAGCCACAAGCTGGACGCATG
AAAAAAATTGGGAAAAAAGCGCATAATATCTTACTTCCAAGCTTTAGTCAGCAGGCAATGAAAGGCTATC
ATGCGGTGATGGTCGATATCGCCGTGCAGCTTGTTCAAAAGTGGGAGCGTCTAAATGCAGATGAGCATAT
TGAAGTATCGGAAGACATGACACGTTTAACGCTTGATACAATTGGTCTTTGCGGCTTTAACTATCGCTTT
AACAGCTTTTACCGAGATCAGCCTCATCCATTTATTATAAGTCTGGTCCGTGCACTGGATGAAGTAATGA
ACAAGCTGCAGCGAGCAAATCCAGACGACCCAGCTTATGATGAAAACAAGCGCCAGTTTCAAGAAGATAT
CAAGGTGATGAACGACCTAGTAGATAAAATTATTGCAGATCGCAAAGCAAGGGGTGAACAAAGCGATGAT
TTATTAACGCAGATGCTAAACGGAAAAGATCCAGAAACGGGTGAGCCGCTTGATGACGGGAACATTCGCT
ATCAAATTATTACATTCTTATATGCGGGAGTTGAAGGTACAAGTGGTCTTTTATCATTTGCGCTGTATTT
CTTAGTGAAAAATCCACATGTATTACAAAAAGTAGCAGAAGAAGCAGCACGAGTTCTAGTAGATCCTGTT
CCAAGCTACAAACAAGTCAAACAGCTTAAATATGTCGGCATGGTCTTAAACGAAGCGCTGCGCTTATGGC
CAATTGTTCCTCAGTTTTCCCTATATGCAAAAGAAGATACGGTGCTTGGAGGAGAATATCCTTTAGAAAA
AGGCGACGAAGTAATGGTTCTGATTCCTCAGCTTCACCGTGATAAAACAGTTTGGGGAGACGATGTGGAG
GAGTTCCGTCCAGAGCGTTTTGAAAATCCAAGTGCGATTCCGCAGCATGCGTTTAAACCGTTTGGAAACG

GTCAGCGTGCGTCTATCGGTCAGCAGTTCGCTCTTCATGAAGCAACGCTGGTACTTGGTATGATGCTAAA
ACACTTTGACTTTGAAGATCATACAAACTACGAGCTCGATATTAAAGAACTGCTGACGTTAAAACCTAAA
GGCTTTGTGGTAAAAGCAAAATCGAAAAAAATTCCGCTTGGCGGTATTCCTTCACCTAGCACTGAACAGT
CTGCTAAAAAAGTACGCAAAAAGGCAGAAAACGCTCATAATACGCCGCTGCTTGTGCTATACGGTTCAAA
TATGGGTACCGCTGAAGGAACGGCGCGTGATTTAGCAGATATTGCAATGAGCAAAGGATTTGCACCGCAG
GTCGCAACGCTTGATTCACACGCCGGAAATCTTCCGCGCGAAGGAGCTGTATTAATTGTAACGGCGTCTT
ATAACGGTCATCCGCCTGATAACGCAAAGCAATTTGTCGACTGGTTAGACCAAGCGTCTGCTGATGAAGT
AAAAGGCGTTCGCTACTCCGTATTTGGATGCGGCGATAAAAACTGGGCTACTACGTATCAAAAAGTGCCT
GCTTTTATCGATGAAACGCTTGCCGCTAAAGGGGCAGAAAACATCGCTGACCGCGGTGAAGCAGATGCAA
GCGACGACTTTGAAGGCACATATGAAGAATGGCGTGAACATATGTGGAGTGACGTAGCAGCCTACTTTAA
CCTCGACATTGAAAACAGTGAAGATAATAAATCTACTCTTTCACTTCAATTTGTCGACAGCGCCGCGGAT
ATGCCGCTTGCGAAAATGCACGGTGCGTTTTCAACGAACGTCGTAGCAAGCAAAGAACTTCAACAGCCAG
GCAGTGCACGAAGCACGCGACATCTTGAAATTGAACTTCCAAAAGAAGCTTCTTATCAAGAAGGAGATCA
TTTAGGTGTTATTCCTCGCAACTATGAAGGAATAGTAAACCGTGTAACAGCAAGGTTCGGCCTAGATGCA
TCACAGCAAATCCGTCTGGAAGCAGAAGAAGAAAAATTAGCTCATTTGCCACTCGCTAAAACAGTATCCG
TAGAAGAGCTTCTGCAATACGTGGAGCTTCAAGATCCTGTTACGCGCACGCAGCTTCGCGCAATGGCTGC
TAAAACGGTCTGCCCGCCGCATAAAGTAGAGCTTGAAGCCTTGCTTGAAAAGCAAGCCTACAAAGAACAA
GTGCTGGCAAAACGTTTAACAATGCTTGAACTGCTTGAAAAATACCCGGCGTGTGAAATGAAATTCAGCG
AATTTATCGCCCTTCTGCCAAGCATACGCCCGCGCTATTACTCGATTTCTTCATCACCTCGTGTCGATGA
AAAACAAGCAAGCATCACGGTCAGCGTTGTCTCAGGAGAAGCGTGGAGCGGATATGGAGAATATAAAGGA
ATTGCGTCGAACTATCTTGCCGAGCTGCAAGAAGGAGATACGATTACGTGCTTTATTTCCACACCGCAGT
CAGAATTTACGCTGCCAAAAGACCCTGAAACGCCGCTTATCATGGTCGGACCGGGAACAGGCGTCGCGCC
GTTTAGAGGCTTTGTGCAGGCGCGCAAACAGCTAAAAGAACAAGGACAGTCACTTGGAGAAGCACATTTA
TACTTCGGCTGCCGTTCACCTCATGAAGACTATCTGTATCAAGAAGAGCTTGAAAACGCCCAAAGCGAAG
GCATCATTACGCTTCATACCGCTTTTTCTCGCATGCCAAATCAGCCGAAAACATACGTTCAGCACGTAAT
GGAACAAGACGGCAAGAAATTGATTGAACTTCTTGATCAAGGAGCGCACTTCTATATTTGCGGAGACGGA
AGCCAAATGGCACCTGCCGTTGAAGCAACGCTTATGAAAAGCTATGCTGACGTTCACCAAGTGAGTGAAG
CAGACGCTCGCTTATGGCTGCAGCAGCTAGAAGAAAAAGGCCGATACGCAAAAGACGTGTGGGCTGGGCT
CGAGCACCACCACCACCACCACTGA

MTIKEMPQPKTFGELKNLPLLNTDKPVQALMKIADELGEIFKFEAPGRVTRYLSSQRLIKEACDESRFDK
NLWQGEKCLRDFLGDGLATSWTHEKNWKKAHNILLPSFSQQAMKGYHAVMVDIAVQLVQKWERLNADEHI
EVSEDMTRLTLDTIGLCGFNYRFNSFYRDQPHPFIISLVRALDEVMNKLQRANPDDPAYDENKRQFQEDI
KVMNDLVDKIIADRKARGEQSDDLLTQMLNGKDPETGEPLDDGNIRYQIITFLYAGVEGTSGLLSFALYF
LVKNPHVLQKVAEEAARVLVDPVPSYKQVKQLKYVGMVLNEALRLWPIVPQFSLYAKEDTVLGGEYPLEK
GDEVMVLIPQLHRDKTVWGDDVEEFRPERFENPSAIPQHAFKPFGNGQRASIGQQFALHEATLVLGMMLK
HFDFEDHTNYELDIKELLTLKPKGFVVKAKSKKIPLGGIPSPSTEQSAKKVRKKAENAHNTPLLVLYGSN
MGTAEGTARDLADIAMSKGFAPQVATLDSHAGNLPREGAVLIVTASYNGHPPDNAKQFVDWLDQASADEV
KGVRYSVFGCGDKNWATTYQKVPAFIDETLAAKGAENIADRGEADASDDFEGTYEEWREHMWSDVAAYFN
LDIENSEDNKSTLSLQFVDSAADMPLAKMHGAFSTNVVASKELQQPGSARSTRHLEIELPKEASYQEGDH
LGVIPRNYEGIVNRVTARFGLDASQQIRLEAEEEKLAHLPLAKTVSVEELLQYVELQDPVTRTQLRAMAA
KTVCPPHKVELEALLEKQAYKEQVLAKRLTMLELLEKYPACEMKFSEFIALLPSIRPRYYSISSSPRVDE
KQASITVSVVSGEAWSGYGEYKGIASNYLAELQEGDTITCFISTPQSEFTLPKDPETPLIMVGPGTGVAP
FRGFVQARKQLKEQGQSLGEAHLYFGCRSPHEDYLYQEELENAQSEGIITLHTAFSRMPNQPKTYVQHVM
EQDGKKLIELLDQGAHFYICGDGSQMAPAVEATLMKSYADVHQVSEADARLWLQQLEEKGRYAKDVWAGL
EHHHHHH

Nucleotide and amino acid sequences of P411-PYS-5148:
ATGACAATTAAAGAAATGCCTCAGCCAAAAACGTTTGGAGAGCTTAAAAATTTACCGTTATTAAACACAG
ATAAACCGGTTCAAGCTTTGATGAAAATTGCGGATGAATTAGGAGAAATCTTTAAATTCGAGGCGCCTGG
TCGTGTAACGCGCTACTTATCAAGTCAGCGTCTAATTAAAGAAGCATGCGATGAATCACGCTTTGATAAA

AACTTATGGGCGGGTGAGAAATGTCTGCGTGATTTTCTTGGAGACGGGTTAGCCACAAGCTGGACGCATG
AAAAAAATTGGAAAAAAGCGCATAATATCTTACTTCCAAGCTTTAGTCAGCAGGCAATGAAAGGCTATCA
TGCGGTGATGGTCGATATCGCCGTGCAGCTTGTTCAAAAGTGGGAGCGTCTAAATGCAGATGAGCATATT
GAAGTATCGGAAGACATGACACGTTTAACGCTTGATACAATTGGTCTTTGCGGCTTTAACTATCGCTTTA
ACAGCTTTTACCGAGATCAGCCTCATCCATTTATTATAAGTCTGGTCCGTGCACTGGATGAAGTAATGAA
CAAGCTGCAGCGAGCAAATCCAGACGACCCAGCTTATGATGAAAACAAGCGCCAGTTTCAAGAAGATATC
AAGGTGATGAACGACCTAGTAGATAAAATTATTGCAGATCGCAAAGCAAGGGGTGAACAAAGCGATGATT
TATTAACGCAGATGCTAAACGGAAAAGATCCAGAAACGGGTGAGCCGCTTGATGACGGGAACATTCGCTA
TCAAATTATTACATTCTTATATGCGGGAGTTGAAGGTACAAGTGGTCTTTTATCATTTGCGCTGTATTTC
TTAGTGAAAAATCCACATGTATTACAAAAAGTAGCAGAAGAAGCAGCACGAGTTCTAGTAGATCCTGTTC
CAAGCTACAAACAAGTCAAACAGCTTAAATATGTCGGCATGGTCTTAAACGAAGCGCTGCGCTTATGGCC
AATTGTTCCTCAGTTTTCCCTATATGCAAAAGAAGATACGGTGCTTGGAGGAGAATATCCTTTAGAAAAA
GGCGACGAAGTAATGGTTCTGATTCCTCAGCTTCACCGTGATAAAACAGTTTGGGGAGACGATGTGGAGG
AGTTCCGTCCAGAGCGTTTTGAAAATCCAAGTGCGATTCCGCAGCATGCGTTTAAACCGTTTGGAAACGG
TCAGCGTGCGTCTATCGGTCAGCAGTTCGCTCTTCATGAAGCAACGCTGGTACTTGGTATGATGCTAAAA
CACTTTGACTTTGAAGATCATACAAACTACGAGCTCGATATTAAAGAACTGCTGACGTTAAAACCTAAAG
GCTTTGTGGTAAAAGCAAAATCGAAAAAAATTCCGCTTGGCGGTATTCCTTCACCTAGCACTGAACAGTC
TGCTAAAAAAGTACGCAAAAAGGCAGAAAACGCTCATAATACGCCGCTGCTTGTGCTATACGGTTCAAAT
ATGGGTACCGCTGAAGGAACGGCGCGTGATTTAGCAGATATTGCAATGAGCAAAGGATTTGCACCGCAGG
TCGCAACGCTTGATTCACACGCCGGAAATCTTCCGCGCGAAGGAGCTGTATTAATTGTAACGGCGTCTTA
TAACGGTCATCCGCCTGATAACGCAAAGCAATTTGTCGACTGGTTAGACCAAGCGTCTGCTGATGAAGTA
AAAGGCGTTCGCTACTCCGTATTTGGATGCGGCGATAAAAACTGGGCTACTACGTATCAAAAAGTGCCTG
CTTTTATCGATGAAACGCTTGCCGCTAAAGGGGCAGAAAACATCGCTGACCGCGGTGAAGCAGATGCAAG
CGACGACTTTGAAGGCACATATGAAGAATGGCGTGAACATATGTGGAGTGACGTAGCAGCCTACTTTAAC
CTCGACATTGAAAACAGTGAAGATAATAAATCTACTCTTTCACTTCAATTTGTCGACAGCGCCGCGGATA
TGCCGCTTGCGAAAATGCACGGTGCGTTTTCAACGAACGTCGTAGCAAGCAAAGAACTTCAACAGCCAGG
CAGTGCACGAAGCACGCGACATCTTGAAATTGAACTTCCAAAAGAAGCTTCTTATCAAGAAGGAGATCAT
TTAGGTGTTATTCCTCGCAACTATGAAGGAATAGTAAACCGTGTAACAGCAAGGTTCGGCCTAGATGCAT
CACAGCAAATCCGTCTGGAAGCAGAAGAAGAAAAATTAGCTCATTTGCCACTCGCTAAAACAGTATCCGT
AGAAGAGCTTCTGCAATACGTGGAGCTTCAAGATCCTGTTACGCGCACGCAGCTTCGCGCAATGGCTGCT
AAAACGGTCTGCCCGCCGCATAAAGTAGAGCTTGAAGCCTTGCTTGAAAAGCAAGCCTACAAAGAACAAG
TGCTGGCAAAACGTTTAACAATGCTTGAACTGCTTGAAAAATACCCGGCGTGTGAAATGAAATTCAGCGA
ATTTATCGCCCTTCTGCCAAGCATACGCCCGCGCTATTACTCGATTTCTTCATCACCTCGTGTCGATGAA
AAACAAGCAAGCATCACGGTCAGCGTTGTCTCAGGAGAAGCGTGGAGCGGATATGGAGAATATAAAGGAA
TTGCGTCGAACTATCTTGCCGAGCTGCAAGAAGGAGATACGATTACGTGCTTTATTTCCACACCGCAGTC
AGAATTTACGCTGCCAAAAGACCCTGAAACGCCGCTTATCATGGTCGGACCGGGAACAGGCGTCGCGCCG
TTTAGAGGCTTTGTGCAGGCGCGCAAACAGCTAAAAGAACAAGGACAGTCACTTGGAGAAGCACATTTAT
ACTTCGGCTGCCGTTCACCTCATGAAGACTATCTGTATCAAGAAGAGCTCGAAAACGCCCAAAGCGAAGG
CATCATTACGCTTCATACCGCTTTTTCTCGCATGCCAAATCAGCCGAAAACATACGTTCAGCACGTAATG
GAACAAGACGGCAAGAAATTGATTGAACTTCTTGATCAAGGAGCGCACTTCTATATTTGCGGAGACGGAA
GCCAAATGGCACCTGCCGTTGAAGCAACGCTTATGAAAAGCTATGCTGACGTTCACCAAGTGAGTGAAGC
AGACGCTCGCTTATGGCTGCAGCAGCTAGAAGAAAAAGGCCGATACGCAAAAGACGTGTGGGCTGGGCTC
GAGCACCACCACCACCACCACTGA

MTIKEMPQPKTFGELKNLPLLNTDKPVQALMKIADELGEIFKFEAPGRVTRYLSSQRLIKEACDESRFDK
NLWAGEKCLRDFLGDGLATSWTHEKNWKKAHNILLPSFSQQAMKGYHAVMVDIAVQLVQKWERLNADEHI
EVSEDMTRLTLDTIGLCGFNYRFNSFYRDQPHPFIISLVRALDEVMNKLQRANPDDPAYDENKRQFQEDI
KVMNDLVDKIIADRKARGEQSDDLLTQMLNGKDPETGEPLDDGNIRYQIITFLYAGVEGTSGLLSFALYF
LVKNPHVLQKVAEEAARVLVDPVPSYKQVKQLKYVGMVLNEALRLWPIVPQFSLYAKEDTVLGGEYPLEK
GDEVMVLIPQLHRDKTVWGDDVEEFRPERFENPSAIPQHAFKPFGNGQRASIGQQFALHEATLVLGMMLK
HFDFEDHTNYELDIKELLTLKPKGFVVKAKSKKIPLGGIPSPSTEQSAKKVRKKAENAHNTPLLVLYGSN
MGTAEGTARDLADIAMSKGFAPQVATLDSHAGNLPREGAVLIVTASYNGHPPDNAKQFVDWLDQASADEV

KGVRYSVFGCGDKNWATTYQKVPAFIDETLAAKGAENIADRGEADASDDFEGTYEEWREHMWSDVAAYFN
LDIENSEDNKSTLSLQFVDSAADMPLAKMHGAFSTNVVASKELQQPGSARSTRHLEIELPKEASYQEGDH
LGVIPRNYEGIVNRVTARFGLDASQQIRLEAEEEKLAHLPLAKTVSVEELLQYVELQDPVTRTQLRAMAA
KTVCPPHKVELEALLEKQAYKEQVLAKRLTMLELLEKYPACEMKFSEFIALLPSIRPRYYSISSSPRVDE
KQASITVSVVSGEAWSGYGEYKGIASNYLAELQEGDTITCFISTPQSEFTLPKDPETPLIMVGPGTGVAP
FRGFVQARKQLKEQGQSLGEAHLYFGCRSPHEDYLYQEELENAQSEGIITLHTAFSRMPNQPKTYVQHVM
EQDGKKLIELLDQGAHFYICGDGSQMAPAVEATLMKSYADVHQVSEADARLWLQQLEEKGRYAKDVWAGL
EHHHHHH

Nucleotide and amino acid sequences of P411-PYS-5149:
ATGACAATTAAAGAAATGCCTCAGCCAAAAACGTTTGGAGAGCTTAAAAATTTACCGTTATTAAACACAG
ATAAACCGGTTCAAGCTTTGATGAAAATTGCGGATGAATTAGGAGAAATCTTTAAATTCGAGGCGCCTGG
TCGTGTAACGCGCTACTTATCAAGTCAGCGTCTAATTAAAGAAGCATGCGATGAATCACGCTTTGATAAA
AACTTATGGGCGGGTGAGAAATGTCTGCGTGATTTTCTTGGAGACGGGTTAGCCACAAGCTGGACGCATG
AAAAAAATTGGAAAAAAGCGCATAATATCTTACTTCCAAGCTTTAGTCAGCAGGCAATGAAAGGCTATCA
TGCGGTGATGGTCGATATCGCCGTGCAGCTTGTTCAAAAGTGGGAGCGTCTAAATGCAGATGAGCATATT
GAAGTATCGGAAGACATGACACGTTTAACGCTTGATACAATTGGTCTTTGCGGCTTTAACTATCGCTTTA
ACAGCTTTTACCGAGATCAGCCTCATCCATTTATTATAAGTCTGGTCCGTGCACTGGATGAAGTAATGAA
CAAGCTGCAGCGAGCAAATCCAGACGACCCAGCTTATGATGAAAACAAGCGCCAGTTTCAAGAAGATATC
AAGGTGATGAACGACCTAGTAGATAAAATTATTGCAGATCGCAAAGCAAGGGGTGAACAAAGCGATGATT
TATTAACGCAGATGCTAAACGGAAAAGATCCAGAAACGGGTGAGCCGCTTGATGACGGGAACATTCGCTA
TCAAATTATTACATTCTTATATGCGGGAGTTGAAGGTACAAGTGGTCTTTTATCATTTGCGCTGTATTTC
TTAGTGAAAAATCCACATGTATTACAAAAAGTAGCAGAAGAAGCAGCACGAGTTCTAGTAGATCCTGTTC
CAAGCTACAAACAAGTCAAACAGCTTAAATATGTCGGCATGGTCTTAAACGAAGCGCTGCGCTTATGGCC
AATTGTTCCTCAGTTTTCCCTATATGCAAAAGAAGATACGGTGCTTGGAGGAGAATATCCTTTAGAAAAA
GGCGACGAAGTAATGGTTCTGATTCCTCAGCTTCACCGTGATAAAACAGTTTGGGGAGACGATGTGGAGG
AGTTCCGTCCAGAGCGTTTTGAAAATCCAAGTGCGATTCCGCAGCATGCGTTTAAACCGTTTGGAAACGG
TCAGCGTGCGTCTATCGGTCAGCAGTTCGCTCTTCATGAAGCAACGCTGGTACTTGGTATGATGCTAAAA
CACTTTGACTTTGAAGATCATACAAACTACGAGCTCGATATTAAAGAACGTCTGACGTTAAAACCTAAAG
GCTTTGTGGTAAAAGCAAAATCGAAAAAAATTCCGCTTGGCGGTATTCCTTCACCTAGCACTGAACAGTC
TGCTAAAAAAGTACGCAAAAAGGCAGAAAACGCTCATAATACGCCGCTGCTTGTGCTATACGGTTCAAAT
ATGGGTACCGCTGAAGGAACGGCGCGTGATTTAGCAGATATTGCAATGAGCAAAGGATTTGCACCGCAGG
TCGCAACGCTTGATTCACACGCCGGAAATCTTCCGCGCGAAGGAGCTGTATTAATTGTAACGGCGTCTTA
TAACGGTCATCCGCCTGATAACGCAAAGCAATTTGTCGACTGGTTAGACCAAGCGTCTGCTGATGAAGTA
AAAGGCGTTCGCTACTCCGTATTTGGATGCGGCGATAAAAACTGGGCTACTACGTATCAAAAAGTGCCTG
CTTTTATCGATGAAACGCTTGCCGCTAAAGGGGCAGAAAACATCGCTGACCGCGGTGAAGCAGATGCAAG
CGACGACTTTGAAGGCACATATGAAGAATGGCGTGAACATATGTGGAGTGACGTAGCAGCCTACTTTAAC
CTCGACATTGAAAACAGTGAAGATAATAAATCTACTCTTTCACTTCAATTTGTCGACAGCGCCGCGGATA
TGCCGCTTGCGAAAATGCACGGTGCGTTTTCAACGAACGTCGTAGCAAGCAAAGAACTTCAACAGCCAGG
CAGTGCACGAAGCACGCGACATCTTGAAATTGAACTTCCAAAAGAAGCTTCTTATCAAGAAGGAGATCAT
TTAGGTGTTATTCCTCGCAACTATGAAGGAATAGTAAACCGTGTAACAGCAAGGTTCGGCCTAGATGCAT
CACAGCAAATCCGTCTGGAAGCAGAAGAAGAAAAATTAGCTCATTTGCCACTCGCTAAAACAGTATCCGT
AGAAGAGCTTCTGCAATACGTGGAGCTTCAAGATCCTGTTACGCGCACGCAGCTTCGCGCAATGGCTGCT
AAAACGGTCTGCCCGCCGCATAAAGTAGAGCTTGAAGCCTTGCTTGAAAAGCAAGCCTACAAAGAACAAG
TGCTGGCAAAACGTTTAACAATGCTTGAACTGCTTGAAAAATACCCGGCGTGTGAAATGAAATTCAGCGA
ATTTATCGCCCTTCTGCCAAGCATACGCCCGCGCTATTACTCGATTTCTTCATCACCTCGTGTCGATGAA
AAACAAGCAAGCATCACGGTCAGCGTTGTCTCAGGAGAAGCGTGGAGCGGATATGGAGAATATAAAGGAA
TTGCGTCGAACTATCTTGCCGAGCTGCAAGAAGGAGATACGATTACGTGCTTTATTTCCACACCGCAGTC
AGAATTTACGCTGCCAAAAGACCCTGAAACGCCGCTTATCATGGTCGGACCGGGAACAGGCGTCGCGCCG
TTTAGAGGCTTTGTGCAGGCGCGCAAACAGCTAAAAGAACAAGGACAGTCACTTGGAGAAGCACATTTAT
ACTTCGGCTGCCGTTCACCTCATGAAGACTATCTGTATCAAGAAGAGCTCGAAAACGCCCAAAGCGAAGG

CATCATTACGCTTCATACCGCTTTTTCTCGCATGCCAAATCAGCCGAAAACATACGTTCAGCACGTAATG
GAACAAGACGGCAAGAAATTGATTGAACTTCTTGATCAAGGAGCGCACTTCTATATTTGCGGAGACGGAA
GCCAAATGGCACCTGCCGTTGAAGCAACGCTTATGAAAAGCTATGCTGACGTTCACCAAGTGAGTGAAGC
AGACGCTCGCTTATGGCTGCAGCAGCTAGAAGAAAAAGGCCGATACGCAAAAGACGTGTGGGCTGGGCTC
GAGCACCACCACCACCACCACTGA

MTIKEMPQPKTFGELKNLPLLNTDKPVQALMKIADELGEIFKFEAPGRVTRYLSSQRLIKEACDESRFDK
NLWAGEKCLRDFLGDGLATSWTHEKNWKKAHNILLPSFSQQAMKGYHAVMVDIAVQLVQKWERLNADEHI
EVSEDMTRLTLDTIGLCGFNYRFNSFYRDQPHPFIISLVRALDEVMNKLQRANPDDPAYDENKRQFQEDI
KVMNDLVDKIIADRKARGEQSDDLLTQMLNGKDPETGEPLDDGNIRYQIITFLYAGVEGTSGLLSFALYF
LVKNPHVLQKVAEEAARVLVDPVPSYKQVKQLKYVGMVLNEALRLWPIVPQFSLYAKEDTVLGGEYPLEK
GDEVMVLIPQLHRDKTVWGDDVEEFRPERFENPSAIPQHAFKPFGNGQRASIGQQFALHEATLVLGMMLK
HFDFEDHTNYELDIKERLTLKPKGFVVKAKSKKIPLGGIPSPSTEQSAKKVRKKAENAHNTPLLVLYGSN
MGTAEGTARDLADIAMSKGFAPQVATLDSHAGNLPREGAVLIVTASYNGHPPDNAKQFVDWLDQASADEV
KGVRYSVFGCGDKNWATTYQKVPAFIDETLAAKGAENIADRGEADASDDFEGTYEEWREHMWSDVAAYFN
LDIENSEDNKSTLSLQFVDSAADMPLAKMHGAFSTNVVASKELQQPGSARSTRHLEIELPKEASYQEGDH
LGVIPRNYEGIVNRVTARFGLDASQQIRLEAEEEKLAHLPLAKTVSVEELLQYVELQDPVTRTQLRAMAA
KTVCPPHKVELEALLEKQAYKEQVLAKRLTMLELLEKYPACEMKFSEFIALLPSIRPRYYSISSSPRVDE
KQASITVSVVSGEAWSGYGEYKGIASNYLAELQEGDTITCFISTPQSEFTLPKDPETPLIMVGPGTGVAP
FRGFVQARKQLKEQGQSLGEAHLYFGCRSPHEDYLYQEELENAQSEGIITLHTAFSRMPNQPKTYVQHVM
EQDGKKLIELLDQGAHFYICGDGSQMAPAVEATLMKSYADVHQVSEADARLWLQQLEEKGRYAKDVWAGL
EHHHHHH

Nucleotide and amino acid sequences of P411-INS-5150:

ATGACAATTAAAGAAATGCCTCAGCCAAAAACGTTTGGAGAGCTTAAAAATTTACCGTTATTAAACACAG
ATAAACCGGTTCAAGCTTTGATGAAAATTGCGGATGAATTAGGAGAAATCTTTAAATTCGAGGCGCCTGG
TCGTGTAACGCGCTACTTATCAAGTCAGCGTCTAATTAAAGAAGCATGCGATGAATCACGCTTTGATAAA
AACTTATGGGCGGGTGAGAAATGTCTGCGTGATTTTCTTGGAGACGGGTTAGCCACAAGCTGGACGCATG
AAAAAAATTGGAAAAAAGCGCATAATATCTTACTTCCAAGCTTTAGTCAGCAGGCAATGAAAGGCTATCA
TGCGGTGATGGTCGATATCGCCGTGCAGCTTGTTCAAAAGTGGGAGCGTCTAAATGCAGATGAGCATATT
GAAGTATCGGAAGACATGACACGTTTAACGCTTGATACAATTGGTCTTTGCGGCTTTAACTATCGCTTTA
ACAGCTTTTACCGAGATCAGCCTCATCCATTTATTATAAGTCTGGTCCGTGCACTGGATGAAGTAATGAA
CAAGCTGCAGCGAGCAAATCCAGACGACCCAGCTTATGATGAAAACAAGCGCCAGTTTCAAGAAGATATC
AAGGTGATGAACGACCTAGTAGATAAAATTATTGCAGATCGCAAAGCAAGGGGTGAACAAAGCGATGATT
TATTAACGCAGATGCTAAACGGAAAAGATCCAGAAACGGGTGAGCCGCTTGATGACGGGAACATTCGCTA
TCAAATTATTACATTCTTATATGCGGGAGTTGAAGGTACAAGTGGTCTTTTATCATTTGCGCTGTATTTC
TTAGTGAAAAATCCACATGTATTACAAAAAGTAGCAGAAGAAGCAGCACGAGTTCTAGTAGATCCTGTTC
CAAGCTACAAACAAGTCAAACAGCTTAAATATGTCGGCATGGTCTTAAACGAAGCGCTGCGCTTATGGCC
AATTGTTCCTCAGTTTTCCCTATATGCAAAAGAAGATACGGTGCTTGGAGGAGAATATCCTTTAGAAAAA
GGCGACGAAGTAATGGTTCTGATTCCTCAGCTTCACCGTGATAAAACAGTTTGGGGAGACGATGTGGAGG
AGTTCCGTCCAGAGCGTTTTGAAAATCCAAGTGCGATTCCGCAGCATGCGTTTAAACCGTTTGGAAACGG
TCAGCGTGCGTCTATCGGTCAGCAGTTCGCTCTTCATGAAGCAACGCTGGTACTTGGTATGATGCTAAAA
CACTTTGACTTTGAAGATCATACAAACTACGAGCTCGATATTAAAGAACTGCCGACGTTAAAACCTAAAG
GCTTTGTGGTAAAAGCAAAATCGAAAAAAATTCCGCTTGGCGGTATTCCTTCACCTAGCACTGAACAGTC
TGCTAAAAAAGTACGCAAAAAGGCAGAAAACGCTCATAATACGCCGCTGCTTGTGCTATACGGTTCAAAT
ATGGGTACCGCTGAAGGAACGGCGCGTGATTTAGCAGATATTGCAATGAGCAAAGGATTTGCACCGCAGG
TCGCAACGCTTGATTCACACGCCGGAAATCTTCCGCGCGAAGGAGCTGTATTAATTGTAACGGCGTCTTA
TAACGGTCATCCGCCTGATAACGCAAAGCAATTTGTCGACTGGTTAGACCAAGCGTCTGCTGATGAAGTA
AAAGGCGTTCGCTACTCCGTATTTGGATGCGGCGATAAAAACTGGGCTACTACGTATCAAAAAGTGCCTG
CTTTTATCGATGAAACGCTTGCCGCTAAAGGGGCAGAAAACATCGCTGACCGCGGTGAAGCAGATGCAAG
CGACGACTTTGAAGGCACATATGAAGAATGGCGTGAACATATGTGGAGTGACGTAGCAGCCTACTTTAAC

CTCGACATTGAAAACAGTGAAGATAATAAATCTACTCTTTCACTTCAATTTGTCGACAGCGCCGCGGATA
TGCCGCTTGCGAAAATGCACGGTGCGTTTTCAACGAACGTCGTAGCAAGCAAAGAACTTCAACAGCCAGG
CAGTGCACGAAGCACGCGACATCTTGAAATTGAACTTCCAAAAGAAGCTTCTTATCAAGAAGGAGATCAT
TTAGGTGTTATTCCTCGCAACTATGAAGGAATAGTAAACCGTGTAACAGCAAGGTTCGGCCTAGATGCAT
CACAGCAAATCCGTCTGGAAGCAGAAGAAGAAAAATTAGCTCATTTGCCACTCGCTAAAACAGTATCCGT
AGAAGAGCTTCTGCAATACGTGGAGCTTCAAGATCCTGTTACGCGCACGCAGCTTCGCGCAATGGCTGCT
AAAACGGTCTGCCCGCCGCATAAAGTAGAGCTTGAAGCCTTGCTTGAAAAGCAAGCCTACAAAGAACAAG
TGCTGGCAAAACGTTTAACAATGCTTGAACTGCTTGAAAAATACCCGGCGTGTGAAATGAAATTCAGCGA
ATTTATCGCCCTTCTGCCAAGCATACGCCCGCGCTATTACTCGATTTCTTCATCACCTCGTGTCGATGAA
AAACAAGCAAGCATCACGGTCAGCGTTGTCTCAGGAGAAGCGTGGAGCGGATATGGAGAATATAAAGGAA
TTGCGTCGAACTATCTTGCCGAGCTGCAAGAAGGAGATACGATTACGTGCTTTATTTCCACACCGCAGTC
AGAATTTACGCTGCCAAAAGACCCTGAAACGCCGCTTATCATGGTCGGACCGGGAACAGGCGTCGCGCCG
TTTAGAGGCTTTGTGCAGGCGCGCAAACAGCTAAAAGAACAAGGACAGTCACTTGGAGAAGCACATTTAT
ACTTCGGCTGCCGTTCACCTCATGAAGACTATCTGTATCAAGAAGAGCTCGAAAACGCCCAAAGCGAAGG
CATCATTACGCTTCATACCGCTTTTTCTCGCATGCCAAATCAGCCGAAAACATACGTTCAGCACGTAATG
GAACAAGACGGCAAGAAATTGATTGAACTTCTTGATCAAGGAGCGCACTTCTATATTTGCGGAGACGGAA
GCCAAATGGCACCTGCCGTTGAAGCAACGCTTATGAAAAGCTATGCTGACGTTCACCAAGTGAGTGAAGC
AGACGCTCGCTTATGGCTGCAGCAGCTAGAAGAAAAAGGCCGATACGCAAAAGACGTGTGGGCTGGGCTC
GAGCACCACCACCACCACCACTGA

MTIKEMPQPKTFGELKNLPLLNTDKPVQALMKIADELGEIFKFEAPGRVTRYLSSQRLIKEACDESRFDK
NLWAGEKCLRDFLGDGLATSWTHEKNWKKAHNILLPSFSQQAMKGYHAVMVDIAVQLVQKWERLNADEHI
EVSEDMTRLTLDTIGLCGFNYRFNSFYRDQPHPFIISLVRALDEVMNKLQRANPDDPAYDENKRQFQEDI
KVMNDLVDKIIADRKARGEQSDDLLTQMLNGKDPETGEPLDDGNIRYQIITFLYAGVEGTSGLLSFALYF
LVKNPHVLQKVAEEAARVLVDPVPSYKQVKQLKYVGMVLNEALRLWPIVPQFSLYAKEDTVLGGEYPLEK
GDEVMVLIPQLHRDKTVWGDDVEEFRPERFENPSAIPQHAFKPFGNGQRASIGQQFALHEATLVLGMMLK
HFDFEDHTNYELDIKELPTLKPKGFVVKAKSKKIPLGGIPSPSTEQSAKKVRKKAENAHNTPLLVLYGSN
MGTAEGTARDLADIAMSKGFAPQVATLDSHAGNLPREGAVLIVTASYNGHPPDNAKQFVDWLDQASADEV
KGVRYSVFGCGDKNWATTYQKVPAFIDETLAAKGAENIADRGEADASDDFEGTYEEWREHMWSDVAAYFN
LDIENSEDNKSTLSLQFVDSAADMPLAKMHGAFSTNVVASKELQQPGSARSTRHLEIELPKEASYQEGDH
LGVIPRNYEGIVNRVTARFGLDASQQIRLEAEEEKLAHLPLAKTVSVEELLQYVELQDPVTRTQLRAMAA
KTVCPPHKVELEALLEKQAYKEQVLAKRLTMLELLEKYPACEMKFSEFIALLPSIRPRYYSISSSPRVDE
KQASITVSVVSGEAWSGYGEYKGIASNYLAELQEGDTITCFISTPQSEFTLPKDPETPLIMVGPGTGVAP
FRGFVQARKQLKEQGQSLGEAHLYFGCRSPHEDYLYQEELENAQSEGIITLHTAFSRMPNQPKTYVQHVM
EQDGKKLIELLDQGAHFYICGDGSQMAPAVEATLMKSYADVHQVSEADARLWLQQLEEKGRYAKDVWAGL
EHHHHHH

Nucleotide and amino acid sequences of P411-INS-5151:

ATGACAATTAAAGAAATGCCTCAGCCAAAAACGTTTGGAGAGCTTAAAAATTTACCGTTATTAAACACAG
ATAAACCGGTTCAAGCTTTGATGAAAATTGCGGATGAATTAGGAGAAATCTTTAAATTCGAGGCGCCTGG
TCGTGTAACGCGCTACTTATCAAGTCAGCGTCTAATTAAAGAAGCATGCGATGAATCACGCTTTGATAAA
AACTTATGGGCGGGTGAGAAATGTCTGCGTGATTTTCTTGGAGACGGGTTAGCCACAAGCTGGACGCATG
AAAAAAATTGGAAAAAAGCGCATAATATCTTACTTCCAAGCTTTAGTCAGCAGGCAATGAAAGGCTATCA
TGCGGTGATGGTCGATATCGCCGTGCAGCTTGTTCAAAAGTGGGAGCGTCTAAATGCAGATGAGCATATT
GAAGTATCGGAAGACATGACACGTTTAACGCTTGATACAATTGGTCTTTGCGGCTTTAACTATCGCTTTA
ACAGCTTTTACCGAGATCAGCCTCATCCATTTATTATAAGTCTGGTCCGTGCAAATGATGAAGTAATGAA
CAAGCTGCAGCGAGCAAATCCAGACGACCCAGCTTATGATGAAAACAAGCGCCAGTTTCAAGAAGATATC
AAGGTGATGAACGACCTAGTAGATAAAATTATTGCAGATCGCAAAGCAAGGGGTGAACAAAGCGATGATT
TATTAACGCAGATGCTAAACGGAAAAGATCCAGAAACGGGTGAGCCGCTTGATGACGGGAACATTCGCTA
TCAAATTATTACATTCTTATATGCGGGAGTTGAAGGTACAAGTGGTCTTTTATCATTTGCGCTGTATTTC
TTAGTGAAAAATCCACATGTATTACAAAAAGTAGCAGAAGAAGCAGCACGAGTTCTAGTAGATCCTGTTC

CAAGCTACAAACAAGTCAAACAGCTTAAATATGTCGGCATGGTCTTAAACGAAGCGCTGCGCTTATGGCC
AATTGTTCCTCAGTTTTCCCTATATGCAAAAGAAGATACGGTGCTTGGAGGAGAATATCCTTTAGAAAAA
GGCGACGAAGTAATGGTTCTGATTCCTCAGCTTCACCGTGATAAAACAGTTTGGGGAGACGATGTGGAGG
AGTTCCGTCCAGAGCGTTTTGAAAATCCAAGTGCGATTCCGCAGCATGCGTTTAAACCGTTTGGAAACGG
TCAGCGTGCGTCTATCGGTCAGCAGTTCGCTCTTCATGAAGCAACGCTGGTACTTGGTATGATGCTAAAA
CACTTTGACTTTGAAGATCATACAAACTACGAGCTCGATATTAAAGAACTGCCGACGTTAAAACCTAAAG
GCTTTGTGGTAAAAGCAAAATCGAAAAAAATTCCGCTTGGCGGTATTCCTTCACCTAGCACTGAACAGTC
TGCTAAAAAAGTACGCAAAAAGGCAGAAAACGCTCATAATACGCCGCTGCTTGTGCTATACGGTTCAAAT
ATGGGTACCGCTGAAGGAACGGCGCGTGATTTAGCAGATATTGCAATGAGCAAAGGATTTGCACCGCAGG
TCGCAACGCTTGATTCACACGCCGGAAATCTTCCGCGCGAAGGAGCTGTATTAATTGTAACGGCGTCTTA
TAACGGTCATCCGCCTGATAACGCAAAGCAATTTGTCGACTGGTTAGACCAAGCGTCTGCTGATGAAGTA
AAAGGCGTTCGCTACTCCGTATTTGGATGCGGCGATAAAAACTGGGCTACTACGTATCAAAAAGTGCCTG
CTTTTATCGATGAAACGCTTGCCGCTAAAGGGGCAGAAAACATCGCTGACCGCGGTGAAGCAGATGCAAG
CGACGACTTTGAAGGCACATATGAAGAATGGCGTGAACATATGTGGAGTGACGTAGCAGCCTACTTTAAC
CTCGACATTGAAAACAGTGAAGATAATAAATCTACTCTTTCACTTCAATTTGTCGACAGCGCCGCGGATA
TGCCGCTTGCGAAAATGCACGGTGCGTTTTCAACGAACGTCGTAGCAAGCAAAGAACTTCAACAGCCAGG
CAGTGCACGAAGCACGCGACATCTTGAAATTGAACTTCCAAAAGAAGCTTCTTATCAAGAAGGAGATCAT
TTAGGTGTTATTCCTCGCAACTATGAAGGAATAGTAAACCGTGTAACAGCAAGGTTCGGCCTAGATGCAT
CACAGCAAATCCGTCTGGAAGCAGAAGAAGAAAAATTAGCTCATTTGCCACTCGCTAAAACAGTATCCGT
AGAAGAGCTTCTGCAATACGTGGAGCTTCAAGATCCTGTTACGCGCACGCAGCTTCGCGCAATGGCTGCT
AAAACGGTCTGCCCGCCGCATAAAGTAGAGCTTGAAGCCTTGCTTGAAAAGCAAGCCTACAAAGAACAAG
TGCTGGCAAAACGTTTAACAATGCTTGAACTGCTTGAAAAATACCCGGCGTGTGAAATGAAATTCAGCGA
ATTTATCGCCCTTCTGCCAAGCATACGCCCGCGCTATTACTCGATTTCTTCATCACCTCGTGTCGATGAA
AAACAAGCAAGCATCACGGTCAGCGTTGTCTCAGGAGAAGCGTGGAGCGGATATGGAGAATATAAAGGAA
TTGCGTCGAACTATCTTGCCGAGCTGCAAGAAGGAGATACGATTACGTGCTTTATTTCCACACCGCAGTC
AGAATTTACGCTGCCAAAAGACCCTGAAACGCCGCTTATCATGGTCGGACCGGGAACAGGCGTCGCGCCG
TTTAGAGGCTTTGTGCAGGCGCGCAAACAGCTAAAAGAACAAGGACAGTCACTTGGAGAAGCACATTTAT
ACTTCGGCTGCCGTTCACCTCATGAAGACTATCTGTATCAAGAAGAGCTCGAAAACGCCCAAAGCGAAGG
CATCATTACGCTTCATACCGCTTTTTCTCGCATGCCAAATCAGCCGAAAACATACGTTCAGCACGTAATG
GAACAAGACGGCAAGAAATTGATTGAACTTCTTGATCAAGGAGCGCACTTCTATATTTGCGGAGACGGAA
GCCAAATGGCACCTGCCGTTGAAGCAACGCTTATGAAAAGCTATGCTGACGTTCACCAAGTGAGTGAAGC
AGACGCTCGCTTATGGCTGCAGCAGCTAGAAGAAAAAGGCCGATACGCAAAAGACGTGTGGGCTGGGCTC
GAGCACCACCACCACCACCACTGA

MTIKEMPQPKTFGELKNLPLLNTDKPVQALMKIADELGEIFKFEAPGRVTRYLSSQRLIKEACDESRFDK
NLWAGEKCLRDFLGDGLATSWTHEKNWKKAHNILLPSFSQQAMKGYHAVMVDIAVQLVQKWERLNADEHI
EVSEDMTRLTLDTIGLCGFNYRFNSFYRDQPHPFIISLVRANDEVMNKLQRANPDDPAYDENKRQFQEDI
KVMNDLVDKIIADRKARGEQSDDLLTQMLNGKDPETGEPLDDGNIRYQIITFLYAGVEGTSGLLSFALYF
LVKNPHVLQKVAEEAARVLVDPVPSYKQVKQLKYVGMVLNEALRLWPIVPQFSLYAKEDTVLGGEYPLEK
GDEVMVLIPQLHRDKTVWGDDVEEFRPERFENPSAIPQHAFKPFGNGQRASIGQQFALHEATLVLGMMLK
HFDFEDHTNYELDIKELPTLKPKGFVVKAKSKKIPLGGIPSPSTEQSAKKVRKKAENAHNTPLLVLYGSN
MGTAEGTARDLADIAMSKGFAPQVATLDSHAGNLPREGAVLIVTASYNGHPPDNAKQFVDWLDQASADEV
KGVRYSVFGCGDKNWATTYQKVPAFIDETLAAKGAENIADRGEADASDDFEGTYEEWREHMWSDVAAYFN
LDIENSEDNKSTLSLQFVDSAADMPLAKMHGAFSTNVVASKELQQPGSARSTRHLEIELPKEASYQEGDH
LGVIPRNYEGIVNRVTARFGLDASQQIRLEAEEEKLAHLPLAKTVSVEELLQYVELQDPVTRTQLRAMAA
KTVCPPHKVELEALLEKQAYKEQVLAKRLTMLELLEKYPACEMKFSEFIALLPSIRPRYYSISSSPRVDE
KQASITVSVVSGEAWSGYGEYKGIASNYLAELQEGDTITCFISTPQSEFTLPKDPETPLIMVGPGTGVAP
FRGFVQARKQLKEQGQSLGEAHLYFGCRSPHEDYLYQEELENAQSEGIITLHTAFSRMPNQPKTYVQHVM
EQDGKKLIELLDQGAHFYICGDGSQMAPAVEATLMKSYADVHQVSEADARLWLQQLEEKGRYAKDVWAGL
EHHHHHH

X. Computational Methods

DFT Calculations

A conformational search was conducted using CREST of the XTB program (with the searching parameters -rthr 0.5 -metac -T 8).[12] DFT calculation of each low-energy conformer was performed using Gaussian 09 program package.[13] The structure of each species was submitted for geometry optimization at the level of B3LYP/(SDD for Fe and 6-31G(d) for other atoms) for singlet state and uB3LYP/(SDD for Fe and 6-31G(d) for other atoms) for triplet state, with CPCM solvation model for diethyl ether with the integration grid set to ultrafine level, followed by frequency calculation at the same theoretical level. All reported Gibbs free energies are for 298K and are after quasi-harmonic correction using the GoodVibes program.[14] Single point energy calculations of the optimized geometries were performed at uB3LYP-D3/DEF2TZVP level with CPCM solvation model for diethyl ether and with the integration grid set to ultrafine level. We have calculated energies for singlet, triplet, and quintet. All reported energies are in the lowest energy states of the corresponding species.

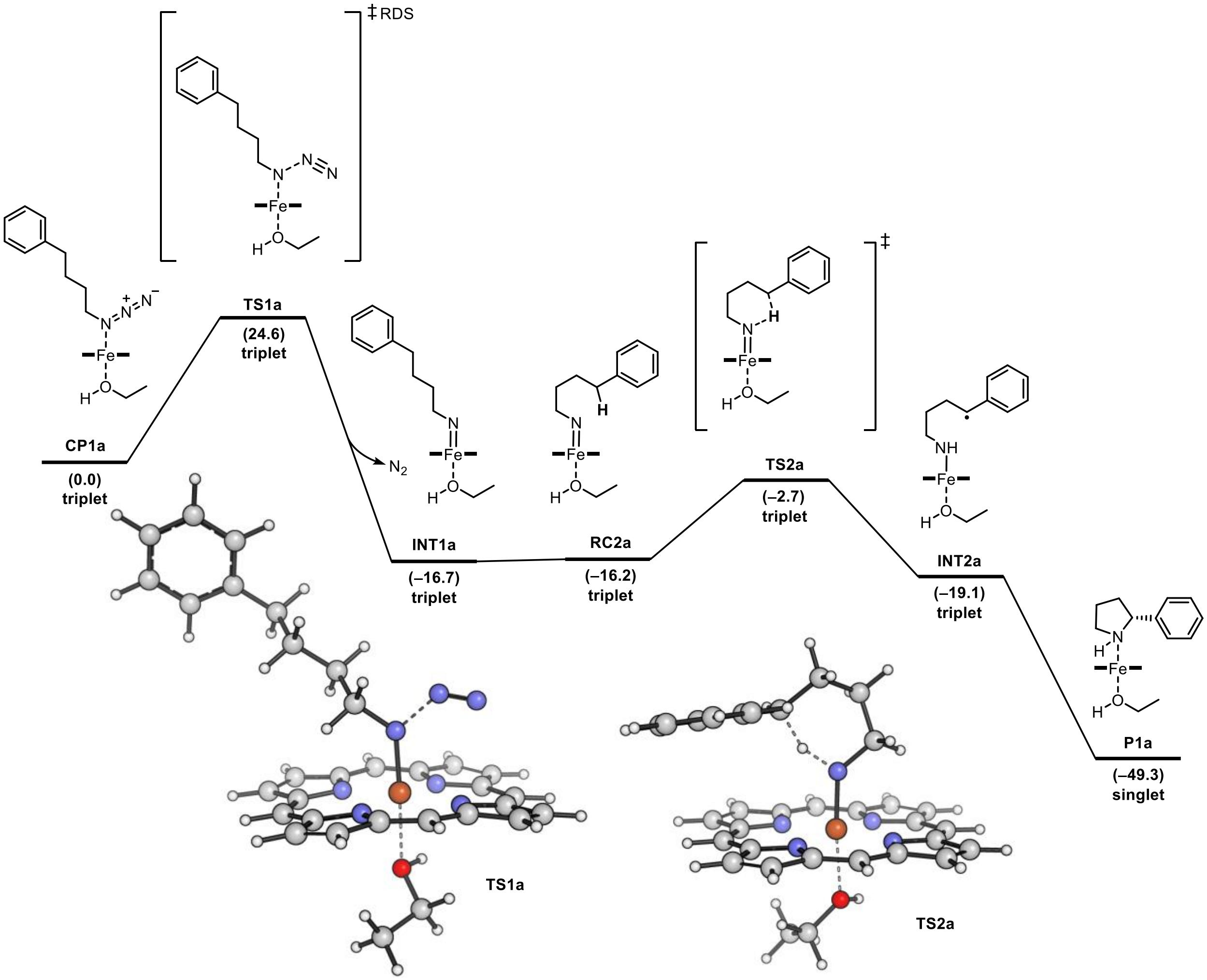

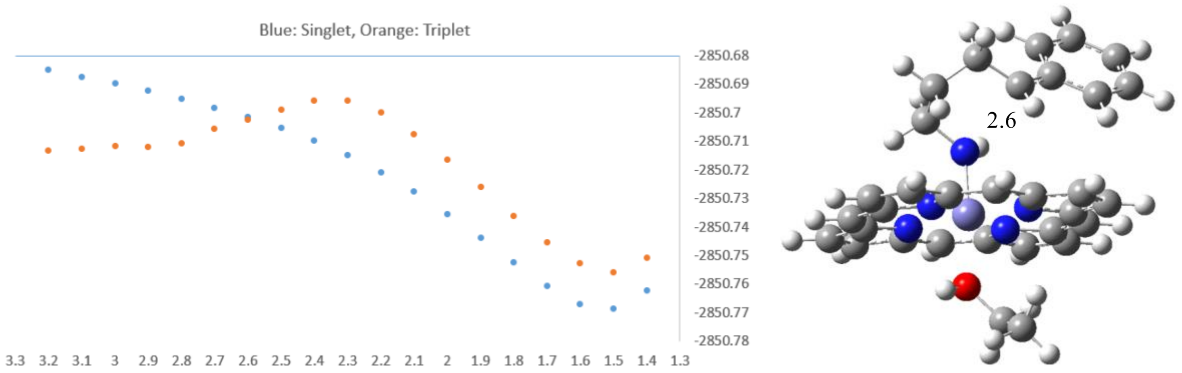

Figure S8. Calculated reaction pathway for the alkyl azide substrate **1a**. The MECP for triplet **INT2a** to singlet **P1a** occurs when the bond forming distance is 2.6 Angstrom.

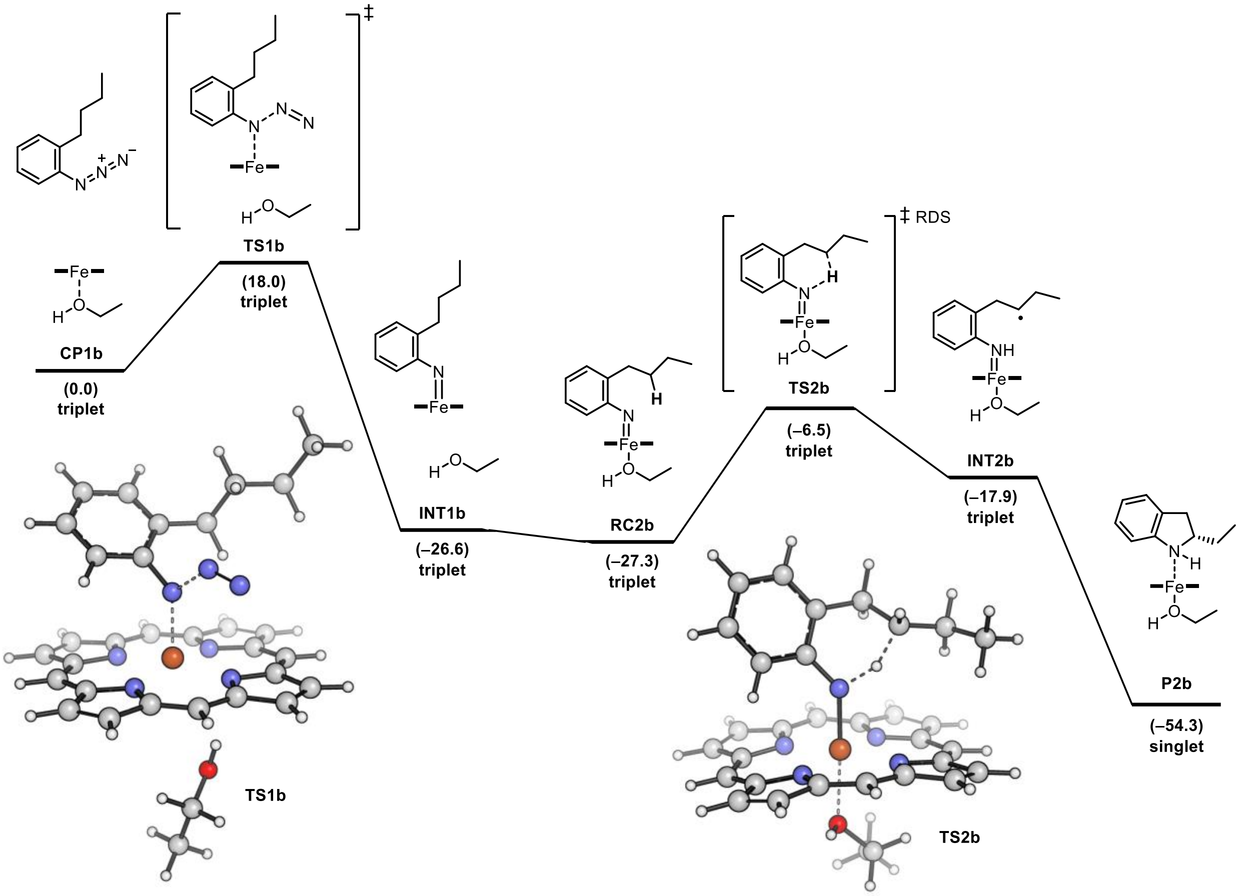

Figure S9. Calculated reaction pathway for the aryl azide substrate **3b**.

Molecular Dynamics Simulations

Molecular dynamics (MD) simulations were performed using the Amber 16 program and AmberTools 16 packages.[15] For the MD simulations of proteins with substrates, the structures resulting from the docking calculations were used for the simulations (where the intrinsically preferred antipode led to docked poses, whereas the opposite antipode does not provide any reasonable docking pose). The protonation state of each protein was first determined from the

PDB2PQR Server using PROPKA[16] to assign protonation states at pH 7.3. MD simulations in explicit water were performed using the GPU-accelerated code (*pmemd*). For the protein scaffold, an evolved version of the Stony Brook modification of the Amber 99 force field (*ff14SB*)[17] parameters were applied. TIP3P[18] parameters were assigned to water molecules. MCPB.py[19] was applied to prepare for the force field of the ligand and Fe atom. The partial charges of the ligand were set to fit the electrostatic potential generated at the HF/6-31 G(d) level by the RESP (restrained electrostatic potential) model.[20] The charges were calculated according to the Merz-Singh-Kollman scheme using the Gaussian 09 program package. For the simulations involving a transition state bound, the two bond-forming distances of the ligand were restrained to the distances according to the QM optimized transition state structure, respectively, applying a harmonic constraint with a force constant with the number corresponding to the imaginary frequency obtained from DFT calculations. For the simulations not involving a transition state bound, no constraint was applied. Each protein complex was placed in a pre-equilibrated cubic box with a 12-Å buffer of TIP3P water molecules using the *leap* module. The systems were neutralized by the addition of explicit counter ions (Na^+). Long-range electrostatic effects were modeled using the particle mesh Ewald method with periodic boundary conditions.[21] An 8-Å cutoff was applied to Lennard-Jones and electrostatic interactions. Molecular dynamics simulations were performed according to the following steps:
(1) Each system was minimized with a maximum cycle of 50000 and with the steepest descent algorithm for the first 25000 cycles, with a periodic boundary for constant volume (NVT) and without the SHAKE algorithm activated. Positional restraint of 2 kcal mol^{-1} $Å^{-2}$ was applied on the protein backbone atoms (C and N) and heavy atoms of the ligand. (2) A 1 ns heating process was performed with a periodic boundary for constant volume (NVT) with the SHAKE algorithm turned on such that the angle between the hydrogen atoms was kept fixed. The temperature increased from 0 K to 300 K in a time period of 1 ns with Langevin dynamics with the collision frequency of 5 ps^{-1}. Positional restraint of 2 kcal mol^{-1} $Å^{-2}$ was applied on the protein backbone atoms (C and N) and heavy atoms of the ligand. (3) a 2 ns equilibrium process was performed with a periodic boundary for constant pressure (NPT) and with a constant temperature of 300 K. Positional restraint of 2 kcal mol^{-1} $Å^{-2}$ was applied on the protein backbone atoms (C and N) and heavy atoms of the ligand. (4) a 2 ns equilibrium process was performed for constant pressure (NPT) and with a constant temperature of 300 K. Positional restraint of 0.5 kcal mol^{-1} $Å^{-2}$ was applied on the protein backbone atoms (C and N), and heavy atoms of the ligand. (5) A 1000 ns production was performed by applying the standard simulation condition for constant pressure (NPT) and with a constant temperature of 300K.

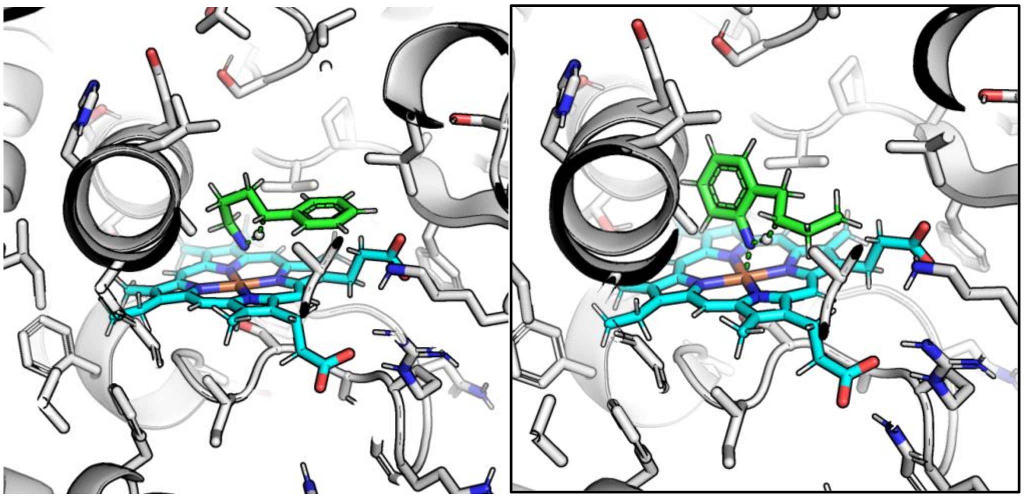

Figure S10. Reaction model of the hydrogen abstraction step derived from MD simulations.

XYZ coordinates and energies for the DFT calculations

**CP1a**			
E[M06-2X/6-31G(d,p)/CPCM(ether)]= -1820.51428283			
Zero-point correction= 0.575950			
Thermal correction to Energy= 0.614033			
Thermal correction to Enthalpy= 0.614978			
Thermal correction to Gibbs Free Energy= 0.495862			
Fe	0.398231	0.471892	0.434531
N	1.758604	1.927010	0.148921
N	1.239127	-0.591553	-1.057537
N	-0.410546	1.502004	1.966395
N	-0.913242	-1.031899	0.779858
C	1.853546	3.110522	0.842744
C	0.837109	-1.823878	-1.520222
C	2.778877	1.951030	-0.776262
C	2.328984	-0.240524	-1.823588
C	-0.020337	2.741315	2.416989
C	-1.039820	-2.196365	0.051618
C	-1.460871	1.122756	2.773501
C	-1.918756	-1.068271	1.725364
C	2.954467	3.899251	0.339520
C	1.689902	-2.254009	-2.604240
C	3.530270	3.178965	-0.663187
C	2.617145	-1.273308	-2.790305
C	-0.844872	3.152722	3.529687
C	-2.139706	-2.981863	0.556154
C	-1.737897	2.148346	3.751901
C	-2.683316	-2.283712	1.594612

H	3.238619	4.873012	0.718594
H	1.581566	-3.189010	-3.139751
H	4.382591	3.439426	-1.278010
H	3.424774	-1.236209	-3.510491
H	-0.738302	4.089527	4.062317
H	-2.444797	-3.941831	0.158451
H	-2.515469	2.090408	4.503166
H	-3.523619	-2.554460	2.221537
C	1.024802	3.495671	1.892230
C	-0.226597	-2.566599	-1.015593
C	-2.167558	-0.070916	2.663127
C	3.050459	0.941663	-1.693960
H	1.219631	4.460699	2.350604
H	-0.434888	-3.520991	-1.490173
H	-2.984198	-0.233895	3.360076
H	3.888789	1.091523	-2.367614
N	-0.812984	1.846132	-2.313686
N	-0.292833	2.077341	-3.303635
C	-5.859108	-0.368053	-1.961537
H	-5.706282	-0.337124	-3.048265
H	-5.487648	-1.342214	-1.617890
C	-5.014795	0.744592	-1.306740
H	-5.384744	1.722694	-1.643573
C	-2.693592	1.717165	-0.958267
H	-3.015349	2.710125	-1.297283
H	-5.162834	0.713689	-0.218623
H	-2.803275	1.677184	0.126868
C	-3.519796	0.614206	-1.626384
H	-3.147731	-0.360151	-1.286670
H	-3.370070	0.653926	-2.714315
N	-1.232379	1.563896	-1.186411
O	1.785157	-1.007076	2.278405
C	2.852538	-1.911255	1.961180
H	2.758595	-2.258960	0.923630
H	2.805050	-2.790912	2.619643
H	0.937854	-1.451560	2.102572
C	4.170793	-1.180918	2.153785
H	5.010736	-1.850521	1.937969
H	4.268641	-0.825164	3.185378
H	4.234424	-0.317794	1.482257
C	-7.336084	-0.245453	-1.650310
C	-7.887334	-0.866934	-0.520190
C	-8.181592	0.521760	-2.465185
C	-9.241868	-0.725304	-0.210418

H	-7.248500	-1.471676	0.120262
C	-9.536764	0.666982	-2.160282
H	-7.773765	1.006086	-3.350124
C	-10.071980	0.043567	-1.030134
H	-9.649439	-1.219068	0.668071
H	-10.175090	1.262810	-2.807711
H	-11.126918	0.152263	-0.792997

CP1b

E[M06-2X/6-31G(d,p)/CPCM(ether)]= -1820.51559468
Zero-point correction= 0.574840
Thermal correction to Energy= 0.613114
Thermal correction to Enthalpy= 0.614058
Thermal correction to Gibbs Free Energy= 0.495698

Fe	0.384426	0.145536	0.734914
N	1.255724	1.944407	0.510569
N	1.363161	-0.524411	-0.893477
N	-0.702617	0.853820	2.275452
N	-0.581265	-1.624159	0.886582
C	1.076436	3.054081	1.304906
C	1.286595	-1.783165	-1.445911
C	2.178371	2.313289	-0.445029
C	2.272491	0.167398	-1.664146
C	-0.618072	2.107506	2.836278
C	-0.406705	-2.733429	0.086503
C	-1.649242	0.177541	3.014278
C	-1.558215	-1.970509	1.798008
C	1.906354	4.141332	0.842224
C	2.168346	-1.884900	-2.584871
C	2.589052	3.682501	-0.244325
C	2.779374	-0.674615	-2.720896
C	-1.528303	2.221256	3.951610
C	-1.281834	-3.798969	0.511805
C	-2.169726	1.024649	4.060963
C	-1.996838	-3.325722	1.571608
H	1.947082	5.122427	1.298730
H	2.289079	-2.772671	-3.193145
H	3.305858	4.208304	-0.862470
H	3.504117	-0.364164	-3.463013
H	-1.651157	3.109694	4.558376
H	-1.335490	-4.774954	0.045674
H	-2.926806	0.727224	4.775548

H	-2.755925	-3.833348	2.153344
C	0.212290	3.131452	2.392674
C	0.471323	-2.814364	-0.989438
C	-2.051364	-1.135450	2.794513
C	2.655236	1.488259	-1.457754
H	0.174318	4.072865	2.932571
H	0.509953	-3.755368	-1.530139
H	-2.814857	-1.540084	3.452099
H	3.384432	1.909617	-2.143231
N	-2.955514	1.786966	-0.634009
N	-3.475239	2.259410	0.266669
N	-2.407241	1.107696	-1.506204
O	1.991669	-0.897686	2.298337
C	3.346859	-1.269750	1.984844
H	3.408121	-1.604077	0.941225
H	3.653172	-2.102844	2.631411
C	4.243350	-0.065582	2.213032
H	5.283941	-0.325041	1.988585
H	4.186785	0.266968	3.255295
H	3.946461	0.765580	1.565563
C	-2.055777	1.656252	-2.780078
C	-1.355332	2.427015	-5.371877
C	-1.541709	2.954525	-2.985048
C	-2.182067	0.754741	-3.846890
C	-1.837892	1.136768	-5.139977
C	-1.210048	3.308801	-4.302142
H	-2.562727	-0.240646	-3.640433
H	-1.947116	0.431356	-5.958606
H	-0.810406	4.304774	-4.479855
H	-1.080259	2.739981	-6.375015
C	-1.320339	3.960439	-1.873701
H	-0.398052	4.513214	-2.092435
H	-1.137649	3.451892	-0.920630
C	-2.469016	4.978801	-1.711014
H	-3.412208	4.450713	-1.517460
H	-2.608081	5.512373	-2.661597
C	-2.210353	5.989097	-0.586091
H	-1.263701	6.512743	-0.781142
H	-2.071144	5.446658	0.359607
H	1.434632	-1.693365	2.260417
C	-3.340031	7.012564	-0.429654
H	-4.292183	6.518631	-0.199882
H	-3.127424	7.720267	0.379910
H	-3.480357	7.591342	-1.351067

INT1a			
E[M06-2X/6-31G(d,p)/CPCM(ether)]= -1710.99957173			
Zero-point correction= 0.566329			
Thermal correction to Energy= 0.601098			
Thermal correction to Enthalpy= 0.602043			
Thermal correction to Gibbs Free Energy= 0.494821			
Fe	0.350852	0.151080	0.425007
N	1.061442	1.817449	-0.529137
N	1.471098	-1.004837	-0.818457
N	-0.497009	1.338082	1.805542
N	-0.072763	-1.467199	1.529698
C	0.731625	3.126617	-0.260773
C	1.579683	-2.372077	-0.794465
C	1.802132	1.845276	-1.688987
C	2.171526	-0.589764	-1.922811
C	-0.601395	2.710501	1.776452
C	0.245957	-2.774304	1.244155
C	-1.201303	0.927778	2.914345
C	-0.827397	-1.502221	2.677869
C	1.295182	4.000415	-1.262034
C	2.375679	-2.834131	-1.909762
C	1.959103	3.205494	-2.147717
C	2.743023	-1.726873	-2.610991
C	-1.382560	3.173635	2.898959
C	-0.316957	-3.653241	2.242660
C	-1.753440	2.069145	3.604652
C	-0.982298	-2.864726	3.131932
H	1.187888	5.077835	-1.271787
H	2.613142	-3.871260	-2.110749
H	2.508379	3.496869	-3.034229
H	3.345179	-1.664650	-3.508796
H	-1.604104	4.213002	3.106449
H	-0.202683	-4.729925	2.248956
H	-2.342630	2.013909	4.511446
H	-1.527732	-3.159558	4.019612
C	-0.037693	3.546733	0.818335
C	1.009865	-3.202215	0.164226
C	-1.358814	-0.391283	3.324420
C	2.324645	0.730391	-2.335119
H	-0.215533	4.612858	0.921663
H	1.177961	-4.270360	0.065357

H	-1.939082	-0.567852	4.224979
H	2.900633	0.904830	-3.239003
C	-5.781002	-0.616728	-2.241241
H	-5.640410	0.232635	-2.922563
H	-5.451819	-1.512998	-2.783214
C	-4.877441	-0.420641	-1.006412
H	-5.204980	0.475511	-0.461023
C	-2.498950	-0.097446	-0.128886
H	-2.827438	0.802234	0.420784
H	-5.018597	-1.267985	-0.320929
H	-2.640295	-0.948146	0.559939
C	-3.393122	-0.291384	-1.369494
H	-3.061361	-1.188279	-1.909430
H	-3.247211	0.558640	-2.049379
N	-1.122212	0.025542	-0.490115
O	2.368370	0.432244	1.585157
C	2.572706	0.573569	3.007924
H	1.665313	0.981738	3.467539
H	3.393025	1.281849	3.176407
H	2.474364	1.301998	1.162034
C	2.917261	-0.783595	3.596274
H	3.070464	-0.691820	4.677562
H	3.835926	-1.178136	3.148835
H	2.109174	-1.500051	3.421831
C	-7.246990	-0.746704	-1.884222
C	-8.068543	0.386478	-1.791037
C	-7.810910	-1.999770	-1.602079
C	-9.411712	0.273644	-1.425063
H	-7.651051	1.366676	-2.012248
C	-9.153399	-2.118834	-1.235623
H	-7.191402	-2.891334	-1.675039
C	-9.959348	-0.981017	-1.145277
H	-10.031219	1.164772	-1.363062
H	-9.570745	-3.100340	-1.025333
H	-11.005092	-1.071644	-0.863794

INT1b

E[M06-2X/6-31G(d,p)/CPCM(ether)]= -1711.01292976
Zero-point correction= 0.565376
Thermal correction to Energy= 0.600836
Thermal correction to Enthalpy= 0.601781
Thermal correction to Gibbs Free Energy= 0.492742

Fe	0.798039	1.359037	-0.028810
N	2.496994	2.360066	-0.355661
N	1.382009	-0.048803	-1.329699
N	0.504028	2.487562	1.639396
N	-0.616973	0.051963	0.665665
C	2.889336	3.547120	0.229815
C	0.690507	-1.187699	-1.691335
C	3.465602	2.075432	-1.295513
C	2.498039	-0.023941	-2.138993
C	1.148354	3.658120	1.963876
C	-1.076195	-1.076681	0.018404
C	-0.513013	2.360218	2.559142
C	-1.505038	0.269931	1.699663
C	4.112297	4.021948	-0.367996
C	1.379149	-1.877621	-2.754252
C	4.475564	3.103944	-1.305494
C	2.505542	-1.162663	-3.023716
C	0.536099	4.273119	3.117169
C	-2.253743	-1.589382	0.670970
C	-0.492696	3.463803	3.490405
C	-2.516290	-0.757144	1.718785
H	4.625085	4.929987	-0.077463
H	1.040253	-2.800749	-3.206895
H	5.345888	3.103410	-1.949284
H	3.282562	-1.372718	-3.747600
H	0.869473	5.196924	3.572675
H	-2.792571	-2.476253	0.362646
H	-1.183160	3.584985	4.315474
H	-3.316351	-0.819712	2.445455
C	2.250100	4.170286	1.291784
C	-0.470996	-1.652345	-1.090732
C	-1.448632	1.333909	2.590901
C	3.477818	0.959213	-2.120802
H	2.669702	5.104838	1.650746
H	-0.917677	-2.554943	-1.495819
H	-2.204806	1.372666	3.369023
H	4.308341	0.854145	-2.811675
N	-0.249871	2.266870	-1.072772
O	1.882506	-1.145173	2.165330
C	1.819875	-1.061666	3.590640
H	1.031634	-1.724592	3.979936
H	1.576756	-0.036562	3.907584
C	3.170227	-1.475175	4.153271
H	3.158227	-1.433643	5.248038

H	3.959502	-0.807823	3.789669
H	3.417307	-2.498090	3.848292
C	-0.384827	2.906300	-2.243737
C	-0.785738	4.274266	-4.687009
C	-1.581187	3.690405	-2.450799
C	0.578809	2.842603	-3.296183
C	0.375864	3.516278	-4.491169
C	-1.742620	4.349077	-3.666574
H	1.474383	2.256072	-3.140814
H	1.125073	3.453257	-5.276190
H	-2.640779	4.943774	-3.819920
H	-0.944746	4.804806	-5.621432
C	-2.644719	3.768867	-1.381135
H	-3.240010	4.678027	-1.536375
H	-2.171060	3.857173	-0.395952
C	-3.591375	2.549580	-1.366632
H	-2.996690	1.636713	-1.233189
H	-4.083215	2.464369	-2.346129
C	-4.653996	2.633405	-0.263603
H	-5.241134	3.553997	-0.392745
H	-4.153121	2.722247	0.710947
H	1.031186	-0.844820	1.801674
C	-5.594148	1.423180	-0.244488
H	-6.345932	1.513455	0.548447
H	-6.126219	1.319399	-1.198427
H	-5.035309	0.495123	-0.071864

INT2a

E[M06-2X/6-31G(d,p)/CPCM(ether)]= -1710.99829055

Zero-point correction= 0.565464

Thermal correction to Energy= 0.600051

Thermal correction to Enthalpy= 0.600995

Thermal correction to Gibbs Free Energy= 0.495476

N	-2.112991	23.301363	-28.790109
C	-3.118253	22.256637	-28.815395
C	-2.815304	21.136593	-29.830413
C	-1.609679	20.237523	-29.478909
C	-0.272067	20.892529	-29.644284
O	-2.576940	27.017087	-27.334655
H	-3.426271	27.342246	-27.677051
N	-0.766257	24.743747	-26.871507
N	-3.628050	24.458580	-26.729969

N	-0.995661	25.745808	-29.513620
C	1.107589	25.466882	-28.283790
C	-2.031759	23.872309	-24.953880
C	-5.741345	24.811318	-27.929977
C	-2.587873	26.248990	-31.318790
C	0.570603	24.960712	-27.103701
C	-3.315943	23.989170	-25.478387
C	-5.205337	25.286709	-29.123500
C	-1.305164	26.164579	-30.783893
C	1.347724	24.578724	-25.945861
C	-4.522643	23.648974	-24.757423
C	-5.979053	25.661862	-30.284535
C	-0.099690	26.527750	-31.497221
C	0.465135	24.123990	-25.014023
C	-5.566564	23.920959	-25.588441
C	-5.089054	26.056995	-31.237675
C	0.940072	26.318821	-30.642579
C	-0.852504	24.229292	-25.600901
C	-4.998358	24.424213	-26.818786
C	-3.770774	25.927544	-30.658856
C	0.370158	25.824626	-29.408881
N	-3.863430	25.459538	-29.369722
Fe	-2.306646	24.997162	-28.157029
H	-4.078790	22.709910	-29.079867
H	-3.256651	21.792374	-27.820370
H	-2.667007	21.580369	-30.824421
H	-3.706133	20.497377	-29.898055
H	-1.654371	19.351703	-30.137326
H	-1.738738	19.851971	-28.459466
H	-1.185919	22.921305	-28.599654
H	-0.155065	21.528910	-30.520788
H	2.186269	25.581018	-28.334001
H	-1.943899	23.482511	-23.943953
H	-6.821997	24.727292	-27.864036
H	-2.674057	26.605940	-32.341033
H	-6.626691	23.801519	-25.402589
H	-7.059570	25.622985	-30.345183
H	-5.289473	26.408962	-32.242052
H	-0.072598	26.894107	-32.515985
H	1.997520	26.477032	-30.814352
H	2.425458	24.655025	-25.874287
H	0.668257	23.750831	-24.017959
H	-4.547756	23.259927	-23.747119
C	-1.718201	29.095769	-28.360859

H	-0.998097	29.907301	-28.204648
H	-1.486977	28.601333	-29.308465
H	-2.715207	29.548617	-28.435413
C	-1.649528	28.114829	-27.200082
H	-1.856812	28.623755	-26.250052
H	-0.665653	27.648213	-27.130891
C	0.876137	20.710588	-28.832623
C	2.085342	21.399868	-29.157417
C	0.907640	19.860170	-27.686239
C	3.232888	21.250626	-28.393482
H	2.092394	22.055150	-30.025373
C	2.063455	19.717861	-26.929555
H	0.016669	19.309233	-27.402105
C	3.235448	20.407747	-27.271197
H	4.136020	21.789577	-28.668748
H	2.056870	19.060847	-26.063328
H	4.135385	20.290221	-26.674397

INT2b

E[M06-2X/6-31G(d,p)/CPCM(ether)]= -1710.99422569
Zero-point correction= 0.564434
Thermal correction to Energy= 0.599336
Thermal correction to Enthalpy= 0.600280
Thermal correction to Gibbs Free Energy= 0.496860

Fe	0.608653	0.502097	0.267943
N	2.257584	1.597183	-0.061896
N	1.223090	-0.805456	-1.141506
N	0.111133	1.672213	1.841079
N	-0.929898	-0.739622	0.757636
C	2.577565	2.782706	0.552635
C	0.535593	-1.912804	-1.578099
C	3.272018	1.345508	-0.954372
C	2.372263	-0.750304	-1.896323
C	0.720923	2.852463	2.187808
C	-1.336204	-1.843643	0.041748
C	-0.941094	1.516323	2.708769
C	-1.860494	-0.571075	1.757910
C	3.820872	3.298638	0.025146
C	1.265743	-2.565735	-2.640876
C	4.256166	2.401987	-0.902998
C	2.409546	-1.850862	-2.830949
C	0.035400	3.457394	3.308440

C	-2.544144	-2.389543	0.613897
C	-0.991613	2.625214	3.635946
C	-2.864934	-1.605691	1.682361
H	4.290525	4.220320	0.345095
H	0.936496	-3.460100	-3.154886
H	5.154889	2.436917	-1.505904
H	3.210670	-2.034323	-3.535921
H	0.321885	4.391738	3.774686
H	-3.063117	-3.263233	0.239981
H	-1.724591	2.735478	4.425291
H	-3.701101	-1.704174	2.363220
C	1.854862	3.381780	1.580254
C	-0.663624	-2.383467	-1.050030
C	-1.856752	0.467735	2.683942
C	3.342991	0.241674	-1.799026
H	2.231739	4.323698	1.967427
H	-1.092965	-3.269189	-1.508609
H	-2.648901	0.478535	3.426342
H	4.204680	0.166999	-2.455350
N	-0.520196	1.512391	-0.796246
O	1.671873	-0.716801	1.762683
C	2.968801	-1.358871	1.669530
H	3.209655	-1.522254	0.615066
H	2.903130	-2.333432	2.165423
C	4.011472	-0.486239	2.345925
H	4.996032	-0.961870	2.270289
H	3.774109	-0.348150	3.406165
H	4.066282	0.496599	1.869141
C	-0.405678	2.388908	-1.858259
C	-0.231358	4.133931	-4.088125
C	-1.268199	3.526315	-1.958246
C	0.531916	2.171895	-2.893799
C	0.618195	3.029528	-3.986241
C	-1.162376	4.363429	-3.070892
H	1.176146	1.305436	-2.834414
H	1.348076	2.826793	-4.766635
H	-1.822374	5.226605	-3.134688
H	-0.171320	4.806176	-4.939495
C	-2.273064	3.846140	-0.866971
H	-2.675171	4.858228	-1.058215
H	-1.762476	3.927564	0.107089
C	-3.411073	2.871726	-0.743629
H	-1.372325	1.688273	-0.268680
H	-3.738514	2.357709	-1.647180

C	-4.345296	2.919772	0.424413
H	-4.958046	3.841039	0.373607
H	-3.766602	3.016912	1.355979
H	0.976945	-1.395138	1.816792
C	-5.282318	1.707922	0.517566
H	-4.712512	0.780221	0.643355
H	-5.968204	1.803063	1.366964
H	-5.887287	1.609099	-0.391886

P1a

E[M06-2X/6-31G(d,p)/CPCM(ether)]= -1711.05336838

Zero-point correction= 0.572279

Thermal correction to Energy= 0.605238

Thermal correction to Enthalpy= 0.606182

Thermal correction to Gibbs Free Energy= 0.508138

N	-0.479298	23.466083	-29.370636
C	-1.116416	22.801133	-30.558550
C	-0.314385	21.501090	-30.826803
C	0.888969	21.590578	-29.867910
C	0.328480	22.408297	-28.674216
O	-2.143906	27.012356	-27.650766
N	0.547127	25.871634	-28.235337
N	-1.375273	24.562051	-26.562160
N	-1.385131	26.001687	-30.347243
C	0.913329	26.866100	-30.452579
C	0.919408	25.157747	-25.913756
C	-3.684805	23.735646	-26.445459
C	-3.682469	25.415950	-30.988395
C	1.326672	26.517147	-29.167184
C	-0.341319	24.621870	-25.656606
C	-4.075280	24.028774	-27.750961
C	-2.434853	25.986637	-31.233610
C	2.635816	26.797377	-28.617842
C	-0.755029	24.065345	-24.386510
C	-5.401468	23.788089	-28.283533
C	-2.055442	26.634038	-32.471404
C	2.636342	26.318170	-27.342050
C	-2.052038	23.674681	-24.533185
C	-5.399582	24.256374	-29.561983
C	-0.761274	27.034493	-32.324353
C	1.328562	25.742303	-27.111954
C	-2.430557	23.986677	-25.894138

C	-4.073341	24.786153	-29.808035
C	-0.351252	26.635633	-30.994511
N	-3.279018	24.617052	-28.701298
Fe	-1.360438	25.208944	-28.482379
H	-1.083259	23.498891	-31.396253
H	-2.166907	22.604484	-30.342600
H	-0.003982	21.415465	-31.872378
H	-0.922636	20.621414	-30.592278
H	1.637017	27.373990	-31.084249
H	1.642800	25.132162	-25.103298
H	-4.426077	23.274758	-25.798396
H	-4.420537	25.479826	-31.783398
H	-2.704202	23.216578	-23.799631
H	-6.215326	23.330834	-27.734013
H	-6.211649	24.264026	-30.278864
H	-2.705038	26.756980	-33.329493
H	-0.131754	27.554645	-33.036093
H	3.435201	27.299987	-29.148677
H	3.436542	26.347017	-26.612443
H	-0.125850	23.995786	-23.507493
C	-2.365015	29.395628	-27.143967
H	-1.922756	30.380440	-27.334356
H	-3.399508	29.405179	-27.503281
H	-2.377052	29.237922	-26.058521
C	-1.555902	28.316577	-27.848290
H	-0.515756	28.305885	-27.504859
H	-1.554953	28.462312	-28.929650
C	-0.395883	21.497929	-27.694938
C	0.383756	20.871014	-26.707970
C	-1.751191	21.154390	-27.778880
C	-0.165035	19.926096	-25.841364
H	1.438009	21.126249	-26.621910
C	-2.304923	20.206561	-26.912716
H	-2.393357	21.641097	-28.502870
C	-1.516368	19.584455	-25.944552
H	0.461523	19.458890	-25.085933
H	-3.360452	19.960631	-26.996338
H	-1.949211	18.847375	-25.273476
H	1.125776	22.923362	-28.132316
H	0.243010	24.065565	-29.768392
H	1.276320	20.612646	-29.568926
H	1.710903	22.150538	-30.332190
H	-2.071988	26.771726	-26.709862

**P2b**			
E[M06-2X/6-31G(d,p)/CPCM(ether)]= -1711.05390925			
Zero-point correction= 0.570621			
Thermal correction to Energy= 0.603943			
Thermal correction to Enthalpy= 0.604888			
Thermal correction to Gibbs Free Energy= 0.507355			
Fe	0.379977	0.444249	0.402194
N	1.685154	1.914431	-0.025457
N	1.099485	-0.650282	-1.141213
N	-0.214956	1.445068	2.046765
N	-0.873815	-1.079988	0.871035
C	1.801414	3.124436	0.612285
C	0.633240	-1.863154	-1.590122
C	2.645943	1.909863	-1.006721
C	2.169662	-0.334479	-1.943904
C	0.183156	2.697390	2.439268
C	-1.111389	-2.220612	0.135913
C	-1.039612	0.979692	3.040233
C	-1.664408	-1.179947	1.993428
C	2.843341	3.919913	-0.004191
C	1.426780	-2.321498	-2.710453
C	3.375601	3.160752	-1.001456
C	2.390227	-1.379647	-2.919040
C	-0.425873	3.045088	3.707148
C	-2.102334	-3.041696	0.795003
C	-1.173402	1.971938	4.087483
C	-2.434776	-2.402850	1.952906
H	3.128957	4.916147	0.310790
H	1.264747	-3.249568	-3.245013
H	4.184460	3.407556	-1.678247
H	3.174263	-1.373095	-3.666404
H	-0.274915	3.981447	4.230382
H	-2.467259	-3.990600	0.421336
H	-1.770101	1.849619	4.983200
H	-3.133150	-2.718971	2.718194
C	1.086460	3.502859	1.747584
C	-0.426643	-2.574332	-1.026276
C	-1.715271	-0.238920	3.020200
C	2.895122	0.853529	-1.879859
H	1.298967	4.483517	2.164374
H	-0.700588	-3.514151	-1.497441
H	-2.360939	-0.462218	3.865098

H	3.704301	0.979724	-2.593799
N	-1.264076	1.238403	-1.064572
O	1.730624	-0.588704	1.704762
C	3.169617	-0.700975	1.564856
H	3.435370	-0.604635	0.508491
H	3.465906	-1.698002	1.910202
C	3.840335	0.372358	2.403282
H	4.928622	0.297804	2.295751
H	3.588446	0.252840	3.462609
H	3.528666	1.369349	2.079463
C	-0.755986	1.842696	-2.275908
C	0.041532	3.413035	-4.428171
C	-1.000460	3.224113	-2.267161
C	-0.176663	1.224030	-3.378855
C	0.225904	2.028503	-4.453617
C	-0.589082	4.017551	-3.331618
H	-0.045569	0.149007	-3.419234
H	0.681463	1.560351	-5.322362
H	-0.782547	5.087592	-3.328729
H	0.358672	4.018777	-5.272701
C	-1.817444	3.553054	-1.040833
H	-2.585015	4.311436	-1.220939
H	-1.189646	3.892433	-0.204871
C	-2.419853	2.167787	-0.728826
H	-1.617911	0.298662	-1.239449
H	-3.186343	1.974712	-1.497682
C	-3.078395	1.991996	0.632252
H	-2.422932	2.374016	1.415685
H	-3.219926	0.923474	0.830093
H	1.329514	-1.463531	1.570928
C	-4.437092	2.704100	0.700596
H	-4.337308	3.785344	0.548573
H	-5.129651	2.323175	-0.060464
H	-4.901792	2.551017	1.680837

RC2a

E[M06-2X/6-31G(d,p)/CPCM(ether)]= -1710.99898058
Zero-point correction= 0.566426
Thermal correction to Energy= 0.600994
Thermal correction to Enthalpy= 0.601938
Thermal correction to Gibbs Free Energy= 0.495824

N	-2.221773	23.404981	-29.402152

C	-3.230321	22.393327	-29.399256
C	-2.730665	21.074100	-30.027097
C	-1.543698	20.417509	-29.305986
C	-1.856530	19.937657	-27.872357
O	-2.239508	27.177436	-28.059422
H	-1.982523	27.716344	-28.826163
N	-0.316512	24.849912	-27.949450
N	-2.987802	24.515711	-27.038393
N	-1.305792	25.854403	-30.454189
C	1.061211	25.601839	-29.840793
C	-0.962634	23.964355	-25.756853
C	-5.355326	24.801674	-27.633114
C	-3.325701	26.296703	-31.773075
C	0.890175	25.085541	-28.559653
C	-2.341875	24.056365	-25.914041
C	-5.182735	25.270554	-28.930919
C	-1.946122	26.259807	-31.600891
C	1.976905	24.723750	-27.676075
C	-3.304896	23.697971	-24.898759
C	-6.264187	25.625672	-29.821763
C	-0.981111	26.642592	-32.605580
C	1.410770	24.259723	-26.527559
C	-4.538307	23.945105	-25.421242
C	-5.693787	26.033753	-30.988797
C	0.251218	26.453062	-32.056887
C	-0.021410	24.340666	-26.710159
C	-4.331852	24.448861	-26.759300
C	-4.262762	25.934680	-30.810929
C	0.039718	25.951370	-30.717963
N	-3.973369	25.465911	-29.552023
Fe	-2.160528	25.037488	-28.791710
H	-4.111684	22.741943	-29.965126
H	-3.585363	22.211336	-28.371185
H	-2.447852	21.273288	-31.068835
H	-3.578487	20.374787	-30.058088
H	-0.703287	21.122442	-29.274040
H	-1.204901	19.555888	-29.896567
H	-2.722340	19.262499	-27.903104
H	-2.146428	20.796006	-27.253998
H	2.081025	25.732906	-30.190325
H	-0.592697	23.580696	-24.810812
H	-6.374388	24.697192	-27.273051
H	-3.702097	26.646282	-32.729704
H	-5.507423	23.805506	-24.958834

H	-7.315247	25.567081	-29.568076
H	-6.178718	26.382092	-31.892080
H	-1.231145	27.006109	-33.594451
H	1.222319	26.627065	-32.503344
H	3.027632	24.819906	-27.919680
H	1.899605	23.898088	-25.631589
H	-3.051226	23.315549	-23.918024
C	-0.349435	28.357728	-26.985710
H	-0.035619	28.809210	-26.037405
H	0.367634	27.577039	-27.254690
H	-0.316279	29.142591	-27.752239
C	-1.753842	27.788498	-26.847249
H	-2.460389	28.573734	-26.547979
H	-1.788024	26.996327	-26.096823
C	-0.683342	19.229763	-27.226953
C	-0.555641	17.834717	-27.294857
C	0.323930	19.957161	-26.574609
C	0.543780	17.183455	-26.730236
H	-1.328991	17.252813	-27.792216
C	1.425520	19.311306	-26.009312
H	0.240742	21.040118	-26.507142
C	1.539773	17.920488	-26.084983
H	0.619814	16.100685	-26.791105
H	2.192983	19.894039	-25.505898
H	2.394672	17.416114	-25.642374

RC2b

E[M06-2X/6-31G(d,p)/CPCM(ether)]= -1711.01513944
Zero-point correction= 0.565739
Thermal correction to Energy= 0.600543
Thermal correction to Enthalpy= 0.601487
Thermal correction to Gibbs Free Energy= 0.497100

Fe	0.786754	0.745042	0.382055
N	2.569022	1.604411	0.052759
N	1.143164	-0.588652	-1.087902
N	0.547975	1.898892	2.038420
N	-0.871468	-0.335526	0.911561
C	3.100057	2.685519	0.716185
C	0.310767	-1.596472	-1.517316
C	3.483276	1.264646	-0.915639
C	2.244476	-0.629712	-1.910564
C	1.340297	2.945272	2.431154

C	-1.449281	-1.362807	0.202774
C	-0.500079	1.861438	2.921852
C	-1.734295	-0.052511	1.944449
C	4.378771	3.040376	0.144290
C	0.898773	-2.280463	-2.646359
C	4.619776	2.155037	-0.862336
C	2.100504	-1.686435	-2.885391
C	0.781001	3.588000	3.600984
C	-2.696402	-1.753259	0.817155
C	-0.359965	2.912473	3.907989
C	-2.870809	-0.943583	1.900501
H	5.002437	3.855196	0.489997
H	0.442557	-3.113394	-3.166502
H	5.480233	2.094950	-1.516895
H	2.833812	-1.928103	-3.644552
H	1.221402	4.436710	4.109140
H	-3.339713	-2.547041	0.458586
H	-1.053534	3.092365	4.719910
H	-3.687897	-0.936326	2.610989
C	2.526058	3.324605	1.810272
C	-0.902189	-1.949780	-0.934159
C	-1.558244	0.957193	2.886294
C	3.342481	0.221238	-1.826105
H	3.061705	4.171203	2.229158
H	-1.455668	-2.764122	-1.391972
H	-2.320292	1.056154	3.653729
H	4.147750	0.069121	-2.538432
N	-0.109743	1.857245	-0.634967
O	1.744478	-0.715759	1.811570
C	2.947997	-1.499794	1.630327
H	3.136556	-1.626403	0.559346
H	2.784979	-2.489585	2.071808
C	4.109598	-0.799195	2.313161
H	5.027689	-1.380959	2.171944
H	3.924900	-0.699136	3.388113
H	4.265055	0.198066	1.891559
C	-0.226131	2.590591	-1.745789
C	-0.537901	4.173015	-4.073794
C	-1.342985	3.502296	-1.865073
C	0.706080	2.514361	-2.829557
C	0.545851	3.291069	-3.966457
C	-1.461204	4.264426	-3.023964
H	1.542991	1.834382	-2.740325
H	1.268553	3.214628	-4.774989

H	-2.298188	4.954676	-3.108981
H	-0.662209	4.785756	-4.962143
C	-2.377167	3.603039	-0.768993
H	-2.843968	4.596034	-0.809383
H	-1.890385	3.512927	0.209024
C	-3.482974	2.530889	-0.873512
H	-3.021370	1.535675	-0.834683
H	-3.971425	2.613018	-1.855047
C	-4.536418	2.651302	0.234916
H	-4.984603	3.654838	0.202443
H	-4.040250	2.567009	1.212019
H	0.971389	-1.305676	1.794715
C	-5.640102	1.593025	0.130415
H	-5.221766	0.581804	0.205000
H	-6.381577	1.708266	0.929875
H	-6.169160	1.665415	-0.828154

TS1a

E[M06-2X/6-31G(d,p)/CPCM(ether)]= -1820.47131100
Zero-point correction= 0.574028
Thermal correction to Energy= 0.610724
Thermal correction to Enthalpy= 0.611668
Thermal correction to Gibbs Free Energy= 0.501557

Fe	0.602248	0.899615	0.650682
N	2.276997	1.800920	1.362247
N	1.728081	-0.185175	-0.617423
N	-0.447485	1.786858	2.129557
N	-0.992118	-0.225822	0.181386
C	2.386741	2.658158	2.436419
C	1.320562	-1.229928	-1.410765
C	3.525227	1.761106	0.780542
C	3.052495	0.026592	-0.915654
C	0.017207	2.628486	3.113360
C	-1.047399	-1.266122	-0.715987
C	-1.808284	1.709647	2.309499
C	-2.279096	-0.033964	0.623988
C	3.734866	3.160196	2.536037
C	2.416366	-1.689272	-2.232448
C	4.436263	2.617432	1.500623
C	3.487796	-0.903297	-1.933072
C	-1.077989	3.091722	3.932598
C	-2.402431	-1.752161	-0.830586

C	-2.212500	2.535799	3.423647
C	-3.168012	-0.980480	-0.008884
H	4.081812	3.845945	3.298680
H	2.355432	-2.506727	-2.939824
H	5.478772	2.760261	1.245345
H	4.489979	-0.944670	-2.340799
H	-0.974904	3.762114	4.776543
H	-2.710754	-2.573064	-1.465696
H	-3.232541	2.647702	3.769146
H	-4.233533	-1.041245	0.173626
C	1.342588	3.013555	3.282465
C	0.031917	-1.752649	-1.446861
C	-2.669588	0.888725	1.588918
C	3.882824	0.954242	-0.294392
H	1.568094	3.689579	4.101408
H	-0.154459	-2.580357	-2.124250
H	-3.724436	0.927422	1.843302
H	4.910159	1.013017	-0.640818
N	0.190468	3.756522	-0.407423
N	0.478678	4.343733	0.535013
C	1.498448	2.457953	-5.534200
H	1.737188	3.522856	-5.413750
H	2.422761	1.902636	-5.327408
C	0.436336	2.064048	-4.486771
H	-0.491268	2.616735	-4.692374
C	-0.179681	1.946013	-2.012533
H	-1.102207	2.510166	-2.215657
H	0.193885	0.998691	-4.606898
H	-0.428176	0.887621	-2.119701
C	0.892060	2.330376	-3.046543
H	1.809053	1.766818	-2.834527
H	1.136939	3.394502	-2.927044
N	0.218222	2.143913	-0.624297
O	1.253086	-0.723308	2.193337
C	0.503872	-1.192974	3.338102
H	-0.199637	-0.414579	3.653618
H	1.205050	-1.378766	4.160301
H	1.975664	-0.152108	2.505798
C	-0.226669	-2.471401	2.966904
H	-0.795100	-2.836492	3.829868
H	0.482389	-3.249791	2.665079
H	-0.924532	-2.296992	2.142958
C	1.052792	2.193314	-6.957049
C	0.350468	3.165882	-7.684217

C	1.299200	0.956188	-7.570749
C	-0.094822	2.911446	-8.983312
H	0.154994	4.134128	-7.227844
C	0.856365	0.695899	-8.869594
H	1.848159	0.191156	-7.025366
C	0.156466	1.673743	-9.581126
H	-0.633687	3.681260	-9.529908
H	1.062036	-0.268499	-9.327241
H	-0.185911	1.474789	-10.593170

TS1b

E[M06-2X/6-31G(d,p)/CPCM(ether)]= -1820.47606862
Zero-point correction= 0.571115
Thermal correction to Energy= 0.609387
Thermal correction to Enthalpy= 0.610331
Thermal correction to Gibbs Free Energy= 0.494222

Fe	0.472053	0.724918	-0.091014
N	2.047016	1.906103	-0.507624
N	1.303865	-0.725289	-1.247118
N	0.018152	1.942403	1.465165
N	-0.731546	-0.681746	0.714060
C	2.304207	3.160232	0.007663
C	0.819123	-1.997389	-1.461629
C	3.013133	1.698020	-1.470461
C	2.356816	-0.578734	-2.123974
C	0.536509	3.193861	1.714129
C	-0.944124	-1.970505	0.250048
C	-1.004295	1.777649	2.374018
C	-1.659781	-0.501951	1.727312
C	3.441113	3.749091	-0.649606
C	1.578359	-2.657956	-2.492547
C	3.881319	2.841792	-1.565754
C	2.532723	-1.777228	-2.903873
C	-0.169741	3.823754	2.800300
C	-1.997835	-2.602815	0.993540
C	-1.126671	2.944789	3.209622
C	-2.442475	-1.692938	1.906413
H	3.844442	4.726800	-0.419214
H	1.396270	-3.667494	-2.838104
H	4.720969	2.919854	-2.244421
H	3.297577	-1.912795	-3.657764
H	0.054919	4.808208	3.190245

H	-2.342502	-3.615431	0.827889
H	-1.851217	3.056674	4.005963
H	-3.225389	-1.806371	2.645185
C	1.596927	3.772204	1.031733
C	-0.231574	-2.582856	-0.769798
C	-1.798141	0.645757	2.493295
C	3.155077	0.550260	-2.236572
H	1.909245	4.766287	1.334517
H	-0.506025	-3.598692	-1.034723
H	-2.568253	0.648985	3.257752
H	3.959851	0.527954	-2.964169
N	-2.278724	0.897173	-1.675805
N	-2.822355	-0.006486	-1.227480
N	-0.822599	1.474569	-1.412616
O	1.823966	-1.118795	2.600593
C	2.548151	-2.321369	2.330500
H	2.815206	-2.379990	1.264955
H	1.931786	-3.202439	2.567204
C	3.805768	-2.322555	3.184482
H	4.385053	-3.236344	3.011965
H	3.548987	-2.271212	4.248345
H	4.437013	-1.460687	2.941135
C	-0.652425	2.644011	-2.199688
C	-0.166653	4.914201	-3.789704
C	-1.299780	3.868025	-1.891420
C	0.209287	2.582836	-3.311452
C	0.464393	3.705357	-4.095334
C	-1.039076	4.981667	-2.703040
H	0.680594	1.632411	-3.541347
H	1.140917	3.635883	-4.943169
H	-1.527495	5.925396	-2.467811
H	0.017985	5.798748	-4.393778
C	-2.271654	4.000829	-0.739465
H	-2.079395	4.947704	-0.216181
H	-2.096675	3.195635	-0.020230
C	-3.750673	3.979749	-1.179891
H	-3.952586	3.049608	-1.727131
H	-3.928904	4.800045	-1.889895
C	-4.725196	4.102309	-0.001612
H	-4.513697	5.030344	0.548942
H	-4.541614	3.279704	0.704234
H	1.042405	-1.094578	2.022342
C	-6.195530	4.087338	-0.433589
H	-6.866968	4.175047	0.428600

H	-6.416441	4.918770	-1.114598
H	-6.444096	3.155801	-0.957084

TS2a

E[M06-2X/6-31G(d,p)/CPCM(ether)]= -1710.96742569
Zero-point correction= 0.561017
Thermal correction to Energy= 0.594784
Thermal correction to Enthalpy= 0.595729
Thermal correction to Gibbs Free Energy= 0.493900

N	-2.320466	23.136089	-28.743342
C	-3.365418	22.149725	-28.883638
C	-3.021034	21.117013	-29.976439
C	-1.661420	20.460265	-29.697354
C	-0.555557	21.508286	-29.634766
O	-2.555857	26.902471	-27.328292
H	-3.293448	27.276051	-27.839131
N	-0.839605	24.529244	-26.786562
N	-3.704328	24.352614	-26.697945
N	-0.977378	25.488019	-29.459843
C	1.084471	25.174758	-28.167384
C	-2.169842	23.718449	-24.886828
C	-5.775072	24.815059	-27.933249
C	-2.509436	26.067648	-31.292306
C	0.506954	24.704612	-26.992267
C	-3.437853	23.880593	-25.436974
C	-5.199092	25.264578	-29.118788
C	-1.241212	25.918766	-30.736444
C	1.248643	24.316015	-25.813081
C	-4.672750	23.605868	-24.735055
C	-5.938262	25.669288	-30.292229
C	-0.007529	26.237200	-31.421724
C	0.334249	23.895308	-24.896282
C	-5.686783	23.924847	-25.585850
C	-5.018688	26.012157	-31.237764
C	1.003086	25.999759	-30.539377
C	-0.967029	24.032066	-25.513121
C	-5.072124	24.390629	-26.809493
C	-3.715533	25.821462	-30.643354
C	0.386847	25.530322	-29.318251
N	-3.847204	25.373867	-29.348747
Fe	-2.340639	24.808187	-28.118376
H	-4.309436	22.650361	-29.136735

H	-3.534645	21.618042	-27.929141
H	-2.997963	21.619909	-30.952803
H	-3.812239	20.358524	-30.019258
H	-1.434135	19.718277	-30.477403
H	-1.722706	19.906650	-28.752134
H	-1.227271	22.501890	-29.053891
H	-0.368896	21.960770	-30.614550
H	2.166749	25.257507	-28.193243
H	-2.115447	23.335270	-23.871993
H	-6.859582	24.785933	-27.884964
H	-2.562509	26.422028	-32.317705
H	-6.754696	23.862750	-25.417057
H	-7.018603	25.683105	-30.366201
H	-5.189978	26.365115	-32.247166
H	0.057067	26.600919	-32.439775
H	2.068808	26.127113	-30.683819
H	2.326452	24.361804	-25.719852
H	0.505229	23.527780	-23.892081
H	-4.735050	23.228124	-23.722051
C	-1.349733	28.862569	-28.236102
H	-0.605038	29.621479	-27.969302
H	-0.991267	28.314440	-29.111728
H	-2.274578	29.388423	-28.506402
C	-1.578370	27.926919	-27.057711
H	-1.905403	28.492418	-26.175219
H	-0.665863	27.387273	-26.797379
C	0.699617	21.234566	-28.903881
C	1.922620	21.735721	-29.398516
C	0.735130	20.505810	-27.696040
C	3.124234	21.508159	-28.730239
H	1.921179	22.304373	-30.325503
C	1.936629	20.280555	-27.025557
H	-0.186169	20.114789	-27.273989
C	3.139278	20.776638	-27.538595
H	4.051685	21.898240	-29.142022
H	1.934392	19.713951	-26.097796
H	4.074836	20.595824	-27.016387

TS2b

E[M06-2X/6-31G(d,p)/CPCM(ether)]= -1710.97079311

Zero-point correction= 0.560320

Thermal correction to Energy= 0.594318

Thermal correction to Enthalpy= 0.595262

Thermal correction to Gibbs Free Energy=
0.494675

Fe	0.625537	0.956074	0.129973
N	2.143491	2.180649	-0.341501
N	1.298672	-0.378555	-1.237929
N	0.175881	2.107824	1.723854
N	-0.712041	-0.421593	0.782901
C	2.369420	3.429112	0.181384
C	0.674952	-1.546665	-1.618089
C	3.146972	1.960414	-1.252372
C	2.417501	-0.266216	-2.035293
C	0.660170	3.369598	1.966944
C	-1.063456	-1.584118	0.140003
C	-0.727350	1.844866	2.723511
C	-1.500971	-0.354688	1.905665
C	3.530955	4.027339	-0.439390
C	1.412148	-2.176535	-2.687533
C	4.022205	3.109736	-1.317068
C	2.500203	-1.392509	-2.934033
C	0.042134	3.920127	3.152695
C	-2.110811	-2.261972	0.870553
C	-0.806946	2.967794	3.631212
C	-2.369971	-1.508190	1.975411
H	3.918612	5.010439	-0.203353
H	1.132535	-3.108041	-3.163562
H	4.892457	3.186325	-1.956824
H	3.289155	-1.545342	-3.659790
H	0.255370	4.900021	3.561240
H	-2.562001	-3.200523	0.573727
H	-1.439570	3.007883	4.509190
H	-3.082797	-1.696782	2.768435
C	1.654810	4.004426	1.227955
C	-0.447987	-2.090911	-1.001941
C	-1.494583	0.688054	2.827639
C	3.299164	0.810385	-2.022434
H	1.946569	5.002690	1.540319
H	-0.831065	-3.025224	-1.401238
H	-2.172224	0.612164	3.672637
H	4.144418	0.772653	-2.702947
N	-0.588502	1.824327	-0.936974
O	2.207934	-0.141701	1.377177
C	2.126444	-0.587631	2.750802
H	2.648536	-1.547949	2.835575
H	1.076248	-0.740981	3.019898

C	2.776583	0.449271	3.650072
H	2.708069	0.129446	4.696124
H	2.276354	1.417055	3.551457
H	3.834321	0.575460	3.395057
C	-0.522769	2.615806	-2.068954
C	-0.461940	4.249249	-4.386854
C	-1.401009	3.737536	-2.197422
C	0.356456	2.337631	-3.142485
C	0.390355	3.147962	-4.274072
C	-1.356458	4.523059	-3.347377
H	1.001081	1.473209	-3.078007
H	1.080242	2.906539	-5.079237
H	-2.035347	5.369651	-3.428681
H	-0.441779	4.877686	-5.272818
C	-2.366260	4.020865	-1.071386
H	-3.166554	4.690185	-1.421113
H	-1.854527	4.549405	-0.251352
C	-2.943866	2.725620	-0.529105
H	-1.743611	2.055944	-0.456154
H	-3.449055	2.141245	-1.308045
C	-3.726935	2.806956	0.765003
H	-4.359292	3.711028	0.724027
H	-3.045098	2.967155	1.610360
H	2.189378	-0.915003	0.788635
C	-4.622510	1.591715	1.028285
H	-4.037322	0.667894	1.076263
H	-5.162695	1.697303	1.976356
H	-5.366996	1.476731	0.230776

XI. NMR Spectra

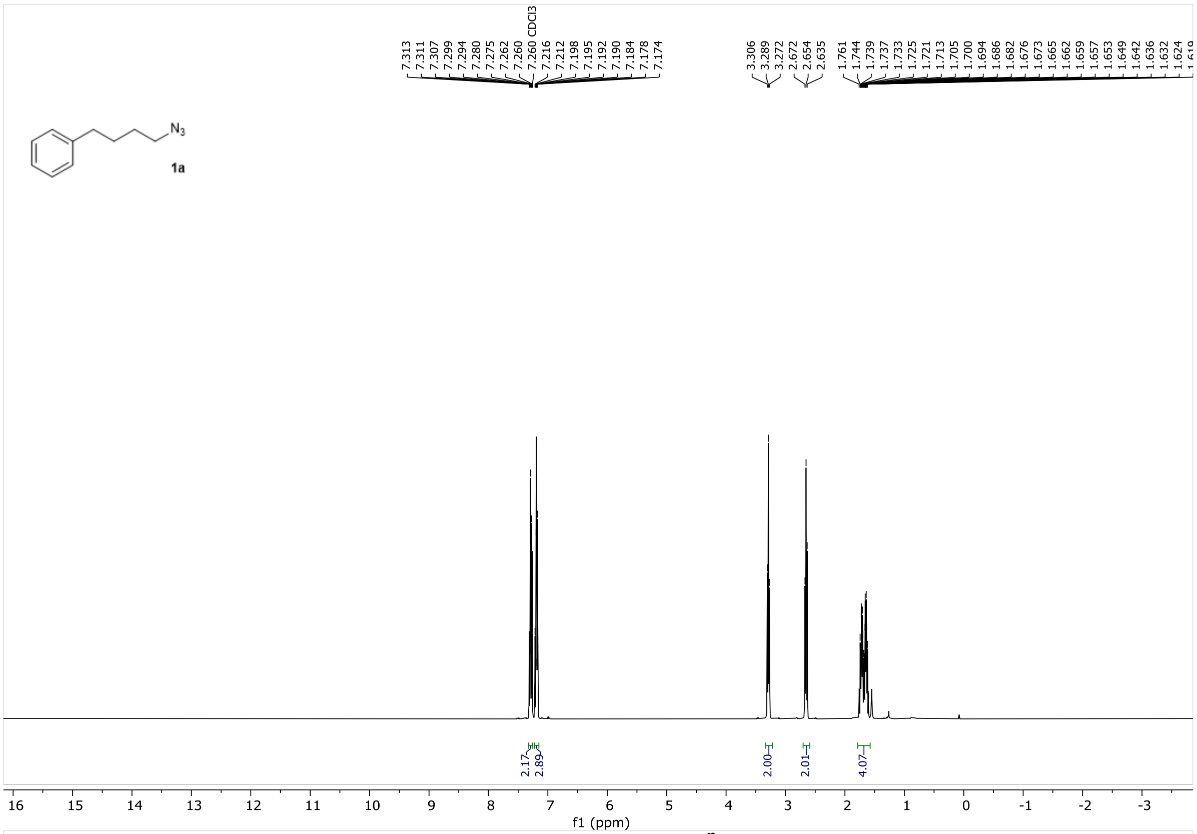

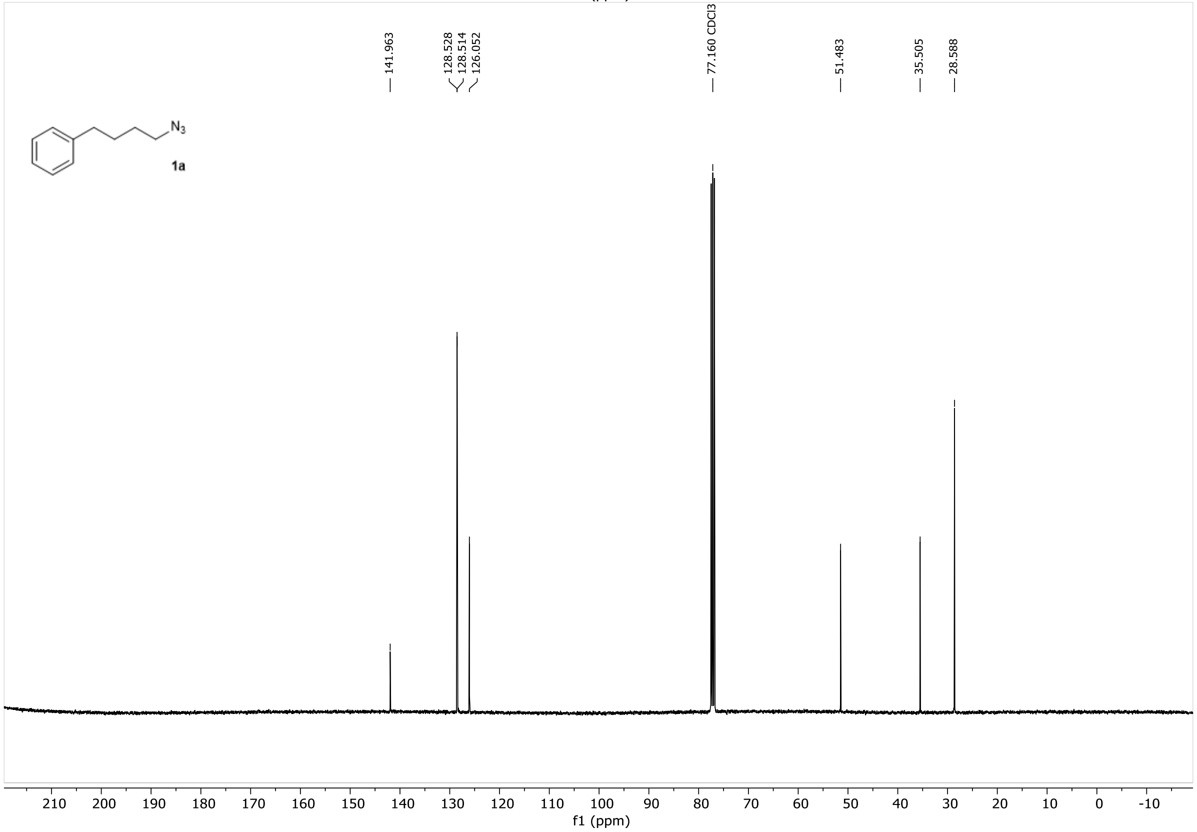

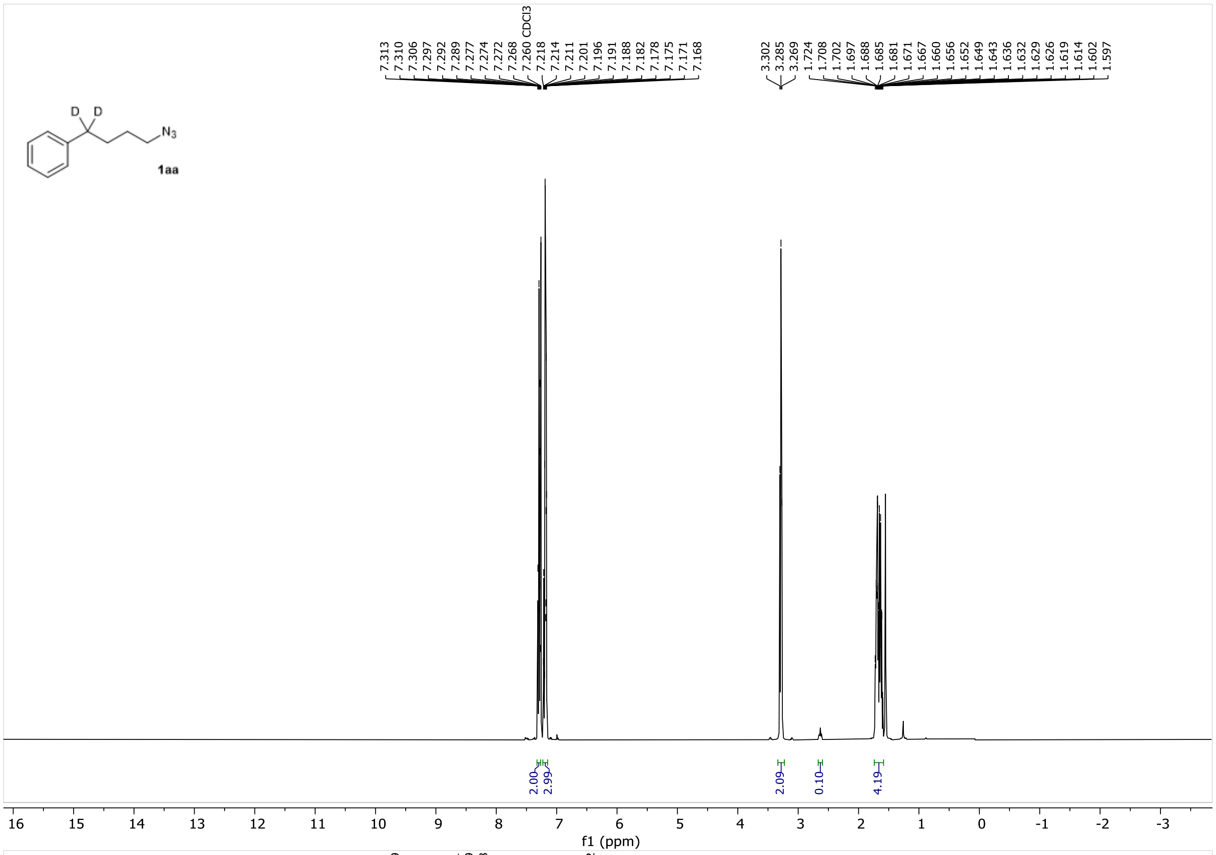
7.313
7.310
7.306
7.297
7.292
7.289
7.277
7.274
7.272
7.268
7.260 CDCl3
7.218
7.214
7.211
7.201
7.196
7.191
7.188
7.182
7.178
7.175
7.171
7.168
3.302
3.285
3.269
1.724
1.708
1.702
1.697
1.688
1.685
1.681
1.671
1.667
1.660
1.656
1.652
1.649
1.643
1.636
1.632
1.629
1.626
1.619
1.614
1.602
1.597
D D
N3
1aa
2.00
2.99
2.09
0.10
4.19
f1 (ppm)

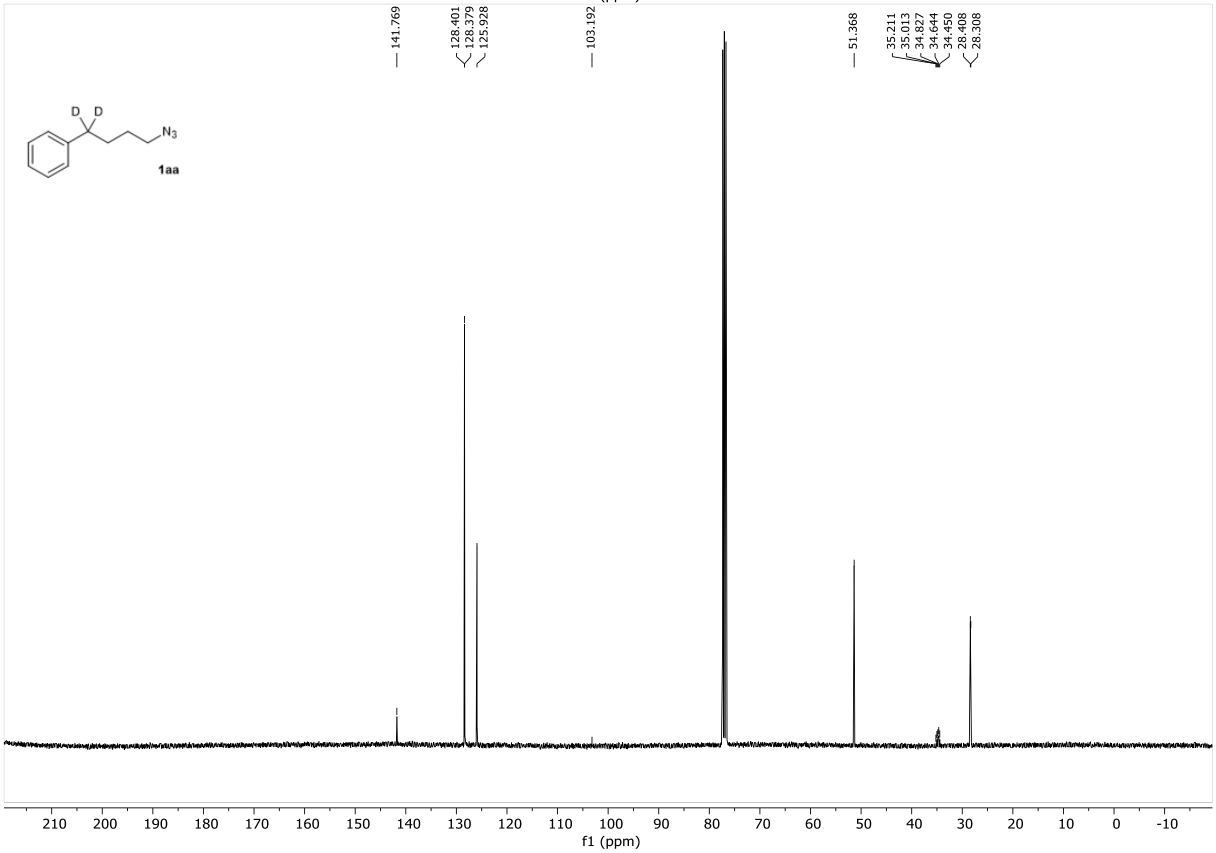
141.769
128.401
128.379
125.928
103.192
51.368
35.211
35.013
34.827
34.644
34.450
28.408
28.308
D D
N3
1aa
f1 (ppm)

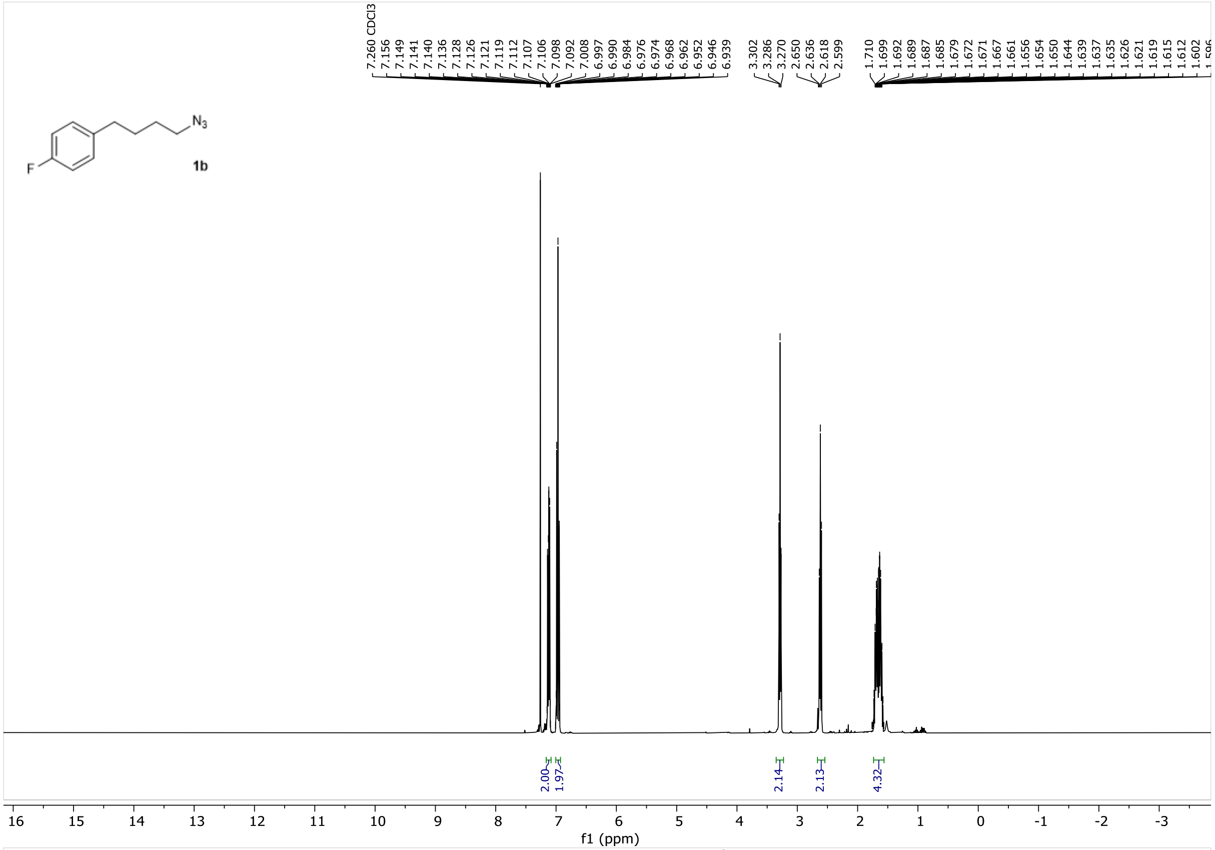
7.260 CDCl3
7.156
7.149
7.141
7.140
7.136
7.128
7.126
7.121
7.119
7.112
7.107
7.106
7.098
7.092
7.008
6.997
6.990
6.984
6.976
6.974
6.968
6.962
6.952
6.946
6.939
3.302
3.286
3.270
2.650
2.636
2.618
2.599
1.710
1.699
1.692
1.689
1.687
1.685
1.679
1.672
1.671
1.667
1.661
1.656
1.654
1.650
1.644
1.639
1.637
1.635
1.626
1.621
1.619
1.615
1.612
1.602
N3
F
1b
2.00
1.97
2.14
2.13
4.32
f1 (ppm)

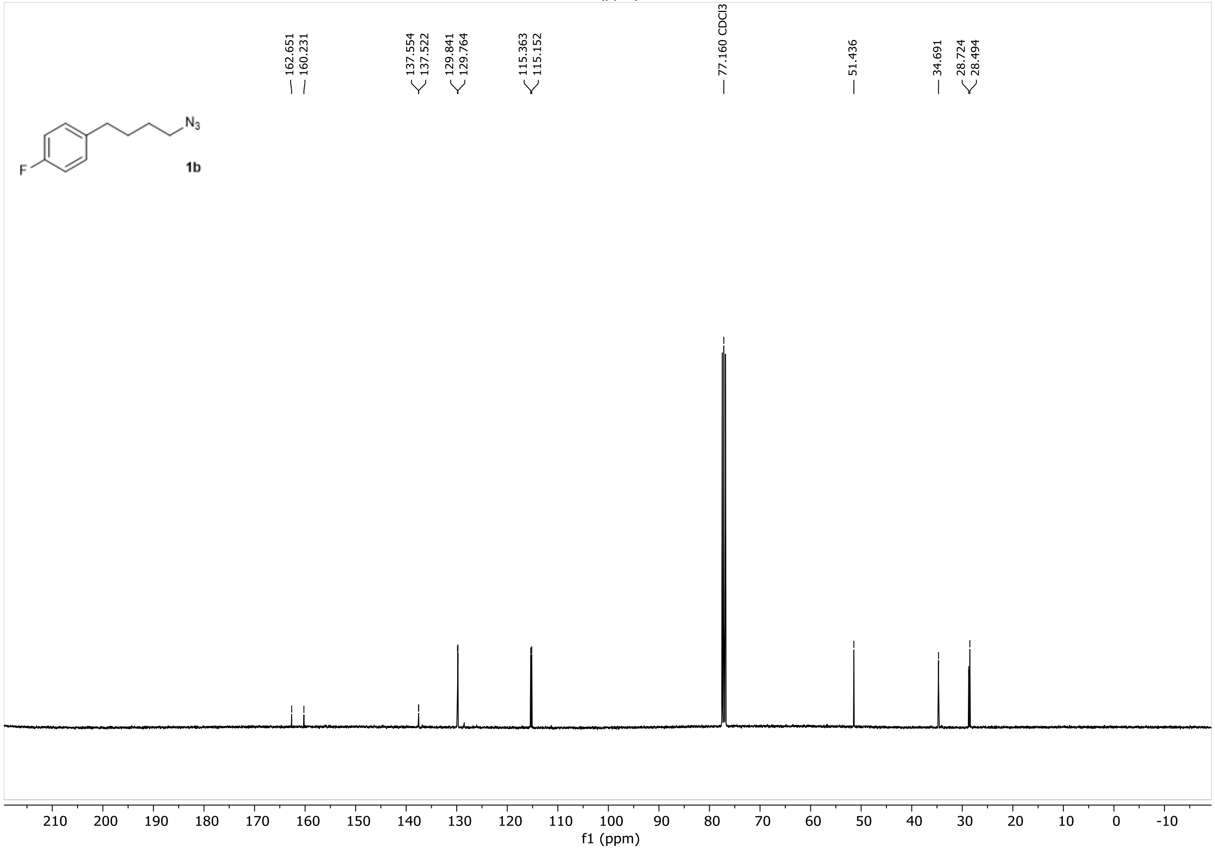
162.651
160.231
137.554
137.522
129.841
129.764
115.363
115.152
77.160 CDCl3
51.436
34.691
28.724
28.494
N3
F
1b
f1 (ppm)

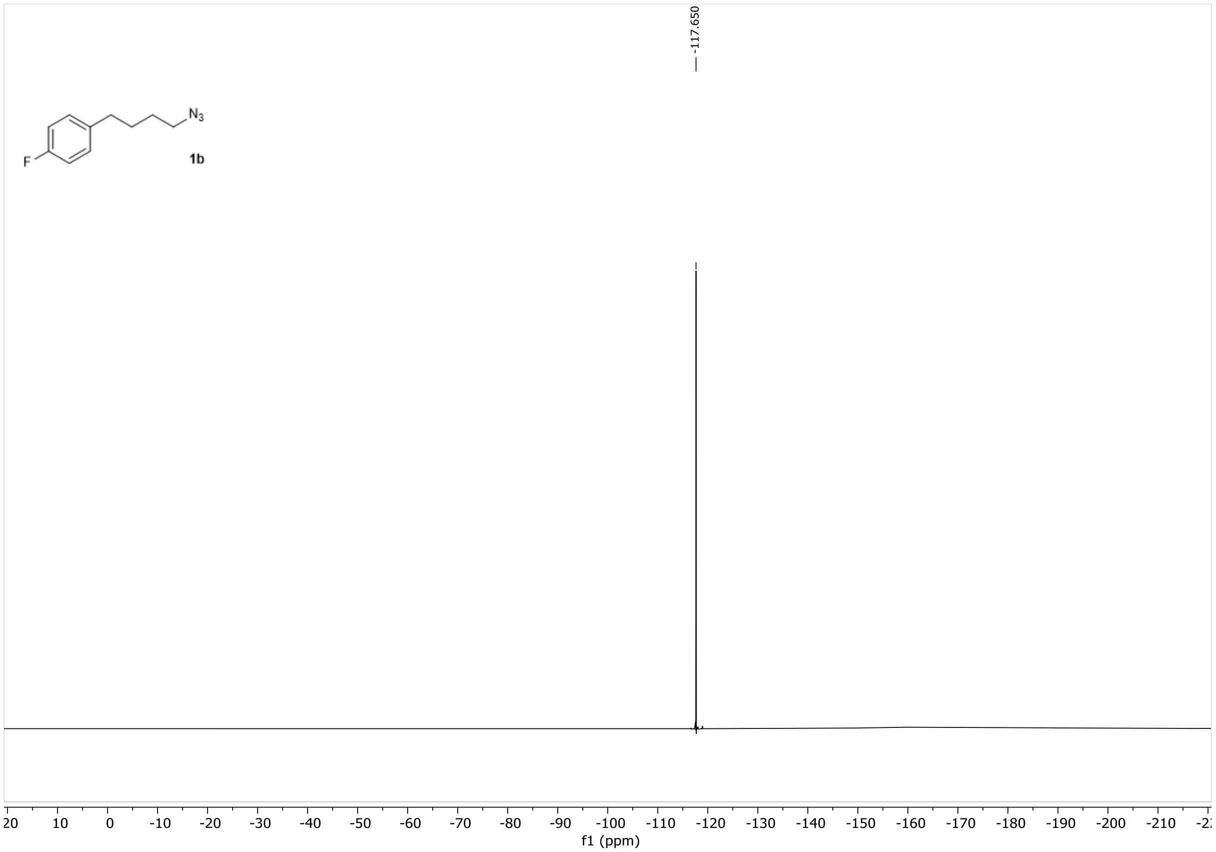

N3
F
1b
-117.650
20
10
0
-10
-20
-30
-40
-50
-60
-70
-80
-90
-100
-110
-120
-130
-140
-150
-160
-170
-180
-190
-200
-210
f1 (ppm)

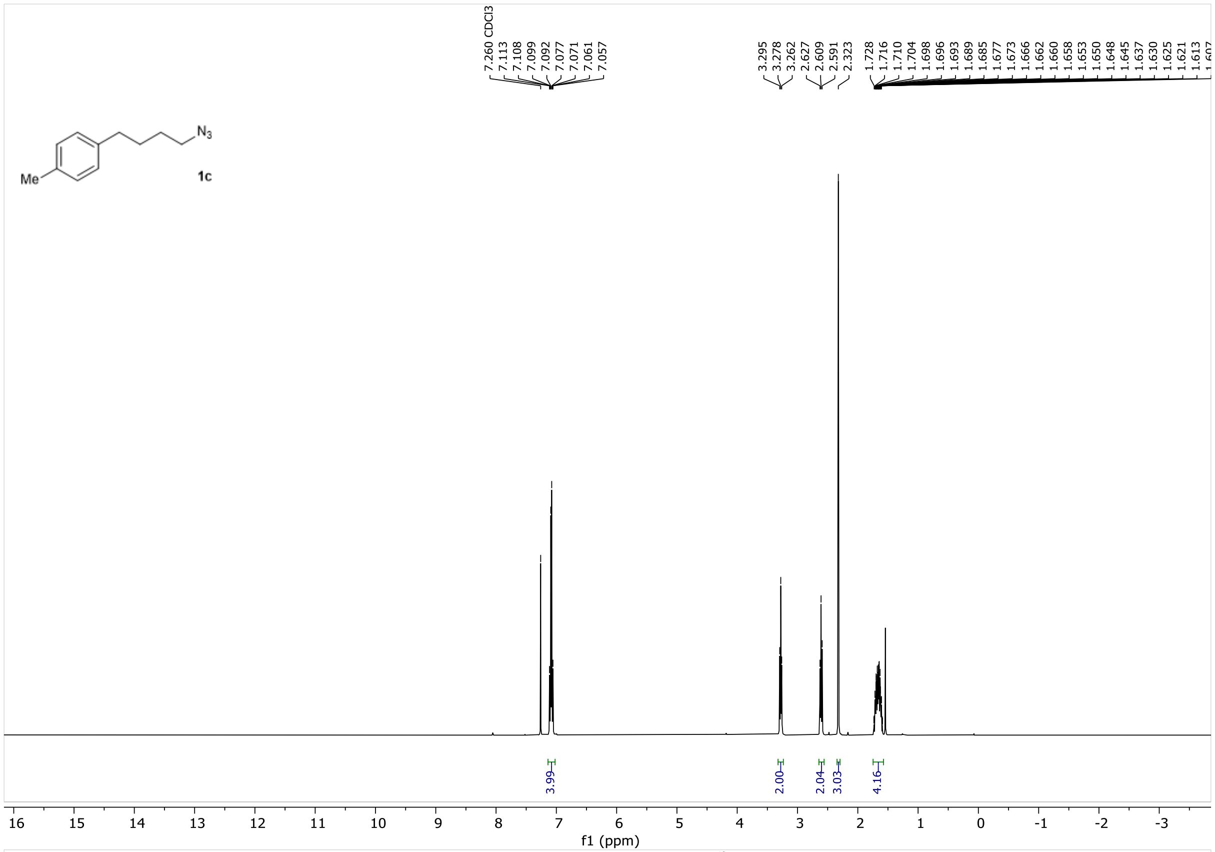
7.260 CDCl3
7.113
7.108
7.099
7.092
7.077
7.071
7.061
7.057
3.295
3.278
3.262
2.627
2.609
2.591
2.323
1.728
1.716
1.710
1.704
1.698
1.696
1.693
1.689
1.685
1.677
1.673
1.666
1.662
1.660
1.658
1.653
1.650
1.648
1.645
1.637
1.630
1.625
1.621
1.613
N3
Me
1c
3.99
2.00
2.04
3.03
4.16
16 15 14 13 12 11 10 9 8 7 6 5 4 3 2 1 0 -1 -2 -3
f1 (ppm)

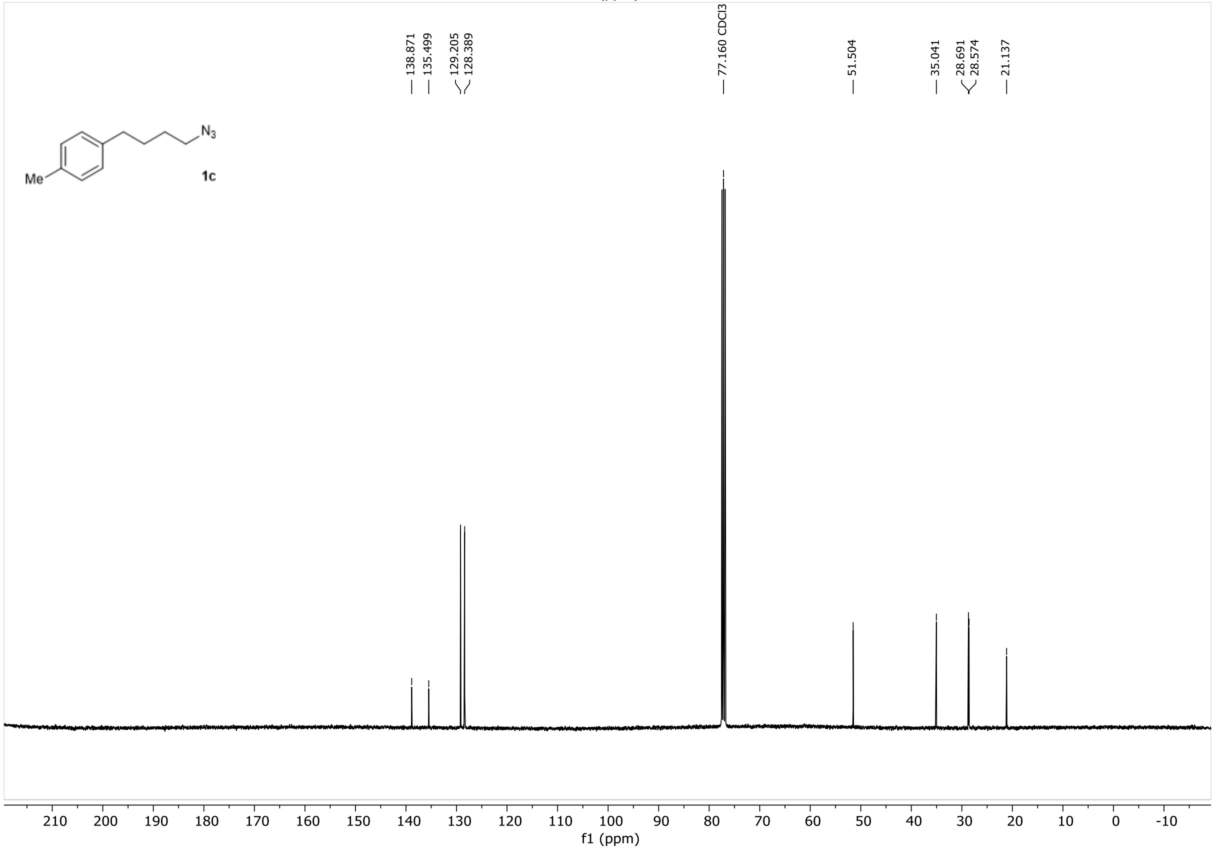
138.871
135.499
129.205
128.389
77.160 CDCl3
51.504
35.041
28.691
28.574
21.137
N3
Me
1c
210 200 190 180 170 160 150 140 130 120 110 100 90 80 70 60 50 40 30 20 10 0 -10
f1 (ppm)

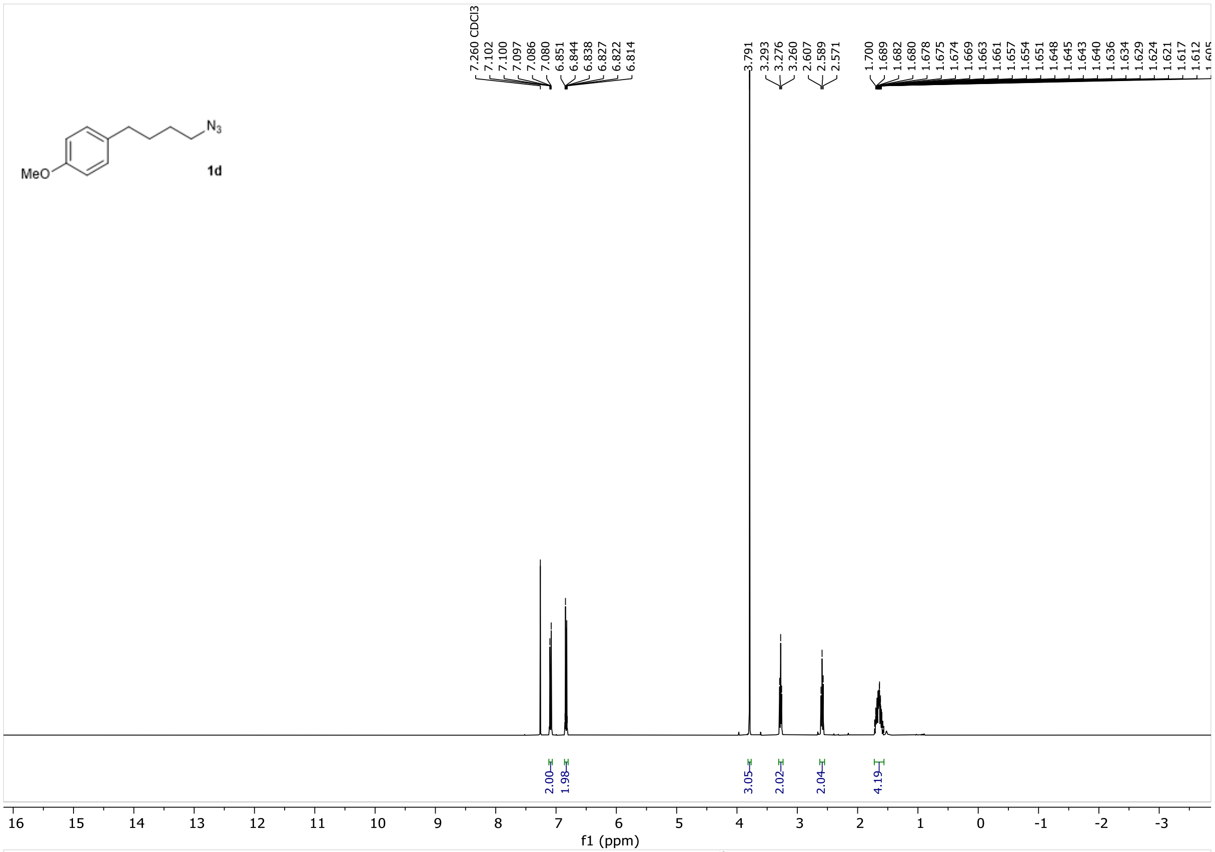
7.260 CDCl3
7.102
7.100
7.097
7.086
7.080
6.851
6.844
6.838
6.827
6.822
6.814
3.791
3.293
3.276
3.260
2.607
2.589
2.571
1.700
1.689
1.682
1.680
1.678
1.675
1.674
1.669
1.663
1.661
1.657
1.654
1.651
1.648
1.645
1.643
1.640
1.636
1.634
1.629
1.624
1.621
1.617
1.612
N_3
MeO
1d
2.00
1.98
3.05
2.02
2.04
4.19
f1 (ppm)

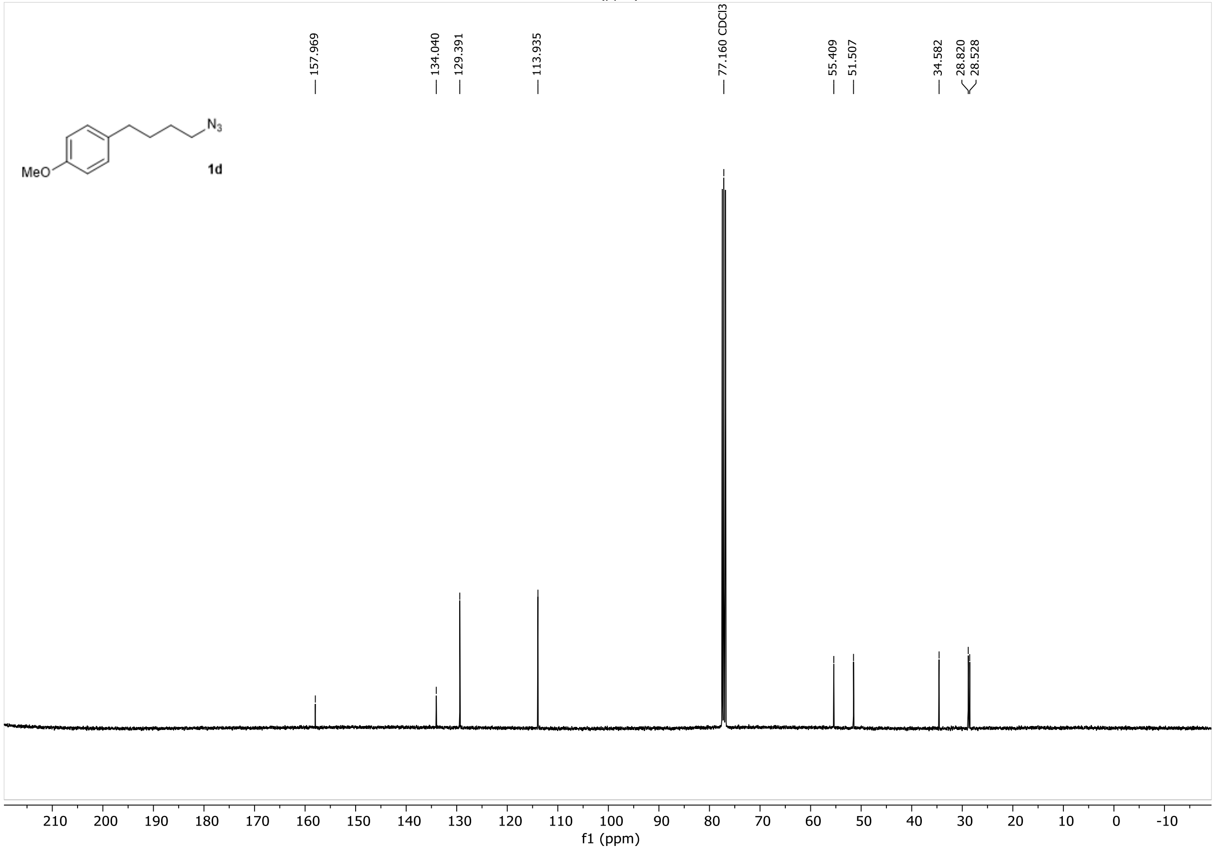
157.969
134.040
129.391
113.935
77.160 CDCl3
55.409
51.507
34.582
28.820
28.528
N_3
MeO
1d
f1 (ppm)

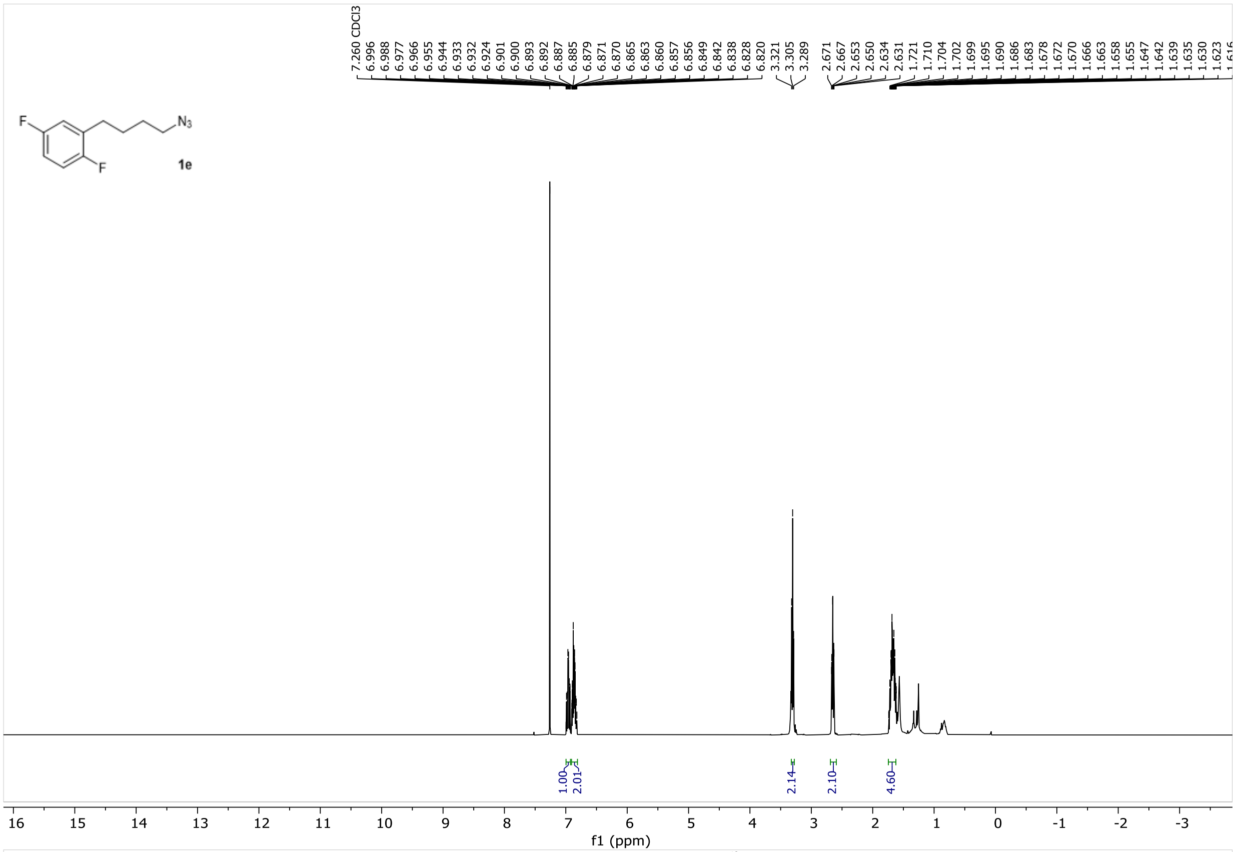
1e
f1 (ppm)

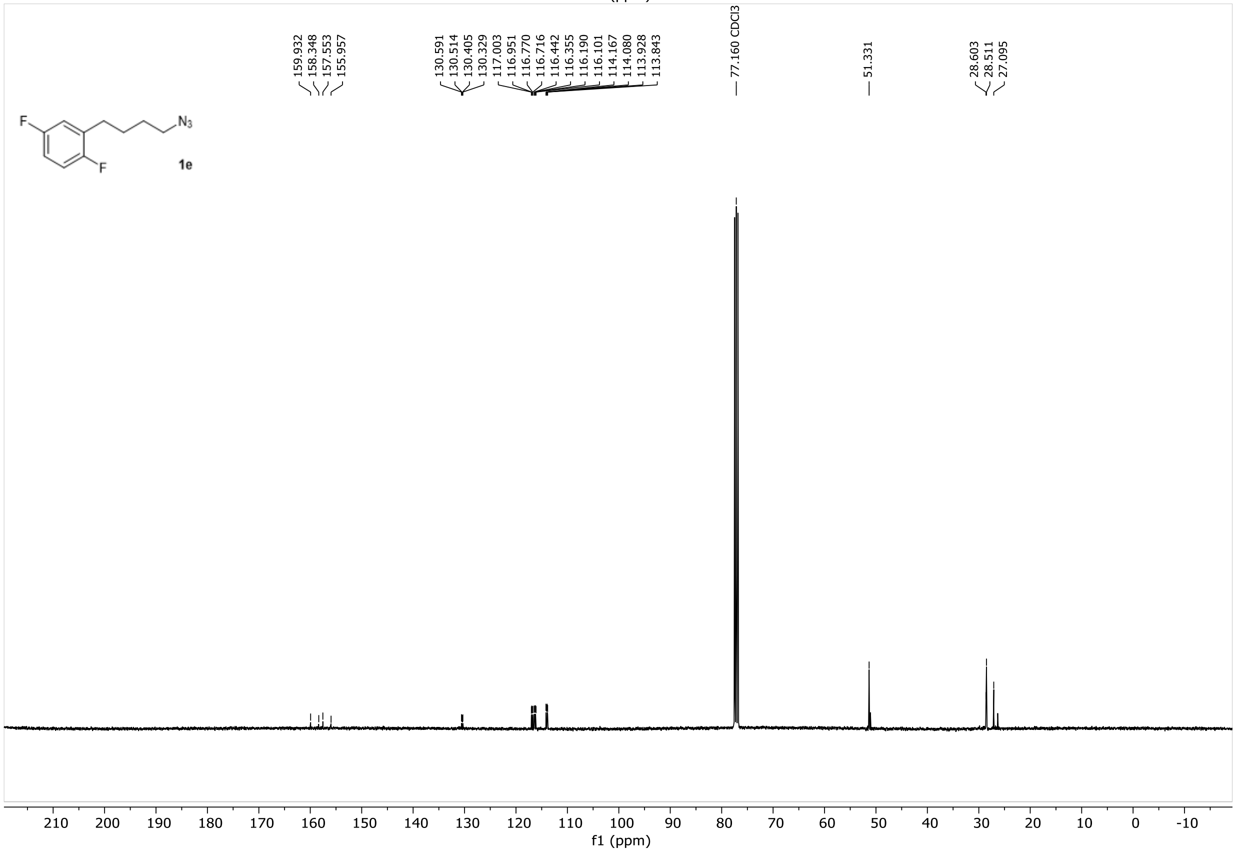
1e
77.160 CDCl3
51.331
f1 (ppm)

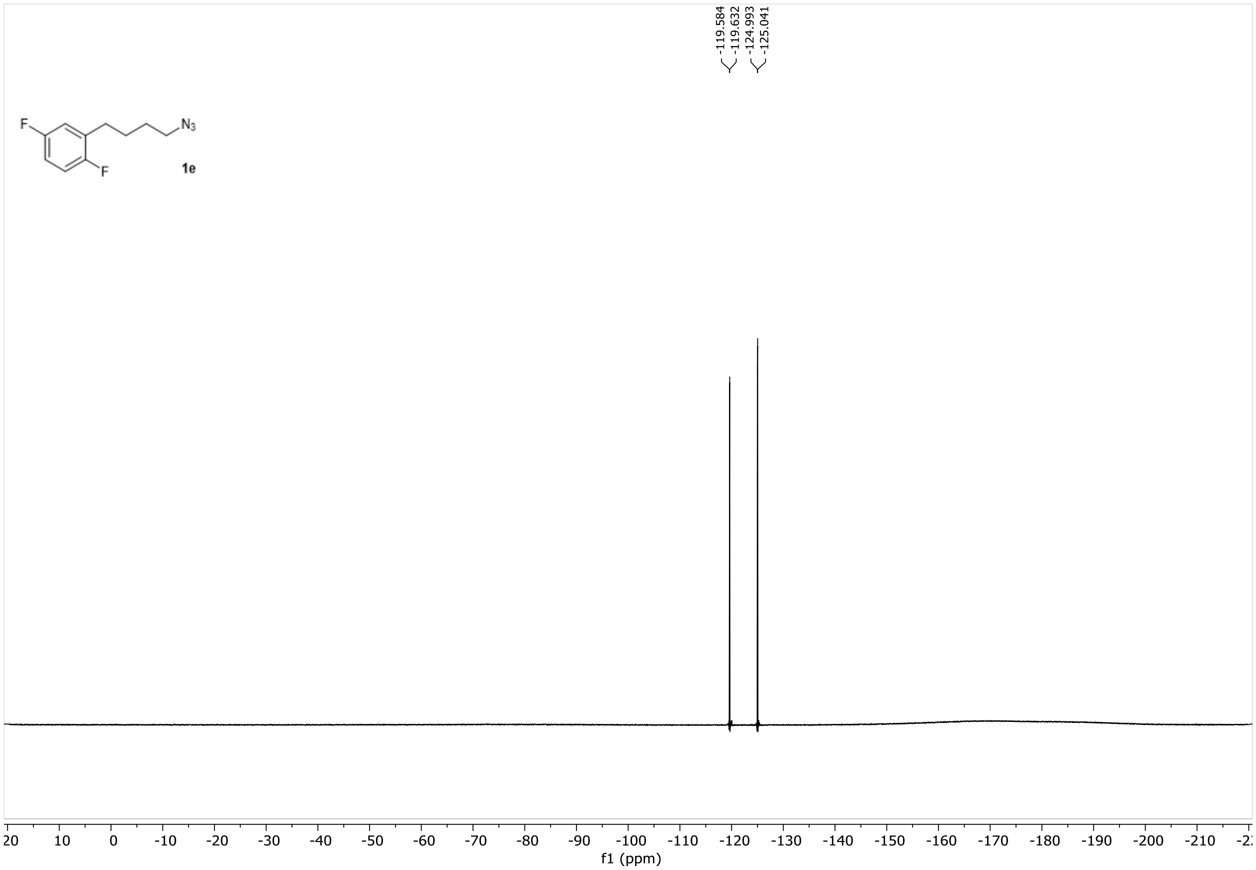
-119.584
-119.632
-124.993
-125.041
F
F
N3
1e
20 10 0 -10 -20 -30 -40 -50 -60 -70 -80 -90 -100 -110 -120 -130 -140 -150 -160 -170 -180 -190 -200 -210
f1 (ppm)

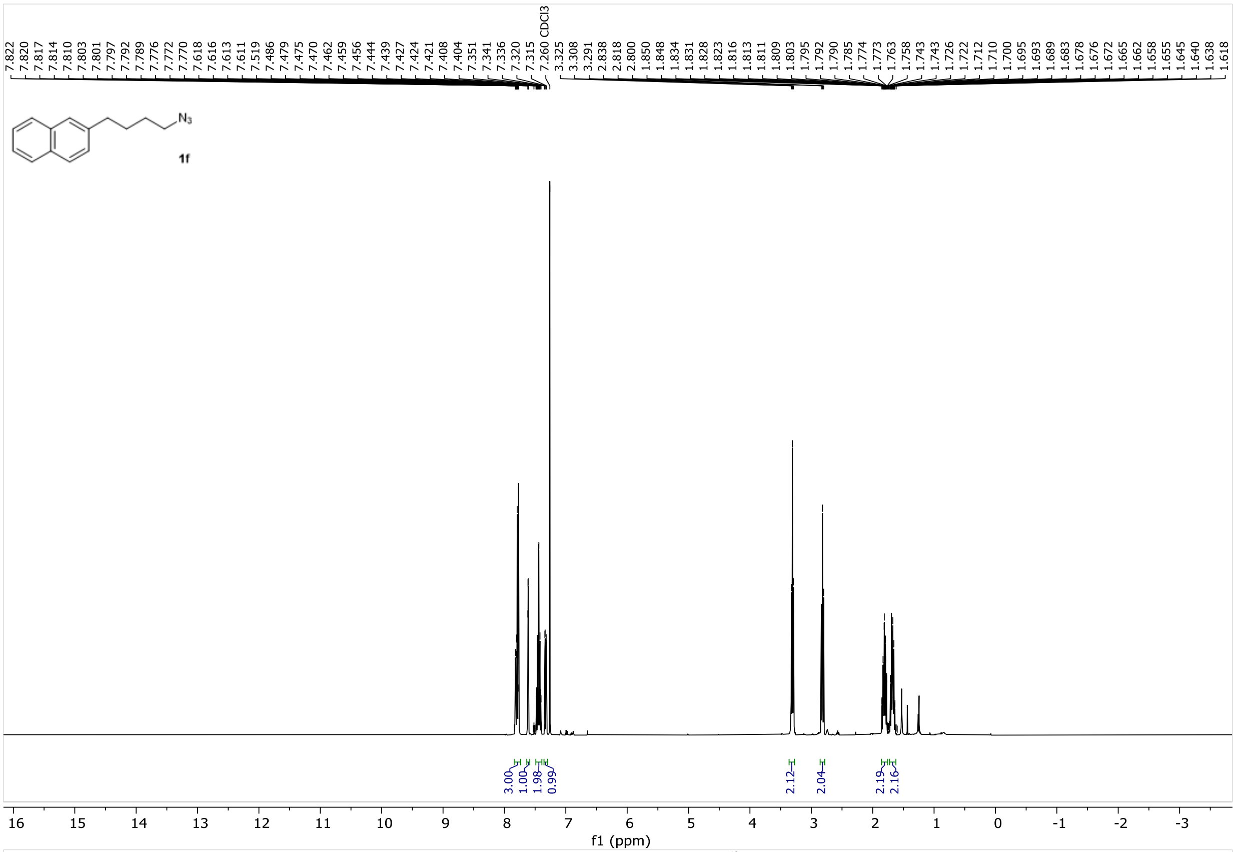

7.822
7.820
7.817
7.814
7.810
7.803
7.801
7.797
7.792
7.789
7.776
7.772
7.770
7.618
7.616
7.613
7.611
7.519
7.486
7.479
7.475
7.470
7.462
7.459
7.456
7.444
7.439
7.427
7.424
7.421
7.408
7.404
7.351
7.341
7.336
7.320
7.315
7.260 CDCl3
3.325
3.308
3.291
2.838
2.818
2.800
1.850
1.848
1.834
1.831
1.828
1.823
1.816
1.813
1.811
1.809
1.803
1.795
1.792
1.790
1.785
1.774
1.773
1.763
1.758
1.743
1.726
1.722
1.712
1.710
1.700
1.695
1.693
1.689
1.683
1.678
1.676
1.672
1.665
1.662
1.658
1.655
1.645
1.640
1.638
1.618
N_3
1f
3.00
1.00
1.98
0.99
2.12
2.04
2.19
2.16
16 15 14 13 12 11 10 9 8 7 6 5 4 3 2 1 0 -1 -2 -3
f1 (ppm)

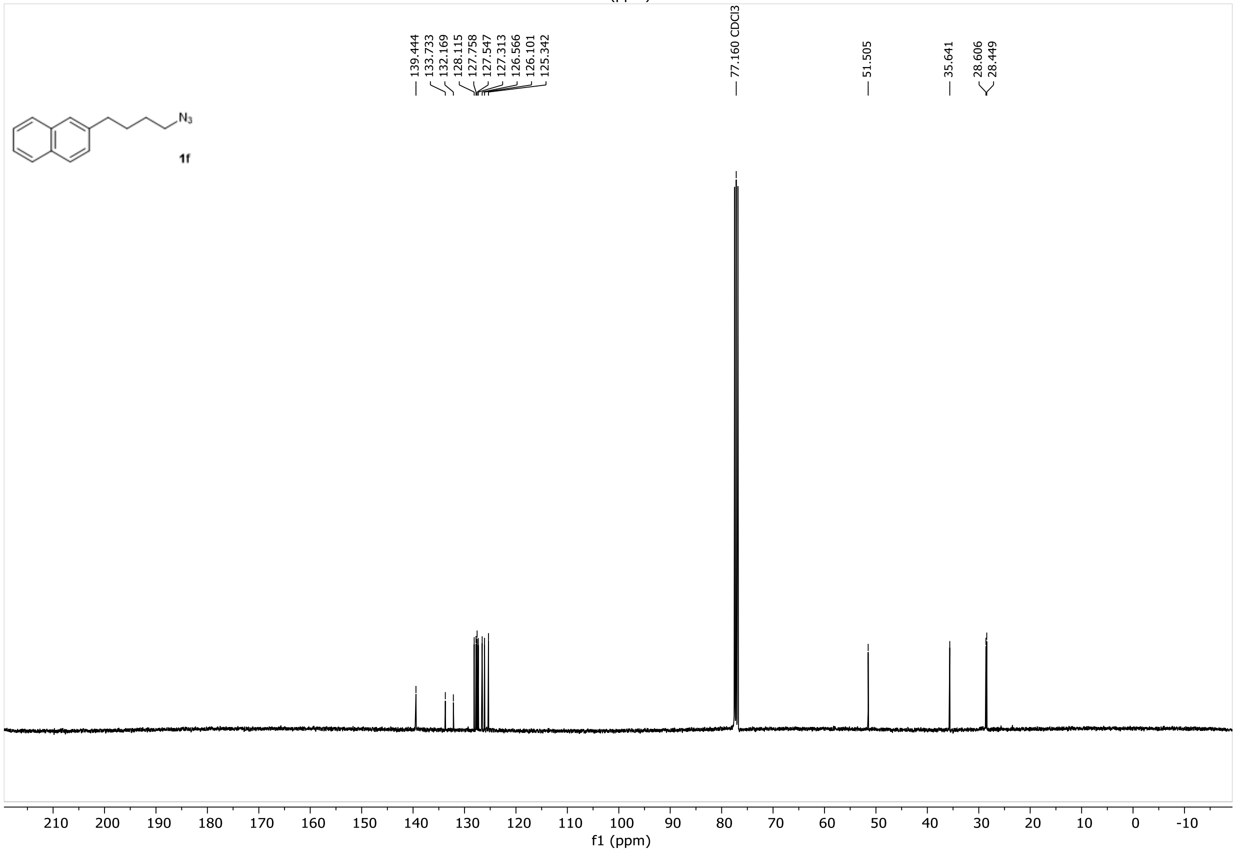

139.444
133.733
132.169
128.115
127.758
127.547
127.313
126.566
126.101
125.342
77.160 CDCl3
51.505
35.641
28.606
28.449
N_3
1f
210 200 190 180 170 160 150 140 130 120 110 100 90 80 70 60 50 40 30 20 10 0 -10
f1 (ppm)

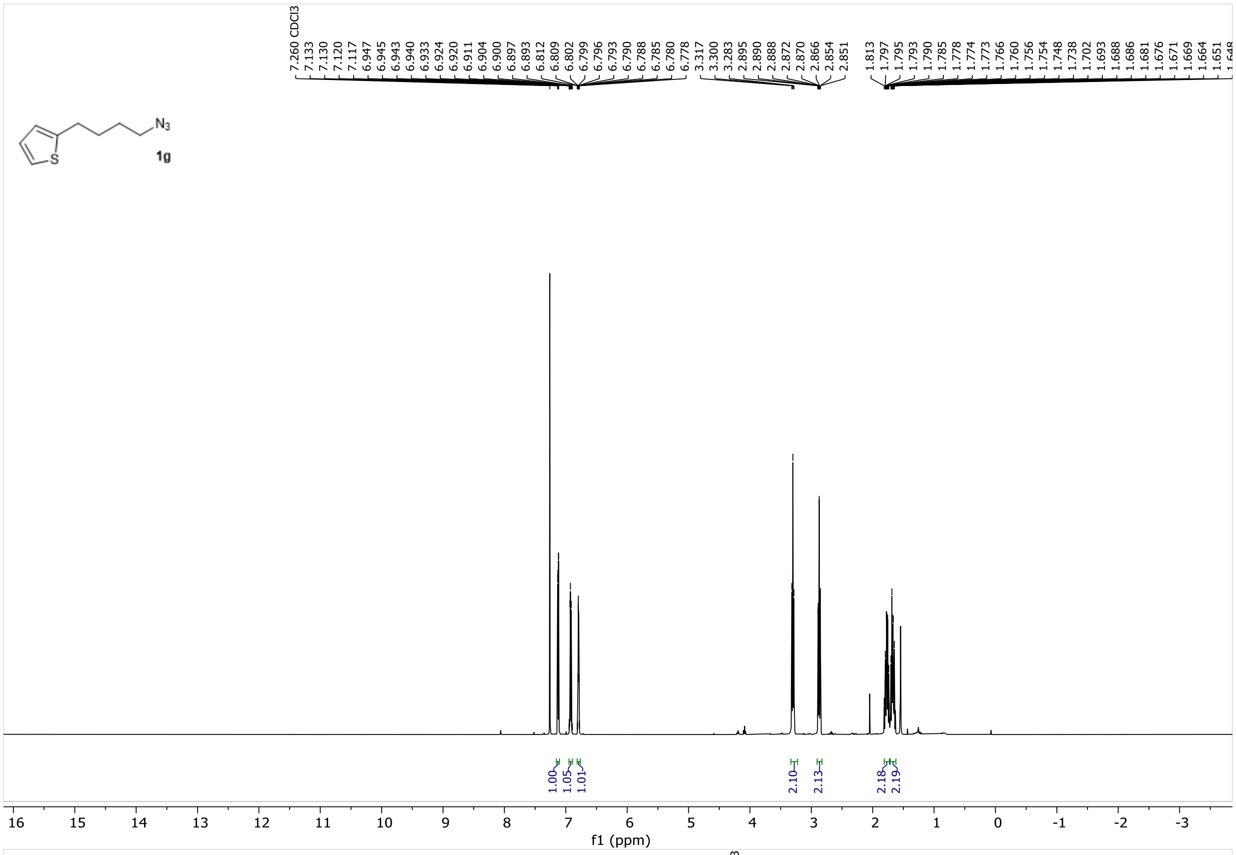

7.260 CDCl3
7.133
7.130
7.120
7.117
6.947
6.945
6.943
6.940
6.933
6.924
6.920
6.911
6.904
6.900
6.897
6.893
6.812
6.809
6.802
6.799
6.796
6.793
6.790
6.788
6.785
6.780
6.778
3.317
3.300
3.283
2.895
2.890
2.888
2.872
2.870
2.866
2.854
2.851
1.813
1.797
1.795
1.793
1.790
1.785
1.778
1.774
1.773
1.766
1.760
1.756
1.754
1.748
1.738
1.702
1.693
1.688
1.686
1.681
1.676
1.671
1.669
1.664
1.651
N_3
S
1g
1.00
1.05
1.01
2.10
2.13
2.18
2.19
16 15 14 13 12 11 10 9 8 7 6 5 4 3 2 1 0 -1 -2 -3
f1 (ppm)

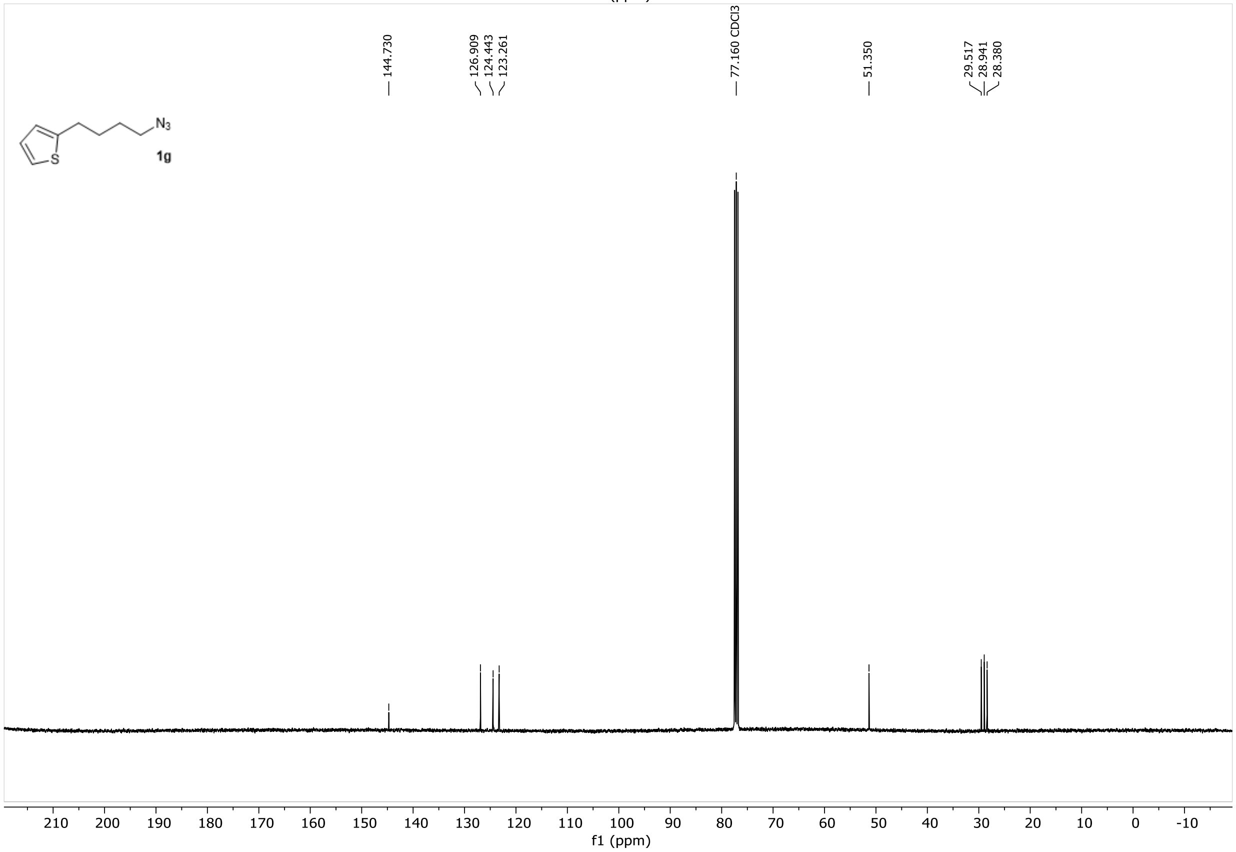

144.730
126.909
124.443
123.261
77.160 CDCl3
51.350
29.517
28.941
28.380
N_3
S
1g
210 200 190 180 170 160 150 140 130 120 110 100 90 80 70 60 50 40 30 20 10 0 -10
f1 (ppm)

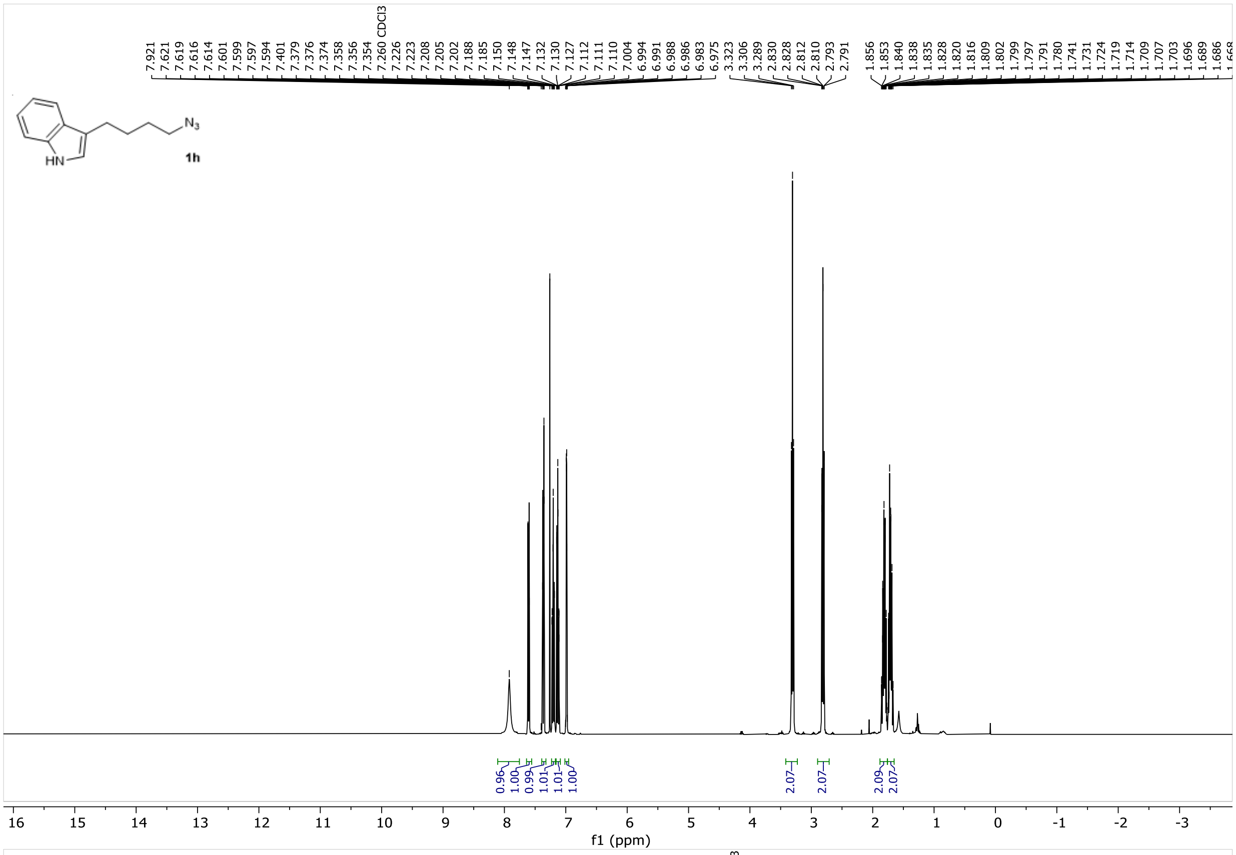
7.921
7.621
7.619
7.616
7.614
7.601
7.599
7.597
7.594
7.401
7.379
7.376
7.374
7.358
7.356
7.354
7.260 CDCl3
7.226
7.223
7.208
7.205
7.202
7.188
7.185
7.150
7.148
7.147
7.132
7.130
7.127
7.112
7.111
7.110
7.004
6.994
6.991
6.988
6.986
6.983
6.975
3.323
3.306
3.289
2.830
2.828
2.812
2.810
2.793
2.791
1.856
1.853
1.840
1.838
1.835
1.828
1.820
1.816
1.809
1.802
1.799
1.797
1.791
1.780
1.741
1.731
1.724
1.719
1.714
1.709
1.707
1.703
1.696
1.689
1.686
1.668
N_3
HN
1h
0.96
1.00
0.99
1.01
1.01
1.00
2.07
2.07
2.09
2.07
f1 (ppm)

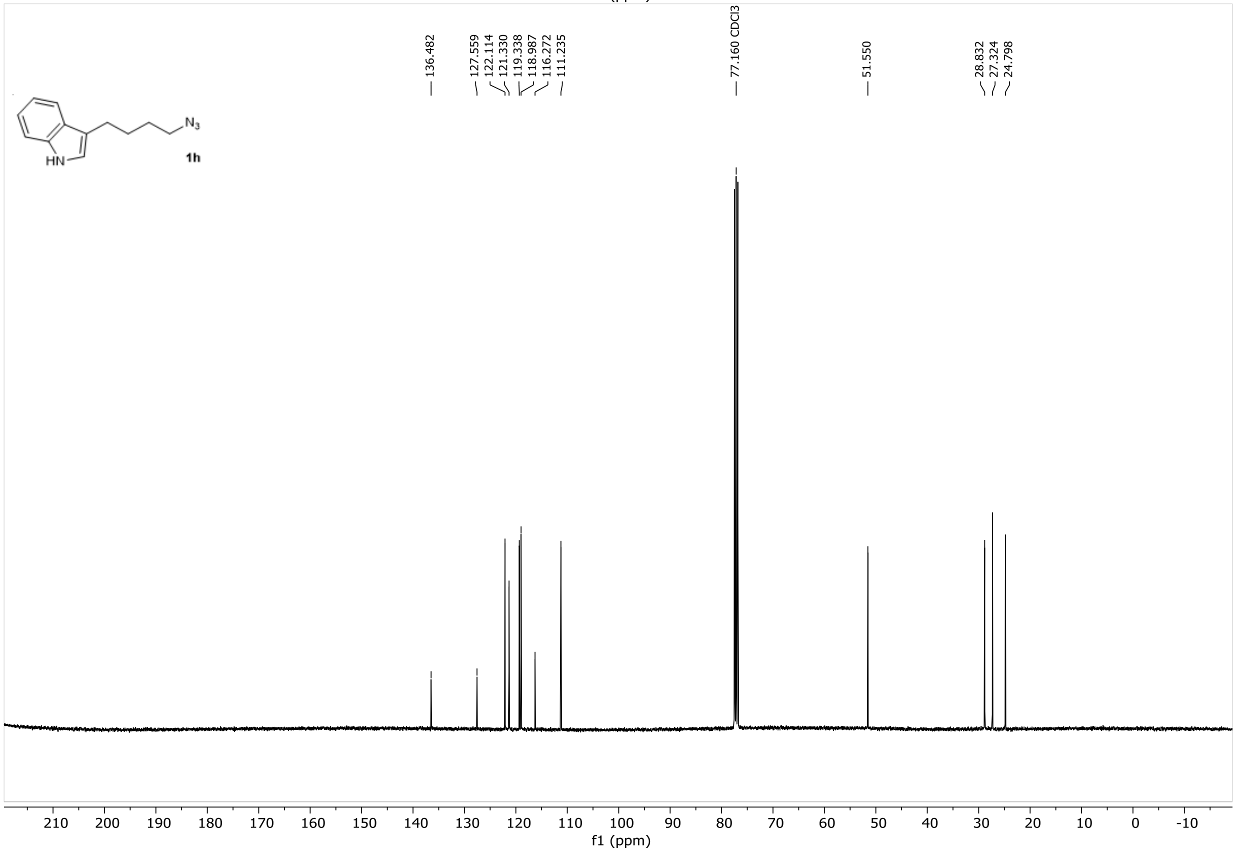
136.482
127.559
122.114
121.330
119.338
118.987
116.272
111.235
77.160 CDCl3
51.550
28.832
27.324
24.798
N_3
HN
1h
f1 (ppm)

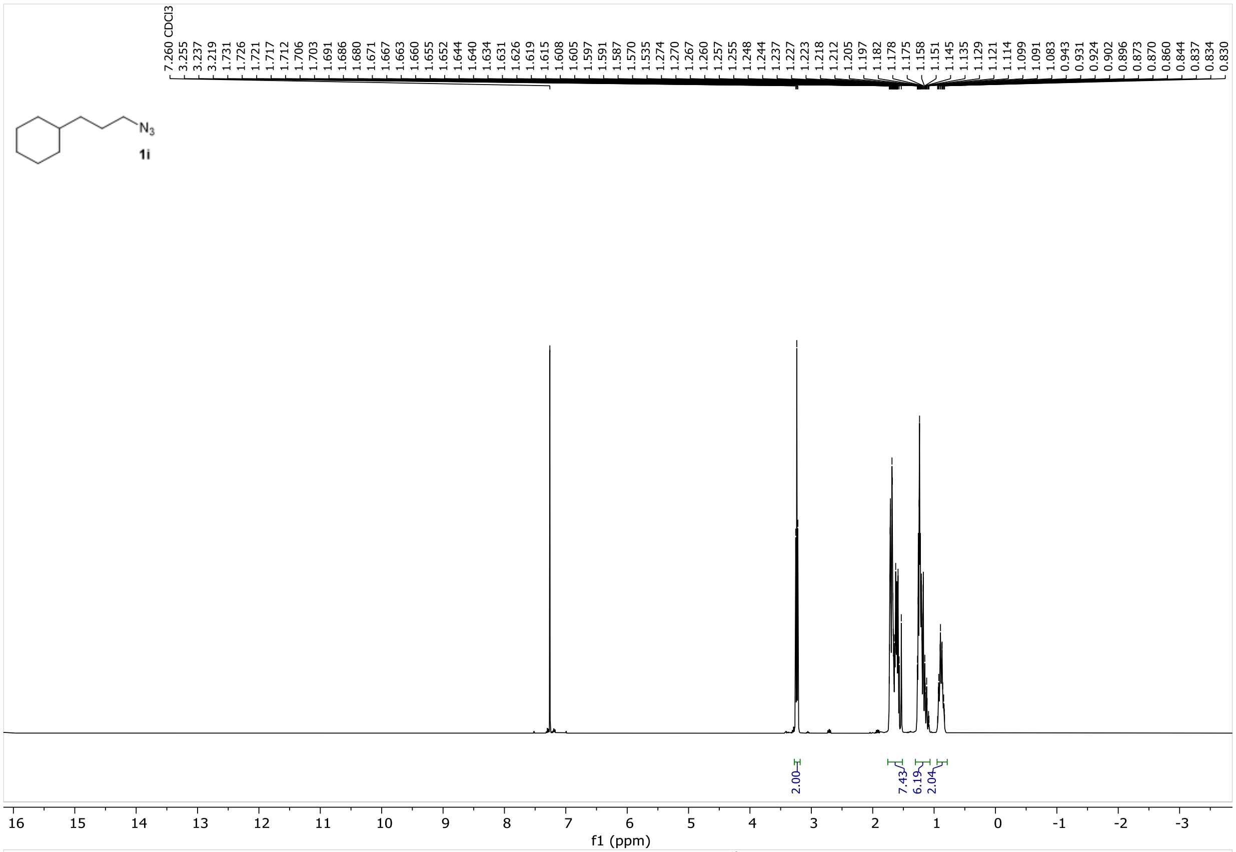
7.260 CDCl3
3.255
3.237
3.219
1.731
1.726
1.721
1.717
1.712
1.706
1.703
1.691
1.686
1.680
1.671
1.667
1.663
1.660
1.655
1.652
1.644
1.640
1.634
1.631
1.626
1.619
1.615
1.608
1.605
1.597
1.591
1.587
1.570
1.535
1.274
1.270
1.267
1.260
1.257
1.255
1.248
1.244
1.237
1.227
1.223
1.218
1.212
1.205
1.197
1.182
1.178
1.175
1.158
1.151
1.145
1.135
1.129
1.121
1.114
1.099
1.091
1.083
0.943
0.931
0.924
0.902
0.896
0.873
0.870
0.860
0.844
0.837
0.834
0.830
N3
1i
2.00
7.43
6.19
2.04
16
15
14
13
12
11
10
9
8
7
6
5
4
3
2
1
0
-1
-2
-3
f1 (ppm)

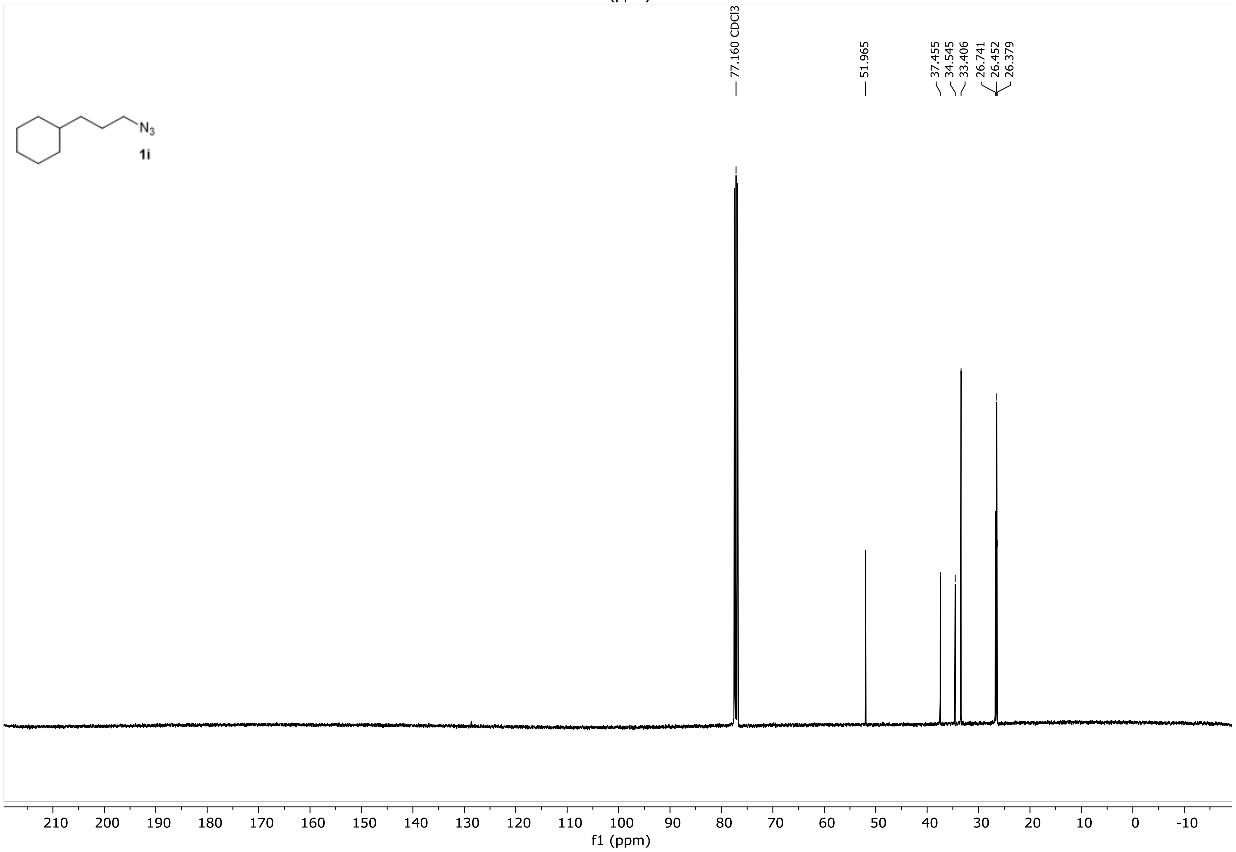
77.160 CDCl3
51.965
37.455
34.545
33.406
26.741
26.452
26.379
N3
1i
210
200
190
180
170
160
150
140
130
120
110
100
90
80
70
60
50
40
30
20
10
0
-10
f1 (ppm)

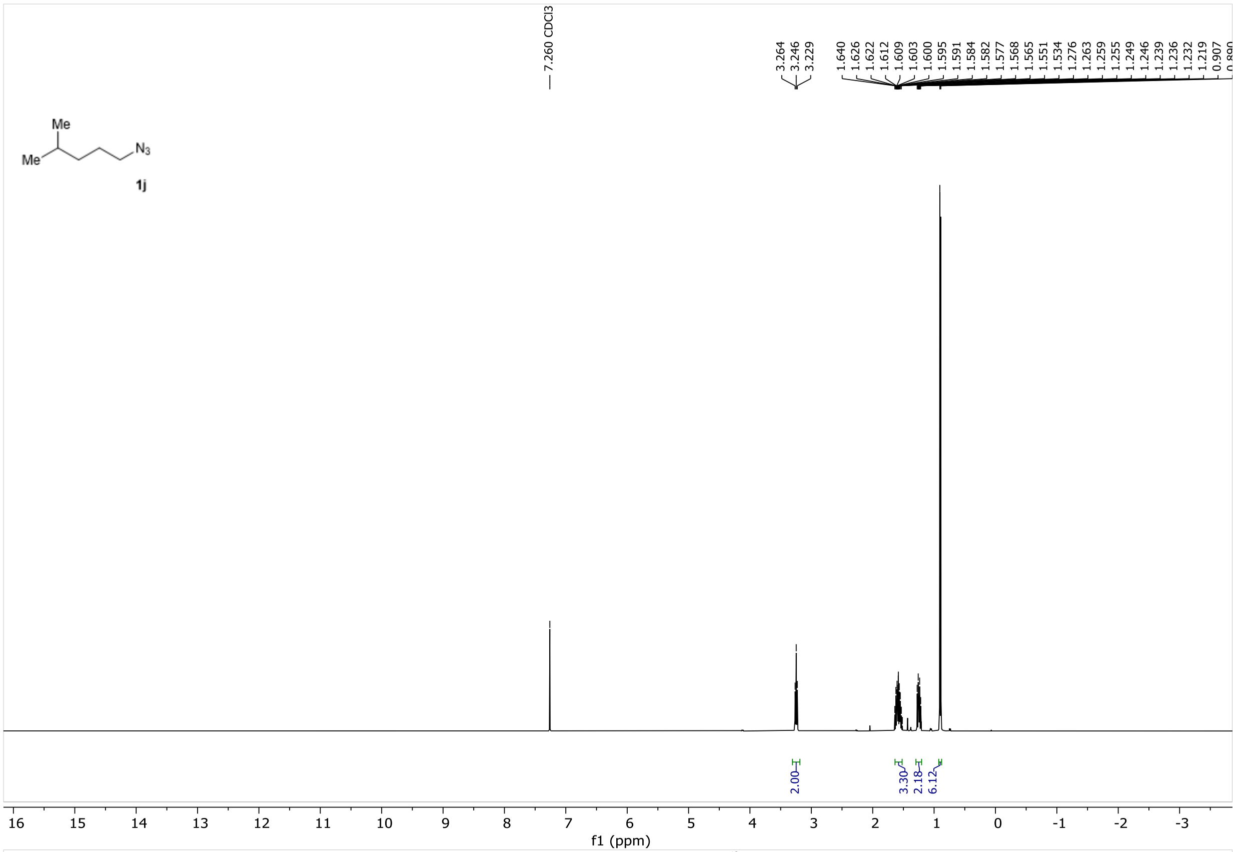

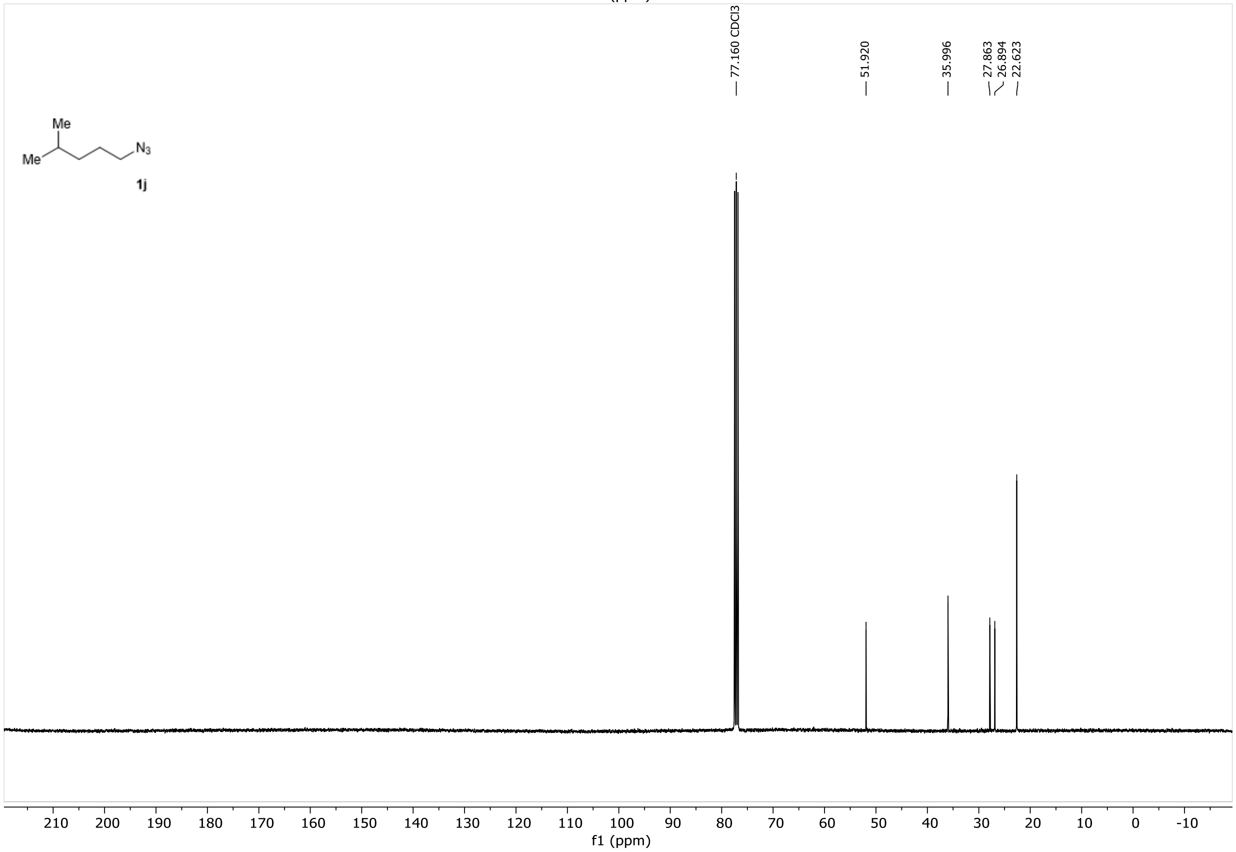

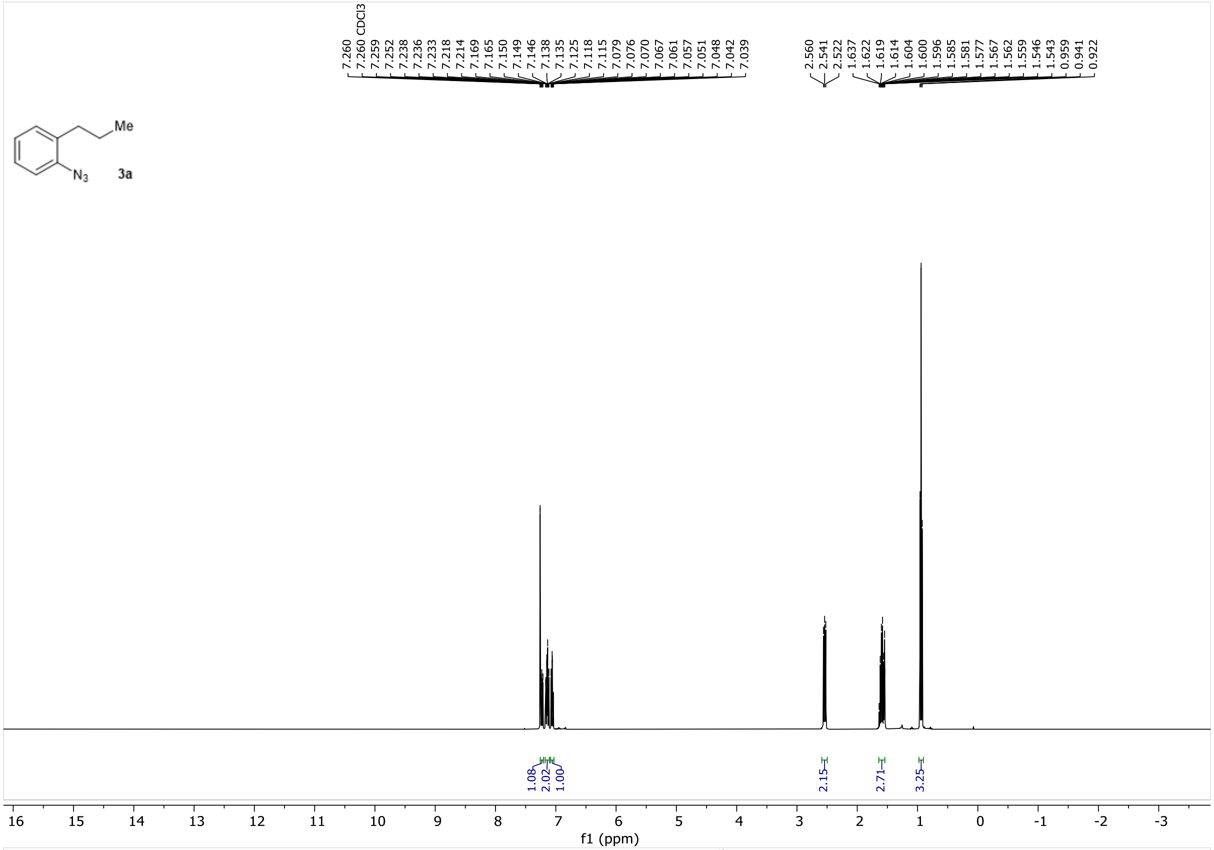
7.260 CDCl3
7.260
7.259
7.252
7.238
7.236
7.233
7.218
7.214
7.169
7.165
7.150
7.149
7.146
7.138
7.135
7.125
7.118
7.115
7.079
7.076
7.070
7.067
7.061
7.057
7.051
7.048
7.042
7.039
2.560
2.541
2.522
1.637
1.622
1.619
1.614
1.604
1.600
1.596
1.585
1.581
1.577
1.567
1.562
1.559
1.546
1.543
0.959
0.941
0.922
Me
N3
3a
1.08
2.02
1.00
2.15
2.71
3.25
f1 (ppm)

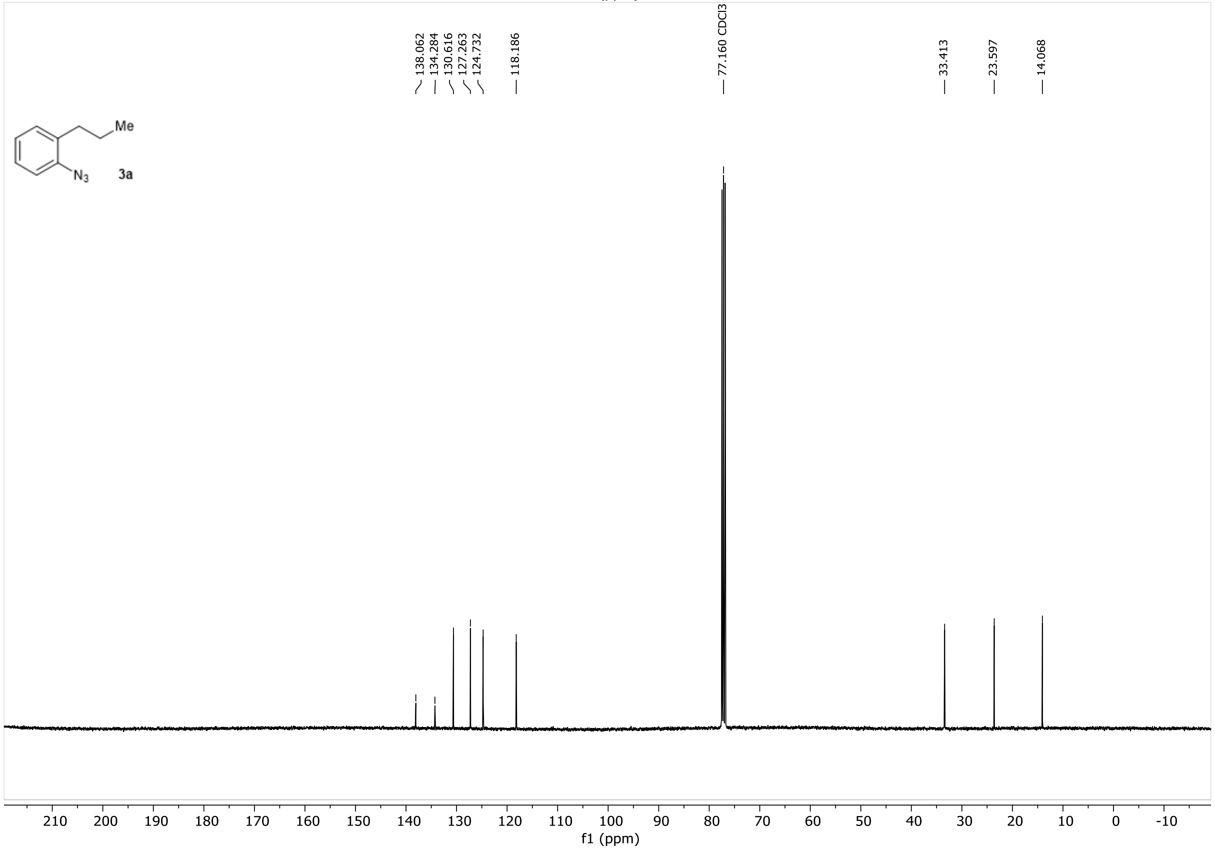
138.062
134.284
130.616
127.263
124.732
118.186
77.160 CDCl3
33.413
23.597
14.068
Me
N3
3a
f1 (ppm)

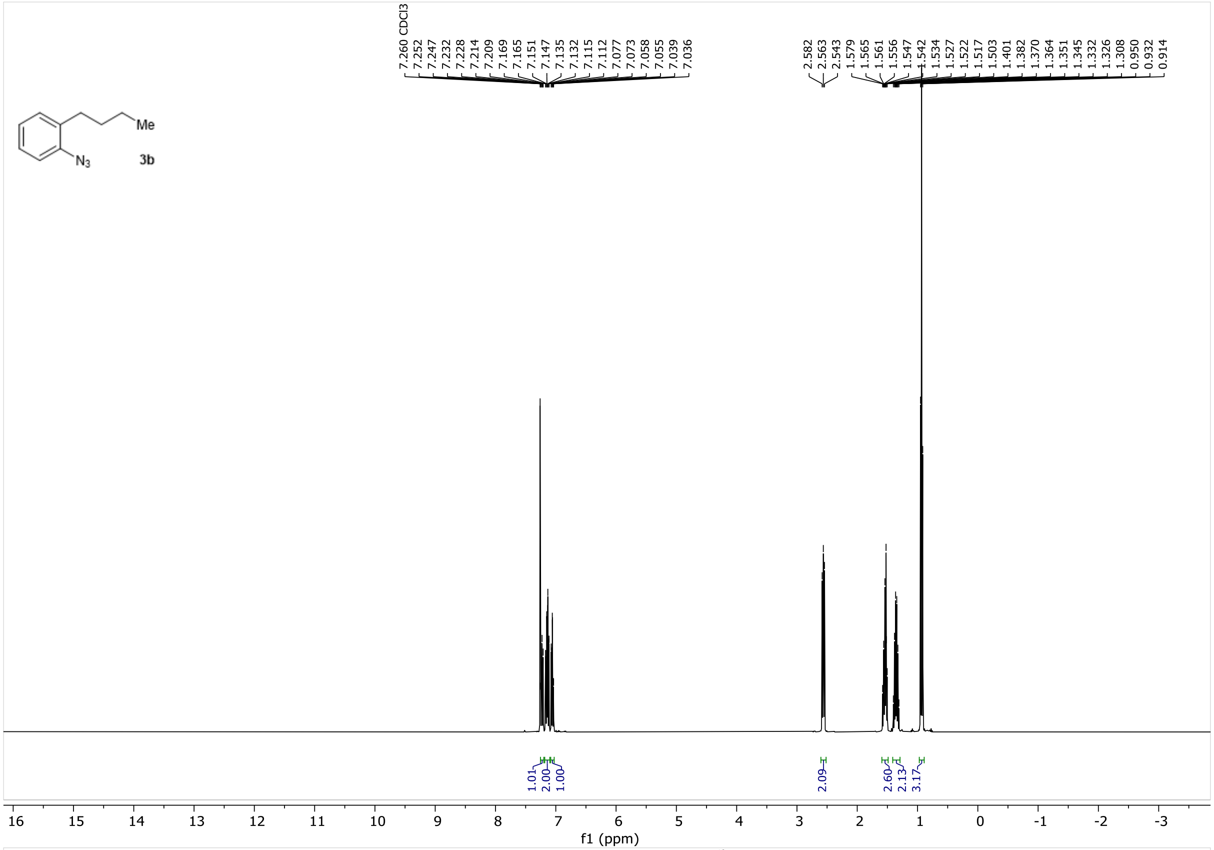
7.260 CDCl3
7.252
7.247
7.232
7.228
7.214
7.209
7.169
7.165
7.151
7.147
7.135
7.132
7.115
7.112
7.077
7.073
7.058
7.055
7.039
7.036
2.582
2.563
2.543
1.579
1.565
1.561
1.556
1.547
1.542
1.534
1.527
1.522
1.517
1.503
1.401
1.382
1.370
1.364
1.351
1.345
1.332
1.326
1.308
0.950
0.932
0.914
Me
N3
3b
1.01
2.00
1.00
2.09
2.60
2.13
3.17
f1 (ppm)

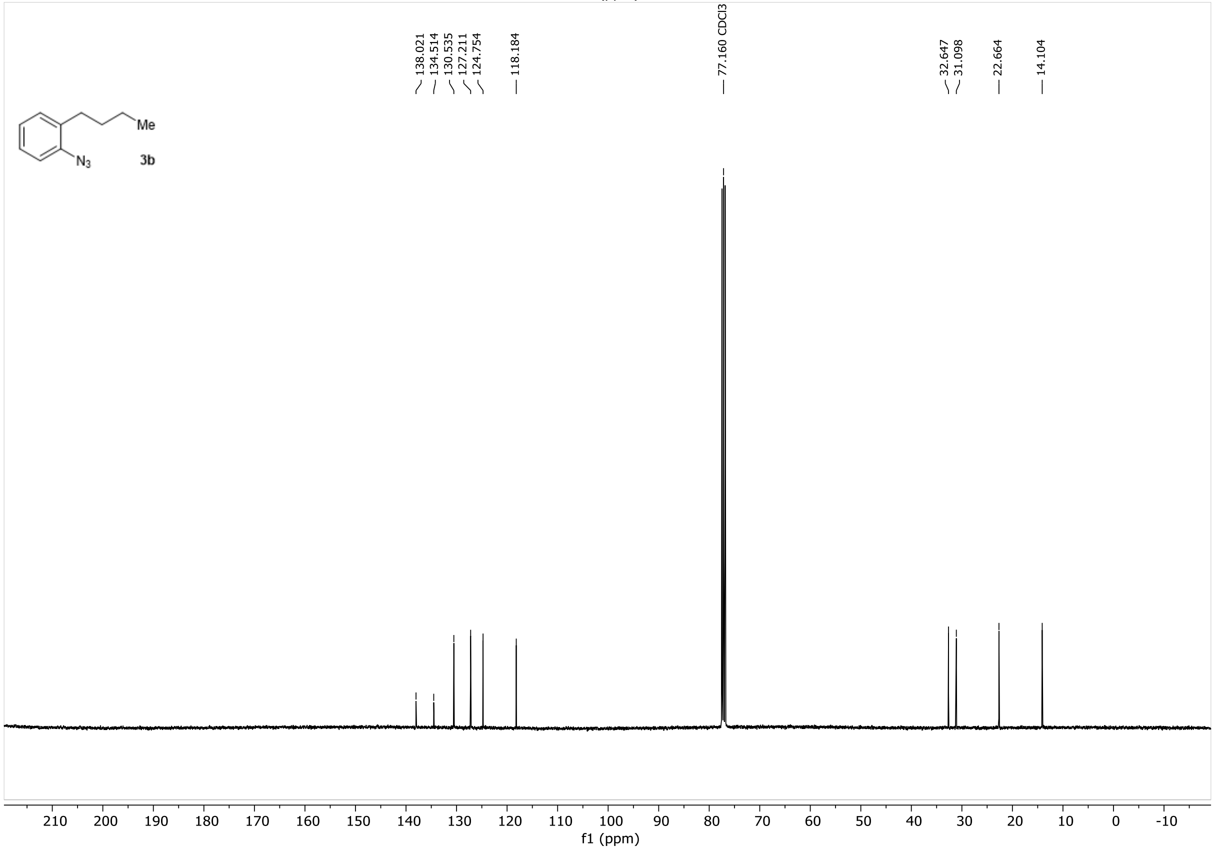
138.021
134.514
130.535
127.211
124.754
118.184
77.160 CDCl3
32.647
31.098
22.664
14.104
Me
N3
3b
f1 (ppm)

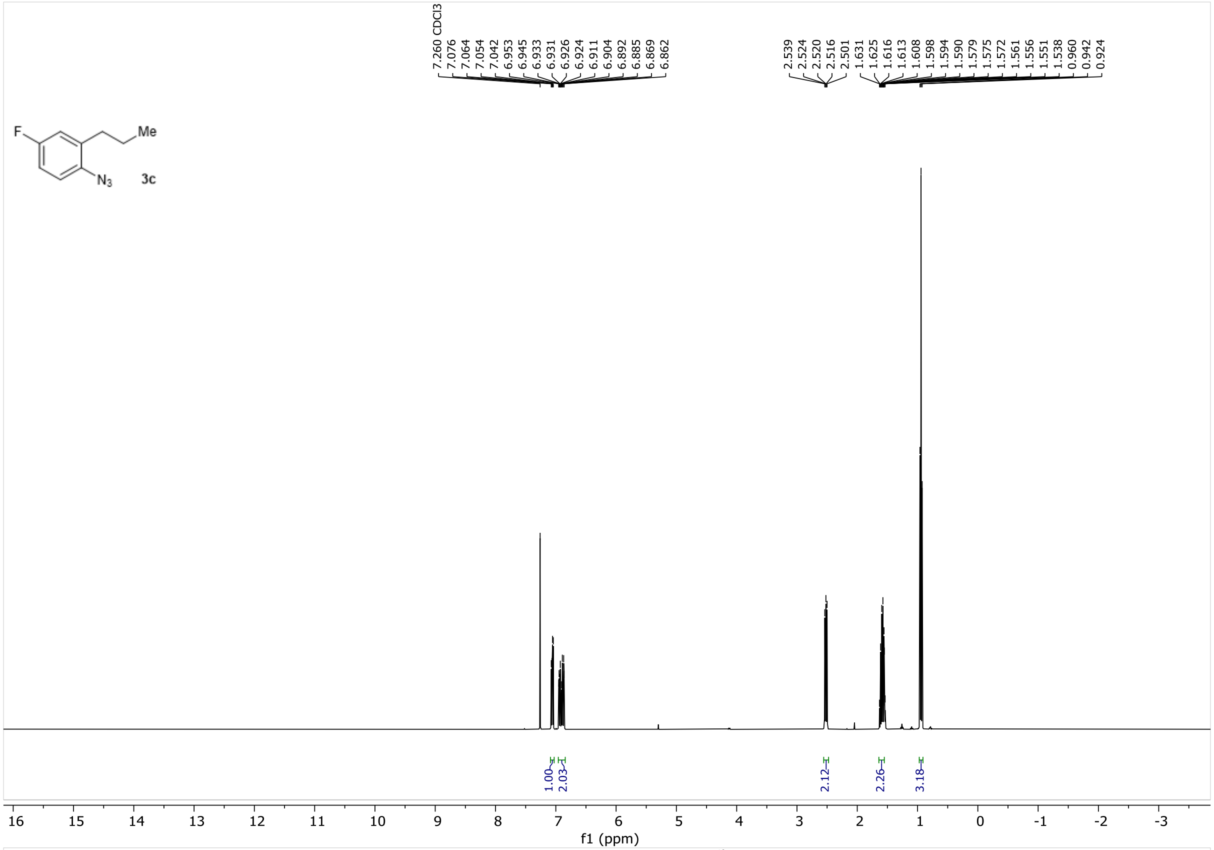

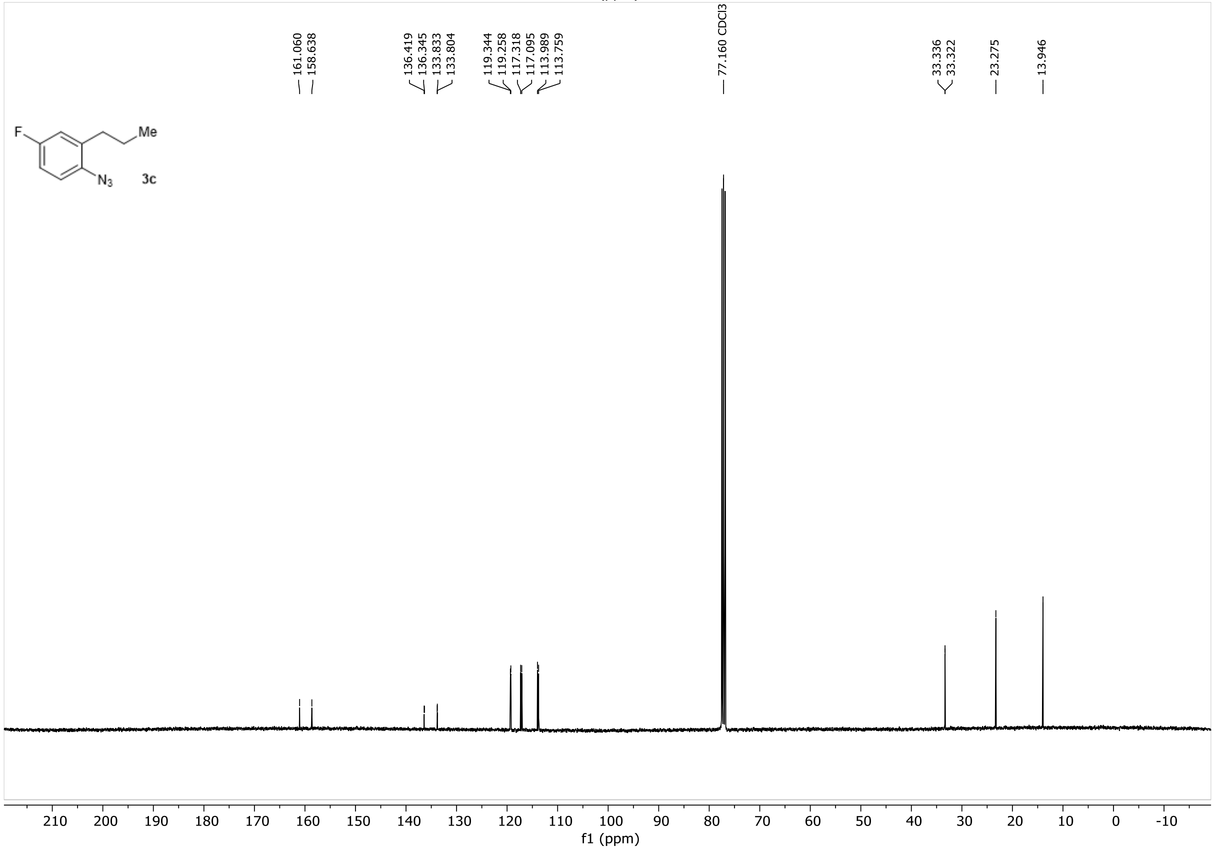

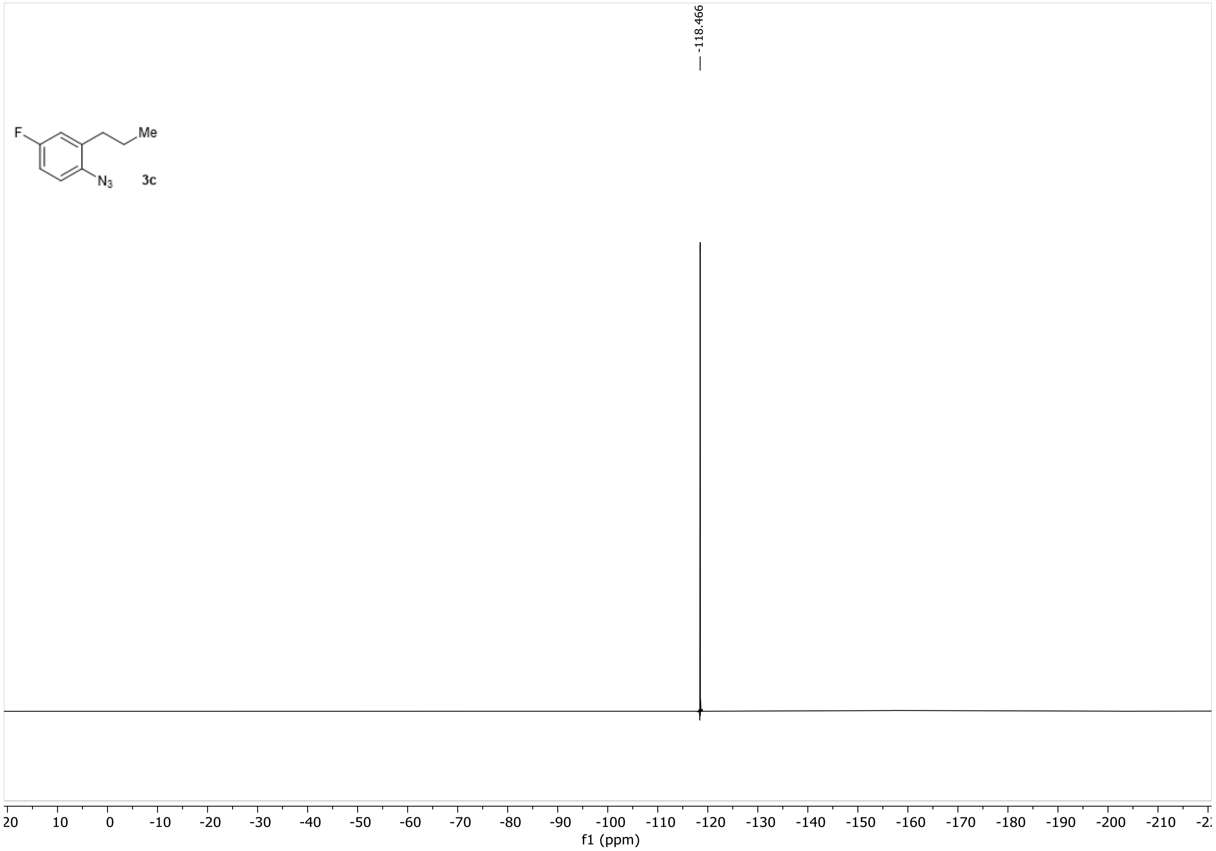
-118.466
F
Me
N3
3c
20 10 0 -10 -20 -30 -40 -50 -60 -70 -80 -90 -100 -110 -120 -130 -140 -150 -160 -170 -180 -190 -200 -210
f1 (ppm)

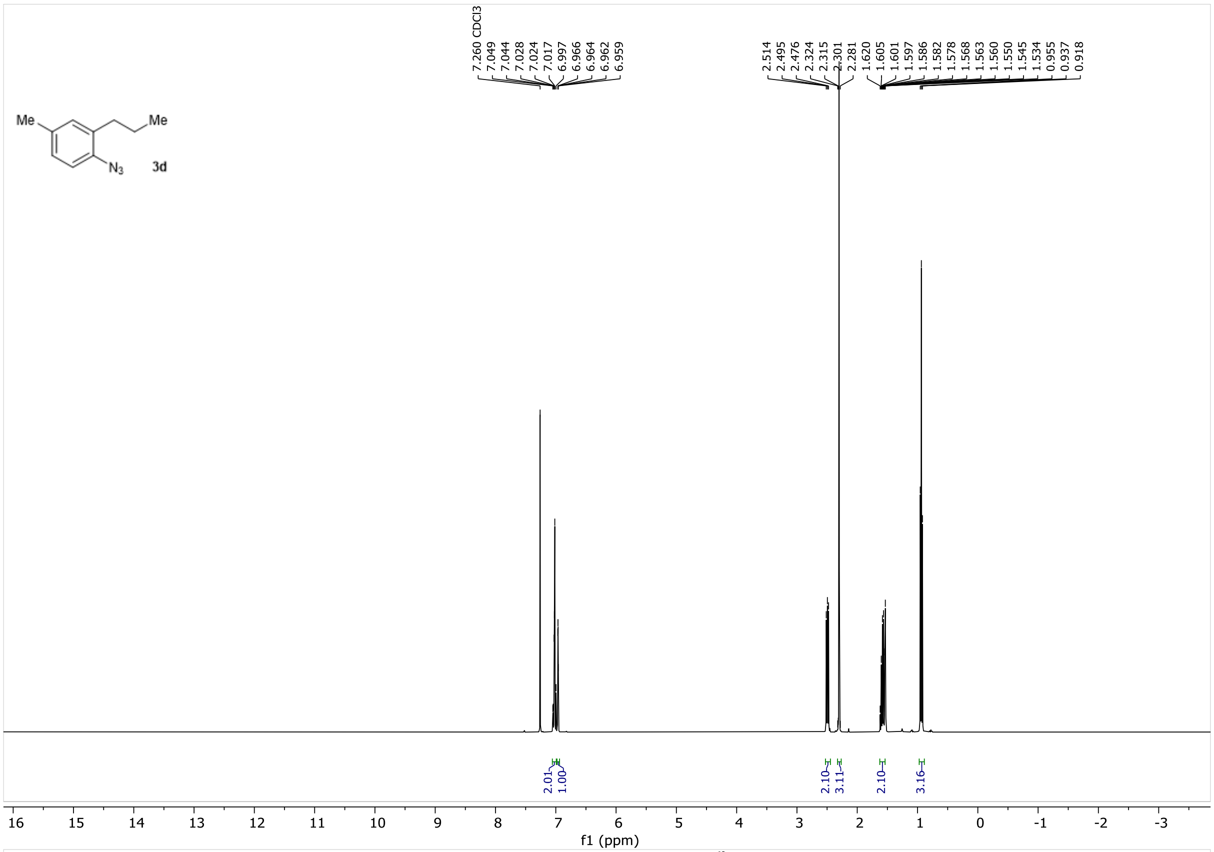
7.260 CDCl3
7.049
7.044
7.028
7.024
7.017
6.997
6.966
6.964
6.962
6.959
2.514
2.495
2.476
2.324
2.315
2.301
2.281
1.620
1.605
1.601
1.597
1.586
1.582
1.578
1.568
1.563
1.560
1.550
1.545
1.534
0.955
0.937
0.918
Me
Me
N3
3d
2.01
1.00
2.10
3.11
2.10
3.16
f1 (ppm)

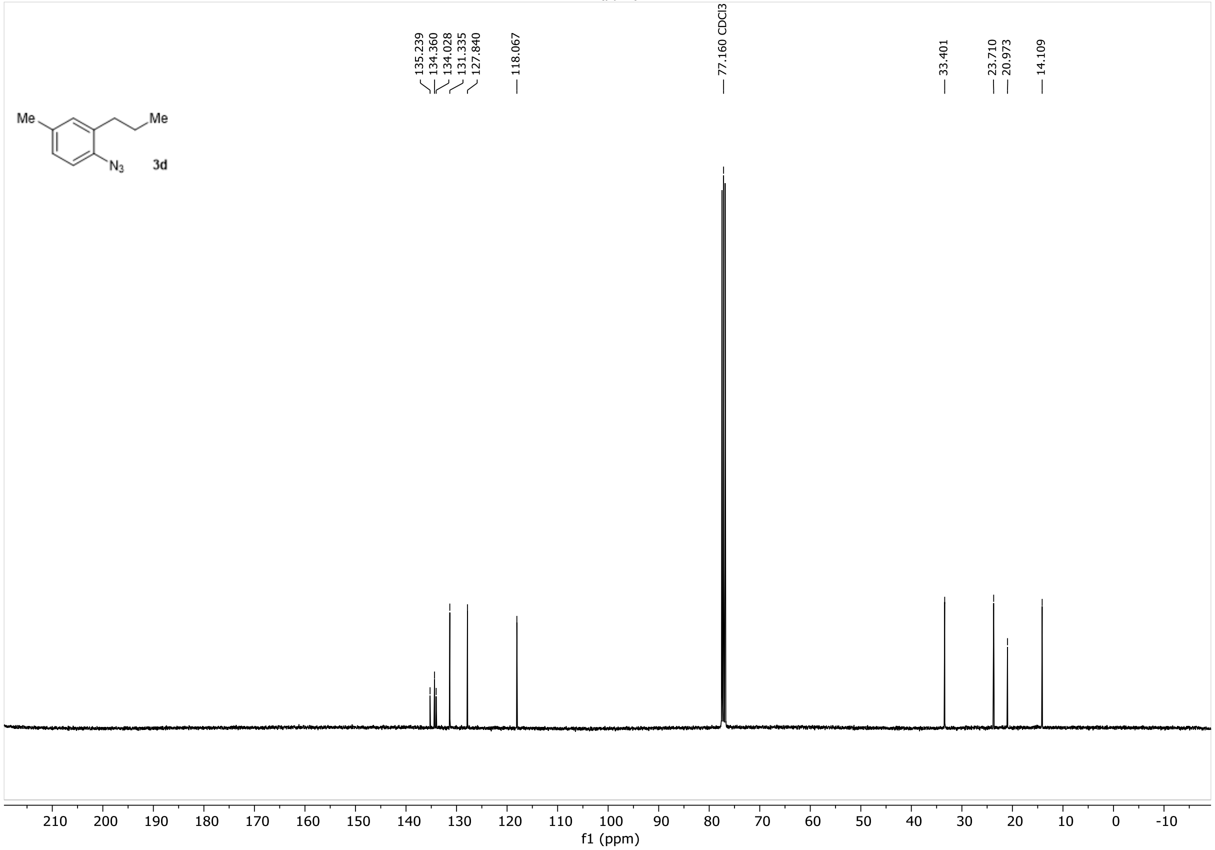
135.239
134.360
134.028
131.335
127.840
118.067
77.160 CDCl3
33.401
23.710
20.973
14.109
Me
Me
N3
3d
f1 (ppm)

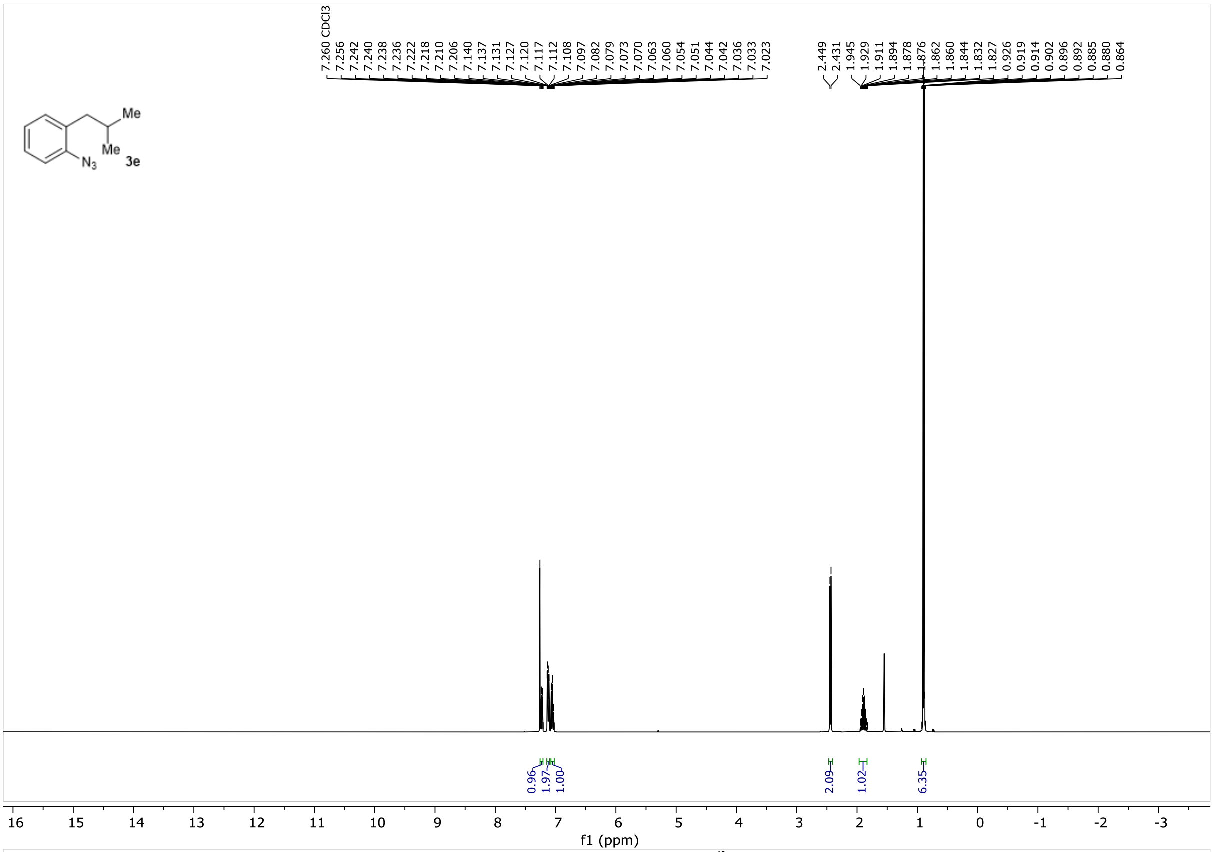
7.260 CDCl3
7.256
7.242
7.240
7.238
7.236
7.222
7.218
7.210
7.206
7.140
7.137
7.131
7.127
7.120
7.117
7.112
7.108
7.097
7.082
7.079
7.073
7.070
7.063
7.060
7.054
7.051
7.044
7.042
7.036
7.033
7.023
2.449
2.431
1.945
1.929
1.911
1.894
1.878
1.876
1.862
1.860
1.844
1.832
1.827
0.926
0.919
0.914
0.902
0.896
0.892
0.885
0.880
0.864
Me
Me
N3
3e
0.96
1.97
1.00
2.09
1.02
6.35
f1 (ppm)

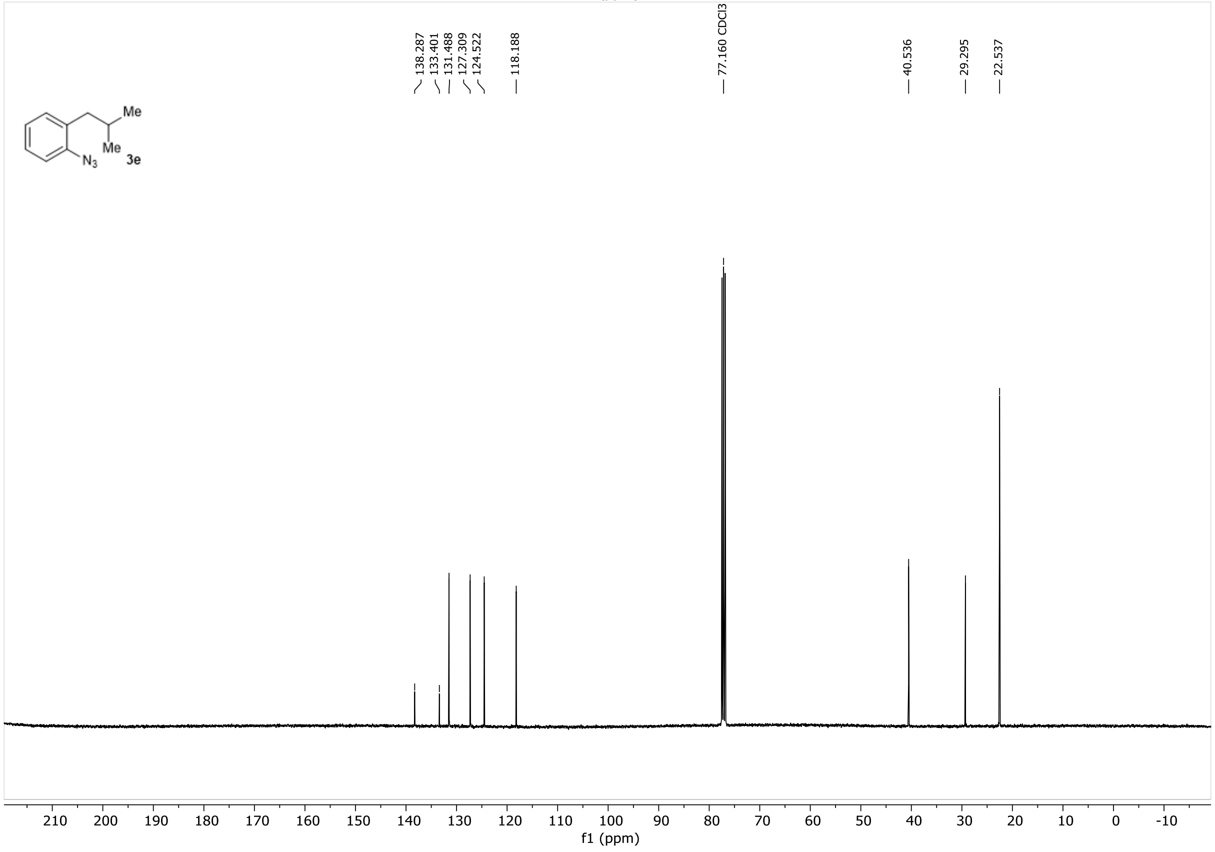
138.287
133.401
131.488
127.309
124.522
118.188
77.160 CDCl3
40.536
29.295
22.537
Me
Me
N3
3e
f1 (ppm)

NMR spectrums for standard products:

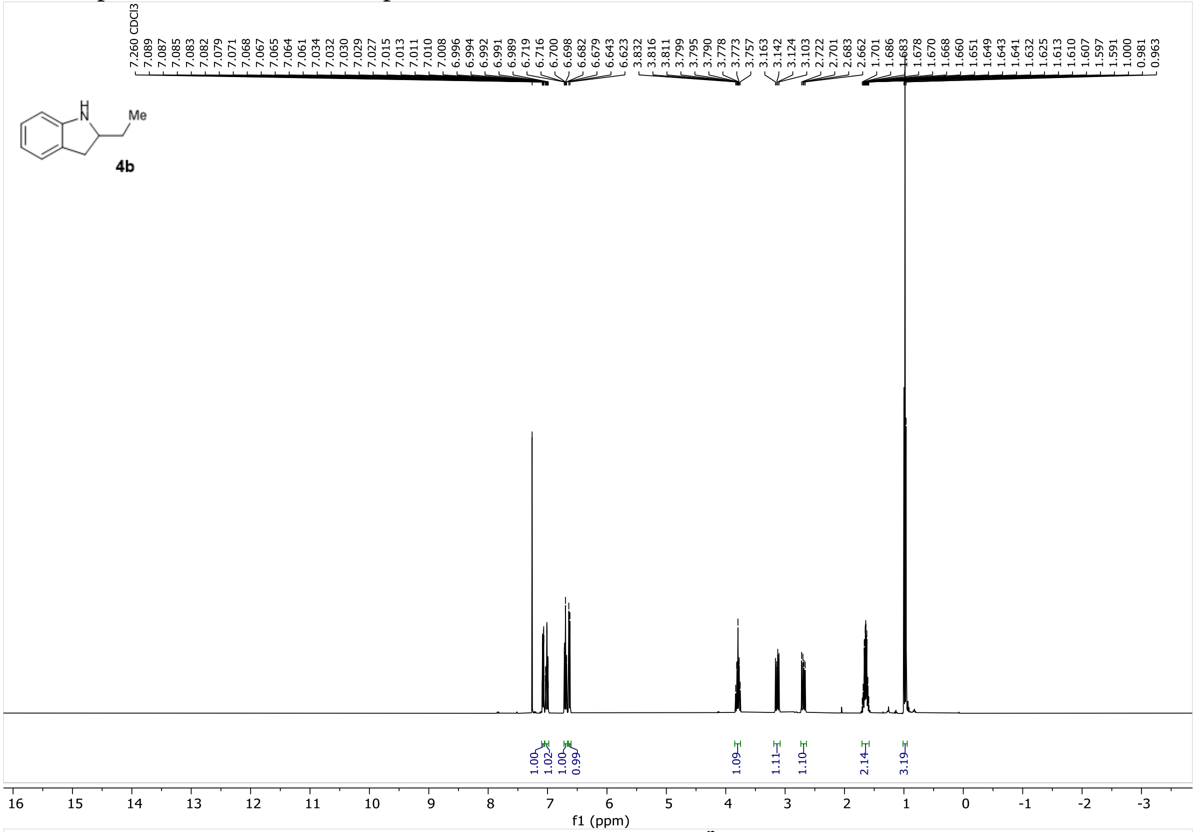

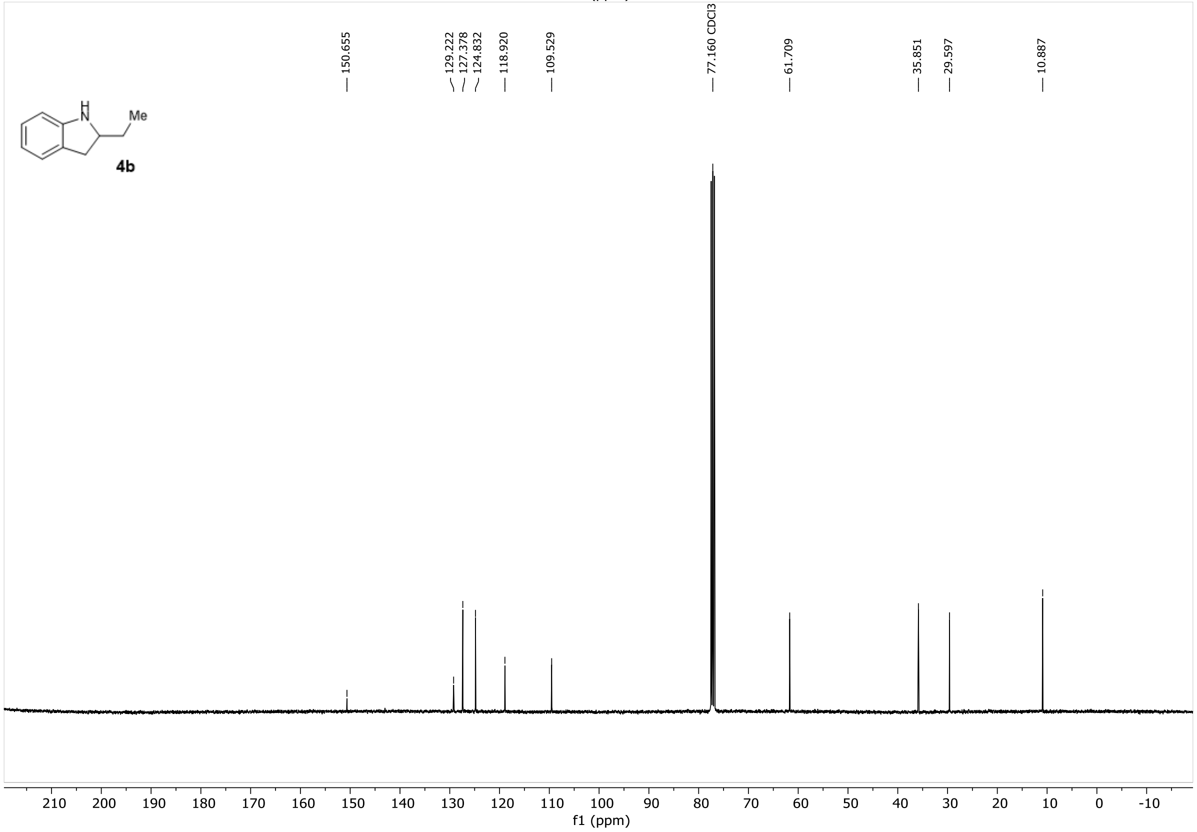

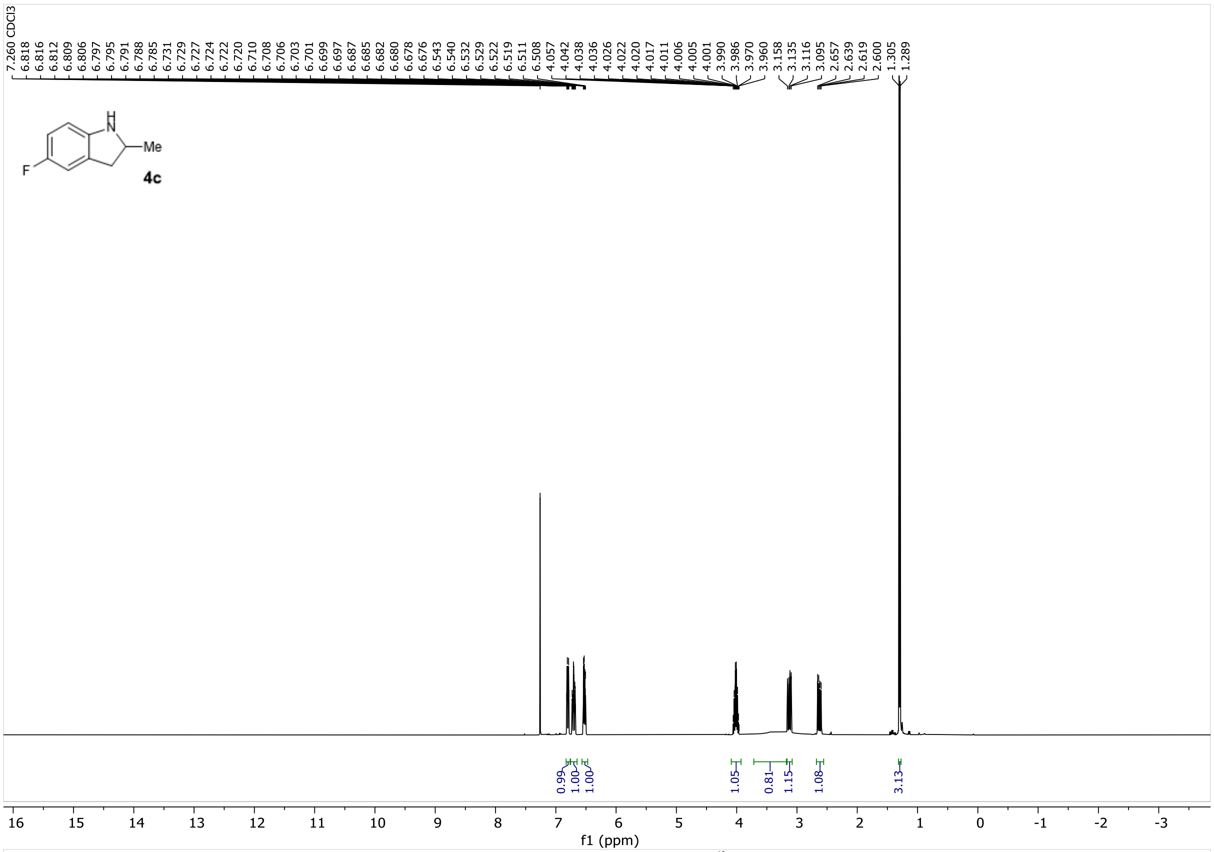
7.260 CDCl3
6.818
6.816
6.812
6.809
6.806
6.797
6.795
6.791
6.788
6.785
6.731
6.729
6.727
6.724
6.722
6.720
6.710
6.708
6.706
6.703
6.701
6.699
6.697
6.687
6.685
6.682
6.680
6.678
6.676
6.543
6.540
6.532
6.529
6.522
6.519
6.511
6.508
4.057
4.042
4.038
4.036
4.026
4.022
4.020
4.017
4.011
4.006
4.005
4.001
3.990
3.986
3.970
3.960
3.158
3.135
3.116
3.095
2.657
2.639
2.619
2.600
1.305
1.289
H
N
Me
F
4c
0.99
1.00
1.00
1.05
0.81
1.15
1.08
3.13
16 15 14 13 12 11 10 9 8 7 6 5 4 3 2 1 0 -1 -2 -3
f1 (ppm)

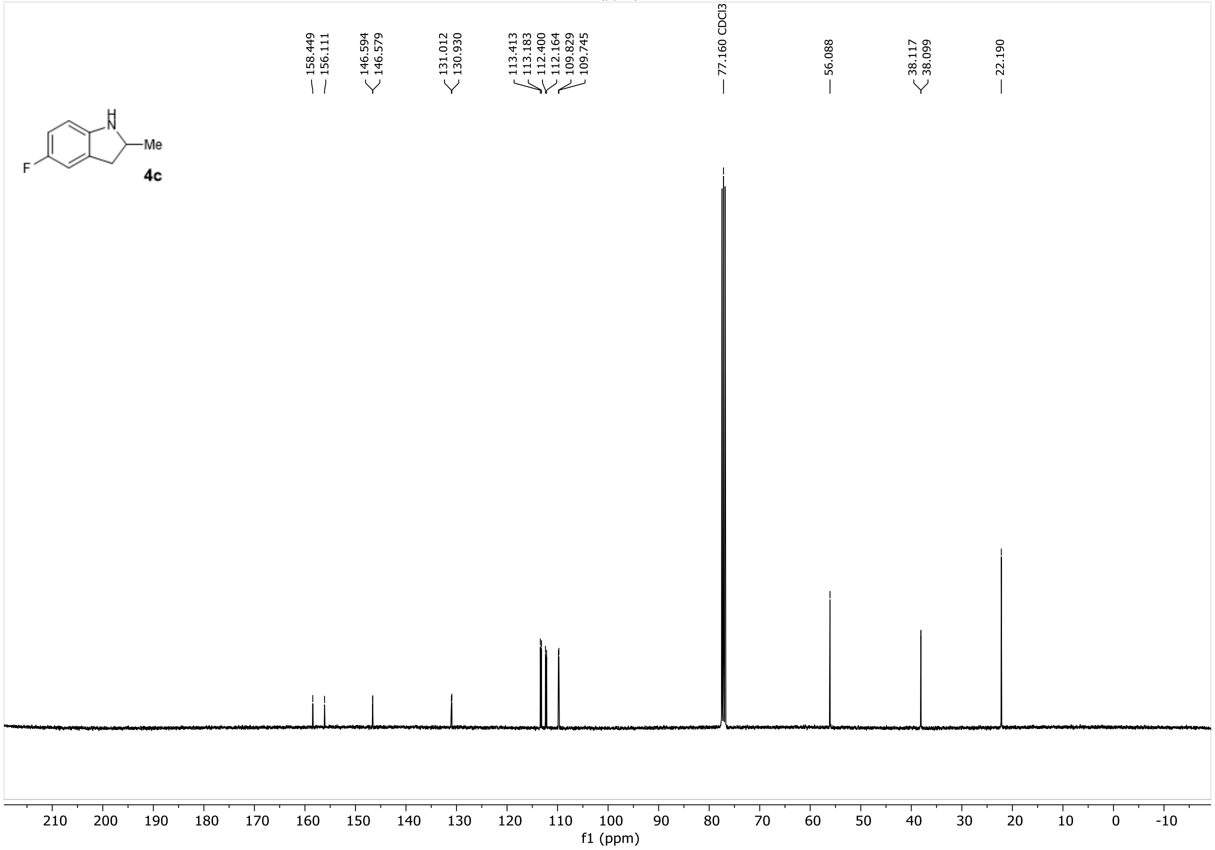
158.449
156.111
146.594
146.579
131.012
130.930
113.413
113.183
112.400
112.164
109.829
109.745
77.160 CDCl3
56.088
38.117
38.099
22.190
H
N
Me
F
4c
210 200 190 180 170 160 150 140 130 120 110 100 90 80 70 60 50 40 30 20 10 0 -10
f1 (ppm)

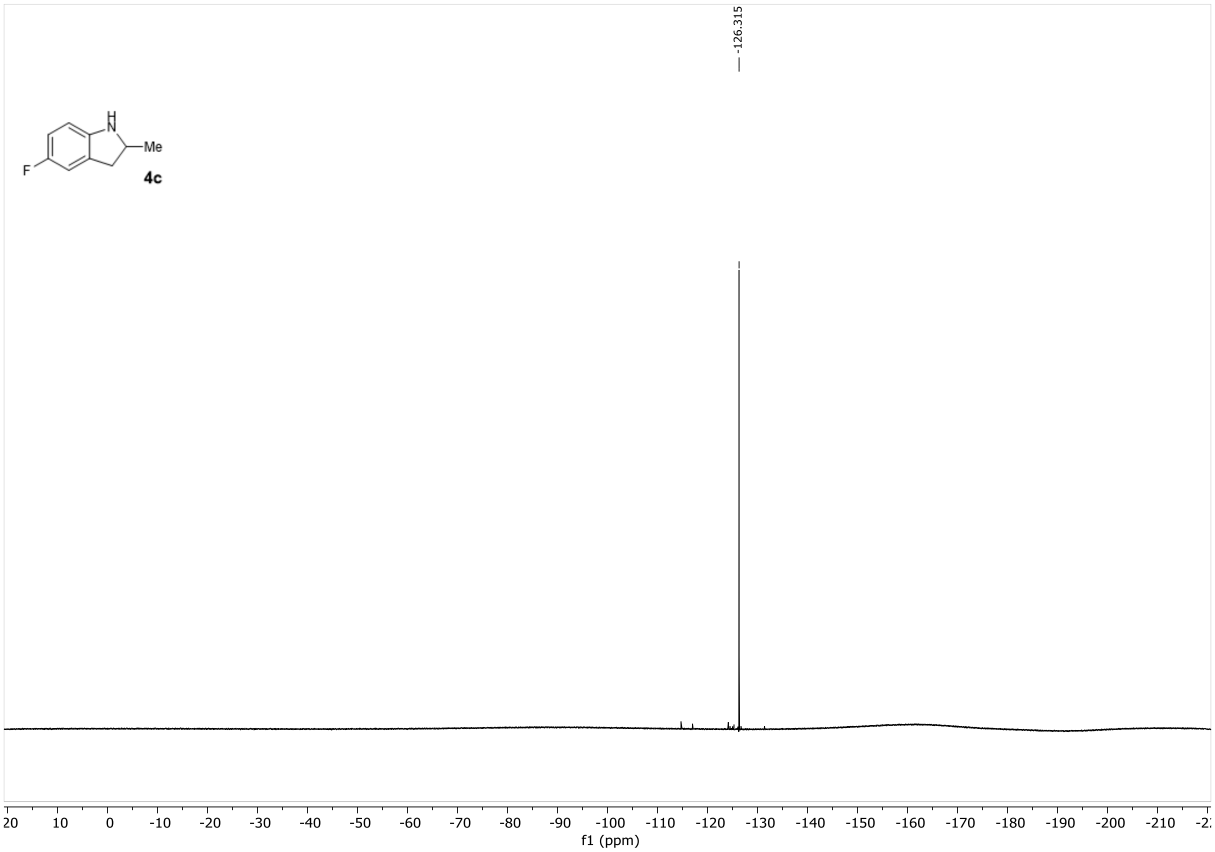
-126.315
Me
F
4c
20
10
0
-10
-20
-30
-40
-50
-60
-70
-80
-90
-100
-110
-120
-130
-140
-150
-160
-170
-180
-190
-200
-210
f1 (ppm)

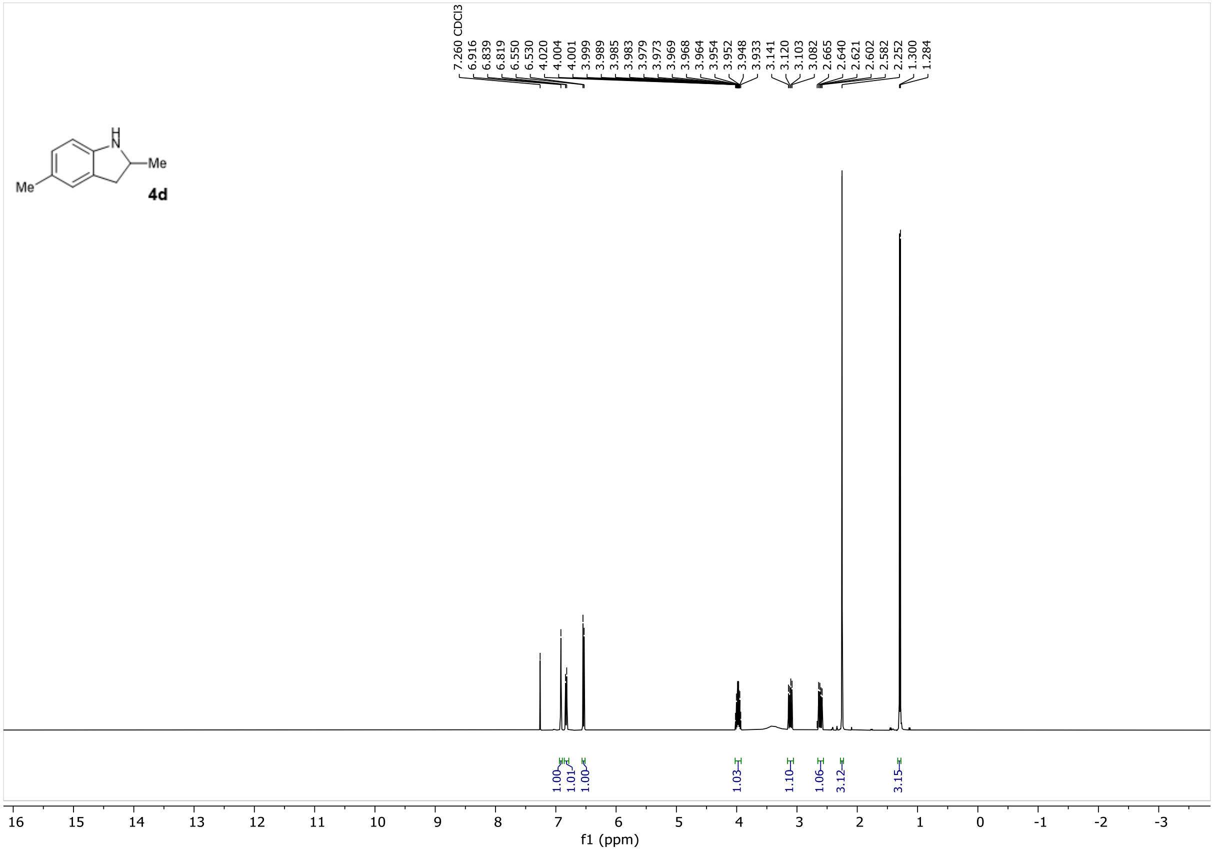
7.260 CDCl3
6.916
6.839
6.819
6.550
6.530
4.020
4.004
4.001
3.999
3.989
3.985
3.983
3.979
3.973
3.969
3.968
3.964
3.954
3.952
3.948
3.933
3.141
3.120
3.103
3.082
2.665
2.640
2.621
2.602
2.582
2.252
1.300
1.284
Me
Me
4d
1.00
1.01
1.00
1.03
1.10
1.06
3.12
3.15
16 15 14 13 12 11 10 9 8 7 6 5 4 3 2 1 0 -1 -2 -3
f1 (ppm)

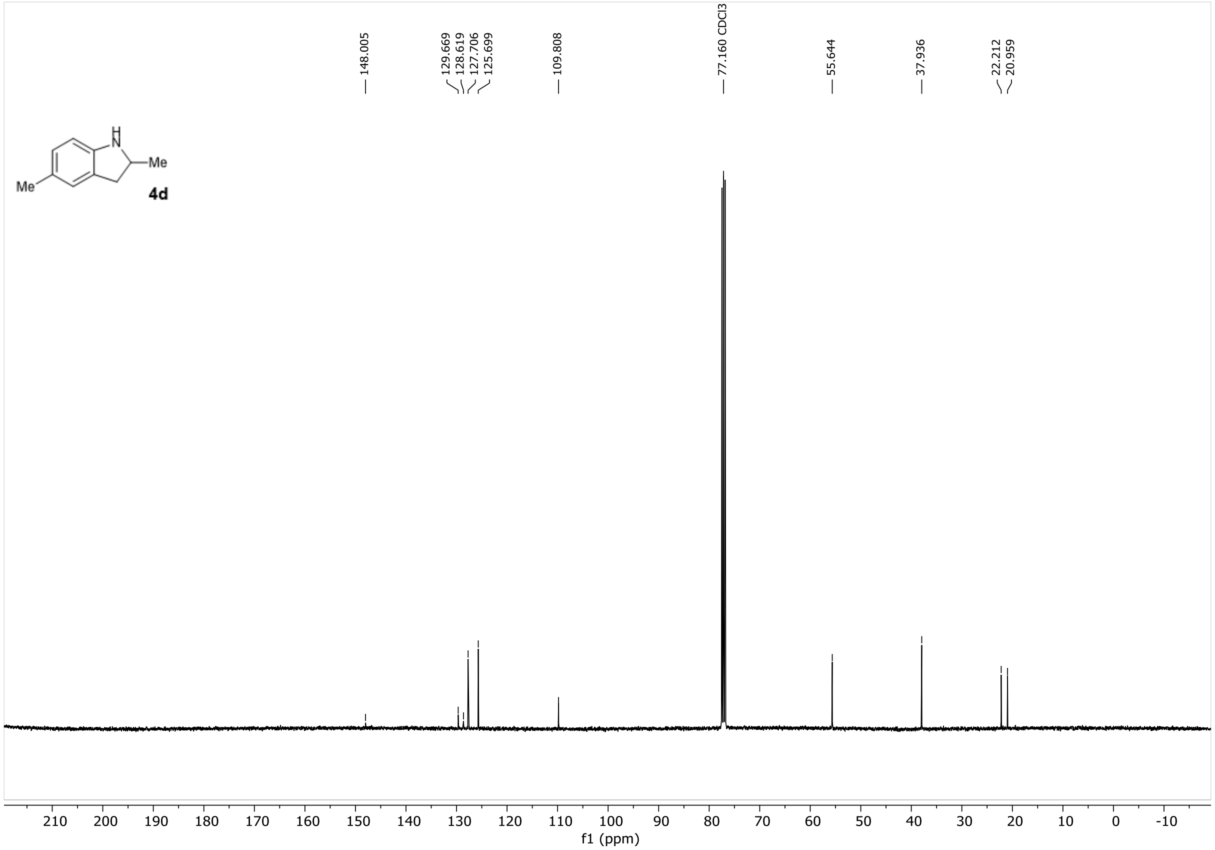
148.005
129.669
128.619
127.706
125.699
109.808
77.160 CDCl3
55.644
37.936
22.212
20.959
Me
Me
4d
f1 (ppm)

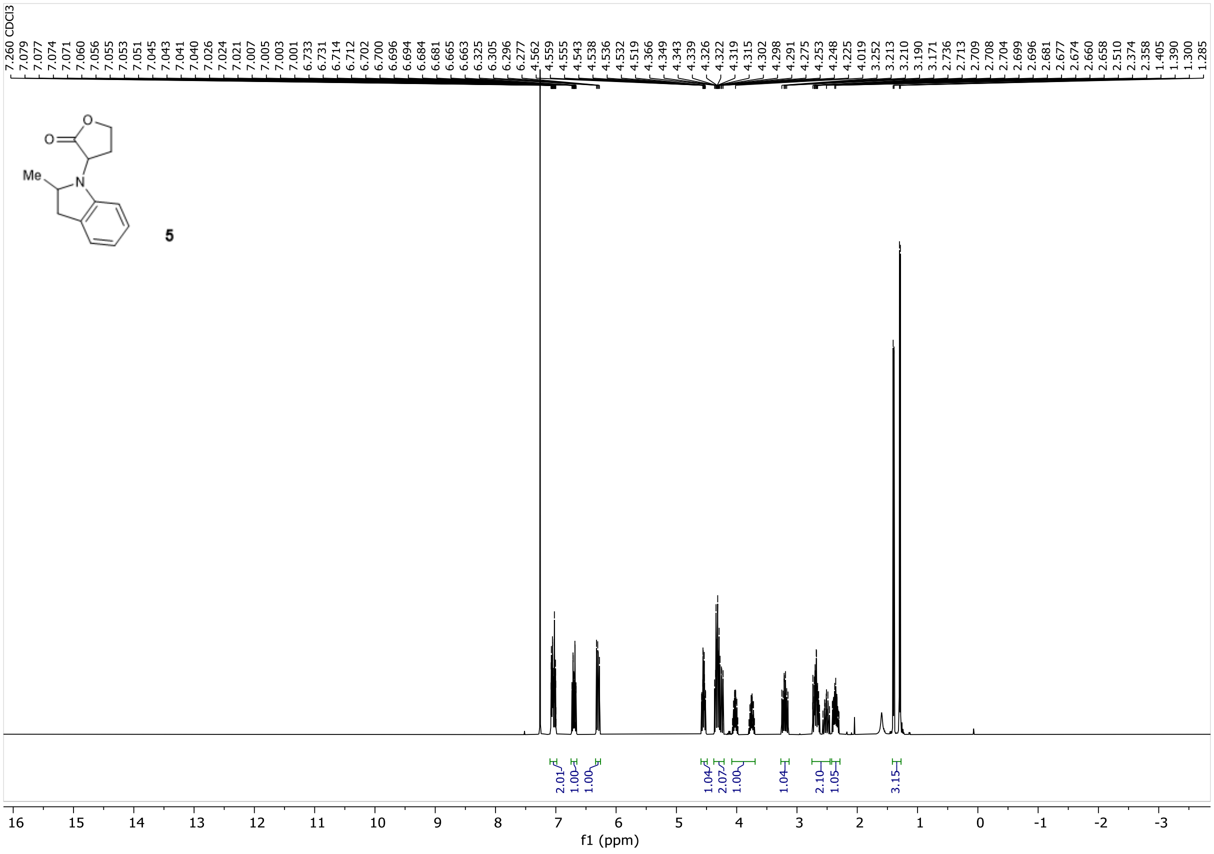
7.260 CDCl3
7.079
7.077
7.074
7.071
7.060
7.056
7.055
7.053
7.051
7.045
7.043
7.041
7.040
7.026
7.024
7.021
7.007
7.005
7.003
7.001
6.733
6.731
6.714
6.712
6.702
6.700
6.696
6.694
6.684
6.681
6.665
6.663
6.325
6.305
6.296
6.277
4.562
4.559
4.555
4.543
4.538
4.536
4.532
4.519
4.366
4.349
4.343
4.339
4.326
4.322
4.319
4.315
4.302
4.298
4.291
4.275
4.253
4.248
4.225
4.019
3.252
3.213
3.210
3.190
3.171
2.736
2.713
2.709
2.708
2.704
2.699
2.696
2.681
2.677
2.674
2.660
2.658
2.510
2.374
2.358
1.405
1.390
1.300
1.285
O
O
Me
N
5
2.01
1.00
1.00
1.04
2.07
1.00
1.04
2.10
1.05
3.15
f1 (ppm)

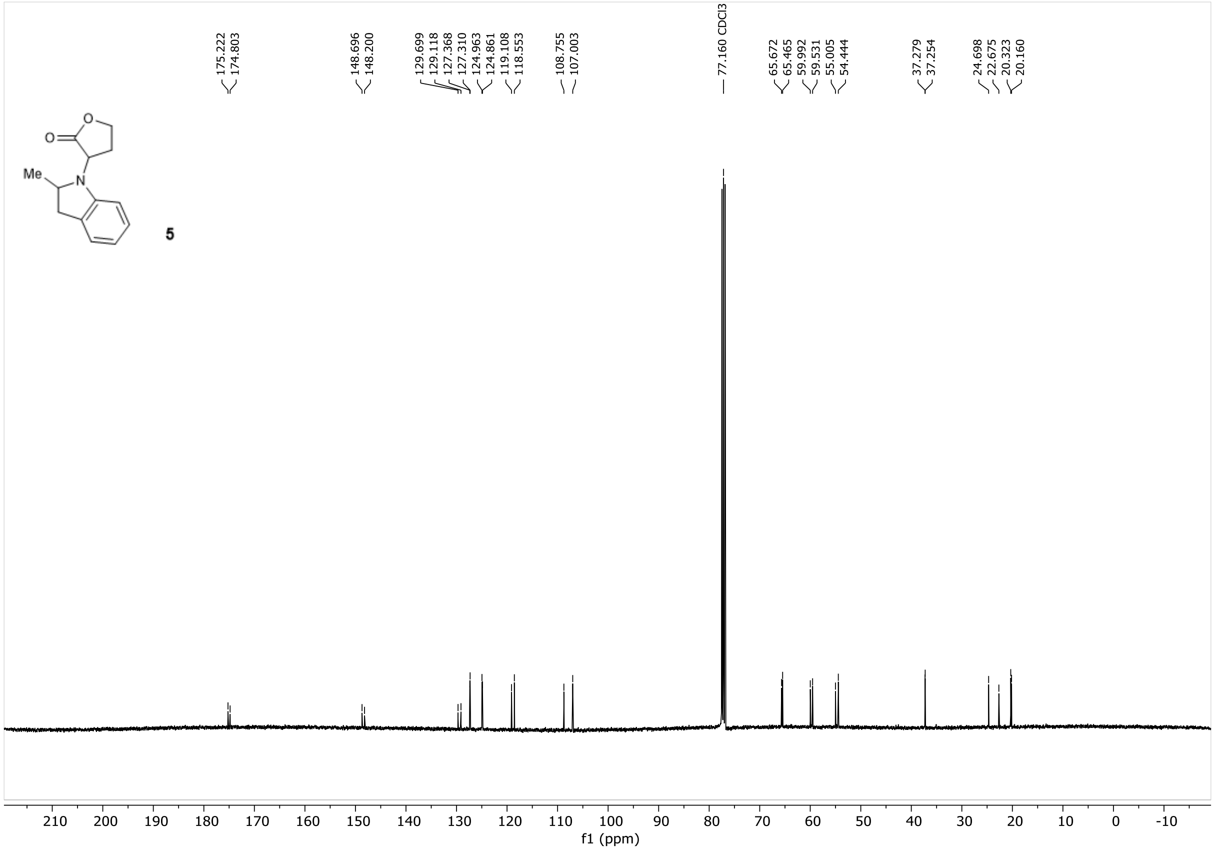

NMR for the preparative-scale enzymatic reaction:

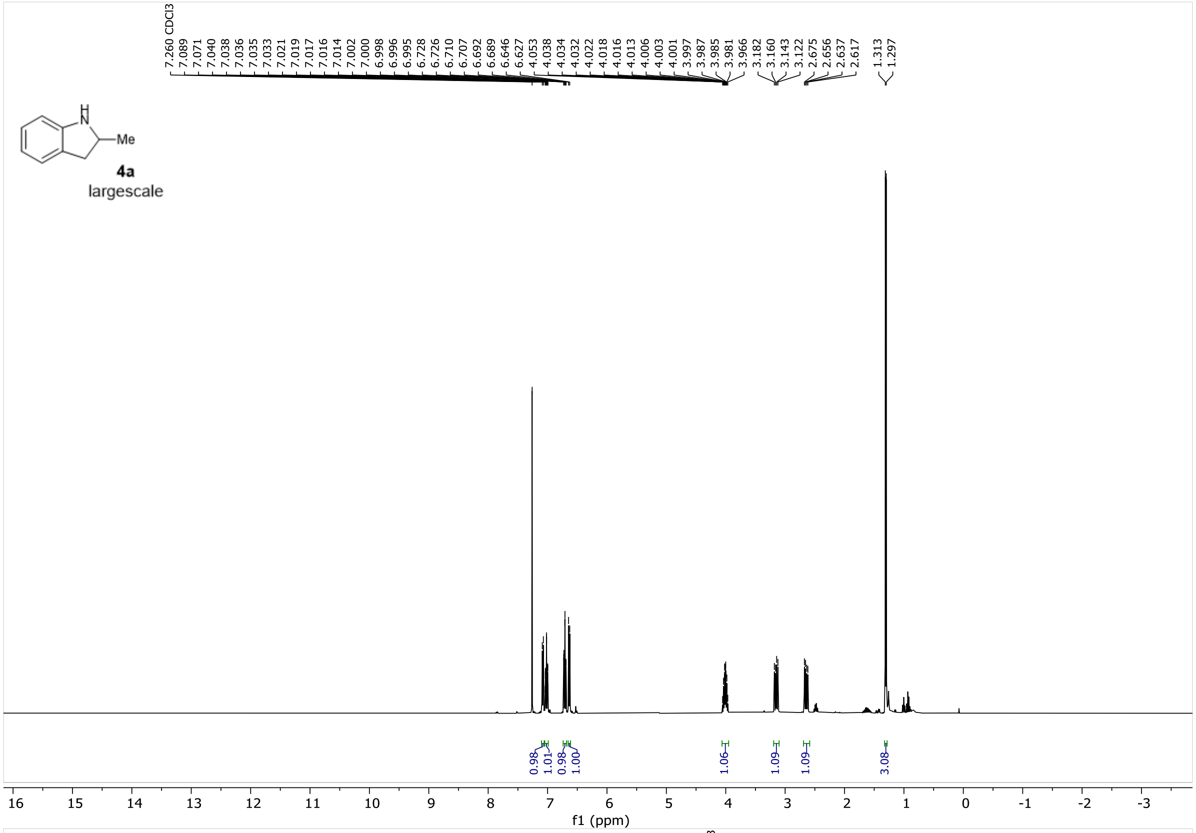
7.260 CDCl3
7.089
7.071
7.040
7.038
7.036
7.035
7.033
7.021
7.019
7.017
7.016
7.014
7.002
7.000
6.998
6.996
6.995
6.728
6.726
6.710
6.707
6.692
6.689
6.646
6.627
4.053
4.038
4.034
4.032
4.022
4.018
4.016
4.013
4.006
4.003
4.001
3.997
3.987
3.985
3.981
3.966
3.182
3.160
3.143
3.122
2.675
2.656
2.637
2.617
1.313
1.297
Me
4a
largescale
0.98
1.01
0.98
1.00
1.06
1.09
1.09
3.08
f1 (ppm)

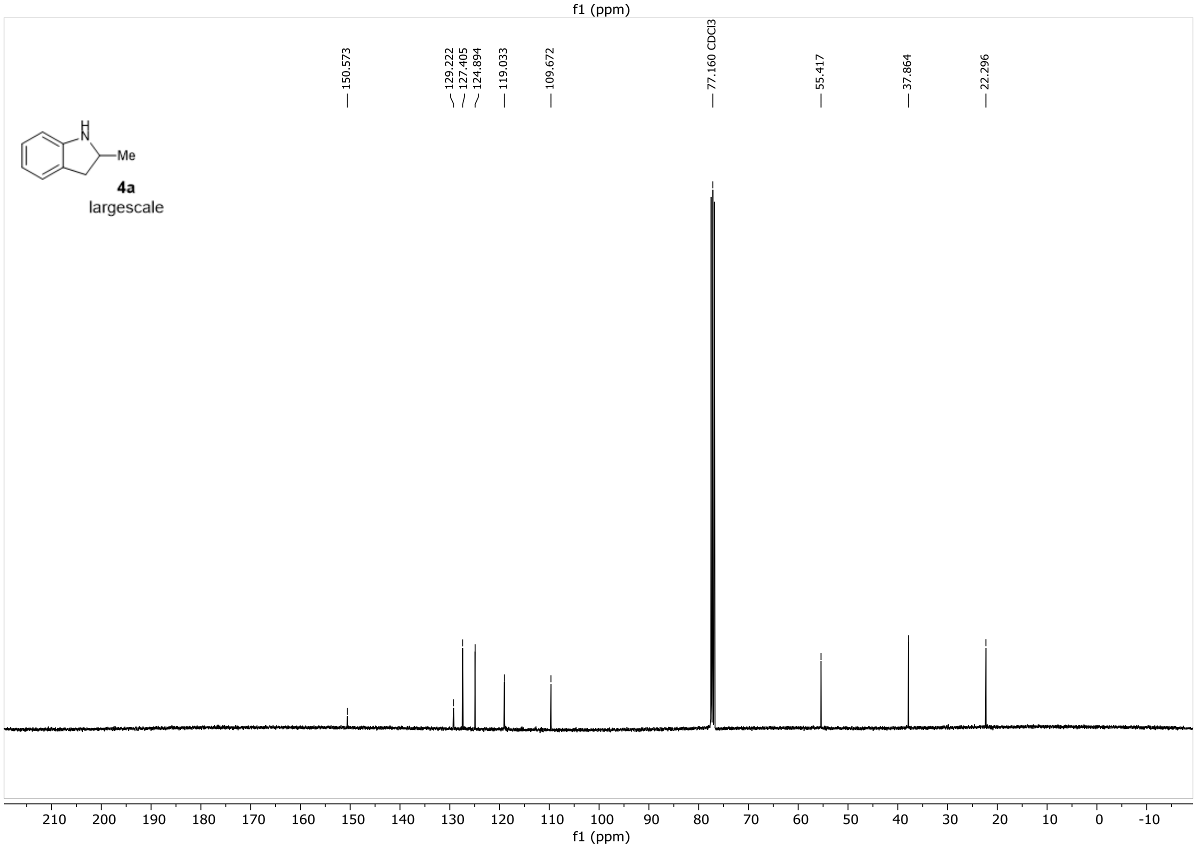
150.573
129.222
127.405
124.894
119.033
109.672
77.160 CDCl3
55.417
37.864
22.296
Me
4a
largescale
f1 (ppm)

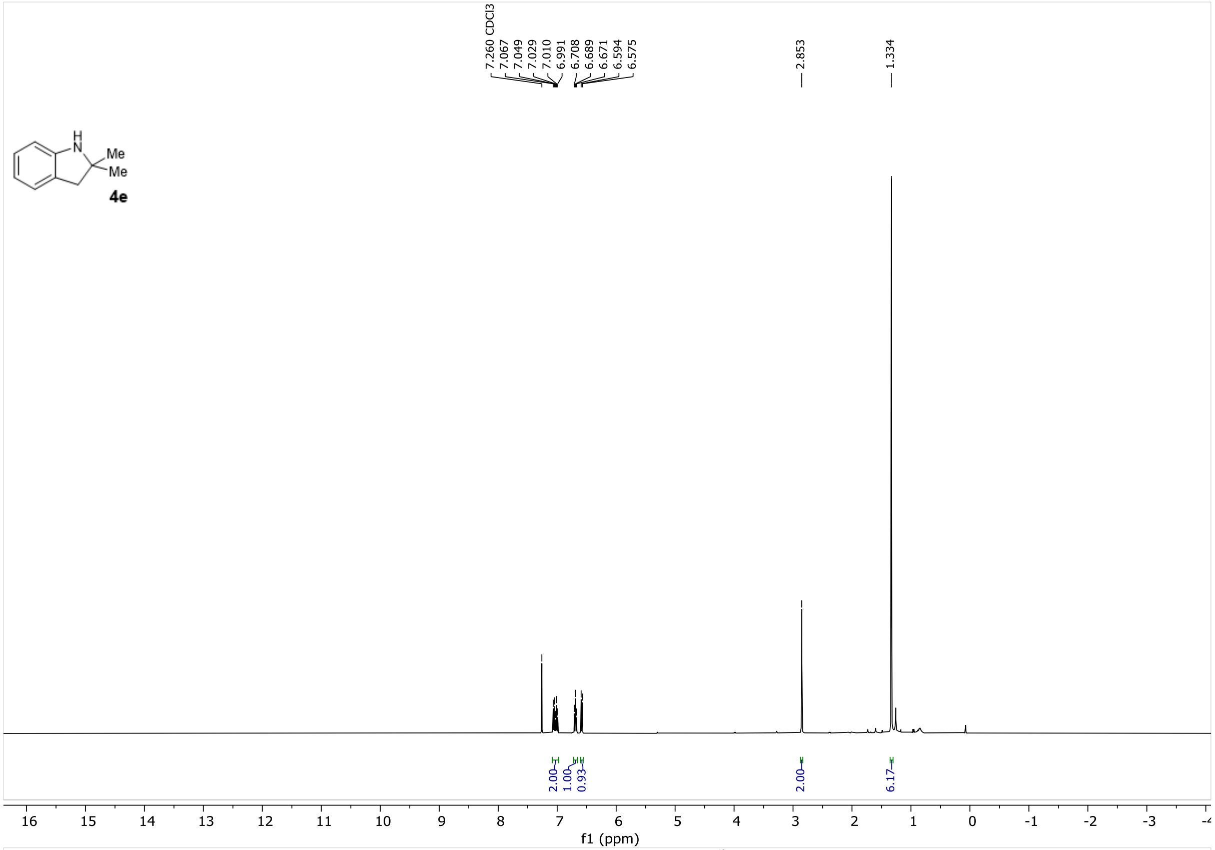
7.260 CDCl3
7.067
7.049
7.029
7.010
6.991
6.708
6.689
6.671
6.594
6.575
2.853
1.334
H
N
Me
Me
4e
2.00
1.00
0.93
2.00
6.17
16 15 14 13 12 11 10 9 8 7 6 5 4 3 2 1 0 -1 -2 -3
f1 (ppm)

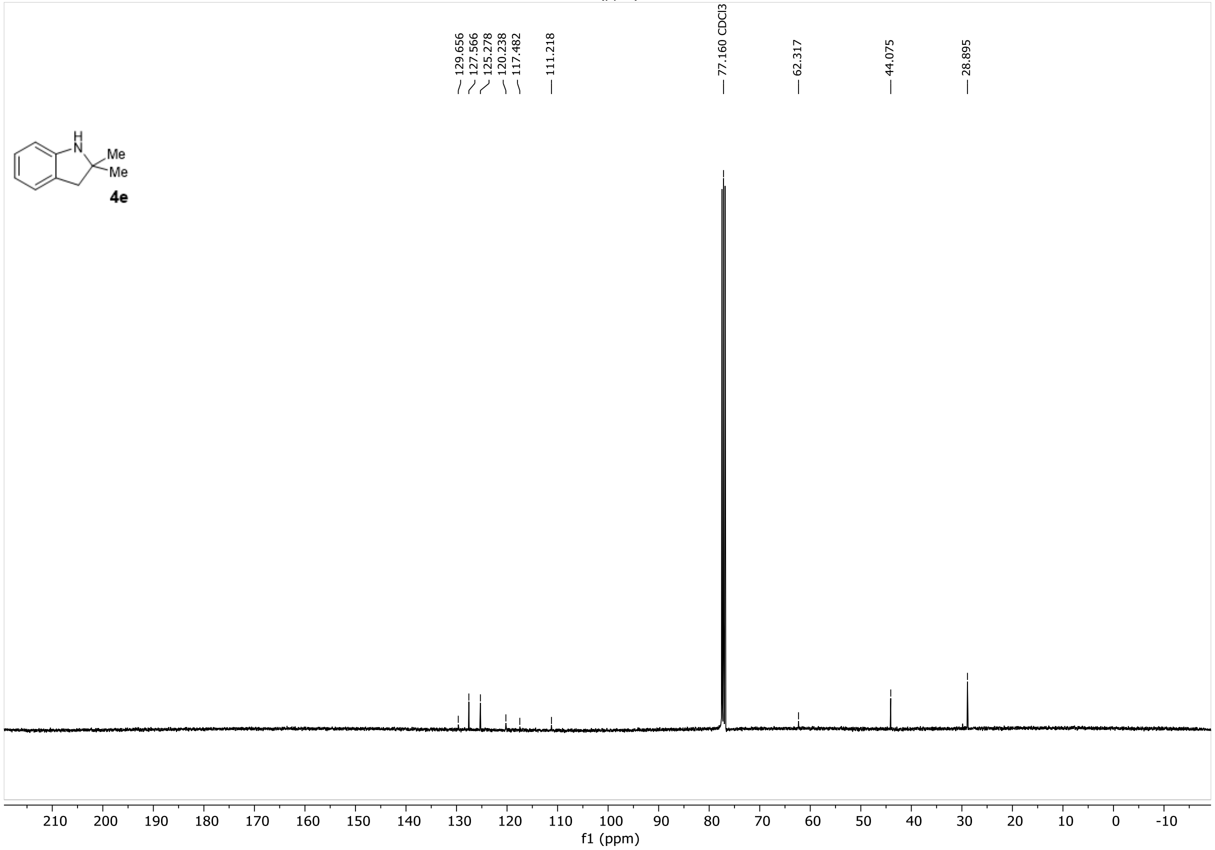
129.656
127.566
125.278
120.238
117.482
111.218
77.160 CDCl3
62.317
44.075
28.895
H
N
Me
Me
4e
210 200 190 180 170 160 150 140 130 120 110 100 90 80 70 60 50 40 30 20 10 0 -10
f1 (ppm)

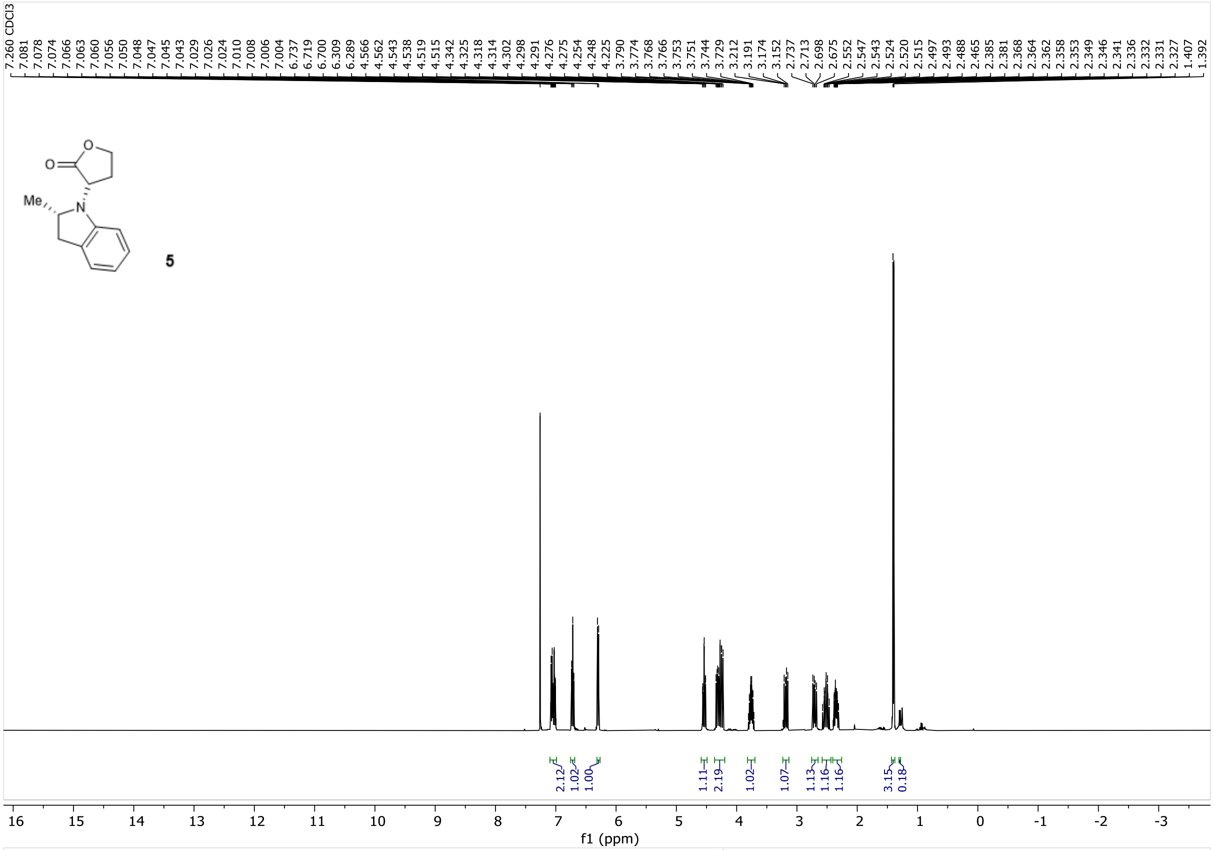
7.260 CDCl3
7.081
7.078
7.074
7.066
7.063
7.060
7.056
7.050
7.048
7.047
7.045
7.043
7.029
7.026
7.024
7.010
7.008
7.006
7.004
6.737
6.719
6.700
6.309
6.289
4.566
4.562
4.543
4.538
4.519
4.515
4.342
4.325
4.318
4.314
4.302
4.298
4.291
4.276
4.275
4.254
4.248
4.225
3.790
3.774
3.768
3.766
3.753
3.751
3.744
3.729
3.212
3.191
3.174
3.152
2.737
2.713
2.698
2.675
2.552
2.547
2.543
2.524
2.520
2.515
2.497
2.493
2.488
2.465
2.385
2.381
2.368
2.364
2.362
2.358
2.353
2.349
2.346
2.341
2.336
2.332
2.331
2.327
1.407
1.392
O
O
O
Me
N
5
2.12
1.02
1.00
1.11
2.19
1.02
1.07
1.13
1.16
1.16
3.15
0.18
f1 (ppm)

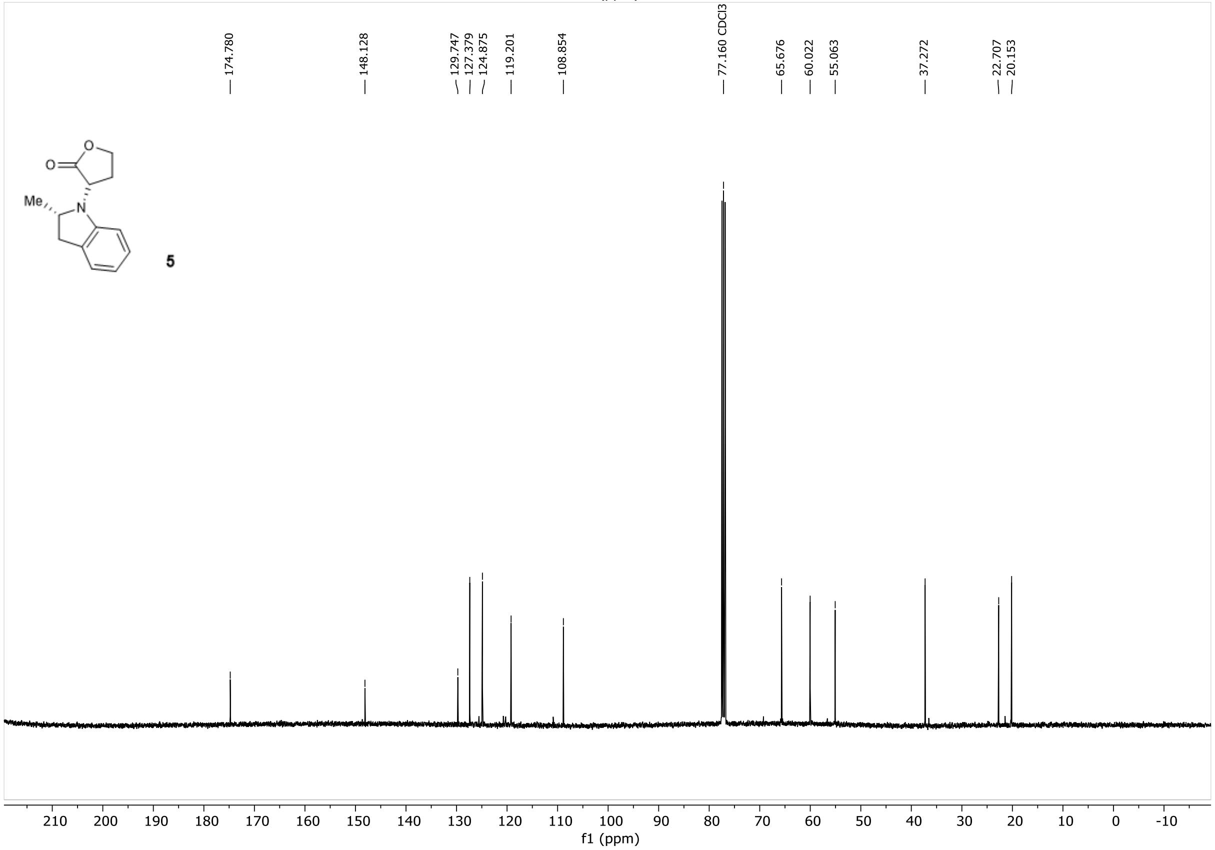
174.780
148.128
129.747
127.379
124.875
119.201
108.854
77.160 CDCl3
65.676
60.022
55.063
37.272
22.707
20.153
O
O
O
Me
N
5
f1 (ppm)

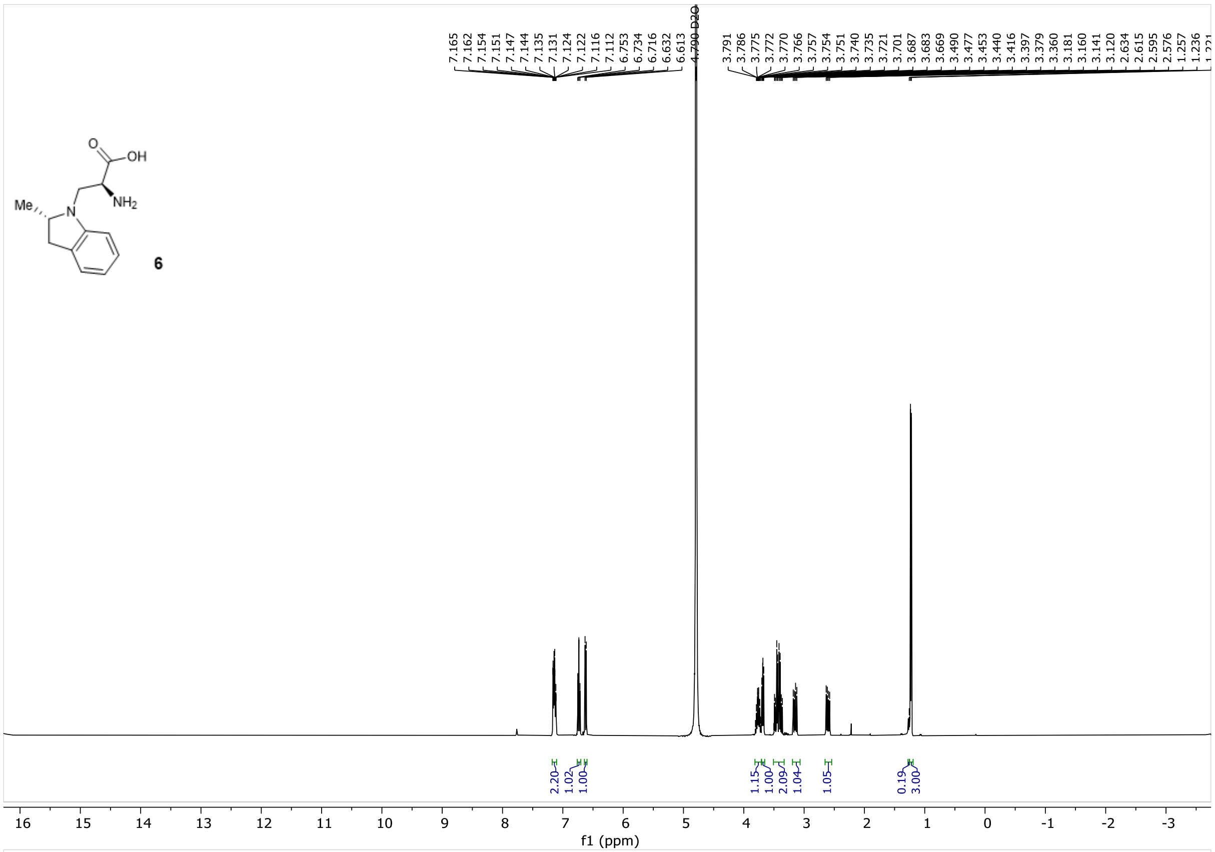

7.165
7.162
7.154
7.151
7.147
7.144
7.135
7.131
7.124
7.122
7.116
7.112
6.753
6.734
6.716
6.632
6.613
4.790 D2O
3.791
3.786
3.775
3.772
3.770
3.766
3.757
3.754
3.751
3.740
3.735
3.721
3.701
3.687
3.683
3.669
3.490
3.477
3.453
3.440
3.416
3.397
3.379
3.360
3.181
3.160
3.141
3.120
2.634
2.615
2.595
2.576
1.257
1.236
OH
NH2
Me
N
6
2.20
1.02
1.00
1.15
1.00
2.09
1.04
1.05
0.19
3.00
f1 (ppm)

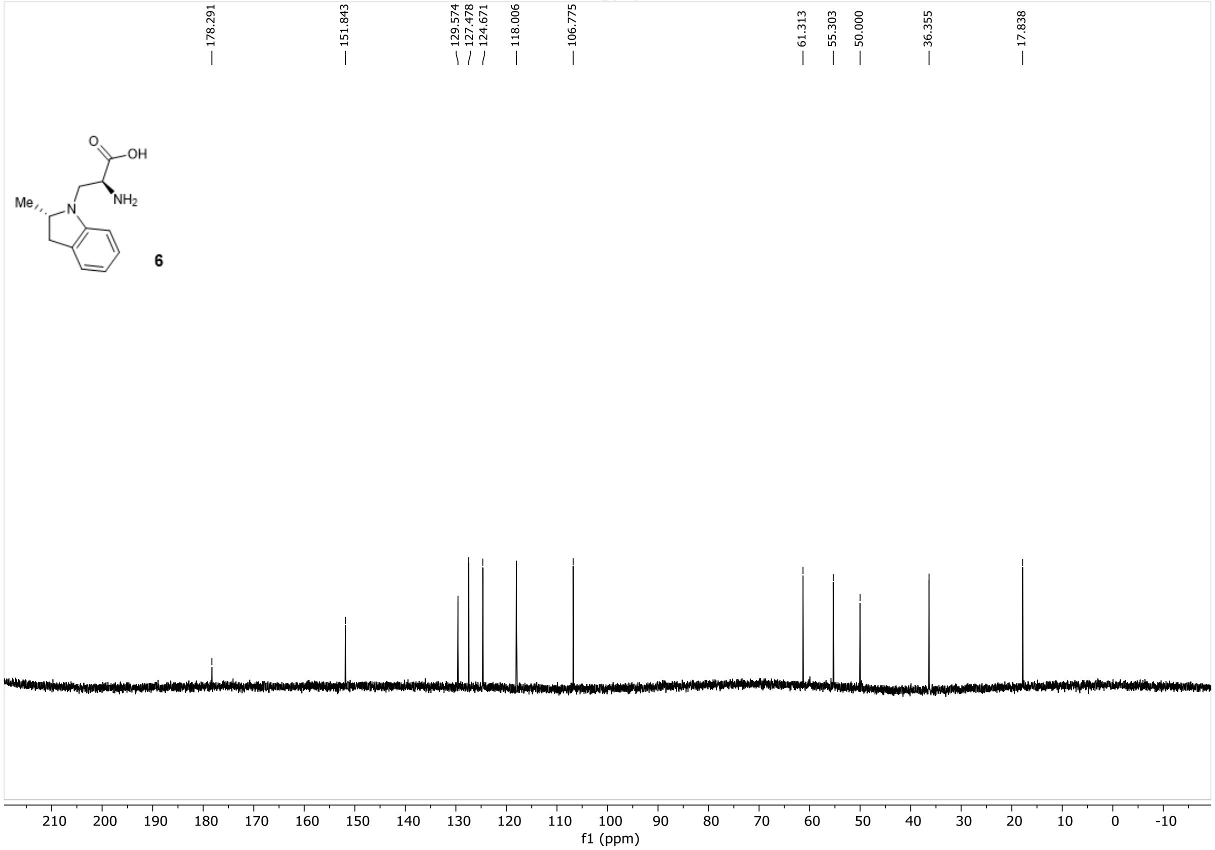

178.291
151.843
129.574
127.478
124.671
118.006
106.775
61.313
55.303
50.000
36.355
17.838
OH
NH2
Me
N
6
f1 (ppm)

XII. References

1. Kille, S.; Acevedo-Rocha, C. G.; Parra, L. P.; Zhang, Z.-G.; Opperman, D. J.; Reetz, M. T.; Acevedo J. P. Reducing codon redundancy and screening effort of combinatorial protein libraries created by saturation mutagenesis. *ACS Synth. Biol.* **2013**, *2*, 83–92.
2. Gibson, D. G.; Young, L.; Chuang, R.-Y.; Venter, J. C.; Hutchinson III, C. A.; Smith, H. O. Enzymatic assembly of DNA molecules up to several hundred kilobases. *Nat. Methods* **2009**, *6*, 343–345.
3. Vatsis, K. P.; Peng, H.-M.; Coon, M. J. Replacement of active-site cysteine-436 by serine converts cytochrome P450 2B4 into an NADPH oxidase with negligible monooxygenase activity. *J. Inorg. Biochem.* **2002**, *91*, 542–553.
4. Seyfried, P.; Heinz, M.; Pintér, G.; Klötzner, D.-P.; Becker, Y.; Bolte, M.; Jonker, H. R. A.; Stelzl, L. S.; Hummer, G.; Schwalbe, H.; Heckel, A. Optimal destabilization of DNA double strands by single-nucleobase caging. *Chem. Eur. J.* **2018**, *24*, 17568–17576.
5. Blair, L. M.; Sperry, J. Palladium-catalyzed heteroannulation approach to 1,2-Bis(3-indolyl)ethanes. *Synlett* **2013**, *24*, 1931–1936.
6. Hardouin, C.; Kelso, M. J.; Romero, F. A.; Rayl, T. J.; Leung, D.; Hwang, I.; Cravatt, B. F.; Boger, D. L. Structure−Activity Relationships of α-Ketooxazole Inhibitors of Fatty Acid Amide Hydrolase. *J. Med. Chem.* **2007**, *50*, 3359–3368.
7. Kuijpers, P. F.; Tiekink, M. J.; Breukelaar, W. B.; Broere, D. L. J.; van Leest, N. P.; van der Vlugt, J. I.; Reek, J. N. H.; de Bruin, B. Cobalt-porphyrin-catalysed intramolecular ring-closing C−H amination of aliphatic azides: a nitrene-radical approach to saturated heterocycles. *Chem. Eur. J.* **2017**, *23*, 7945–7952.
8. Nguyen, Q.; Sun, K.; Driver, T. G. Rh_2(II)-catalyzed intramolecular aliphatic C–H bond amination reactions using Aryl Azides as the N-atom source. *J. Am. Chem. Soc.* **2012**, *134*, 7262–7265.
9. Zhang, H.-Y.; Mao, L.-L.; Yang, B.; Yang, S.-D. Copper-catalyzed radical cascade cyclization for the synthesis of phosphorated indolines. *Chem. Commun.* **2015**, *51*, 4101–4104.
10. Gotor-Fernández V.; Fernández-Torres, P.; Gotor, V. Chemoenzymatic preparation of optically active secondary amines: a new route to enantiomerically pure indolines. *Tetrahedron Asymmetry* **2006**, *17*, 2558–2564.
11. Liu, Z.; Calvó-Tusell, C.; Zhou, A. Z.; Chen, K.; Garcia-Borràs, M.; Arnold, F. H. Dual-function enzyme catalysis for enantioselective carbon-nitrogen bond formation. *Nat. Chem.* **2021**, *13*, 1166–1172.
12. Grimme, S. Exploration of chemical compound, conformer, and reaction space with meta-dynamics simulations based on tight-binding quantum chemical calculations. *J. Chem. Theory Comput.* **2019**, *15*, 2847–2862.
13. Frisch, M. J.; Trucks, G. W.; Schlegel, H. B.; Scuseria, G. E.; Robb, M. A.; Cheeseman, J. R.; Scalmani, G.; Barone, V.; Petersson, G. A.; Nakatsuji, H.; Li, X.; Caricato, M.; Marenich, A.; Bloino, J.; Janesko, B. G.; Gomperts, R.; Mennucci, B.; Hratchian, H. P.; Ortiz, J. V.; Izmaylov, A. F.; Sonnenberg, J. L.; Williams-Young, D.; Ding, F.; Lipparini, F.; Egidi, F.; Goings, J.; Peng, B.; Petrone, A.; Henderson, T.; Ranasinghe, D.; Zakrzewski, V. G.; Gao, J.; Rega, N.; Zheng, G.; Liang, W.; Hada, M.; Ehara, M.; Toyota, K.; Fukuda, R.; Hasegawa, J.; Ishida, M.; Nakajima, T.; Honda, Y.; Kitao, O.; Nakai, H.; Vreven, T.; Throssell, K.; Montgomery, J. A.; Peralta, Jr., J. E.; Ogliaro, F.; Bearpark, M.; Heyd, J. J.; Brothers, E.; Kudin, K. N.; Staroverov, V. N.; Keith, T.; Kobayashi, R.; Normand, J.;

Raghavachari, K.; Rendell, A.; Burant, J. C.; Iyengar, S. S.; Tomasi, J.; Cossi, M.; Millam, J. M.; Klene, M.; Adamo, C.; Cammi, R.; Ochterski, J. W.; Martin, R. L.; Morokuma, K.; Farkas, O.; Foresman, J. B.; Fox, D. J. Gaussian 09, Revision A.02, Gaussian, Inc., Wallingford CT, 2016.

14. Luchini, G.; Alegre-Requena, J. V.; Funes-Ardoiz, I.; Paton, R. S. GoodVibes: automated thermochemistry for heterogeneous computational chemistry data. *F1000Research* **2020**, *9*, 291–305.
15. Case, D. A.; Betz, R. M.; Cerutti, D. S.; Cheatham, III, T. E.; Darden, T. A.; Duke, R. E.; Giese, T. J.; Gohlke, H.; Goetz, A. W.; Homeyer, N.; Izadi, S.; Janowski, P.; Kaus, J.; Kovalenko, A.; Lee, T. S.; LeGrand, S.; Li, P.; Lin, C.; Luchko, T.; Luo, R.; Madej, B.; Mermelstein, D.; Merz, K. M.; Monard, G.; Nguyen, H.; Nguyen, H. T.; Omelyan, I.; Onufriev, A.; Roe, D. R.; Roitberg, A.; Sagui, C.; Simmerling, C. L.; Botello-Smith, W. M.; Swails, J.; Walker, R. C.; Wang, J.; Wolf, R. M.;X. Wu, L. Xiao and P.A. Kollman (2016), AMBER 2016, University of California, San Francisco.)
16. Søndergaard, C. R.; Olsson, M. H. M.; Rostkowski, M.; Jensen, J. H. Improved treatment of ligands and coupling effects in empirical calculation and rationalization of pKa values. *J. Chem. Theory Comput.* **2011**, *7*, 2284–2295.
17. Maier, J. A.; Martinez, C.; Kasavajhala, K.; Wickstrom, L.; Hauser, K. E.; Simmerling, C. ff14SB: Improving the accuracy of protein side chain and backbone parameters from ff99SB. *J. Chem. Theory Comput.* **2015,** *11*, 3696–3713.
18. Jorgensen, W. L., Chandrasekhar, J., Madura, J. D., Impey, R. W.; Klein, M. L. Comparison of simple potential functions for simulating liquid water. *J. Chem. Phys.* **1983**, *79***,** 926–935.
19. Li, P.; Jr. Merz, K. M. MCPB.py: a python based metal center parameter builder. *J. Chem. Inf. Model.* **2016**, *56*, 599–604.
20. (a) Bayly, C. I., Cieplak, P., Cornell, W. D.; Kollman, P. A. A well-behaved electrostatic potential based method using charge restraints for deriving atomic charges: the RESP model. *J. Phys. Chem.* **1993**, *97*, 10269–10280. (b) Besler, B. H., Merz, K. M.; Kollman, P. A. Atomic charges derived from semiempirical methods. *J. Comput. Chem.* **1990**, *11*, 431–439. (c) Singh, U. C.; Kollman, P. A. An approach to computing electrostatic charges for molecules. *J. Comput. Chem.* **1984**, *5*, 129–145.
21. Darden, T., York, D.; Pedersen, L. Particle mesh Ewald: An N-log(N) method for Ewald sums in large systems. *J. Chem. Phys.* **1993**, *98*, 10089–10092.
